# Novel Organoselenium Redox Modulators with Potential Anticancer, Antimicrobial, and Antioxidant Activities

**DOI:** 10.3390/antiox11071231

**Published:** 2022-06-23

**Authors:** Marwa Sak, Yasair S. Al-Faiyz, Hany Elsawy, Saad Shaaban

**Affiliations:** 1 Chemistry Department, College of Science, King Faisal University, Al-Ahsa 31982, Saudi Arabia; 218001929@student.kfu.edu.sa (M.S.); yalfayz@kfu.edu.sa (Y.S.A.-F.); hmostafa@kfu.edu.sa (H.E.); 2 Chemistry Department, Faculty of Science, Tanta University, Tanta 6620010, Egypt; 3 Chemistry Department, Faculty of Science, Mansoura University, Mansoura 35516, Egypt

**Keywords:** anticancer, antimicrobial, antioxidant, organoselenium, redox modulators

## Abstract

Novel organic selenides were developed in good yields (up to 91%), and their chemical entities were confirmed by IR, MS, and ^1^H- and ^13^C-NMR spectroscopy. Their anticancer and antimicrobial properties were estimated against different human cancer (MCF-7 and HepG2) and healthy (WI-38) cell lines, as well as several microbial strains (*Escherichia coli*, *Staphylococcus aureus*, and *Candida albicans*). Furthermore, the 2,2-diphenyl-1-picrylhydrazyl (DPPH) and 2,2′-azino-bis(3-ethylbenzothiazoline-6-sulfonic acid) (ABTS) bioassays were used for the estimation of the antioxidant activities. Generally, cytotoxicity results were more pronounced against the MCF-7 cells than HepG2 cells. Compound 2-((4-((1-hydroxynaphthalen-2-yl)diazenyl)phenyl)selanyl)-*N*-phenylacetamide (**9**) was the most cytotoxic, even more than doxorubicin, with IC_50_ of 3.27 ± 0.2 against 4.17 ± 0.2 µM and twelve-times more selective, respectively. Interestingly, compound **9** exhibited similar antimicrobial potential to reference antibacterial and antifungal drugs and comparable antioxidant activity to vitamin C. These results point to selective cytotoxicity against MCF-7 cells and interesting antimicrobial and antioxidant properties of some newly synthesized organic selenides, which in turn needs further in vitro studies.

## 1. Introduction

Selenium (Se), a non-metal trace element, belongs to the chalcogen family and has an essential role in the immune system protection and the growth suppression of different tumors [1,2,3]. Unsurprisingly, Se deficiency is implicated with the development of various diseases such as autoimmune disorders [4,5]. Furthermore, the function of various redox enzymes such as glutathione peroxidase (GPx), thioredoxin reductases, and iodothyronine deiodinases depends on the Se redox center [6,7]. Generally, organoselenium (OSe) compounds are less toxic than inorganic Se compounds [5,8,9]. The former compounds have recently gained significant attention due to their potential pharmacological properties [8,9,10].

The last decade has witnessed an increasing concern in the synthesis of OSe compounds due to their role in the modulation of oxidative stress related diseases [3,11]. Within this context, OSe compounds might act as antioxidants or pro-oxidants, depending on their environment [12,13,14]. They do not alter the redox balance, but their effectiveness relies on the intracellular redox state [13,14]. The relative nucleophilic character of OSe compounds accounts for their apparent antioxidant properties that usually arise in normal cells [15,16]. On the other hand, OSe compounds switch to prooxidants in cells rich with reactive oxygen species (ROS), such as in the case of several cancer cells (e.g., colon, liver, and breast) [17,18]. In this case, OSe compounds use the pre-existing ROS (e.g., H_2_O_2_) as their substrates and enhance their reaction with different cellular compartments (e.g., endoplasmic reticulum, DNA) and redox-sensible proteins [17,19,20,21,22]. The modification/oxidation of these proteins triggers apoptotic cell death [23,24]. 

**Figure 1:**
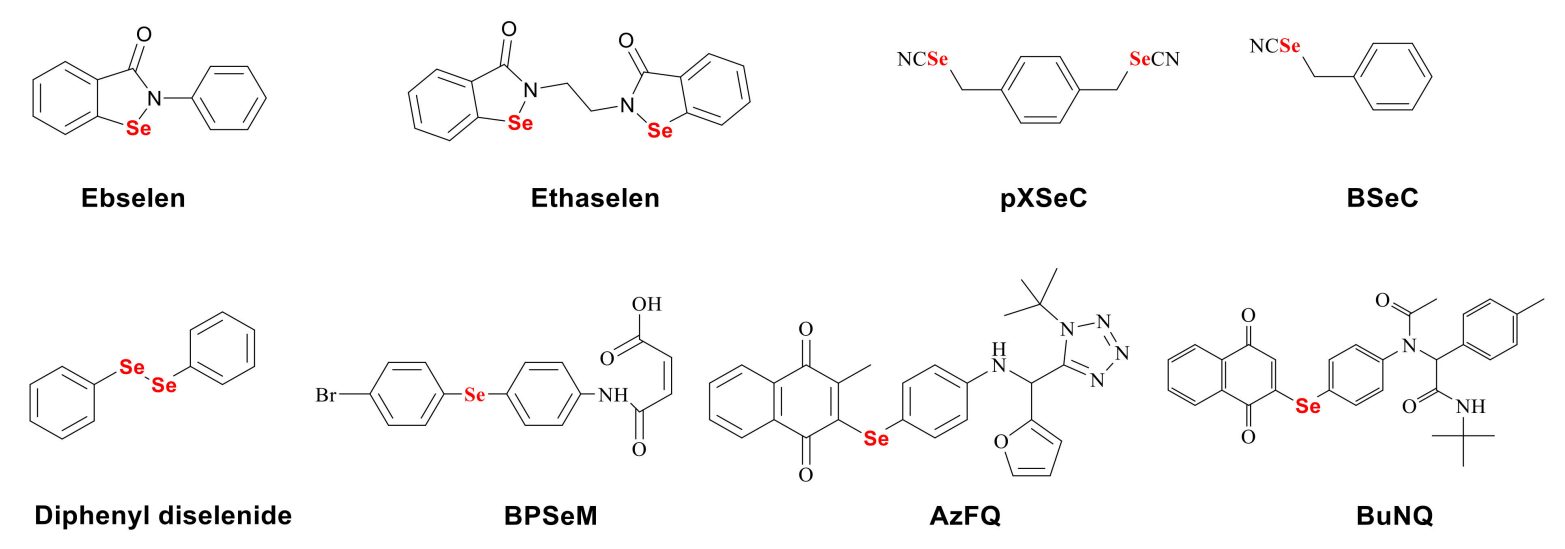
Structure of pharmacologically active OSe compounds.

In this context, ebselen is the most studied OSe heterocycle, with interesting GPx-like, anti-inflammatory, and antioxidant properties. It has recently entered clinical trials in phases two and three as the possible therapy of different bipolar disorders and neurodegenerative diseases (Figure 1) [25]. Furthermore, ethaselen has also reached clinical phase one as a potential anticancer drug in vivo model. Moreover, benzyl selenocyanate (BSeC)and p-xyleneselenocyanate (pXSeC) manifested interesting chemoprotective activities against different cancer models, including lung, liver, and colon cancers (Figure 1) [26]. Finally, diphenyl diselenide has shown excellent GPx-like and antinociceptive agents [27,28].

We recently reported several OSe *N*-substituted maleanilic acids (e.g., 4-((4-((4-bromophenyl)selanyl)phenyl)amino)-4-oxobut-2-enoic acid (BPSeM)) with potential anti-apoptotic properties in oligodendrocytes [11,17,21,29]. Additionally, the OSe compounds 2-((4-(((1-(*tert*-butyl)-1H-tetrazol-5-yl)(furan-2-yl)methyl)amino)phenyl)selanyl)-3-methylnaphthalene-1,4-dione (AzFQ) and *N*-(tert-butyl)-2-(N-(4-((1,4-dioxo-1,4-dihydronaphthalen-2-yl)selanyl)phenyl)acetamido)-2-(p-tolyl)acetamide (BuNQ) showed interesting anti-hepatocellular carcinoma properties by the downregulation of the Bcl-2 and Ki-67 levels and activation of caspase-8 [28,29]. Therefore, our aim was to synthesize OSe compounds and evaluate their biological activities.

In this context, twenty-one types of OSe compounds were synthesized in good yields (up to 91%). Their anticancer and antimicrobial properties were assessed against different human cancer cell lines, as well as several microbial strains. Furthermore, their corresponding antioxidant activities were also evaluated.

## 2. Experimental

### 2.1. Material and Methods

Melting points were measured on the Gallenkamp instrument in degrees centigrade (uncorrected). Elemental analyses were performed at Cairo University. The IR spectra (KBr, ύ cm^−1^) were recorded at King Faisal University on a Mattson 5000 FTIR Spectrophotometer. The Mass spectra were measured at Cairo University on a GC-MS-QP-100 EX Shimadzu instrument. The ^1^H NMR and the ^13^C NMR (100 MHz) spectra were measured at Mansoura University using a Varian Spectrophotometer at 400 MHz, employing the TMS internal reference and DMSO-*d*_6_ as the solvent. The chemical shifts (*δ*) in parts per million were recorded to the residual peak of solvents. Biological experiments were carried out at Mansoura University, Faculty of Pharmacy. Compounds number **1** and **2** were prepared according to our literature procedures [30,31,32]. Additionally, the structure of 4-(methylselanyl)aniline (**3**) was confirmed by the preparation of an authentic sample, according to the reported literature procedure of Fang, Xiao-Li, et al. [33].

### 2.2. The Biological Assays

#### 2.2.1. The Anticancer Activity

Cells were obtained from the ATCC Company (VACSERA), Cairo, Egypt. MTT assay were performed according to the literature method [10,24,25,34]. More details are in the Supporting Information.

#### 2.2.2. The Antimicrobial Activity

The antimicrobial activities of the OSe compounds were evaluated against *C. albicans* yeast, as well as *E. coli* and *S.* bacteria, employing the agar well diffusion assay [31]. Furthermore, the MICs (in µM) were determined by the microdilution method according to the reported protocol [10,24,25,34]. More details are in the Supporting Information.

#### 2.2.3. The Antioxidant Activity

The antioxidant activity was estimated by ABTS and DPPH bioassays, according to the reported literature method [28,29]. More details are in the Supplementary Materials.

### 2.3. Chemistry

General procedure I: Preparation of OSe compounds **4**, **5**, **6**, and **3** via the reduction of diselenide **2** and subsequent SN reaction:

Amine **2** (1 mmol), NaOH (40 mg, 1 mmol), and halo derivatives (2.2 mmol) were mixed in EtOH (15 mL). NaBH_4_ (189.15 mg, 5 mmol) was then added portion-wise over 30 min. The reaction was then stirred for an additional 30 min. The organic layer was dried and evaporated under vacuum, and the residue was recrystallized from a suitable solvent (see the individual procedures).

General procedure II: Preparation of OSe azo dyes **7** and **8**:

Method A (for the synthesis of **9**)

Amine **4** (1.00 mmol) was dissolved in aqueous AcOH (8 mL, 1:1) and cooled to 0–5 °C. NaNO_2_ (0.7 g, 10 mmol, in 3 mL H_2_O) was added slowly and portion-wise while keeping the temperature at 0–5 °C. The diazonium salt solution obtained was added to a cooled and stirred mixture of the aromatic compound (1.2 mmol) dissolved in 20 mL of 10% NaOH solution. Stirring was continued for 1.5 h. The resulting precipitate was collected, washed with water, and recrystallized from EtOH.

Method B (for the preparation of **7** and **8**)

Amine **4** (1.00 mmol) was dissolved in aqueous AcOH (8 mL, 1:1) and cooled to 0–5 °C. NaNO_2_ (0.7 g, 10 mmol, in 3 mL H_2_O) was added slowly and portion-wise while keeping the temperature at 0–5 °C. The diazonium salt solution formed was added to a cooled mixture of the active methylene/heterocycle (1.00 mmol) and NaOAc (2.0 g), dissolved in (10 mL of 50% aqueous EtOH). Stirring was continued for 1.5 h. The resulting residue was collected, washed with water, and recrystallized from ethanol.

General procedure III: The preparation of amide-acids 10, 11, 15, and 16:

Amine **4** (1 mmol) was dissolved in toluene (5 mL), and anhydride (1 mmol) was added. Stirring was continued for three h. The formed precipitate was washed with toluene.

General procedure IV: The synthesis of cyclic imides **13** and **14**:

A mixture was created of appropriate amide-acid (1mmol), NaOAc (100 mg), and Ac_2_O (3 mL). The mixture was gently heated for 2 h at 50–60 °C. The reaction was cooled, ice water was then added, and the solid was separated and recrystallized from EtOH.

The preparation of cyclic imides **12:**

A mixture of amine **4** (1.00 mmol) and phthalic anhydride (1 mmol) dissolved in AcOH (5 mL) was refluxed for 10 h. The formed precipitate was separated by filtration and washed several times with H_2_O and EtOH.

### 2.4. Synthesis of 2-((4-aminophenyl)selanyl)-N-phenylacetamide (**4**)

Compound **4** was synthesized following general procedure I using amine **2** (342 mg, 1 mmol) and 2-chloro-*N*-phenylacetamide (322.27 mg, 1.9 mmol). The reaction was monitored by TLC (CH_2_Cl_2_:MeOH, 10:1), Rf = 0.48, brown crystals, yield: 287.6 mg (94%), MP = 121–122 °C. IR (KBr) λ_max_.cm^−1^ 3385, 3321, 2991, 1638, 1589, 1526, 1488, 1277; ^1^HNMR (400 MHz, DMSO-d_6_) δ 9.97 (s, 1H, NH), 7.53 (d, *J* = 8.0 Hz, 2H, Ar-H), 7.35–7.19 (m, 4H, Ar-H), 7.04 (t, *J* = 7.4 Hz, 1H, Ar-H), 6.54–6.45 (m, 2H, Ar-H), 5.29 (s, 2H, NH_2_), 3.50 (s, 2H, SeCH_2_). ^13^CNMR (101 MHz, DMSO-d_6_) δ 168.34, 149.00, 139.09, 135.74, 128.67, 123.18, 119.04, 114.45, 112.43, 31.96; MS (EI, 70 ev) *m*/*z* (%) = 306 (M^+^,39.28), 213 (2.21), 185 (12.05), 172 (79.63), 145 (8.62), 120 (62.62), 106 (100.0, base peak), 93 (78.91).

### 2.5. Synthesis of 2-((4-aminophenyl)selanyl)-N-(4-ethoxyphenyl)acetamide (**5**)

Compound **5** was synthesized following general procedure I using amine **2** (342 mg, 1 mmol) and 2-chloro-*N*-(4-ethoxyphenyl)acetamide (427.32 mg, 2 mmol). The reaction was monitored by TLC (CH_2_Cl_2_:MeOH, 10:1), Rf = 0.54, white powder, yield: 310.7 mg (89 %), MP = 136–137 °C, IR (KBr):λ_max_.cm^−1^ 3382, 3266, 2979, 1638, 1590, 1508, 1487. ^1^HNMR (400 MHz, DMSO-d_6_) δ 9.82 (s, 1H, NH), 7.42 (s, 2H, Ar-H), 7.24 (s, 2H, Ar-H), 6.86 (s, 2H, Ar-H), 6.49 (s, 2H, Ar-H), 5.30 (s, 2H, NH_2_), 3.98 (d, *J* = 4.8 Hz, 2H, OCH_2_), 3.36 (s, 2H, SeCH_2_), 1.31 (t, *J* = 6.9 Hz, 3H, CH_3_). ^13^C NMR (101 MHz, DMSO) δ 167.76, 154.43, 148.93, 135.70, 132.14, 120.59, 114.47, 114.31, 112.55, 63.03, 31.91, 14.61. MS (EI, 70 ev) *m*/*z* (%) = 350 (M^+^,70.45), 348 (35.47), 257 (2.36), 172 (15.54), 137 (45.58), 122 (17.76), 108 (100.0, base peak), 93 (20.48).

### 2.6. Synthesis of (2-((4-aminophenyl)selanyl)acetyl)tryptophan (**6**)

Compound **(6)** was synthesized following general procedure I from amine **2** (342 mg, 1 mmol) and. (2-chloroacetyl)tryptophan (561.42 mg, 2 mmol). The reaction was monitored by TLC (CH_2_Cl_2_:MeOH, 10:1), Rf = 0.25, white solid, yield: 370.5 mg (89%), MP = 154–156 °C, IR (KBr):λ_max_.cm^−1^ = 3336, 2955, 2926, 1716, 1490; ^1^H NMR (400 MHz, DMSO-d_6_) δ 10.62 (s, 1H, OH), 7.99 (s, 1H, NH), 7.30 (d, *J* = 7.9 Hz, 1H, Ar-H), 7.11 (m, 1H, Ar-H), 6.94 (dd, *J* = 8.4, 1.9 Hz, 5H, Ar-H), 6.83 (s, 1H, =CH), 6.73 (s, 1H, NH), 4.21 (t, *J* = 2.1 Hz, 1H, CH), 3.19 (dt, *J* = 6.9, 1.7 Hz, 2H, CH_2_), 3.19 (s, 2H, SeCH_2_), 2.95 (m, 2H, NH_2_), 2.26 (s, 1H, NH). ^13^C NMR (101 MHz, DMSO-d_6_) δ 173.26, 169.10, 148.73, 136.03, 135.38, 127.24, 123.58, 120.84, 118.31, 118.19, 114.48, 112.91, 111.29, 109.70, 58.94, 24.20, 22.58. MS (EI, 70 ev) *m*/*z* (%) = 417 (M^+^,0.07), 363 (1.17), 347 (0.36), 317 (1.55), 3 305 (5.41), 247 (3.34), 189 (3.13), 143 (4.39), 117 (38.20), 101 (21.56), 59 (100.0, base peak).

### 2.7. Synthesis of 4-((2-oxo-2-(phenylamino)ethyl)selanyl)phenyl)carbonohydrazonoyl dicyanide (**7**)

Compound **7** was synthesized following general procedure II from 2-((4 aminophenyl)selanyl)-*N*-phenylacetamide (**4**) (305.24 mg, 1.00 mmol) and malononitrile (79.27 mg, 1.2 mmol).The product formation was followed by TLC: [pet. ether/ethyl acetate (4:4)]; Rf = 0.6, red powder; yield = 313.05 mg (82%); MP = 177–179 °C; IR (KBr):λ_max_.cm^−1^ = 3295, 3226, 3181, 2247, 1656, 1598, 1530, 1489. ^1^H NMR (400 MHz, DMSO-d_6_) δ 13.03 (s, 1H, NH), 10.18 (s, 1H, NH), 7.64 (dd, *J* = 8.6, 2.1 Hz, 2H, Ar-H), 7.55 (d, *J* = 7.0 Hz, 2H, Ar-H), 7.42 (dd, *J* = 8.6, 2.2 Hz, 2H, Ar-H), 7.31 (t, *J* = 6.8 Hz, 2H, Ar-H), 7.06 (t, *J* = 7.3 Hz, 1H, Ar-H), 3.77 (s, 2H, SeCH_2_). ^13^C NMR (101 MHz, DMSO-d_6_) δ 168.33, 140.85, 139.10, 133.67, 129.11, 127.38, 119.54, 117.61, 114.71, 110.34, 85.32, 30.96. MS (EI, 70 ev) *m*/*z* (%) = 383(M^+^, 6.12), 287 (2.03), 268 (2.12), 249 (2.57), 189 (3.93), 170 (4.64), 131 (6.66), 113 (23.22), 101 (17.52), 93 (100.0, base peak), 59 (45.76).

### 2.8. Synthesis of 2-((4-(2-(3-methyl-5-oxo-1-phenyl-1H-pyrazol-4(5H)-ylidene)hydrazinyl)phenyl)selanyl)-N-phenylacetamide (**8**)

Compound **8** was synthesized following general procedure II from 2-((4 aminophenyl)selanyl)-*N*-phenylacetamide (**4**) (305.24 mg, 1.00 mmol) and 5-methyl-2-phenyl-1,2-dihydro-3*H*-pyrazol-3-one (174.08 mg, 1.00 mmol). The product formation was followed by TLC: [pet. ether/ethyl acetate (4:4)]; Rf = 0.6, orange powder; yield = 173.9 mg (39%); MP = 197–199 °C; IR (KBr): λmax.cm^−1^ = 3277, 3064, 1644, 1551, 1498, 1443, 1257. ^1^H NMR (400 MHz, DMSO-d_6_) δ 13.24 (s, 1H, NH), 10.18 (s, 1H, NH), 7.92 (d, *J* = 6.5 Hz, 3H, Ar-H), 7.64 (m, 2H, Ar-H), 7.56 (m, 3H, Ar-H), 7.45 (m, 2H, Ar-H), 7.31 (m, 2H, Ar-H), 7.21 (d, 1H, Ar-H), 7.06 (d, 1H, Ar-H), 2.52 (s, 2H, SeCH_2_), 2.28 (s, 3H, CH_3_). ^13^C NMR (101 MHz, DMSO) δ 168.44, 156.97, 148.94, 141.02, 139.46, 138.40, 133.59, 129.47, 129.26, 128.39, 127.21, 125.25, 123.88, 119.55, 118.10, 117.37, 31.06, 12.09. MS (EI, 70 ev) *m*/*z* (%) = 491 (M^+^, 13.84), 305 (14.23), 259 (12.22), 247 (16.20), 201 (14.70), 189 (22.24), 159 (15.92), 131 (16.17), 113 (63.35), 103 (32.46), 59 (100.0, base peak).

### 2.9. Synthesis of 2-((4-(2-(3-hydroxynaphthalen-1(4H)-ylidene)hydrazinyl)phenyl)selanyl)-N-phenylacetamide (**9**)

Compound **9** was synthesized following general procedure II from 2-((4 aminophenyl)selanyl)-*N*-phenylacetamide (**9**) (305.24 mg, 1.00 mmol) and 2-naphthol (172.87 mg, 1.2 mmol).The product formation was followed by TLC: [pet. ether/ethyl acetate (4:4)]; Rf = 0.7, red powder; yield = 271.19 mg (59%); MP = 77–78 °C; IR (KBr): λ_max_.cm^−1^ = 3259, 3052, 1731, 1600, 1630, 1512, 1466. ^1^HNMR (400 MHz, DMSO-d_6_) δ 15.62 (s, 1H, Ar-H), 10.23 (s, 1H, NH), 9.73 (s, 1H, OH), 8.5 (d, 1H, Ar-H), 7.86–7.80 (m, 1H, Ar-H), 7.77–7.75 (m, 3H, Ar-H), 7.69–7.57 (m, 2H, Ar-H), 7.48 (t, *J* = 7.5 Hz, 3H, Ar-H), 7.27 (d, *J* = 7.6 Hz, 5H, Ar-H), 2.51 (s, 2H, SeCH_2_); ^13^CNMR (101 MHz, DMSO-d_6_) δ 167.27, 155.24, 134.56, 132.45, 129.25, 128.77, 127.68, 127.49, 126.06, 125.94, 122.59, 119.82, 119.07, 118.56, 108.60, 30.28; MS (EI, 70 ev) *m*/*z* (%) = 305 (M+-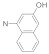, 1.59), 247 (2.55), 201 (1.73), 189 (4.80), 159 (5.09), 117 (39.43), 113 (41.62), 101 (22.20), 87 (21.78), 59 (100.0, base peak).

### 2.10. Synthesis of 4-oxo-4-((4-((2-oxo-2-(phenylamino)ethyl)selanyl)phenyl)amino)but-2-enoic acid (**10**)

Compound **10** was synthesized following general procedure III from 2-((4 aminophenyl)selanyl)-*N*-phenylacetamide (**4**) (305.24 mg, 1.00 mmol) and maleic anhydride (177 mg, 1.8 mmol).The product formation was followed by TLC: [pet. ether/ethyl acetate (4:4)]; Rf = 0.16, yellow powder; yield = 312 mg (77%); MP = 171–172 °C; IR (KBr):λ_max_.cm^−1^ = 3268, 3067, 1710, 1623, 1589, 1538. ^1^H NMR (400 MHz, DMSO-d_6_) δ 12.98 (s, 1H, OH), 10.47 (s, 1H, NH), 10.12 (s, 1H, NH), 7.70–7.45 (m, 6H, Ar-H), 7.41–7.19 (m, 2H, Ar-H), 7.05 (t, *J* = 7.4 Hz, 1H, Ar-H), 6.48 (dd, *J* = 12.0, 1.3 Hz, 1H, CH=), 6.40–6.22 (dd, *J* = 12.0, 1.3 Hz, 1H, CH=), 3.70 (s, 2H, SeCH_2_). ^13^C NMR (101 MHz, DMSO-d_6_) δ 168.01, 166.73, 163.34, 138.95, 137.95, 133.11, 133.02, 131.56, 130.59, 130.34, 128.73, 123.85, 123.35, 120.21, 120.11, 119.07, 30.66. MS (EI, 70 ev) *m*/*z* (%) = 306 (M^+^-COCH=CHCOOH, 27.30), 151 (4.82), 172 (36.97), 159 (5.86), 143 (7.64), 131 (9.36), 113 (39.24), 106 (33.68), 93 (35.18), 59 (100.0, base peak).

### 2.11. Synthesis of 4-oxo-4-((4-((2-oxo-2-(phenylamino)ethyl)selanyl)phenyl)amino)butanoic acid (**11**)

Compound **11** was synthesized following general procedure III from 2-((4 aminophenyl)selanyl)-*N*-phenylacetamide (**4**) (305.24 mg, 1.00 mmol) and succinic anhydride (179, 4 mg, 1.8 mmol). The product formation was followed by TLC: [pet. ether/ethyl acetate (4:4)]; Rf = 0.23, yellow powder; yield = 278.4 mg (69%); MP = 163–165 °C; IR (KBr):λ_max_.cm^−1^ = 3335, 3278, 3253, 1710, 1651, 1549, 1527, 1442. ^1^H NMR (400 MHz, DMSO-d_6_) δ 12.17 (s, 1H, OH), 10.10 (d, *J* = 8.3 Hz, 1H, NH), 10.03 (s, 1H, NH), 7.75–7.42 (m, 6H, Ar-H), 7.39–7.23 (m, 2H, Ar-H), 7.05 (t, *J* = 7.4 Hz, 1H, Ar-H), 2.57 (dd, *J* = 9.8, 4.6 Hz, 2H,SeCH_2_), 2.53 (d, *J* = 5.3 Hz, 2H, CH_2_), 2.43 (s, 2H, CH_2_). ^13^C NMR (101 MHz, DMSO-d_6_) δ 173.57, 170.16, 168.02, 138.95, 138.75, 133.27, 128.72, 123.32, 122.49, 119.61, 119.53, 119.05, 31.01, 30.87, 28.76. MS (EI, 70 ev) *m*/*z* (%) = 406 (M^+^,0.14), 305 (1.98), 259 (2.94), 247 (3.37), 189 (4.75), 175 (2.00), 131 (7.20), 113 (34.40), 101 (23.40), 59 (100.0, base peak).

### 2.12. Synthesis 2-((4-(1,3-dioxoisoindolin-2-yl)phenyl)selanyl)-N-phenylacetamide (**12**)

A mixture of 2-((4-aminophenyl)selanyl)-*N*-phenylacetamide (**4**) (305.24 mg, 1.00 mmol) and (148 mg, 1 mmol) phthalic anhydride was stirred under reflux in acetic acid for 6–10 h. The solution was poured into water, and the resulting precipitate filtered off and recrystallized from ethanol. The product formation was followed by TLC: (CH_2_Cl_2_:MeOH, 10:1); Rf = 0.75; brown powder; yield = 184.7 mg (42%); MP = 261–262 °C; IR (KBr): λmax.cm^−1^ = 3286, 1784, 1701, 1658, 1598, 1494, 1322. ^1^H NMR (400 MHz, DMSO-d6) δ 10.2 (s, 1H, NH), 7.93 (s, 4H, Ar-H), 7.74 (s, 2H, Ar-H), 7.58 (s, 2H, Ar-H), 7.33 (s, 4H, Ar-H), 7.33 (s, 1H, Ar-H), 7.08 (s, 1H, Ar-H), 3.74 (s, 2H, SeCH_2_). ^13^C NMR (101 MHz, DMSO-d_6_) δ 168.46, 167.40, 139.36, 135.22, 132.14, 132.03, 131.14, 130.70, 129.29, 128.47, 123.93, 119.60, 30.66; MS (EI, 70 ev) *m*/*z* (%) = 436 (M^+^, 8.44), 343 (2.01), 315 (5.83), 250 (3.15), 130 (7.29), 117 (11.10), 106 (28.92), 93 (55.05), 76 (38.12), 59 (100.0, base peak).

### 2.13. Synthesis of 2-((4-(2,5-dioxo-2,5-dihydro-1H-pyrrol-1-yl)phenyl)selanyl)-N-phenylacetamide (**13**)

Compound **13** was synthesized following general procedure IV from 4-oxo-4-((4-((2-oxo-2-(phenylamino)ethyl)selanyl)phenyl)amino)but-2-enoic acid **(10)** (404.03 mg, 1.00 mmol) and acetic anhydride (3 mL). The product formation was followed by TLC: (CH_2_Cl_2_:MeOH, 10:1), Rf = 0.53, yellow powder; yield = 227.2 mg (59 %); MP = 169–170 °C; IR (KBr): λ_max_.cm^−1^ = 3249, 3095, 1699, 1647, 1492, 1392, 1148. ^1^H NMR (400 MHz, DMSO-d_6_) δ 10.20 (s, 1H, NH), 7.81–7.65 (m, 2H, Ar-H), 7.55 (t, *J* = 7.7 Hz, 3H, Ar-H), 7.31 (ddt, *J* = 10.9, 5.5, 3.4 Hz, 4H, Ar-H), 7.19 (d, *J* = 1.7 Hz, 2H, Ar-H), 7.06 (t, *J* = 7.4 Hz, 1H, Ar-H), 3.8 (s, 2H, SeCH_2_). ^13^C NMR (101 MHz, DMSO- d_6_) δ 169.76, 167.90, 138.91, 134.69, 131.80, 130.36, 129.67, 128.76, 127.33, 123.41, 119.09, 30.21. MS (EI, 70 ev) *m*/*z* (%) = 386 (M^+^,14.25), 305 (1.99), 265 (7.20), 200 (4.69), 186 (10.57), 143 (10.09), 131 (14.13), 113 (50.67), 101 (28.02), 93 (100.0, base peak), 77 (14.39).

### 2.14. Synthesis of 2-((4-(2,5-dioxopyrrolidin-1-yl)phenyl)selanyl)-N-phenylacetamide (**14**)

Compound **14** was synthesized following general procedure IV from 4-oxo-4-((4-((2-oxo-2-(phenylamino)ethyl)selanyl)phenyl)amino)butanoic acid **(11)** (406.04 mg, 1.00 mmol) and acetic anhydride (3 mL). The product formation was followed by TLC: (CH_2_Cl_2_:MeOH, 10:1), Rf = 0.64, brown powder; yield = 204.10 mg (53%); MP = 159–161 ^0^C; IR (KBr): λmax.cm^−1^ = 3301, 2894, 1777, 1695, 1526, 1491, 1441, 1173. ^1^H NMR (400 MHz, DMSO-d_6_) δ 10.22 (s, 1H, NH), 7.80–7.74 (m, 1H, Ar-H), 7.70 (d, *J* = 7.4 Hz, 2H, Ar-H), 7.57 (d, *J* = 7.1 Hz, 2H, Ar-H), 7.32 (s, 2H, Ar-H), 7.22 (d, *J* = 7.5 Hz, 2H, Ar-H), 7.07 (s, 1H, Ar-H), 3.79 (s, 2H, SeCH_2_), 2.79 (s, 4H, CH_2_CH_2_). ^13^C NMR (101 MHz, DMSO-d_6_) δ 177.19, 168.42, 139.41, 132.06, 131.93, 131.63, 130.76, 129.28, 128.36, 128.20, 123.93, 119.59, 30.47, 28.74. MS (EI, 70 ev) *m*/*z* (%) = 388 (M^+^, 12.20), 295 (1.36), 254 (10.98), 226 (2.34), 188 (3.82), 172 (16.02), 117 (23.00), 106 (33.47), 93 (100.0, base peak), 77 (21.35).

### 2.15. Synthesis of 4-((4-(methylselanyl)phenyl)amino)-4-oxobut-2-enoic acid (**15**)

Compound **15** was synthesized following general procedure III from 4-(methylselanyl)aniline (**3**): (186 mg, 1.00 mmol) and maleic anhydride (177 mg, 1.8 mmol). The product formation was followed by TLC: CH_2_Cl_2_: MeOH (9.50:0.50), Rf = 0.45, yellow powder; yield = 190.5 mg (66.86 %); MP = 171 °C; IR (KBr): λ_max_.cm^−1^ = 3338, 3262, 3080, 2929, 1701, 1632, 1487, 1392. ^1^H NMR (400 MHz, DMSO) δ 13.01 (s, 1H, OH), 10.42 (s, 1H, NH), 7.62–7.54 (m, 2H, Ar-H), 7.39 (dd, *J* = 8.7, 2.0 Hz, 2H, Ar-H), 6.47 (dd, *J* = 12.1, 1.8 Hz, 1H, =CH), 6.31 (dd, *J* = 12.1, 1.8 Hz, 1H, =CH), 2.33 (d, *J* = 1.8 Hz, 3H, CH_3_). ^13^C NMR (101 MHz, DMSO) δ 166.81, 163.13, 136.81, 131.54, 130.42, 125.80, 120.27, 6.94.

### 2.16. Synthesis of 4-((4-(methylselanyl)phenyl)amino)-4-oxobutanoic acid (**16**)

Compound **16** was synthesized following general procedure III from 4-(methylselanyl)aniline (**3**) (186 mg, 1.00 mmol) and succinic anhydride (179, 4 mg, 1.8 mmol). The product formation was followed by TLC: CH_2_Cl_2_: MeOH (9.50:0.50), Rf = 0.28, yellow powder; yield = 219.5 mg (76.5%); MP = 169.6 °C; IR (KBr): λ_max_.cm^−1^ = 3278, 2919, 1690, 1650, 1590, 1527, 1187. ^1^H NMR (400 MHz, DMSO) δ 12.16 (s, 1H, OH), 9.99 (s, 1H, NH), 7.54 (d, *J* = 8.6 Hz, 2H, Ar-H), 7.36 (d, *J* = 8.6 Hz, 2H, Ar-H), 2.54 (d, *J* = 4.6 Hz, 2H, CH_2_), 2.52–2.37 (m, 2H, CH_2_), 2.31 (d, *J* = 5.1 Hz, 3H, CH_3_). ^13^C NMR (101 MHz, DMSO) δ 174.32, 170.53, 138.18, 131.07, 124.87, 120.21, 31.49, 29.23, 7.56. MS (EI, 70 ev) *m*/*z* (%) = 287 (M^+^, 100.0, base peak), 187 (76.60), 172 (86.71), 101 (37.29), 91 (37.11).

### 2.17. Synthesis of 2-((4-formamidophenyl)selanyl)-N-phenylacetamide (**17**)

A mixture of 2-((4-aminophenyl)selanyl)-*N*-phenylacetamide (**4**) (305.24 mg, 1.00 mmol) was added in 5 mL of THF, followed by the dropwise addition of freshly prepared acetic formic anhydride (1.5 mmol). The mixture was stirred at room-temperature and monitored with TLC. After the reaction was complete, it was extracted with CH_2_Cl_2,_ and the organic phase was washed with distilled water and dried over MgSO4. Then, the oily product was washed again with petroleum ether (2 × 10 mL) and ether 10 mL. The product formation was followed by TLC: dichloromethane: methanol (9.50:0.50); Rf = 0.4; brown crystals; yield = 233.3 mg (70%); MP = 143–144 °C. IR (KBr): λmax.cm^−1^ = 3273, 3137, 3111, 2993, 2893, 1659, 1599, 1528, 1491, 1441, 1392, 1315. ^1^H NMR (400 MHz, DMSO-d_6_) δ 10.31 (s, 1H, NH), 10.16 (s, 1H, NH), 8.33 (s, 1H, CHO), 7.57 (s, 5H, Ar-H), 7.35 (d, *J* = 6.7 Hz, 2H, Ar-H), 7.08 (d, *J* = 6.6 Hz, 2H, Ar-H), 3.75 (s, 2H, SeCH_2_). ^13^C NMR (101 MHz, DMSO-d_6_) δ 168.53, 160.16, 139.46, 138.12, 134.42, 133.81, 129.24, 123.95, 123.85, 120.30, 119.56, 31.31; MS (EI, 70 ev) *m*/*z* (%) = 334 (M^+^, 47.6), 332 (24.39), 333 (7.39), 335 (10.81), 93 (100.0, base peak).

### 2.18. Synthesis of 2-((4-acetamidophenyl)selanyl)-N-phenylacetamide (**18**)

A mixture of 2-((4-aminophenyl)selanyl)-*N*-phenylacetamide (**4**) (305.24 mg, 1.00 mmol) and 0.2 mL acetic anhydride was heated in an oil bath at 60–65 °C for 1 h. The reaction mixture was allowed to cool to room temperature and was then poured into cold water. The resulting precipitate was filtered and recrystallized from ethanol. The product formation was followed by TLC: (CH_2_Cl_2_:MeOH, 10:1), Rf = 0.4, brown powder; yield = 200.5 mg (58%); MP = 161–162 °C; IR (KBr): λmax.cm^−1^ = 3234, 3177, 3109, 1670, 1650, 1596, 1491, 1316. ^1^H NMR (400 MHz, DMSO-d_6_) δ 10.11 (s, 1H, NH), 10.02 (s, 1H, NH), 7.54 (s, 4H, Ar-H), 7.53–7.52 (m, 2H, Ar-H), 7.31 (s, 2H, Ar-H), 7.06 (s, 1H, Ar-H), 3.68 (s, 2H, SeCH_2_), 2.17 (s, 3H, CH_3_). ^13^C NMR (101 MHz, DMSO-d_6_) δ 168.86, 168.55, 139.48, 139.29, 133.78, 129.24, 123.84, 123.07, 120.10, 119.55, 31.36, 24.39. MS (EI, 70 ev) *m*/*z* (%) = 348 (M^+^, 30.88), 305 (1.89), 226 (4.64), 189 (8.18), 172 (33.35), 113 (41.93), 106 (59.28), 93 (100.0, base peak), 77 (22.06).

### 2.19. Synthesis of 2-chloro-N-(4-((phenylamino)ethyl)selanyl)phenyl)acetamide (**19**)

To a solution of the corresponding 2-((4-aminophenyl)selanyl)-N-phenylacetamide (**4**) (1.0 mmol) in dry acetone (15 mL) containing K_2_CO_3_ (1 g) and chloroacetyl chloride (1.0 mmol) was added dropwise with stirring at 0–5 °C. Stirring was continued for 4 h, and the reaction mixture was poured into ice cold water. The resulting precipitate was collected, dried, and recrystallized from ethanol to afford the compound **19**. The product formation was followed by TLC: pet. ether/ethyl acetate (4:4)]; Rf = 0.4; white crystals; yield = 317.8 mg (87.8%); MP = 184.8 °C. IR (KBr): λmax.cm^−1^ = 3295, 3182, 2936, 1669, 1602, 1534, 1492, 1328. ^1^H NMR (400 MHz, DMSO) δ 10.39 (s, 1H, NH), 10.13 (s, 1H, NH), 7.55 (d, *J* = 9.1 Hz, 6H, Ar-H), 7.30 (d, *J* = 7.9 Hz, 2H, Ar-H), 7.05 (d, *J* = 7.2 Hz, 1H, Ar-H), 4.27 (s, 2H, COCH_2_Cl), 3.72 (s, 2H, SeCH_2_). ^13^C NMR (101 MHz, DMSO) δ 168.50, 165.18, 139.46, 138.33, 133.69, 129.25, 124.34, 123.86, 120.49, 119.55, 44.04, 31.23. MS (EI, 70 ev) *m*/*z* (%) = 382 (M^+^, 11.61), 291 (1.06), 261 (8.22), 248 (4.99), 196 (6.50), 172 (12.61), 113 (13.63), 106 (30.76), 93 (100.0, base peak), 77 (23.14).

### 2.20. Synthesis of 2-((4-(((dimethylamino)methylene)amino)phenyl)selanyl)-N-phenylacetamide (**20**)

A solution of the 2-((4-aminophenyl)selanyl)-*N*-phenylacetamide (**4**) (305.24 mg, 1.0 mmol) and DMFDMA dimethyl acetal (0.36 g, 3 mmol) in methanol (3.0 mL) was heated to 70 °C, with stirring under nitrogen for 3 h or until completion as indicated by TLC. The reaction was cooled to room temperature, and the product was isolated via evaporation to dryness. The product formation was followed by TLC: (CH_2_Cl_2_:MeOH, 10:1), Rf = 0.4; white powder; yield = 167.7 mg (47%); MP = 118–120 °C; IR (KBr): λmax.cm^−1^ = 3284, 3131, 2936, 1656, 1626, 1571, 1537, 1492, 1440, 1175. ^1^H NMR (400 MHz, DMSO-d_6_) δ 10.09 (s, 1H, NH), 7.73 (s, 1H, N=CH), 7.56 (d, 2H, Ar-H), 7.43 (d, 2H, Ar-H), 7.32–7.31 (m, 2H, Ar-H), 7.06–7.05 (m, 1H, Ar-H), 6.86 (d, 2H, Ar-H), 3.66 (s, 2H, SeCH_2_), 3.05 (s, 3H, CH_3_), 2.91 (s, 3H, CH_3_). ^13^C NMR (101 MHz, DMSO-d_6_) δ 168.68, 154.26, 152.27, 139.55, 134.54, 129.23, 123.79, 122.11, 121.15, 119.54, 34.46, 31.69; (EI, 70 ev) *m*/*z* (%) = 361 (M^+^, 100.0, base peak), 227 (58.69), 185 (14.67), 147 (17.83), 120 (11.15), 106 (31.44), 93 (20.11).

### 2.21. Synthesis of 2-((4-((2-hydroxybenzylidene)amino)phenyl)selanyl)-N-phenylacetamide (**21**)

A solution of (benzo)hydroxybenzaldehyde (1 mmol) and 2-((4-aminophenyl)selanyl)-*N*-phenylacetamide (**4**) (305.24 mg, 1.00 mmol) in methanol (10 mL) was refluxed for 4 h. The crude product precipitated from the reaction mixture was recrystallized from ethanol. The product formation was followed by TLC: CH_2_Cl_2_:MeOH (9.50:0.50), Rf = 0.71; yellow powder; yield = 361.6 mg (88.19 %); MP = 150.3 °C; IR (KBr): λmax.cm^−1^ = 3350, 3195, 1643, 1485, 1328; ^1^H NMR (400 MHz, DMSO-d_6_) δ 13.02 (s, 1H, OH), 10.20 (s, 1H, NH), 8.98 (s, 1H, N=CH), 7.67 (s, 1H, Ar-H), 7.56 (s, 2H, Ar-H), 7.46 (s, 2H, Ar-H), 7.38 (s, 2H, Ar-H), 7.32 (s, 2H, Ar-H), 7.08–7.05 (m, 1H, Ar-H), 7.00 (s, 2H, Ar-H), 6.97 (s, 1H, Ar-H), 3.8 (s, 2H, SeCH_2_). ^13^C NMR (101 MHz, DMSO) δ 168.43, 163.86, 160.74, 147.46, 139.46, 133.86, 133.20, 133.03, 129.27, 129.04, 123.90, 122.68, 119.79, 119.68, 119.55, 117.09, 30.88; MS (EI, 70 ev) *m*/*z* (%) = 410 (M^+^, 44.97), 276 (20.26), 224 (10.44), 196 (23.30), 167 (9.45), 120 (14.44), 106 (32.63), 93 (100.0, base peak), 77 (69.91), 65 (62.71).

## 3. Results and Discussion

### 3.1. Synthesis

Organoselenium compound development has recently gained considerable interest due to the potential chemo-preventive anticancer and antioxidant activities [6,32]. Furthermore, their synthesis is not always straightforward, requires hazardous reagents (e.g., KSeCN), and is carried out under certain conditions (e.g., inert atmosphere) [5,10,33,34]. Within this context, our synthetic strategy relies on developing OSe scaffolds tethered with diverse functionalities (e.g., alkyl, carboxylic, amidic, azo, and formamide). In turn, the latter functionalities are present in natural and pharmacologically active compounds, thus giving access to candidates structurally suitable for biological screening.

**Scheme 1:**
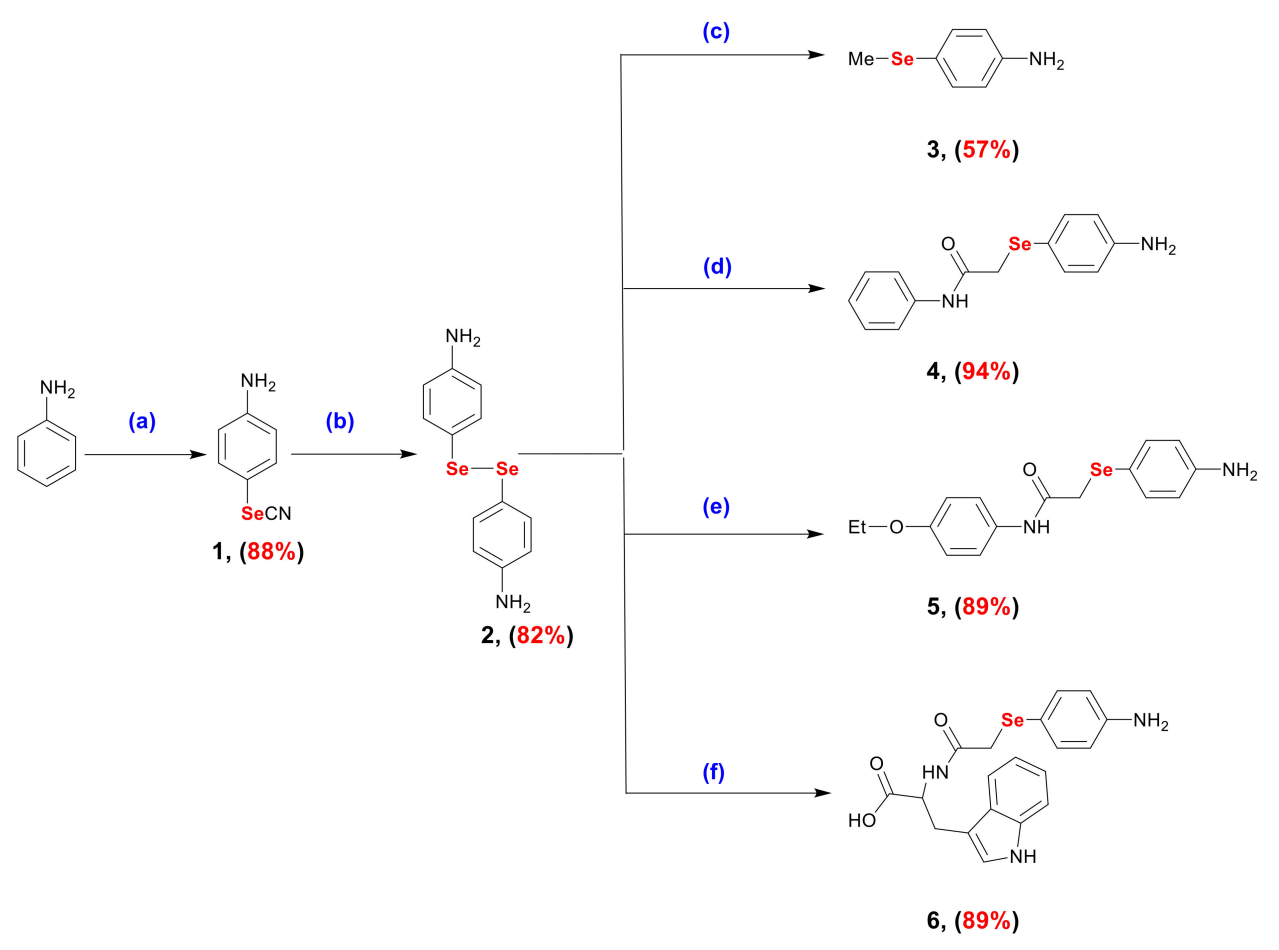
Synthesis of OSe agents **3**, **4**, **5**, **6**. Reagents and conditions: (**a**) Aniline (72 mmol), malononitrile (4.8 mmol), selenium dioxide (9.6 mmol), DMSO (4 mL); (**b**) Amine (**1**) (2 mmol), NaOH (3 mmol), EtOH (15 mL); (**c**) Amine (**2**) (1 mmol), iodomethane (2.2 mmol), NaBH_4_ (3 mmol), NaOH (1 mmol), EtOH (15 mL).; (**d**) 2-chloro-*N*-phenylacetamide (2.2 mmol), NaBH_4_ (3 mmol), NaOH (1 mmol), EtOH (15 mL); (**e**) 2-chloro-*N*-(4-ethoxyphenyl)acetamide (2.2 mmol), NaBH_4_ (3 mmol), NaOH (1 mmol), EtOH (15 mL); (**f**) (2-chloroacetyl)tryptophan (2 mmol), NaBH_4_ (3 mmol), NaOH (1 mmol), EtOH (15 mL).

The synthons **3**, **4**, **5**, and **6** were prepared in good yields (up to 94%) from the corresponding 4,4’-diselanediyldianiline (**2**) [33] by the one-pot reduction of the diselenide bond, using NaBH_4_ in the presence of an equimolar amount of NaOH, followed by a reaction with appropriate alkyl chlorides (Scheme 1). Interestingly, this reaction proceeded smoothly at room temperature, under air, and the use of NaOH accelerated the reaction rate up to 30 min.

**Scheme 2:**
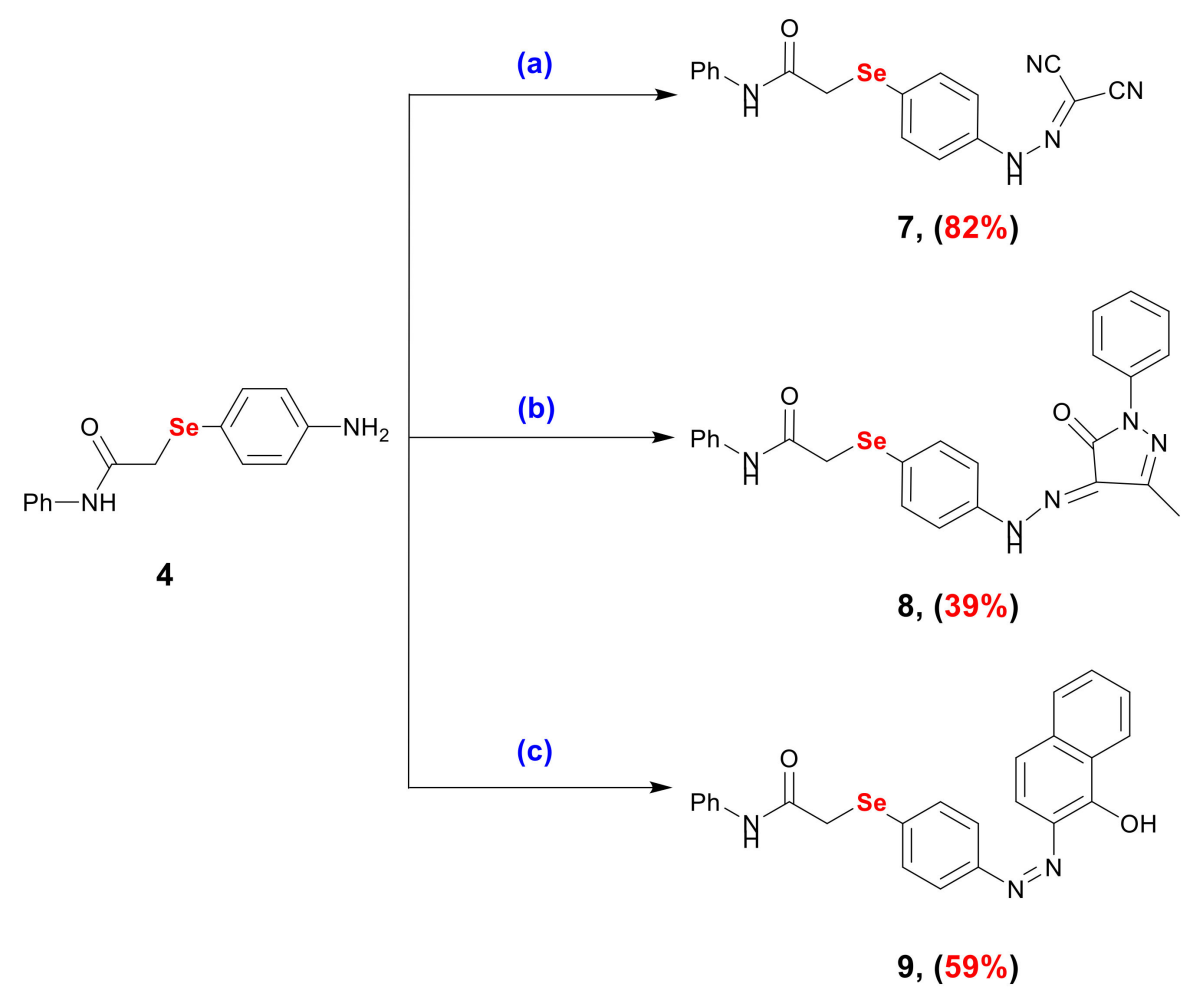
Synthesis of selenide-based azo compounds. Reagents and conditions: Amine **4** (1 mmol), H_2_SO_4_ or AcOH (8 mL), NaNO_2_ (10 mmol) (8 mL), at 0 °C. (**a**) Malononitrile (1.2 mmol); (**b**) 5-methyl-2-phenyl-1,2-dihydro-3*H*-pyrazol-3 (1.2 mmol); (**c**) β-naphthol (1.2 mmol).

Furthermore, the azo functionality has attracted much interest due to its simple accessibility and relative stability [10,34,35,36]. Moreover, azo compounds have diverse applications and are extensively used in cosmetics, painting, and staining several cellular compartments [37,38,39]. Recently, we have shown that OSe-based azo compounds have potential anticancer and antimicrobial properties [28]. In this context, diazotization of 2-((4-aminophenyl)selanyl)-*N*-phenylacetamide (**4**) and the subsequent reaction with malononitrile, 5-methyl-2-phenyl-2,4-dihydro-3*H*-pyrazol-3-one, and β-naphthol afforded the corresponding azo dyes **7**, **8**, and **9**, respectively (Scheme 2). 

**Scheme 3:**
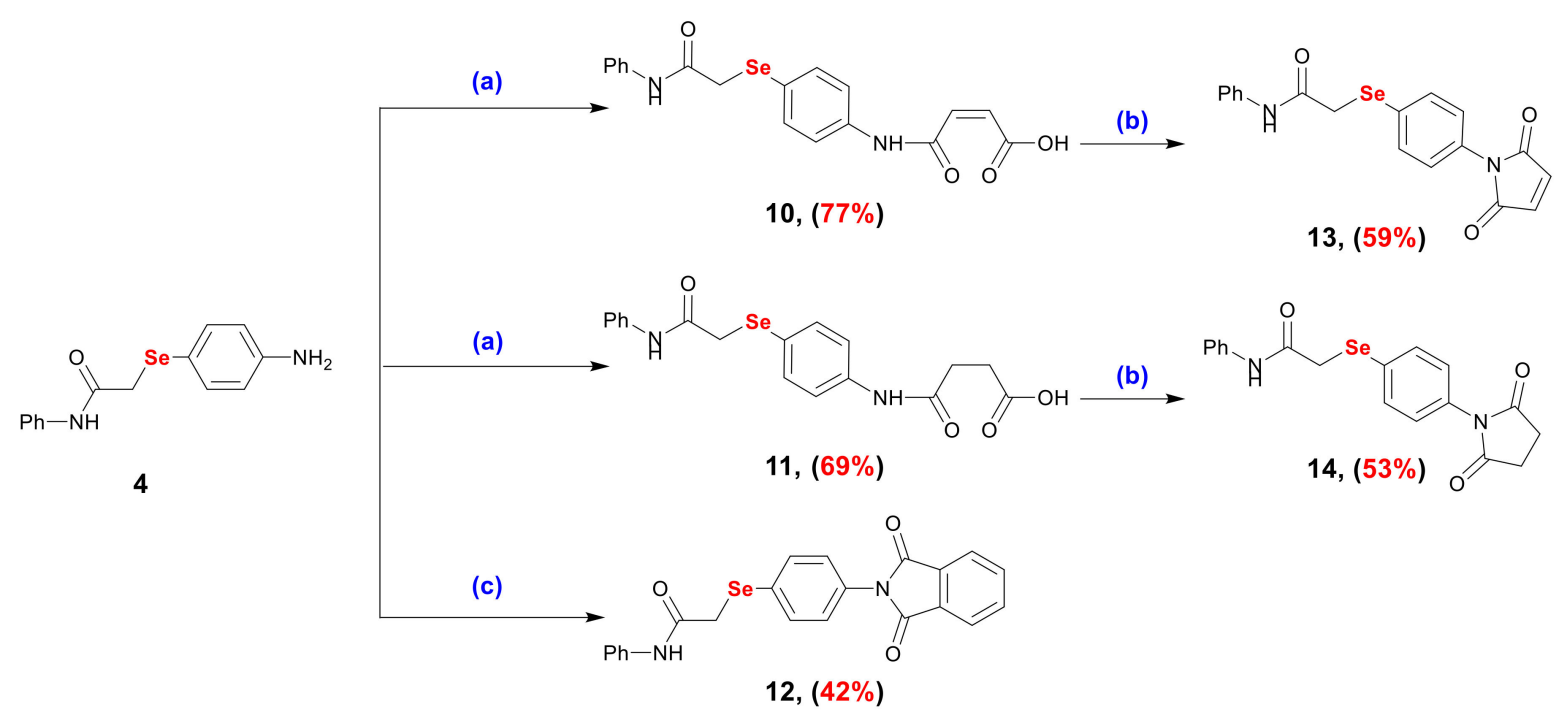
Reactions of selenoamines **4** with anhydrides and subsequent dehydration. Reagents and conditions: (**a**) amine **4** (1 mmol), toluene (5 mL), maleic/succinic anhydride (1 mmol), r.t, 3 h; (**b**) amide-acid (1 eq), NaOAc (100 mg), Ac_2_O (3 mL), 2 h, 50–60 °C; (**c**) phthalic anhydride (1mmol); AcOH (5 mL), reflux for 10 h.

Additionally, cyclic imides are interesting scaffolds and key intermediates in organic and polymer synthesis as pharmacological relevant agents [40,41,42]. Therefore, our strategy was oriented to the reaction of **4** with different anhydrides. Within this context, the reaction of **4** with maleic and succinic anhydrides afforded the respective *N*-maleanilic **10** and *N*-succinanilic **11** acids in 77 and 69% yields, respectively. Furthermore, heating of *N*-maleanilic **10** and N-succinanilic **11** acids with acetic anhydride led to ring-closure and formation of the corresponding N-maleimides **13** and *N*-succinimides **14** in 59 and 53% yields, respectively (Scheme 3).

**Scheme 4:**
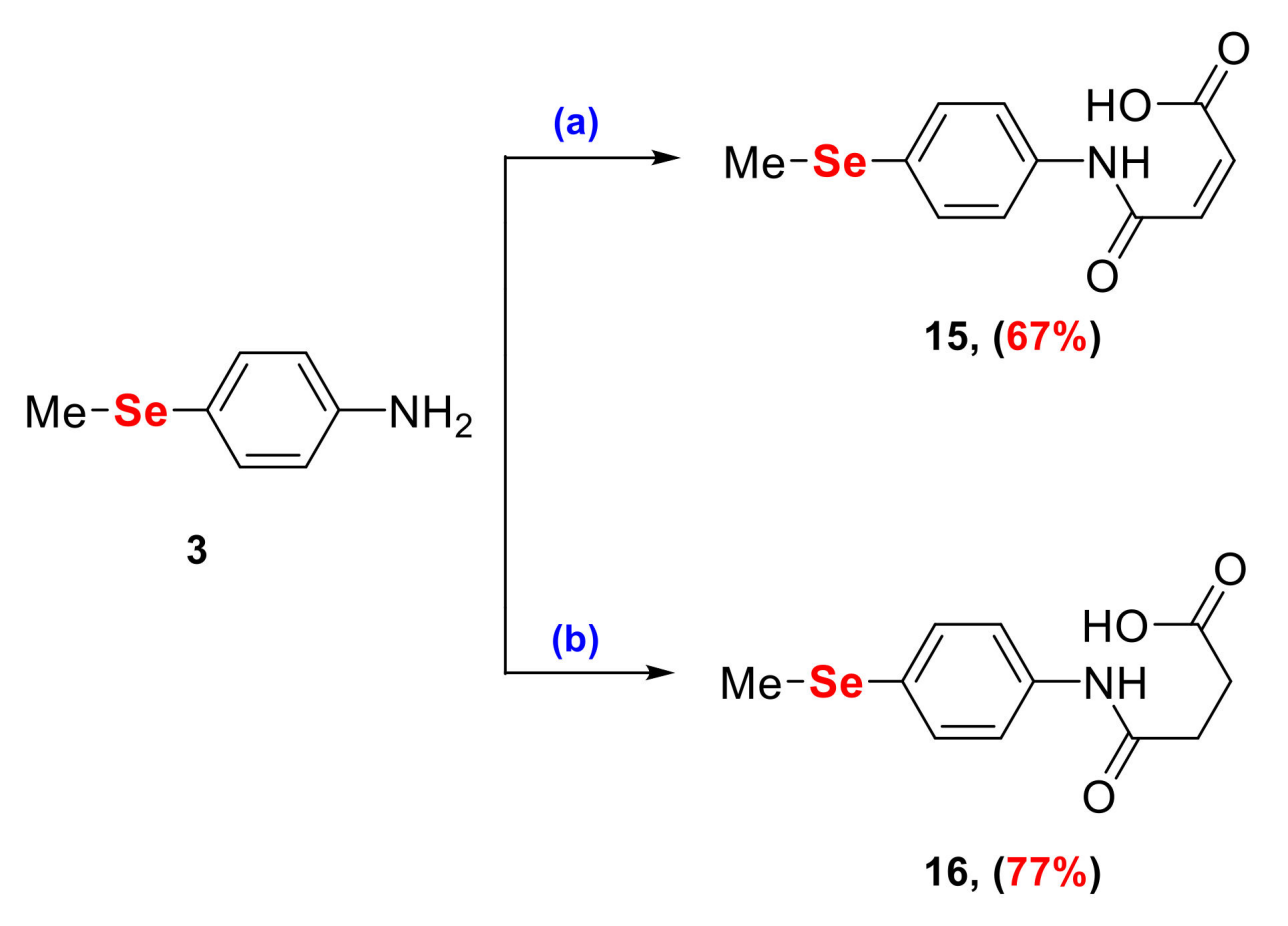
Reactions of 4-(methylselanyl)aniline 4 with anhydride. Reagents and conditions: (1 mmol), toluene (5 mL), anhydride (**a**) maleic anhydride and (**b**) succinic anhydride (1 mmol) (1 mmol), r.t, 3 h.

On the other hand, the reaction of **4** with phthalic anhydride produced the corresponding phthaloyl derivative **12** in 42% yield (Scheme 3). Similarly, the reaction of **3** with maleic and succinic anhydrides furnished the corresponding *N*-maleanilic **15** and *N*-succinanilic **16** acids in 67 and 77% yields, respectively (Scheme 4).

**Scheme 5:**
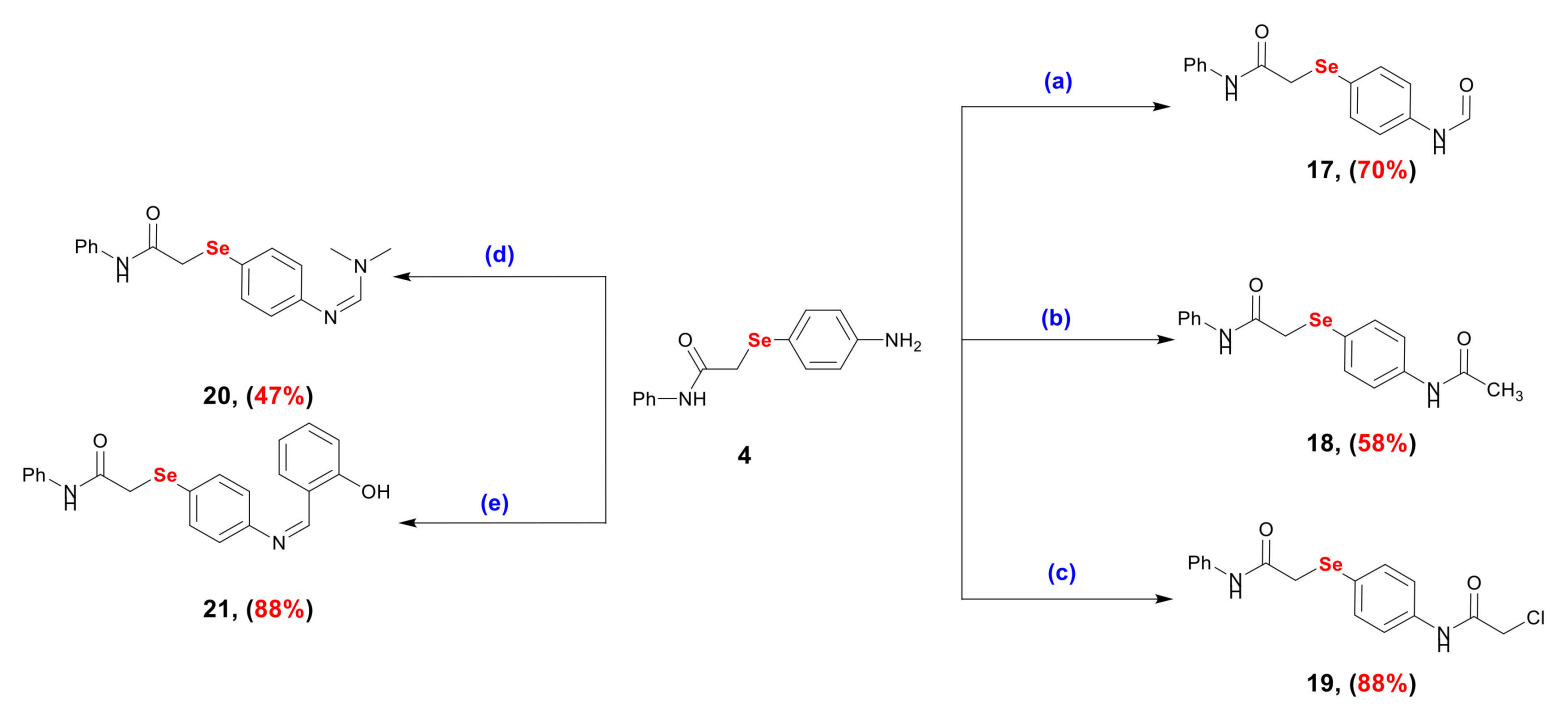
General pathways to prepare selenoamide derivatives. Reagents and conditions: Selenoamines **4** (1 mmol) reaction with (**a**) acetic formic anhydride (1.2 mmol), THF (5 mL), rt; (**b**) Ac_2_O (3 mL), 2 h, 60–65 °C; (**c**) chloroacetyl chloride (1.0 mmol), K_2_CO_3_ (1 g), dry acetone (15 mL); (**d**) **4** (1 mmol), DMFDMA (3 mmol), MeOH (5 mL), reflux, 3 h; (**e**) **4** (1 mmol), salicylaldehyde (1 mmol), MeOH (10 mL), reflux, 1 h.

Additionally, the reaction of **4** with formic acetic anhydride, acetic anhydride, and chloroacetyl chloride afforded the corresponding OSe-based formamide **17**, acetanilide **18**, and 2-chloro-*N*-phenylacetamide **19** in 70, 58, and 88 % yields, respectively (Scheme 5).

Additionally, the reaction of **4** with *N*, *N*-dimethylformamide dimethyl acetal afforded 2-((4-(((dimethylamino)methylene)amino)phenyl)selanyl)-*N*-phenylacetamide **20** in the 47% yield, whereas the reaction of **4** with salicylaldehyde afforded the corresponding Schiff base **21** in the 88% yield (Scheme 5).

### 3.2. Biology

#### 3.2.1. Evaluation of the Cytotoxicity of OSe Compounds

Recently, OSe compounds gained much attention as possible drug candidates due to their various pharmacological properties [6,10,33,34]. Our group has reported several OSe candidates with potential anticancer, antimicrobial, and antioxidant activities [10,23,43,44,45]. Therefore, the anticancer attributes of the newly prepared OSe compounds were estimated using the MTT assay against MCF-7 and HepG2 and compared with their respective cytotoxicity in WI-38 primary cells. The anthracycline drug doxorubicin was used as the standard. The minimal inhibition concentration required to kill 50% of the cells (IC_50_) was calculated from dose-response curves presented in Table 1. Moreover, the therapeutic index (TI) was also calculated (according to Equation (1)), which is considered a measure of the selectivity and safety of the synthesized compounds.

(1) $$TI=\frac{\mathrm{IC}50\;\mathrm{for}\;\mathrm{the}\;\mathrm{normal}\;\left(\mathrm{WI}38\right)\;\mathrm{cells}}{\mathrm{IC}50\;\mathrm{for}\;\mathrm{the}\;\mathrm{cancer}\;\mathrm{cells}}$$

In general, the OSe cytotoxicity was more obvious against the MCF-7 cells than the HepG2 cells. In this context, OSe compound **9** was more cytotoxic than doxorubicin with IC_50_ = 3.27 ± 0.2 against 4.17 ± 0.2 µM, in the case of MCF-7, respectively. Furthermore, (2-((4-aminophenyl)selanyl)acetyl)tryptophan (**6**) and 4-((4-(methylselanyl)phenyl)amino)-4-oxobut-2-enoic acid (**15**) manifested potential cytotoxicity in MCF-7 with IC_50_ = 5.69 ± 0.4 and 7.03 ± 0.6 µM, respectively. Furthermore, OSe compounds **4**, **14**, **11**, and **16** exhibited moderate cytotoxicity with IC_50_ = 11.29 ± 0.8, 17.08 ± 1.4, 21.25 ± 1.8, and 29.53 ± 2.2 µM, respectively. Coincidentally, 2-((4-((1-hydroxynaphthalen-2-yl)diazenyl)phenyl)selanyl)-*N*-phenylacetamide (**9**), (2-((4-aminophenyl)selanyl)acetyl)tryptophan (**6**), and 4-((4-(methylselanyl)phenyl)amino)-4-oxobut-2-enoic acid (**15**) also showed pronounced cytotoxicity against HepG2 cells with IC_50_ = 7.48 ± 0.6, 8.86 ± 0.7, and 9.94 ± 0.8 µM, respectively, whereas **14**, **4**, and **11** displayed moderate cytotoxicity with IC_50_ = 12.57 ± 1.0, 18.33 ± 1.3, and 26.70 ± 1.9 µM, respectively.

Chemo drugs should be cytotoxic to cancer cells with minimal toxicity to primary cells [28,29,46,47,48]. Therefore, higher TI values are highly desirable to guarantee the safety of a specific drug. In this context, promising TI values were observed in the case of MCF-7 cells compared to HepG2 cells. OSe compounds **9**, **6**, **4**, and **5** displayed higher selective toxicity to the MCF-7 cells with TI values of 12, 5, 4.6, and 4, respectively, whereas, in HepG2 cells, compounds **9**, **14**, **6**, and **4** showed good selectivity with TI values of 5.3, 5, 3.2, and 2.9 (Table 1). These promising results are worth more research employing a more comprehensive arsenal of healthiness and tumors for in vivo studies.

#### 3.2.2. Estimation of the Antimicrobial Properties of the OSe Compounds

Compounds were evaluated for their antimicrobial activity against the Gram-negative bacteria *Escherichia coli* (*E. coli*) and the Gram-positive bacteria *Staphylococcus aureus* (*S. aureus*), as well as the *Candida albicans* (*C. albicans*) fungal strain, using the agar diffusion assay. The inhibition zone diameters (IZD) (in mm) are listed in Table 2. Ampicillin and clotrimazole were used as standards. Indeed, the antimicrobial properties of the OSe compounds were also obtained from the activity index percentage (A%) according to Equation (2).

(2) $$A\%=\frac{Zone\;of\;inhibition\;by\;test\;compound\;\left(diametre\right)}{Zone\;of\;inhibition\;by\;standard\;\left(diametre\right)}\times 100\;.$$

Compounds **9**, **6**, **15**, and **4** (good antimicrobial activity compounds) with A% of 87, 74, 65, and 61% in the case of *E. coli*, 91, 86, 71, and 71% in the case of *S. aureus*, and 88, 83, 67, and 54% in the case of *C. albicans* (Table 2). Fortunately, these compounds also exhibited potential cytotoxicity, and these, in turn, need to be further investigated and explored against broader series of fungal and bacterial strains. 

Furthermore, the most active OSe compounds **6** and **9** were further tested in the minimum inhibitory concentration (MIC) assay to estimate their corresponding lowest microbial inhibition growth concentrations (Table 3). Within this regard, **9** exhibited superior activity to **6** and similar antimicrobial potential to ampicillin and clotrimazole drugs with MICs of 0.5, 1, and 2 μM against *E. coli*, *S. aureus*, and *C. albicans* strains, respectively.

#### 3.2.3. Evaluation of the Antioxidant Attributes of the OSe Compounds

**Figure 2:**
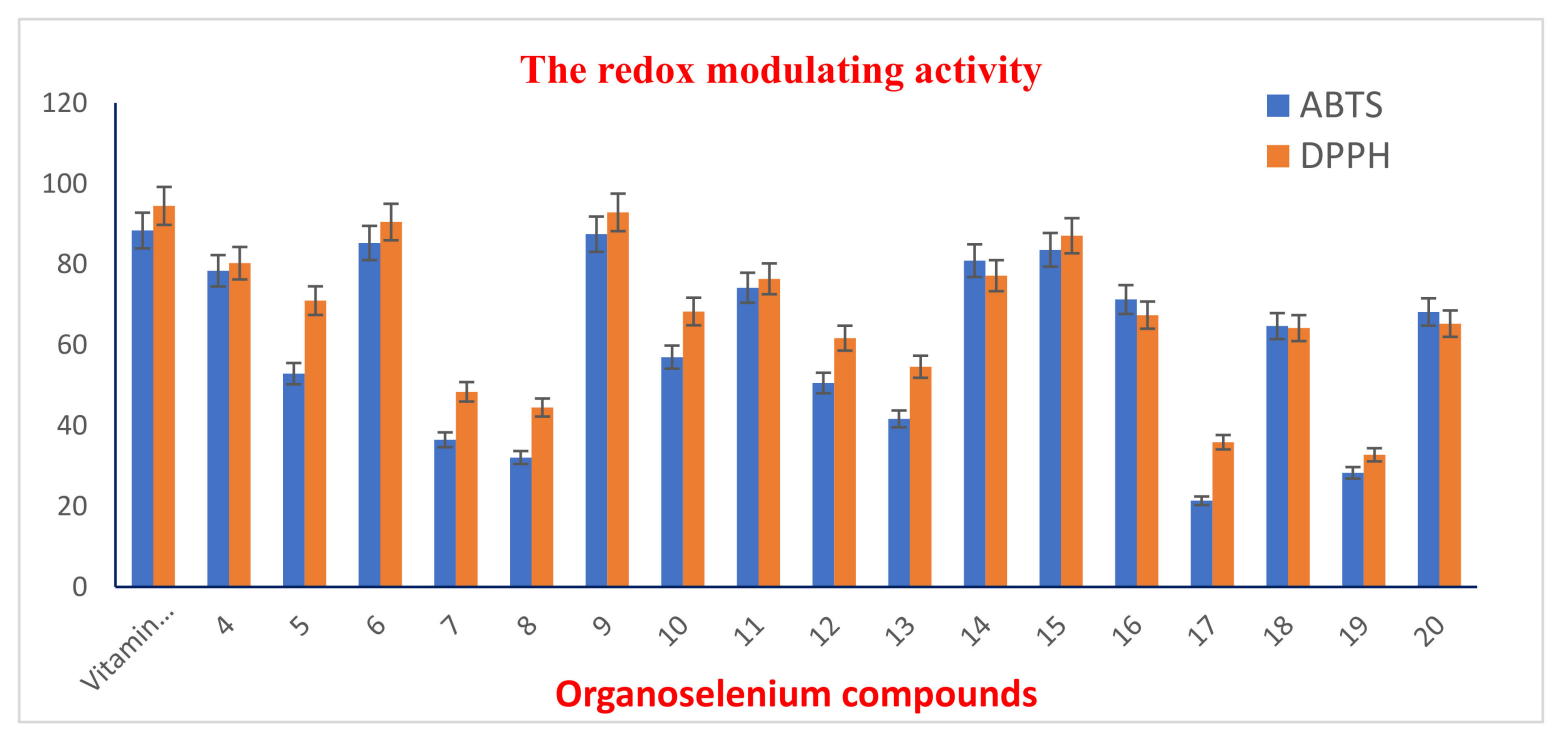
Evaluation of the antioxidant properties of the OSe compounds employing the ABTS and DPPH assays. All measurements are mean ± SD. In the DPPH assay: absorbance was read after 0.5 h of mixing 400 mL of DPPH with 200 mL of OSe compounds (1 mM, MeOH) at 517 nm. In the ABTS assay: 50 mL of each OSe compound (1 mM, phosphate-buffered methanol) was mixed with the ABTS solution (60 mM), and absorbance was monitored at 734 nm.

Recently, OSe compounds were thoroughly used as redox modulators to control many complicated diseases (e.g., cancer and neurodegenerative diseases) [33,49]. The DPPH and ABTS bioassays were extensively used as rapid tools for estimating the antioxidant potency of OSe compounds [50,51]. Ascorbic acid was used as a standard control, and the antioxidant potency was measured by the ability of the compounds to decolorize the characteristic colors of the ABTS**^.^** and DPPH**^.^** radicals by following the colorimetric decline in absorbance at 734 and 517 nm, respectively (Figure 2). 

As presented in Figure 2, compounds **9**, **6**, **15**, **4**, **14**, **11**, **5**, and **16** exhibited 93, 91, 87, 80, 77, 76, 71, 67% and 88, 85, 84, 78, 81, 74, 53, 71% radical scavenging activities compared to 95% and 88% by ascorbic acid in the DPPH and ABTS assays, respectively. 

Furthermore, the most active compounds **6** and **9** were further selected, and the minimal concentration required to decrease the absorbance by 50% in the DPPH and ABTS assays were deduced from their respective dose-response curves and presented in Table 4. Interestingly, compounds **9** and **6** manifested potential antioxidant activity similar to vitamin C. To conclude, our results were in good agreement with the anticancer and antimicrobial data confirming the possible pharmacological activity of such compounds.

These findings are in accordance with our previous results [11,24,25,50], where OSe compounds used preexisting ROS and expedited their reactions with redox-sensitive cellular compartments, leading to cellular malfunction and subsequently cell death [13,15,23,35,52,53]. Mechanistically, OSe compounds undergo oxidation at the Se center via ROS (e.g., H_2_O_2_), followed by the recovery of the Se redox center by reduction with thiols [14,50,54,55,56,57,58]. The latter in this case might be part of the cysteine that presents in redox-sensitive proteins, enzymes, endoplasmic reticulum (ER), or actin. These data introduce new antineoplastic agents to overcome the emergence of resistance and improve the clinical outcomes for patients.

## 4. Conclusions

Twenty-one novel organoselenium compounds were synthesized in good- yields (up to 91%) and were confirmed by IR, MS, and ^1^H- and ^13^C-NMR spectroscopy. In addition, their antitumor, antimicrobial, and antioxidant properties were also evaluated using different bioassays. The cytotoxicity was generally more pronounced against the MCF-7 cells than HepG2 cells. OSe **9** was the most promising; its cytotoxicity was higher than doxorubicin (IC_50_ = 3.27 ± 0.2 vs. 4.17 ± 0.2 µM). Furthermore, OSe compounds **6** and **15** showed potential cytotoxicity in MCF-7 with IC_50_ = 5.69 ± 0.4 and 7.03 ± 0.6 µM, respectively. Moreover, OSe compounds **9**, **6**, and **15** also showed pronounced cytotoxicity against HepG2 cells with IC_50_ = 7.48 ± 0.6, 8.86 ± 0.7, and 9.94 ± 0.8 µM, respectively, and displayed interesting selective toxicity to the MCF-7 cells with TI values up to 12. The same hold was true in the case of the antimicrobial assay, whereas compounds **9**, **6**, **15**, and **4** showed good A% of 87, 74, 65, and 61% in the case of *E. coli*, 91, 86, 71, and 71% in the case of *S. aureus*, and 88, 83, 67, and 54% in the case of *C. albicans.*

Additionally, compounds **9**, **6**, **15**, **4**, **14**, **11**, **5**, and **16** exhibited antioxidant activities in the DPPH and ABTS assays. Compounds **6** and **9** manifested similar antioxidant properties to vitamin C. To this end, our promising results are worth more research using a broader set of healthy and cancer cells in vivo.

## Figures and Tables

**Table 1:** The cytotoxicity of OSe compounds.

Compounds	MCF7 ^a^		HepG2 ^a^		WI38 ^a^
	IC50 (µM) ^a^	TI ^c^	IC_50_ (µM) ^a^	TI ^c^	IC_50_ (µM) ^a^
**Doxorubicin**	4.17 ± 0.2	1.6	4.50 ± 0.2	1.5	6.72 ± 0.5
**4**	11.29 ± 0.8	4.6	18.33 ± 1.3	2.9	52.32 ± 3.0
**5**	25.47 ± 2.0	4	-^b^	-	-^b^
**6**	5.69 ± 0.4	5	8.86 ± 0.7	3.2	28.44 ± 2.2
**7**	-^b^	-	-^b^	-	-^b^
**8**	-^b^	-	-^b^	-	-^b^
**9**	3.27 ± 0.2	12	7.48 ± 0.6	5.3	39.28 ± 2.5
**10**	34.76 ± 2.5	1.3	-^b^	-	46.03 ± 2.9
**11**	21.25 ± 1.8	3.2	26.70 ± 1.9	2.5	67.29 ± 3.9
**12**	-^b^	-	-^b^	-	-^b^
**13**	-^b^	-	-^b^	-	-^b^
**14**	17.08 ± 1.4	3.6	12.57 ± 1.0	5	61.97 ± 3.6
**15**	7.03 ± 0.6	2.8	9.94 ± 0.8	2	19.76 ± 1.5
**16**	29.53 ± 2.2	0.7	32.61 ± 2.3	0.7	22.49 ± 1.9
**17**	-^b^	-	-^b^	-	-^b^
**18**	43.45 ± 2.6	2	47.62 ± 2.8	1.8	85.07 ± 4.7
**19**	-^b^	-	-^b^	-	-^b^
**20**	36.81 ± 2.4	2.7	40.28 ± 2.5	2.5	-^b^

^a^ The anticancer activities of the OSe compounds were assayed by the MTT method against HepG2, MCF-7, and WI-38 cells after twenty-four h of incubation with different concentrations of the OSe compounds. ^b^ Growth inhibition was not detected in the used concentration ranges (IC_50_ > 100 µM); ^c^ TI is the ratio of the IC_50_ of primary WI-38 cells to the cancer (HepG2 or MCF-7) IC_50_ for each compound.

**Table 2:** The antimicrobial attributes of OSe compounds.

Compound	*E. coli*		*S. aureus*		*C. albicans*	
	IZD (mm) ^a^	A%	IZD (mm) ^a^	A%	IZD (mm) ^a^	A%
**4**	14	61	15	71	13	54
**5**	10	44	12	57	8	33
**6**	17	74	18	86	20	83
**7**	3	13	6	29	NA	-
**8**	NA	-	4	19	NA	-
**9**	20	87	19	91	21	88
**10**	9	39	11	52	8	33
**11**	12	52	13	62	13	54
**12**	6	26	8	38	7	29
**13**	5	22	6	29	NA	-
**14**	13	57	14	67	15	63
**15**	15	65	15	71	16	67
**16**	10			43	12	50
**17**	NA	-	NA	-	NA	-
**18**	6	26	8	30	11	46
**19**	NA	-	NA	-	NA	-
**20**	8	35	9	43	11	46
**Ampicillin**	23	100	21	100	-	-
**Clotrimazole**	-	-	-	-	24	100

^a^ Inhibition zone diameters (mm) determined using six-mm disks were soaked with 20 μM of the OSe compounds. Values below 6 mm (25 %) are of limited value, as they refer to inactive or non-diffusing compounds.

**Table 3:** The MIC of OSe compounds **6** and **9**.

Compounds	MIC (μM)		
	*E. coli*	*S. aureus*	*C. albicans*
**6**	1	2	8
**9**	0.5	1	2
**Ampicillin**	0.5	1	-
**Clotrimazole**	-	-	2

**Table 4:** The antioxidant properties of the OSe compounds using the DPPH and ABTS bioassays.

Compounds	IC_50_ (µM)	
	DPPH	ABTS
**Vitamin C**	19.18 ± 0.13	28.16 ± 0.19
**6**	24.03 ± 0.23	31.92 ± 0.29
**9**	20.64 ± 0.14	28.87 ± 0.20

## Data Availability

Data is contained within the article and Supplementary Materials.

## Supplementary Materials

The following supporting information can be downloaded at: https://www.mdpi.com/article/10.3390/antiox11071231/s1, experimental details of the biological assays and Copies of ^1^H & ^13^CNMR spectra IR and MS.
