# Supplementary material for: Novel Organoselenium Redox Modulators with Potential Anticancer, Antimicrobial, and Antioxidant Activities
Source: Antioxidants (Basel). 2022 Jun 23;11(7):1231. doi: 10.3390/antiox11071231 (PMC9312238; doi:10.3390/antiox11071231)
Supplement: Supplementary file 1 [file antioxidants-11-01231-s001.zip › antioxidants-1742994-supplementary.pdf]

Supplementary Materials

Novel organoselenium redox modulators with potential anti-cancer, antimicrobial and antioxidant properties

Marwa Sak[1], Yasair S. Al-Faiyz[1], Hany Elsawy[1,2], Saad Shaaban[1,3]

[1]*Chemistry Department, College of Science, King Faisal University, P.O. Box 400, Al-Ahsa 31982, Saudi Arabia.*
[2]*Chemistry Department, Faculty of Science, Tanta University, Tanta, Egypt.*
[3]*Chemistry Department, Faculty of Science, Mansoura University, Mansoura, Egypt.*
*   *Correspondence: sibrahim@kfu.edu.sa; dr_saad_chem@mans.edu.sa*

SUPPORTING INFORMATION	PAGE
The biological assays	S2
Copies of ^{1}H & ^{13}CNMR spectra IR and MS	S6

1.1. The biological assays

1.1.1. The anticancer activity

The breast adenocarcinoma (MCF-7), Hepatocellular carcinoma (HEPG-2), and normal fibroblast (WI-38) cells were obtained from ATCC via Holding company for biological products and vaccines (VACSERA), Cairo, Egypt. The reagents RPMI-1640 medium, MTT, DMSO, and fetal bovine serum was obtained from Sigma Aldrich. Dulbecco's modified Eagle's medium was used as the culture medium for the cells, and the cytotoxicity was evaluated using the reported MTT assay. Doxorubicin was used as the positive reference, and the IC_{50} values were obtained from the corresponding dose-response curve.

MTT assay[23,24,58]

This colorimetric assay is based on converting the yellow tetrazolium bromide (MTT) to a purple formazan derivative by mitochondrial succinate dehydrogenase in viable cells. Cell lines were cultured in RPMI-1640 medium with 10% fetal bovine serum. Antibiotics added were 100 units/ml penicillin and 100µg/ml streptomycin at 37 C in a 5% Co_2 incubator. The cell lines were seeded in a 96-well plate at a density of $1.0x10^4$ cells/well. at 37 C for 48 h under 5% Co_2. After incubation, the cells were treated with different concentrations of compounds and incubated for 24 h. After 24 h of drug treatment, 20 µl of MTT solution at 5mg/ml was added and incubated for 4 h. Finally, dimethyl sulfoxide (DMSO) in the volume of 100 µl is added to each well to dissolve the purple formazan formed. The colorimetric assay is measured and recorded at an absorbance of 570 nm using a plate reader (EXL 800, USA). The percentage of the relative cell viability was calculated as (A570 of treated samples/A570 of the untreated sample) X 100.

1.1.1. The antimicrobial activity

The antimicrobial activities of the OSe compounds were evaluated against *C. albicans* yeast as well as *E. coli* gram-negative and *S. aureus* gram-positive bacteria employing the agar well diffusion assay [31]. Briefly, a concentration of 1 mM was prepared for each compound by dissolving in DMSO. Paper discs of standard size (5cm) were sterilized in an autoclave and soaked in 20 µL of the test compounds, and placed in the Petri dishes, which in turn contain a nutrient media (agar 20 g, peptone 5 g, and beef extract 3 g) seeded with the dedicated strain. Incubation lasted for 24 h at 36 ^{0}C. Experiments were replicated three times and the antifungal clotrimazole and antibiotic ampicillin were used as standards. The % activity index for the complex was determined and depicted in table 2.

Furthermore, the MICs (in µM) were determined by the microdilution method according to the reported protocol.

1.1.1. The antioxidant activity

The DPPH bioassay

The hydrogen atom or electron donation ability of the corresponding compounds was measured by estimating the bleaching of the purple color of a methanolic solution of DPPH [29]. This spectrophotometric assay uses stable DPPH· reagent. The sample was prepared by adding 200 mL of the OSe compounds (1 mM in methanol) to 400 mL DPPH in methanol. After 30 min of incubation in the dark, the absorbance was read against a blank at 517 nm. Ascorbic acid (vitamin C) and ebselen were used as standard antioxidants (positive control). A blank sample was run without DPPH. A negative control sample was run using methanol instead of the sample. The radical scavenging activity was calculated using the following equation:

$$\text{Inhibition\%} = (A_{blank} - A_{sample})/(A_{blank})*100. \quad (1)$$

The ABTS bioassay

The antioxidant activity of the investigated compounds was assessed using 2,2′-azino-bis(3-ethylbenzothiazoline-6-sulphonic acid (ABTS) method [28]. The radical cation derived from ABTS was prepared by the reaction of 60 mM ABTS solution with 0.3 M manganese dioxide solution in 0.1M phosphate buffer, pH 7. Then, the mixture was shaken, centrifuged, filtered, and the absorbance ($A_{control}$) of the resulting green-blue solution (ABTS radical solution) was measured at wavelength 734 nm. Then, 50 mL of 1 mg/ml test compound in phosphate-buffered methanol was added. The absorbance (A_{test}) was measured. The reduction in color intensity was expressed as % inhibition. The % inhibition for each compound is calculated from the following equation

$$Inhibition\% = (A_{control}-A_{sample})/(A_{control})*100$$

Ascorbic acid (vitamin C) was used as a standard antioxidant (positive control). A blank sample was run without ABTS and using MeOH/phosphate buffer (1:1) instead of the sample. A negative control sample was run with MeOH/phosphate buffer (1:1) instead of a tested compound.

2.-((4-. Aminophenyl)selanyl)-N-phenylacetamide (4).

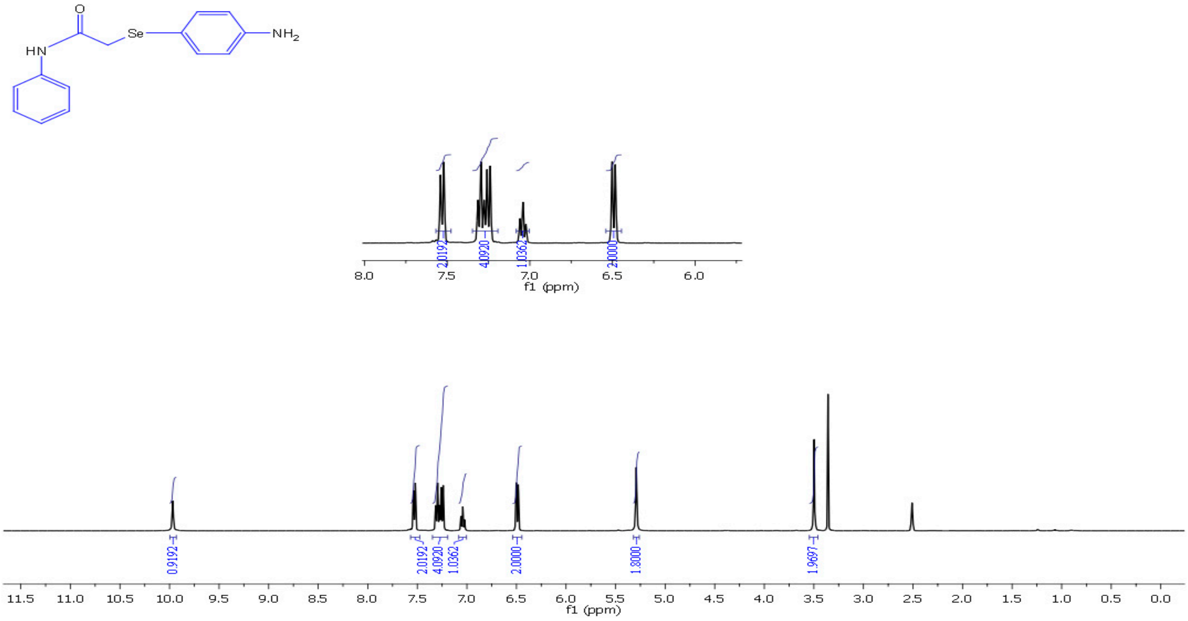

^{1}HNMR chart of compound 4.

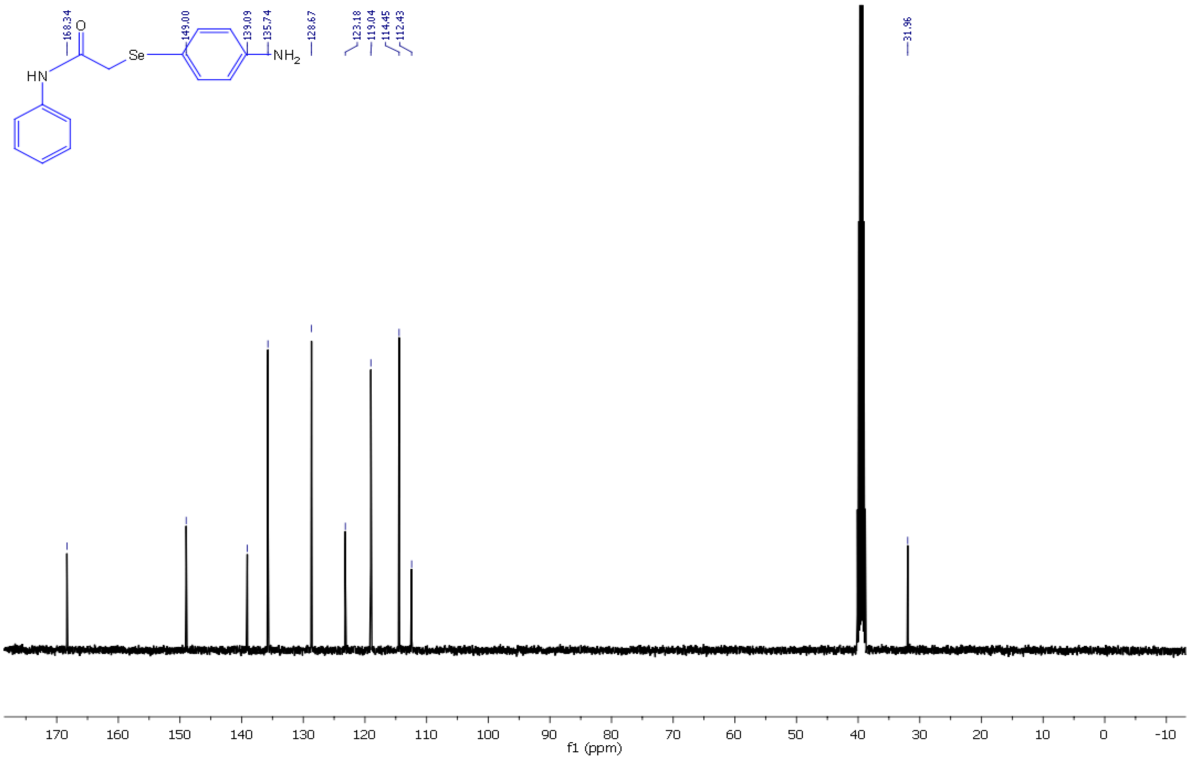

^{13}CNMR chart of compound 4.

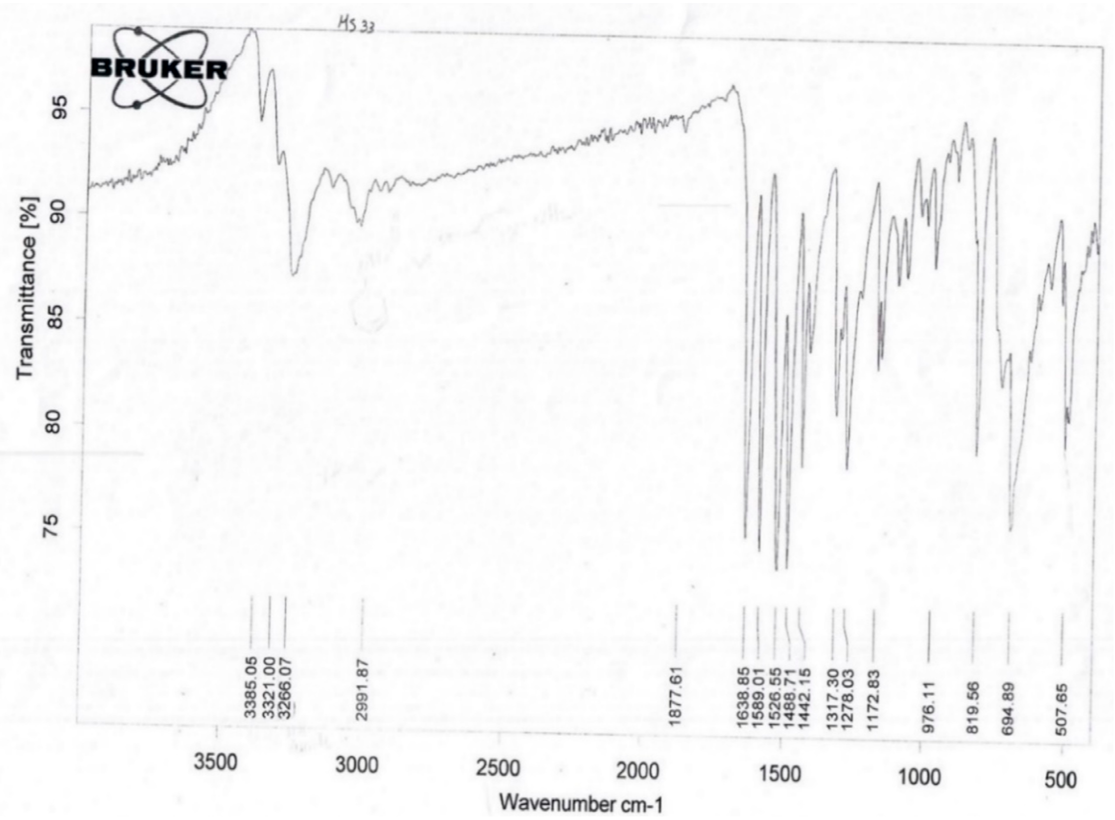

IR chart of compound 4.

Cairo University
Micro Analytical Center

DI Analysis
Shimadzu Qp-2010 Plus

Sample Information

Analyzed by	: Dr. Mai Younis
Analyzed	: 03/01/2007 08:34:43 م
Sample Name	: M1
Sample ID	:
Customer Name	: Dr.Mohamed Soliman - Science - Cairo
Data File	: C:\GCMSsolution\Data\Project1\M1.QGD
Org Data File	: C:\GCMSsolution\Data\Project1\M1.QGD
Method File	: C:\GCMSsolution\Data\Project1\High Temperature Op
Org Method File	: C:\GCMSsolution\Data\Project1\High Temperature Op
Report File	:
Tuning File	: C:\GCMSsolution\System\Tune1_default.qgt
$EndIf$Modified by	: Dr. Mai Younis
Modified	: 03/01/2007 08:39:19 م

Method

——— Analytical Line 1 ———

IonSourceTemp	:250.00 °C
[MS Table]	
--Group 1 - Event 1--	
Start Time	:0.00min
End Time	:10.00min
ACQ Mode	:Scan
Event Time	:0.50sec
Scan Speed	:1000
Start m/z	:50.00
End m/z	:510.00
Electron Voltage	: 70 eV
Ionization Mode	: EI

C:\GCMSsolution\Data\Project1\M1.QGD

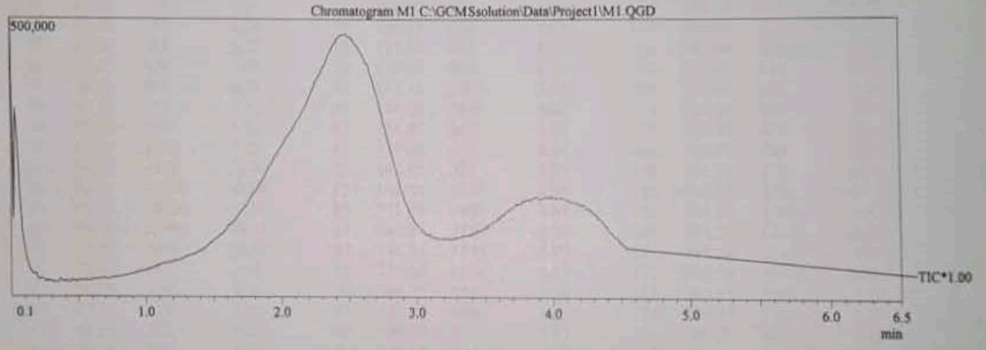

Spectrum

Line#:1 R.Time:2.4(Scan#:294)
MassPeaks:138
RawMode:Single 2.4(294) BasePeak:106(40671)
BG Mode:None Group 1 - Event 1

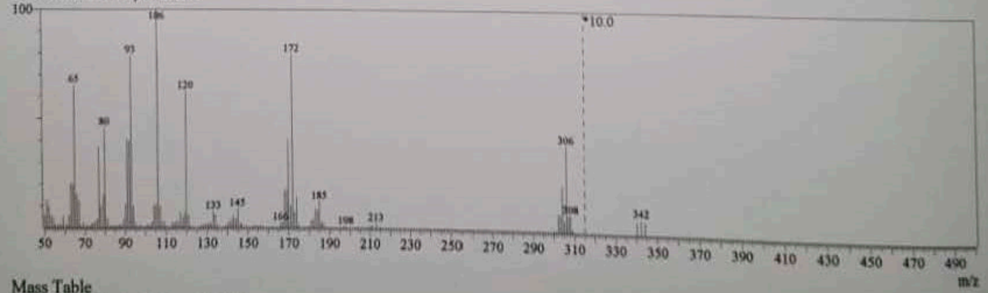

Mass Table
Line#:1 R.Time:2.4(Scan#:294)
MassPeaks:138
RawMode:Single 2.4(294) BasePeak:106(40671)
BG Mode:None Group 1 - Event 1

#	m/z	Abs. In	Rel. Int.	#	m/z	Abs. In	Rel. Int.	#	m/z	Abs. In	Rel. Int.
1	50.05	2145	5.27	4	53.00	2289	5.63	7	56.10	308	0.76
2	51.00	5174	12.72	5	54.05	1813	4.46	8	57.05	773	1.90
3	52.00	4034	9.92	6	55.00	738	1.81	9	58.05	441	1.08

1 / 2

Mass chart of compound 4.

03-Jan-07 20:41:42

#	m/z	Abs. In	Rel. Int.
10	59.05	2117	5.21
11	60.00	263	0.65
12	61.05	741	1.82
13	62.05	2224	5.47
14	63.00	8390	20.63
15	64.05	8024	19.73
16	65.00	26594	65.39
17	66.05	6216	15.28
18	67.05	5126	12.60
19	68.05	469	1.15
20	69.05	1002	2.46
21	70.00	274	0.67
22	71.10	418	1.03
23	72.10	225	0.55
24	73.05	585	1.44
25	74.00	913	2.24
26	75.05	1042	2.56
27	76.05	1684	4.14
28	77.05	15082	37.08
29	78.05	4166	10.24
30	79.15	5967	14.67
31	80.05	18694	45.96
32	81.05	1651	4.06
33	82.10	273	0.67
34	83.20	407	1.00
35	84.20	284	0.70
36	85.15	429	1.05
37	86.10	287	0.71
38	87.05	577	1.42
39	88.15	429	1.05
40	89.10	1559	3.83
41	90.15	4179	10.28
42	91.10	16543	40.68
43	92.10	16103	39.59
44	93.10	32092	78.91
45	94.05	4076	10.02
46	95.00	887	2.18
47	96.00	238	0.59
48	97.15	413	1.02
49	98.10	212	0.52
50	99.10	239	0.59
51	100.10	268	0.66
52	101.10	532	1.31

#	m/z	Abs. In	Rel. Int.
53	102.15	435	1.07
54	103.15	1019	2.51
55	104.10	4239	10.42
56	105.15	4288	10.54
57	106.10	40671	100.00
58	107.10	3977	9.78
59	108.10	1118	2.75
60	109.15	1252	3.08
61	110.10	286	0.70
62	111.10	281	0.69
63	113.05	893	2.20
64	114.05	809	1.99
65	115.00	1428	3.51
66	116.05	1010	2.48
67	117.00	2828	6.95
68	118.10	1710	4.20
69	119.15	2833	6.97
70	120.10	25470	62.62
71	121.10	2334	5.74
72	122.10	340	0.84
73	123.10	204	0.50
74	127.05	478	1.18
75	128.10	481	1.18
76	129.10	521	1.28
77	130.15	663	1.63
78	131.10	681	1.67
79	132.10	866	2.13
80	133.15	3020	7.43
81	134.10	2447	6.02
82	135.20	607	1.49
83	139.00	340	0.84
84	140.05	531	1.31
85	141.00	1198	2.95
86	142.05	1403	3.45
87	143.00	2166	5.33
88	144.05	1546	3.80
89	145.00	3506	8.62
90	146.15	844	2.08
91	147.05	650	1.60
92	149.10	329	0.81
93	152.10	201	0.49
94	153.10	327	0.80
95	154.10	228	0.56

#	m/z	Abs. In	Rel. Int.
96	155.10	233	0.57
97	156.10	322	0.79
98	157.10	284	0.70
99	166.00	964	2.37
100	167.05	939	2.31
101	168.00	7049	17.33
102	169.05	7179	17.65
103	170.00	16562	40.72
104	171.05	4059	9.98
105	172.00	32386	79.63
106	172.95	3009	7.40
107	174.00	5649	13.89
108	174.95	488	1.20
109	180.05	389	0.96
110	181.00	1271	3.13
111	182.05	1585	3.90
112	183.05	3600	8.85
113	184.05	3205	7.88
114	185.00	4900	12.05
115	185.95	1031	2.53
116	187.00	950	2.34
117	188.00	239	0.59
118	197.00	222	0.55
119	198.00	239	0.59
120	204.00	202	0.50
121	209.00	233	0.57
122	210.00	212	0.52
123	210.95	521	1.28
124	213.00	900	2.21
125	215.00	220	0.54
126	300.00	324	0.80
127	301.20	233	0.57
128	302.15	3325	8.18
129	303.20	3262	8.02
130	304.20	8436	20.74
131	305.20	2142	5.27
132	306.20	15977	39.28
133	307.15	3102	7.63
134	308.15	3162	7.77
135	309.15	589	1.45
136	340.20	241	0.59
137	342.20	286	0.70
138	344.20	231	0.57

Mass chart of compound 4.

2. -((4-Aminophenyl)selanyl)-N-(4-ethoxyphenyl)acetamide (5)

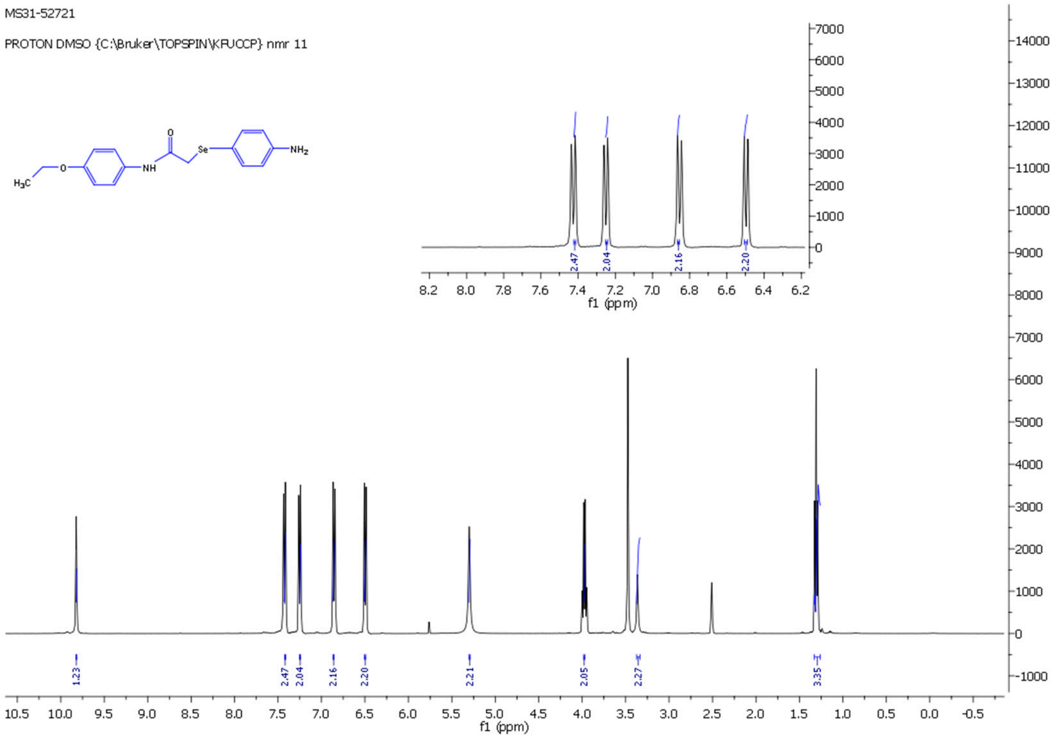

^{1}HNMR chart of compound 5.

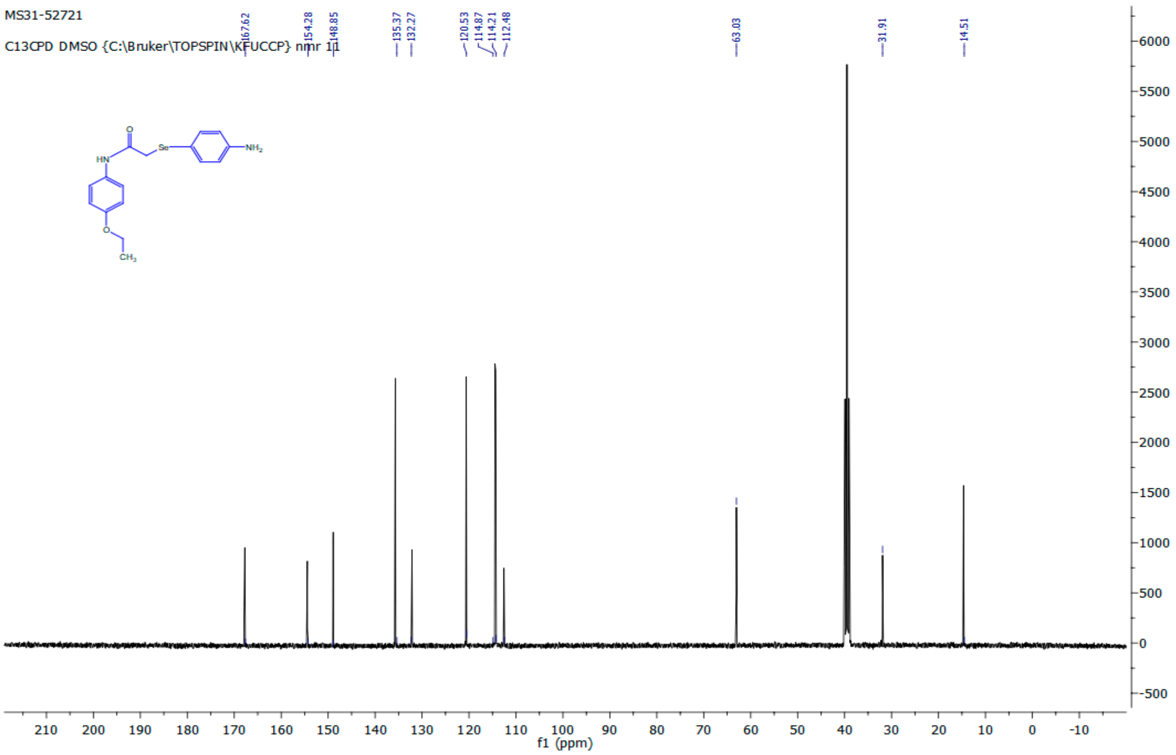

^{13}CNMR chart of compound 5.

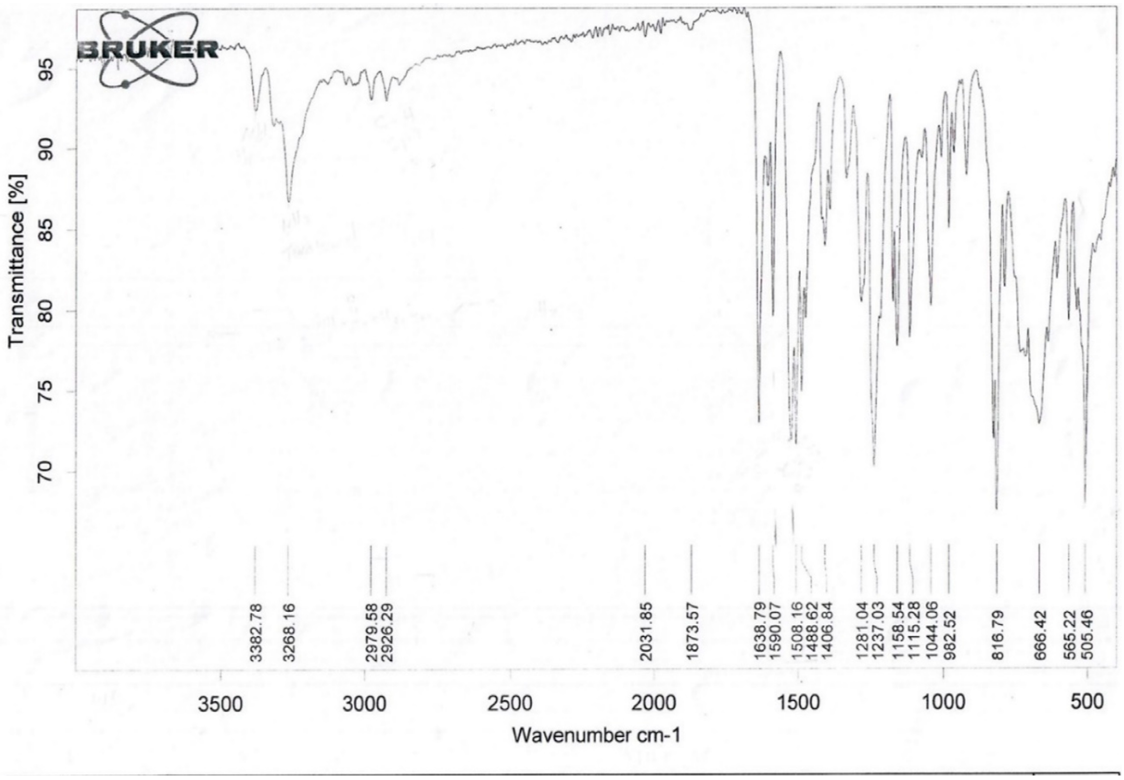

IR chart of compound 5.

Cairo University
Micro Analytical Center

DI Analysis
Shimadzu Qp-2010 Plus

Sample Information

Analyzed by : Dr. Mai Younis
Analyzed : 03/01/2007 05:54:12 م
Sample Name : M3
Sample ID :
Customer Name : Dr.Mohamed Soliman - Science - Cairo
Data File : C:\GCMSsolution\Data\Project1\M3.QGD
Org Data File : C:\GCMSsolution\Data\Project1\M3.QGD
Method File : C:\GCMSsolution\Data\Project1\High Temperature Op
Org Method File : C:\GCMSsolution\Data\Project1\High Temperature Op
Report File :
Tuning File : C:\GCMSsolution\System\Tune1_default.qgt
$EndIf$Modified by : Dr. Mai Younis
Modified : 03/01/2007 05:59:32 م

Method

—— Analytical Line 1 ——
IonSourceTemp :250.00 °C
[MS Table]
--Group 1 - Event 1--
Start Time :0.00min
End Time :10.00min
ACQ Mode :Scan
Event Time :0.50sec
Scan Speed :1000
Start m/z :50.00
End m/z :510.00

Electron Voltage : 70 eV
Ionization Mode : EI

C:\GCMSsolution\Data\Project1\M3.QGD

Chromatogram M3 C:\GCMSsolution\Data\Project1\M3.QGD

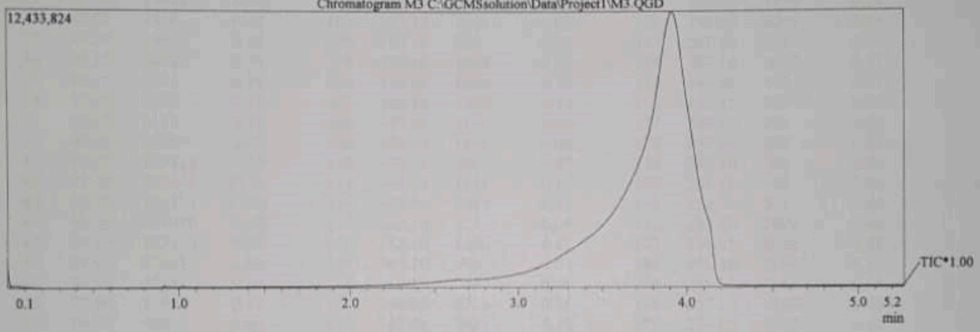

Spectrum

Line#:1 R.Time:3.9(Scan#:471)
MassPeaks:260
RawMode:Single 3.9(471) BasePeak:108(1339614)
BG Mode:None Group 1 - Event 1

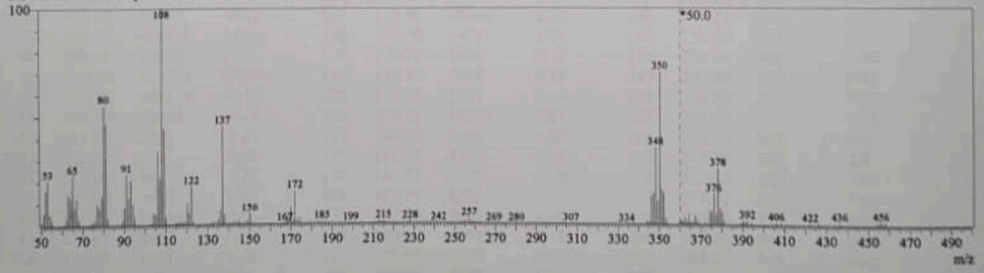

Mass Table
Line#:1 R.Time:3.9(Scan#:471)
MassPeaks:260
RawMode:Single 3.9(471) BasePeak:108(1339614)
BG Mode:None Group 1 - Event 1

#	m/z	Abs. In	Rel. Int.	#	m/z	Abs. In	Rel. Int.	#	m/z	Abs. In	Rel. Int.
1	50.10	26673	1.99	4	53.05	281399	21.01	7	56.05	4321	0.32
2	51.10	74106	5.53	5	54.05	66255	4.95	8	57.05	1924	0.14
3	52.05	214575	16.02	6	55.05	32828	2.45	9	58.05	472	0.04

Mass chart of compound 5.

03-Jan-07 18:01:14

#	m/z	Abs. In	Rel. Int.
10	59.05	1056	0.08
11	60.05	1301	0.10
12	61.05	8261	0.62
13	62.05	39287	2.93
14	63.05	191198	14.27
15	64.05	163198	12.18
16	65.05	311173	23.23
17	66.05	110402	8.24
18	67.05	161694	12.07
19	68.00	36036	2.69
20	69.00	4150	0.31
21	70.05	1415	0.11
22	71.05	1009	0.08
23	72.15	585	0.04
24	73.15	2433	0.18
25	74.05	12751	0.95
26	75.05	16324	1.22
27	76.15	30489	2.28
28	77.05	128811	9.62
29	78.05	106486	7.95
30	79.15	174147	13.00
31	80.10	742854	55.45
32	81.05	628154	46.89
33	82.05	39822	2.97
34	83.00	3810	0.28
35	84.15	1405	0.10
36	85.15	2078	0.16
37	86.05	2441	0.18
38	87.05	3040	0.23
39	88.15	6616	0.49
40	89.15	27966	2.09
41	90.15	105112	7.85
42	91.10	317612	23.71
43	92.10	170391	12.72
44	93.10	274410	20.48
45	94.10	133719	9.98
46	95.05	47668	3.56
47	96.05	5234	0.39
48	97.00	1495	0.11
49	98.00	449	0.03
50	99.05	578	0.04
51	100.15	483	0.04
52	101.15	1547	0.12
53	102.10	5350	0.40
54	103.15	9972	0.74
55	104.10	71010	5.30
56	105.15	64639	4.83
57	106.15	456210	34.06
58	107.15	282877	21.12
59	108.10	133961	100.00
60	109.10	596818	44.55
61	110.10	46042	3.44
62	111.10	3282	0.24
63	112.05	654	0.05
64	113.05	3821	0.29
65	114.05	5816	0.43
66	115.05	11454	0.86
67	116.05	8556	0.64
68	117.00	19577	1.46
69	118.05	14972	1.12
70	119.15	18549	1.38
71	120.15	134892	10.07
72	121.15	71858	5.36
73	122.15	237885	17.76
74	123.10	20335	1.52
75	124.15	1551	0.12
76	125.15	664	0.05
77	126.15	967	0.07
78	127.15	1837	0.14

#	m/z	Abs. In	Rel. Int.
79	128.10	2246	0.17
80	129.10	2785	0.21
81	130.10	3391	0.25
82	131.20	2551	0.19
83	132.20	8154	0.61
84	133.15	17681	1.32
85	134.25	8046	0.60
86	135.20	36110	2.70
87	136.25	88105	6.58
88	137.20	610646	45.58
89	138.15	59665	4.45
90	139.15	5395	0.40
91	140.05	2824	0.21
92	141.05	8164	0.61
93	142.05	9025	0.67
94	143.05	14286	1.07
95	144.05	9493	0.71
96	145.00	22374	1.67
97	146.10	3258	0.24
98	147.10	4793	0.36
99	148.15	8233	0.61
100	149.15	19074	1.42
101	150.15	66118	4.94
102	151.10	6878	0.51
103	152.15	1024	0.08
104	153.10	822	0.06
105	154.10	1298	0.10
106	155.05	1296	0.10
107	156.10	1550	0.12
108	157.05	1163	0.09
109	158.10	1025	0.08
110	159.15	996	0.07
111	160.15	1341	0.10
112	161.10	2889	0.22
113	162.10	1172	0.09
114	163.10	1460	0.11
115	164.10	390	0.03
116	165.15	739	0.06
117	166.05	4753	0.35
118	167.05	4821	0.36
119	168.05	43920	3.28
120	169.05	45472	3.39
121	170.00	106202	7.93
122	171.05	26727	2.00
123	172.00	208121	15.54
124	172.95	20970	1.57
125	174.00	38006	2.84
126	175.00	4471	0.33
127	176.10	2009	0.15
128	177.15	2376	0.18
129	178.15	5351	0.40
130	179.10	10830	0.81
131	180.05	2626	0.20
132	181.05	4219	0.31
133	182.05	6373	0.48
134	183.00	8706	0.65
135	184.00	8463	0.63
136	185.00	15417	1.15
137	186.00	6420	0.48
138	187.00	4713	0.35
139	188.00	2377	0.18
140	189.00	831	0.06
141	190.00	351	0.03
142	195.00	302	0.02
143	196.05	827	0.06
144	197.05	1240	0.09
145	198.05	2148	0.16
146	199.10	4007	0.30
147	200.05	2757	0.21

#	m/z	Abs. In	Rel. Int.
148	201.05	483	0.04
149	202.05	520	0.04
150	207.00	225	0.02
151	208.05	310	0.02
152	209.00	1481	0.11
153	210.05	1594	0.12
154	211.00	5889	0.44
155	212.05	5062	0.38
156	213.00	13601	1.02
157	214.05	5473	0.41
158	215.00	14673	1.10
159	216.00	5822	0.43
160	217.00	2870	0.21
161	217.90	1023	0.08
162	224.05	417	0.03
163	225.15	385	0.03
164	226.15	1530	0.11
165	227.15	8922	0.67
166	228.10	12318	0.92
167	229.05	2235	0.17
168	230.05	607	0.05
169	237.15	436	0.03
170	238.10	999	0.07
171	239.10	1838	0.14
172	240.05	2204	0.16
173	241.10	2817	0.21
174	242.10	3647	0.27
175	243.05	995	0.07
176	244.15	740	0.06
177	245.10	201	0.02
178	247.10	206	0.02
179	249.10	308	0.02
180	251.15	738	0.06
181	252.15	538	0.04
182	253.10	5692	0.42
183	254.15	6384	0.48
184	255.10	15505	1.16
185	256.15	4568	0.34
186	257.10	31590	2.36
187	258.05	5927	0.44
188	259.10	6092	0.45
189	260.05	1456	0.11
190	261.20	524	0.04
191	262.25	433	0.03
192	263.20	524	0.04
193	264.20	274	0.02
194	269.35	553	0.04
195	270.30	374	0.03
196	276.30	282	0.02
197	277.30	239	0.02
198	278.30	340	0.03
199	279.30	210	0.02
200	280.30	241	0.02
201	293.30	238	0.02
202	303.30	262	0.02
203	305.30	292	0.02
204	306.10	258	0.02
205	307.10	644	0.05
206	308.10	305	0.02
207	314.25	1160	0.09
208	315.25	337	0.03
209	320.30	207	0.02
210	334.30	238	0.02
211	336.30	209	0.02
212	341.20	268	0.02
213	342.25	414	0.03
214	343.25	1179	0.09
215	344.20	17007	1.27
216	345.25	13028	0.97

2 / 3

Mass chart of compound 5.

03-Jan-07 18:01:14

#	m/z	Abs. In	Rel. Int.
217	346.20	172303	12.86
218	347.25	193476	14.44
219	348.20	475158	35.47
220	349.25	144284	10.77
221	350.20	943716	70.45
222	351.15	216299	16.15
223	352.15	192110	14.34
224	353.15	38956	2.91
225	354.15	4979	0.37
226	355.15	551	0.04
227	360.30	489	0.04
228	361.25	388	0.03
229	362.20	975	0.07
230	363.25	474	0.04
231	364.20	1335	0.10

#	m/z	Abs. In	Rel. Int.
232	365.20	366	0.03
233	366.25	397	0.03
234	367.30	982	0.07
235	368.30	503	0.04
236	369.30	217	0.02
237	372.30	238	0.02
238	374.25	1653	0.12
239	375.25	1650	0.12
240	376.20	3822	0.29
241	377.25	1254	0.09
242	378.20	6972	0.52
243	379.15	2054	0.15
244	380.20	1518	0.11
245	381.20	476	0.04
246	389.20	238	0.02

#	m/z	Abs. In	Rel. Int.
247	390.20	406	0.03
248	391.20	348	0.03
249	392.25	518	0.04
250	394.20	300	0.02
251	406.20	298	0.02
252	408.20	207	0.02
253	422.20	250	0.02
254	426.20	238	0.02
255	436.25	359	0.03
256	454.20	247	0.02
257	455.10	330	0.02
258	456.10	449	0.03
259	457.10	217	0.02
260	458.25	360	0.03

Mass chart of compound 5.

(2-((4-Aminophenyl)selanyl)acetyl)tryptophan(6)

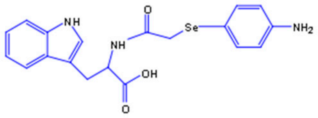

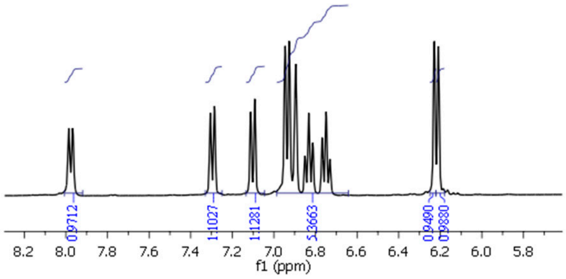

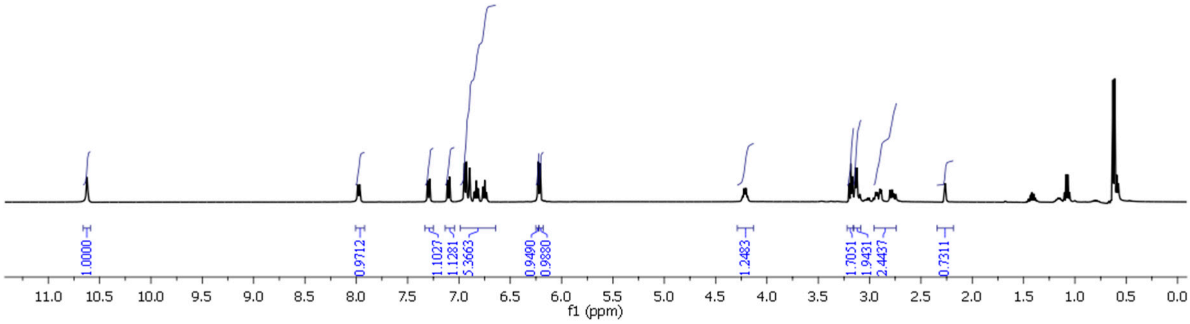

^{1}HNMR chart of compound 6.

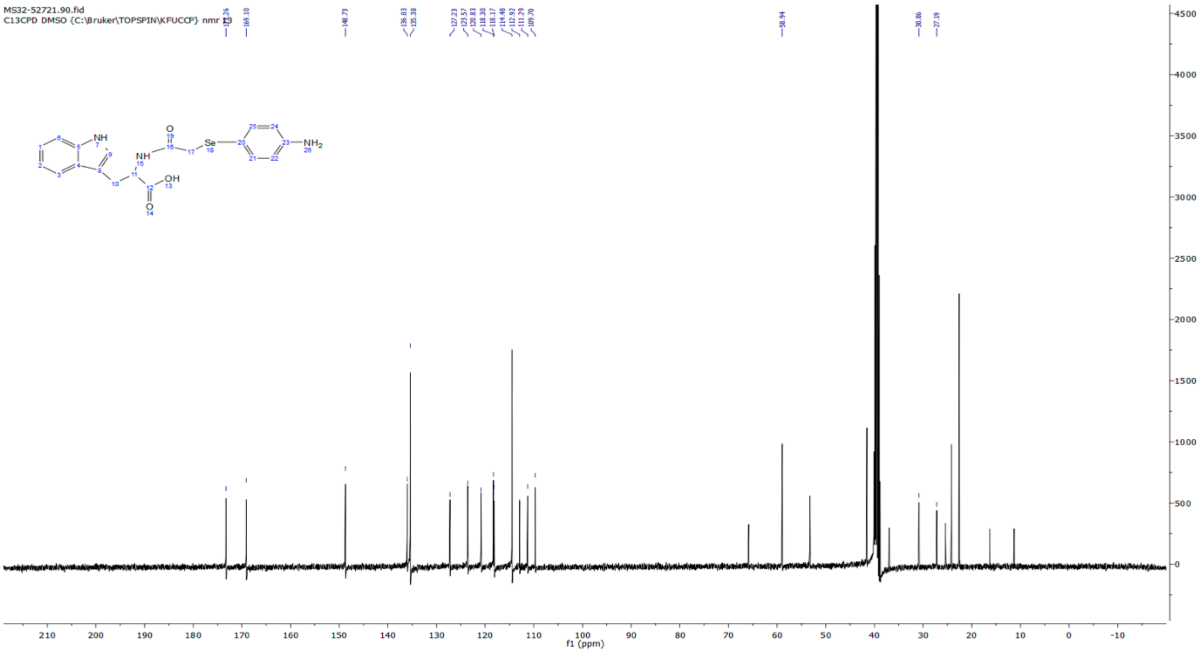

^{13}CNMR chart of compound 6.

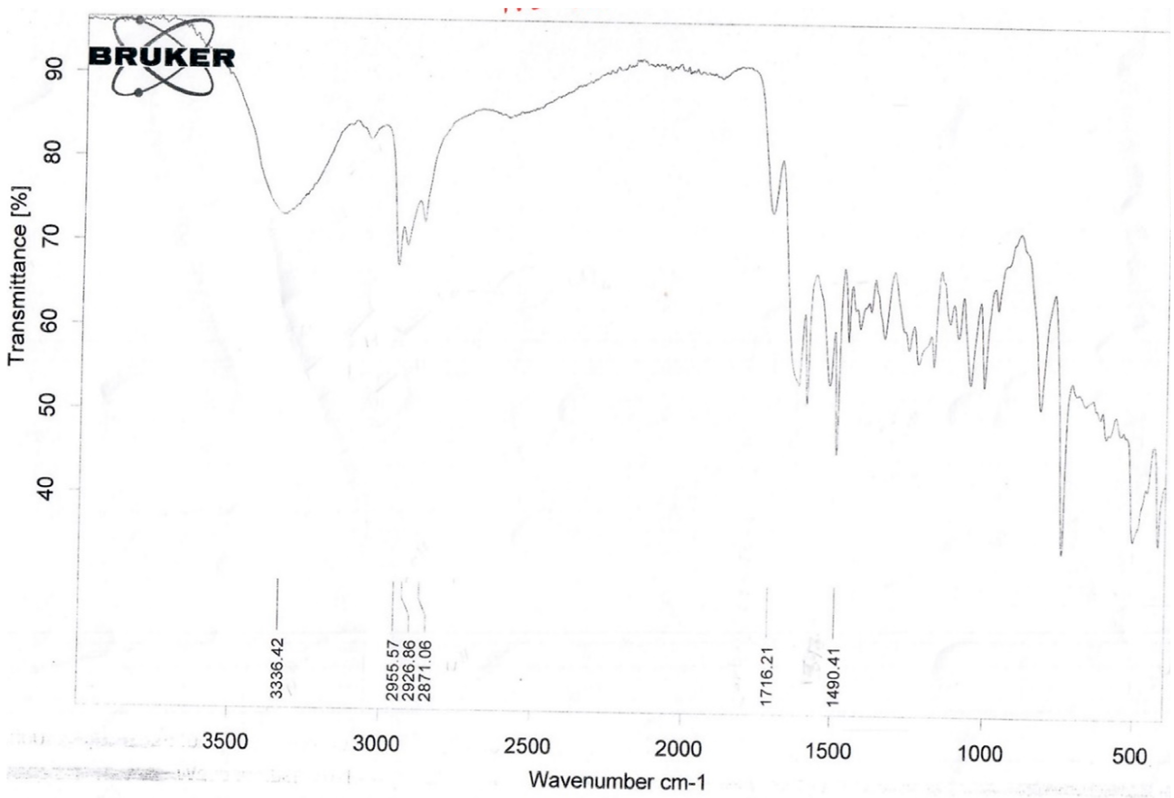

IR chart of compound 6.

09-Jan-07 18:25:29

Cairo University
Micro Analytical Center

DI Analysis
Shimadzu Qp-2010 Plus

Sample Information

Analyzed by	: Dr. Mai Younis
Analyzed	: 09/01/2007 06:16:16 م
Sample Name	: M4
Sample ID	:
Customer Name	: Dr.Mohamed Soliman - Science - Cairo
Data File	: C:\GCMSsolution\Data\Project1\M4.QGD
Org Data File	: C:\GCMSsolution\Data\Project1\M4.QGD
Method File	: C:\GCMSsolution\Data\Project1\High Temperature Op
Org Method File	: C:\GCMSsolution\Data\Project1\High Temperature Op
Report File	:
Tuning File	: C:\GCMSsolution\System\Tune1_default.qgt
$EndIf$Modified by	: Dr. Mai Younis
Modified	: 09/01/2007 06:20:58 م

Method

——— Analytical Line 1 ———

IonSourceTemp	:250.00 °C
[MS Table]	
--Group 1 - Event 1--	
Start Time	:0.00min
End Time	:10.00min
ACQ Mode	:Scan
Event Time	:0.50sec
Scan Speed	:1000
Start m/z	:50.00
End m/z	:510.00
Electron Voltage	: 70 eV
Ionization Mode	: EI

C:\GCMSsolution\Data\Project1\M4.QGD

Chromatogram M4 C:\GCMSsolution\Data\Project1\M4.QGD

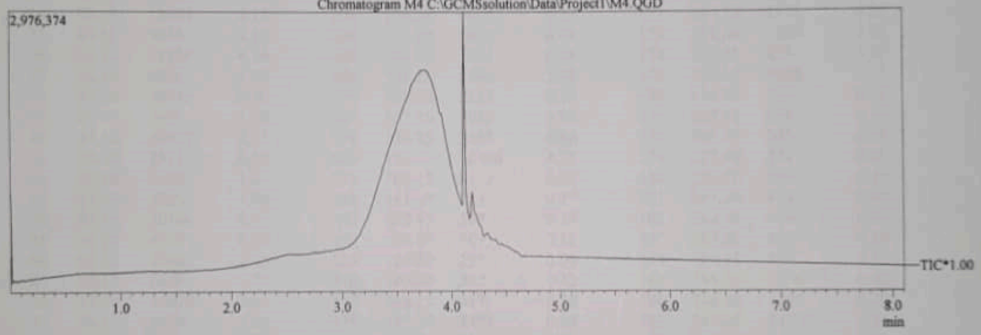

Spectrum

Line#:1 R.Time:3.8(Scan#:462)
MassPeaks:330(Peak Elimination m/z: 467.60, 468.60, 469.55, 477.60, 479.60, 480.60, 481.60, 485.60)
RawMode:Single 3.8(462) BasePeak:59(436001)
BG Mode:None Group 1 - Event 1

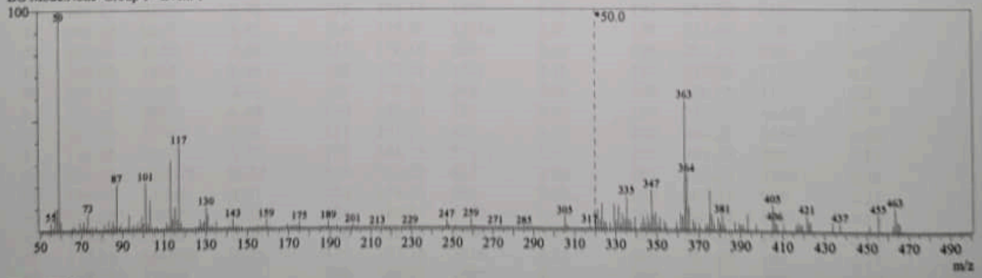

Mass Table
Line#:1 R.Time:3.8(Scan#:462)
MassPeaks:330(Peak Elimination m/z: 467.60, 468.60, 469.55, 477.60, 479.60, 480.60, 481.60, 485.60)
RawMode:Single 3.8(462) BasePeak:59(436001)
BG Mode:None Group 1 - Event 1

#	m/z	Abs. In	Rel. Int.	#	m/z	Abs. In	Rel. Int.	#	m/z	Abs. In	Rel. Int.
1	50.05	1816	0.42	4	53.05	1866	0.43	7	56.15	5626	1.29
2	51.00	2527	0.58	5	54.15	2543	0.58	8	57.05	43118	9.89
3	52.05	2240	0.51	6	55.05	15825	3.63	9	58.15	43832	10.05

1 / 3

Mass chart of compound 6.

#	m/z	Abs. In	Rel. Int.	#	m/z	Abs. In	Rel. Int.	#	m/z	Abs. In	Rel. Int.
10	59.05	436001	100.00	79	128.20	10748	2.47	148	198.20	242	0.06
11	60.05	17815	4.09	80	129.20	14495	3.32	149	199.15	789	0.18
12	61.05	4030	0.92	81	130.15	43369	9.95	150	200.25	567	0.13
13	62.05	1176	0.27	82	131.15	29113	6.68	151	201.20	5857	1.34
14	63.05	2800	0.64	83	132.15	4590	1.05	152	202.15	1228	0.28
15	64.10	1551	0.36	84	133.20	7732	1.77	153	203.20	2090	0.48
16	65.10	5146	1.18	85	134.25	1702	0.39	154	204.15	710	0.16
17	66.10	7649	1.75	86	135.20	15047	3.45	155	205.20	590	0.14
18	67.10	5202	1.19	87	136.25	1511	0.35	156	207.00	338	0.08
19	68.15	2165	0.50	88	137.20	1518	0.35	157	209.00	682	0.16
20	69.05	14302	3.28	89	138.25	617	0.14	158	211.05	539	0.12
21	70.10	6593	1.51	90	139.15	3237	0.74	159	212.20	250	0.06
22	71.05	16994	3.90	91	140.15	1596	0.37	160	213.20	2094	0.48
23	72.15	6785	1.56	92	141.15	4820	1.11	161	214.15	402	0.09
24	73.10	32164	7.38	93	142.15	3122	0.72	162	215.15	698	0.16
25	74.10	4052	0.93	94	143.15	19142	4.39	163	216.20	289	0.07
26	75.10	7815	1.79	95	144.20	3844	0.88	164	217.20	1919	0.44
27	76.10	1767	0.41	96	145.15	7034	1.61	165	218.25	377	0.09
28	77.05	7810	1.79	97	146.20	2071	0.47	166	219.20	737	0.17
29	78.05	2066	0.47	98	147.15	3068	0.70	167	221.20	244	0.06
30	79.15	2355	0.54	99	148.20	428	0.10	168	226.20	422	0.10
31	80.10	2015	0.46	100	149.20	622	0.14	169	227.25	1789	0.41
32	81.10	10654	2.44	101	150.15	540	0.12	170	228.15	668	0.15
33	82.15	3002	0.69	102	151.15	2933	0.67	171	229.30	2366	0.54
34	83.10	18004	4.13	103	152.20	1010	0.23	172	230.25	1114	0.26
35	84.15	5048	1.16	104	153.20	1930	0.44	173	231.30	1292	0.30
36	85.10	18129	4.16	105	154.25	1221	0.28	174	232.35	438	0.10
37	86.15	8280	1.90	106	155.15	5486	1.26	175	233.30	1628	0.37
38	87.10	90745	20.81	107	156.20	2332	0.53	176	234.35	539	0.12
39	88.10	5670	1.30	108	157.15	3950	0.91	177	235.35	638	0.15
40	89.10	10927	2.51	109	158.25	2435	0.56	178	236.40	343	0.08
41	90.10	2511	0.58	110	159.15	18348	4.21	179	237.40	271	0.06
42	91.10	5359	1.23	111	160.15	2311	0.53	180	239.20	503	0.12
43	92.15	5225	1.20	112	161.15	3343	0.77	181	241.10	414	0.09
44	93.10	30106	6.91	113	162.15	568	0.13	182	242.10	306	0.07
45	94.10	4202	0.96	114	163.20	769	0.18	183	243.30	815	0.19
46	95.10	10682	2.45	115	164.20	255	0.06	184	244.35	506	0.12
47	96.15	3429	0.79	116	165.20	522	0.12	185	245.30	1836	0.42
48	97.10	14078	3.23	117	166.15	417	0.10	186	246.35	1250	0.29
49	98.15	5635	1.29	118	167.15	1105	0.25	187	247.25	14552	3.34
50	99.10	26954	6.18	119	168.15	996	0.23	188	248.20	4612	1.06
51	100.15	13281	3.05	120	169.15	6481	1.49	189	249.25	1137	0.26
52	101.10	93946	21.55	121	170.10	1994	0.46	190	250.35	497	0.11
53	102.15	12014	2.76	122	171.15	4688	1.08	191	251.30	3265	0.75
54	103.15	59572	13.66	123	172.15	2573	0.59	192	252.30	716	0.16
55	104.15	5157	1.18	124	173.20	5599	1.28	193	253.30	332	0.08
56	105.15	2101	0.48	125	174.25	1883	0.43	194	254.30	210	0.05
57	106.10	6217	1.43	126	175.20	12236	2.81	195	255.40	518	0.12
58	107.15	2520	0.58	127	176.15	1891	0.43	196	256.35	356	0.08
59	108.20	1471	0.34	128	177.15	1521	0.35	197	257.25	1132	0.26
60	109.15	3448	0.79	129	178.20	268	0.06	198	258.35	1132	0.26
61	110.15	2073	0.48	130	180.20	215	0.05	199	259.25	16443	3.77
62	111.10	14743	3.38	131	181.10	426	0.10	200	260.25	3476	0.80
63	112.15	7557	1.73	132	182.10	564	0.13	201	261.30	2927	0.67
64	113.15	136634	31.34	133	183.15	887	0.20	202	262.25	1230	0.28
65	114.15	21396	4.91	134	184.10	945	0.22	203	263.25	988	0.23
66	115.15	44000	10.09	135	185.15	1757	0.40	204	264.30	812	0.19
67	116.25	18564	4.26	136	186.15	1969	0.45	205	265.30	1057	0.24
68	117.15	166559	38.20	137	187.15	3516	0.81	206	266.20	481	0.11
69	118.15	13405	3.07	138	188.25	1094	0.25	207	267.25	445	0.10
70	119.15	4367	1.00	139	189.20	13610	3.12	208	268.30	202	0.05
71	120.15	2080	0.48	140	190.10	2159	0.50	209	269.20	652	0.15
72	121.20	1478	0.34	141	191.20	1318	0.30	210	270.35	354	0.08
73	122.25	561	0.13	142	192.25	331	0.08	211	271.25	2718	0.62
74	123.25	1731	0.40	143	193.25	4600	1.06	212	272.25	847	0.19
75	124.25	840	0.19	144	194.15	663	0.15	213	273.30	2079	0.48
76	125.15	3208	0.74	145	195.00	406	0.09	214	274.25	816	0.19
77	126.25	4230	0.97	146	196.00	202	0.05	215	275.30	1678	0.38
78	127.15	19386	4.45	147	197.10	410	0.09	216	276.25	792	0.18

Mass chart of compound 6.

09-Jan-07 18:25:29

#	m/z	Abs. In	Rel. Int.
217	277.30	2113	0.48
218	278.25	599	0.14
219	279.25	569	0.13
220	280.20	241	0.06
221	281.10	393	0.09
222	282.15	399	0.09
223	283.20	466	0.11
224	284.25	422	0.10
225	285.30	2534	0.58
226	286.30	1335	0.31
227	287.25	2236	0.51
228	288.25	1106	0.25
229	289.30	2530	0.58
230	290.25	926	0.21
231	291.30	1562	0.36
232	292.25	924	0.21
233	293.30	1343	0.31
234	294.25	314	0.07
235	295.10	412	0.09
236	296.10	228	0.05
237	297.30	519	0.12
238	298.30	271	0.06
239	299.40	628	0.14
240	300.40	292	0.07
241	301.35	748	0.17
242	302.35	483	0.11
243	303.35	2180	0.50
244	304.35	1178	0.27
245	305.35	23581	5.41
246	306.30	6982	1.60
247	307.35	1776	0.41
248	308.30	257	0.06
249	309.30	786	0.18
250	310.25	322	0.07
251	311.30	314	0.07
252	313.45	1021	0.23
253	314.40	271	0.06
254	315.40	553	0.13

#	m/z	Abs. In	Rel. Int.
255	316.45	460	0.11
256	317.35	6775	1.55
257	318.35	1576	0.36
258	319.30	1070	0.25
259	320.25	322	0.07
260	321.35	775	0.18
261	322.45	375	0.09
262	323.40	1015	0.23
263	324.40	262	0.06
264	325.40	268	0.06
265	327.40	222	0.05
266	329.35	956	0.22
267	330.40	345	0.08
268	331.40	914	0.21
269	332.40	215	0.05
270	333.40	609	0.14
271	334.45	399	0.09
272	335.40	1287	0.30
273	336.35	393	0.09
274	337.40	306	0.07
275	339.45	478	0.11
276	342.40	241	0.06
277	343.40	494	0.11
278	344.40	242	0.06
279	345.45	545	0.12
280	346.45	282	0.06
281	347.40	1570	0.36
282	348.35	562	0.13
283	349.40	638	0.15
284	350.40	210	0.05
285	351.40	479	0.11
286	353.40	348	0.08
287	354.40	234	0.05
288	359.40	305	0.07
289	361.40	649	0.15
290	362.45	551	0.13
291	363.40	5110	1.17
292	364.35	2196	0.50

#	m/z	Abs. In	Rel. Int.
293	365.40	918	0.21
294	367.40	345	0.08
295	368.40	241	0.06
296	370.40	226	0.05
297	373.40	222	0.05
298	374.40	241	0.06
299	375.40	1564	0.36
300	376.40	604	0.14
301	377.40	278	0.06
302	379.40	502	0.12
303	380.40	270	0.06
304	381.40	593	0.14
305	382.40	236	0.05
306	387.40	327	0.07
307	389.40	287	0.07
308	390.40	206	0.05
309	391.40	239	0.05
310	393.40	642	0.15
311	397.40	258	0.06
312	405.40	1007	0.23
313	406.35	332	0.08
314	407.40	278	0.06
315	410.40	258	0.06
316	416.40	210	0.05
317	417.40	311	0.07
318	418.40	244	0.06
319	419.40	236	0.05
320	421.45	611	0.14
321	422.40	366	0.08
322	423.40	353	0.08
323	433.65	357	0.08
324	437.20	303	0.07
325	451.20	226	0.05
326	455.50	652	0.15
327	462.50	231	0.05
328	463.50	919	0.21
329	464.60	385	0.09
330	465.60	335	0.08

Mass chart of compound 6.

4. -((2-Oxo-2-(phenylamino)ethyl)selanyl)phenyl)carbonohydrazonoyl dicyanide (7)

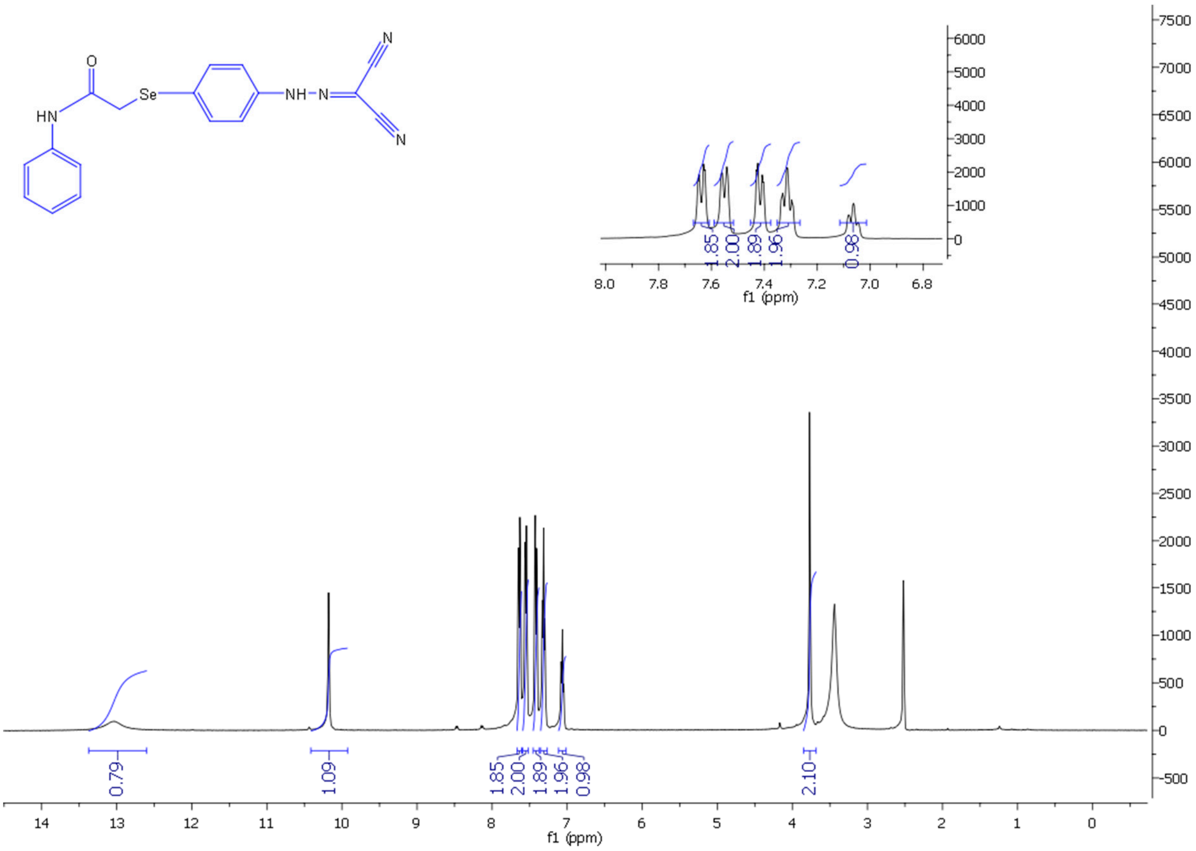

^{1}HNMR chart of compound 7.

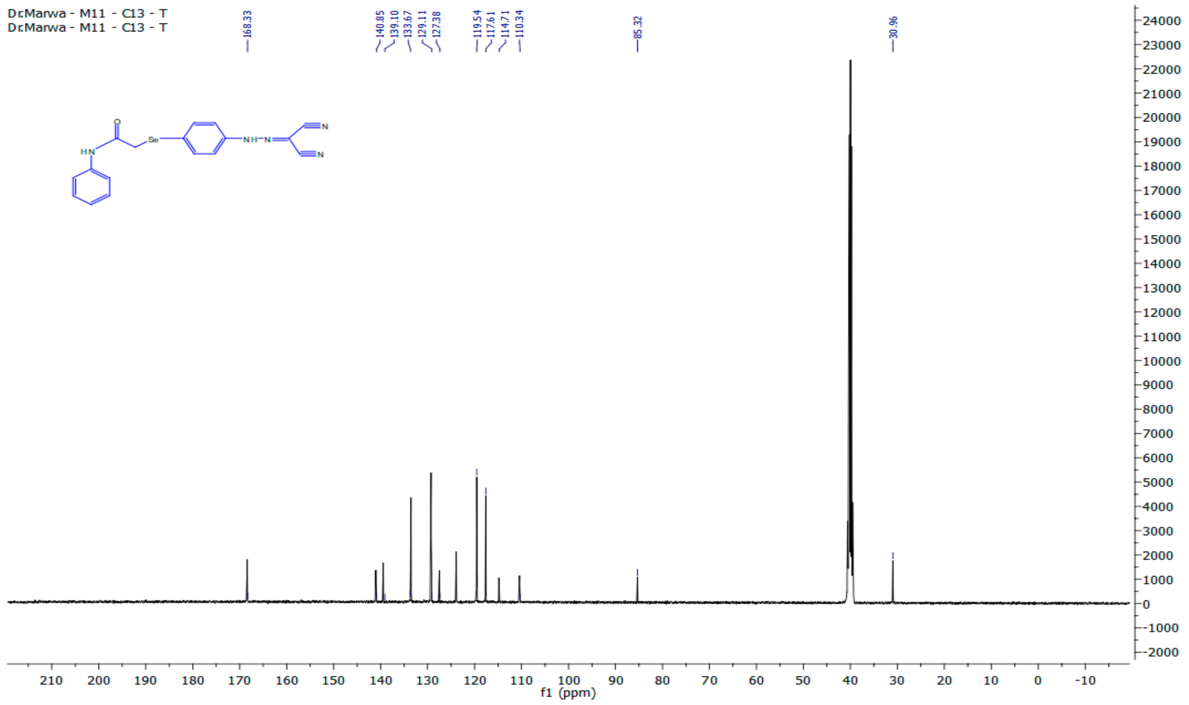

^{13}CNMR chart of compound 7.

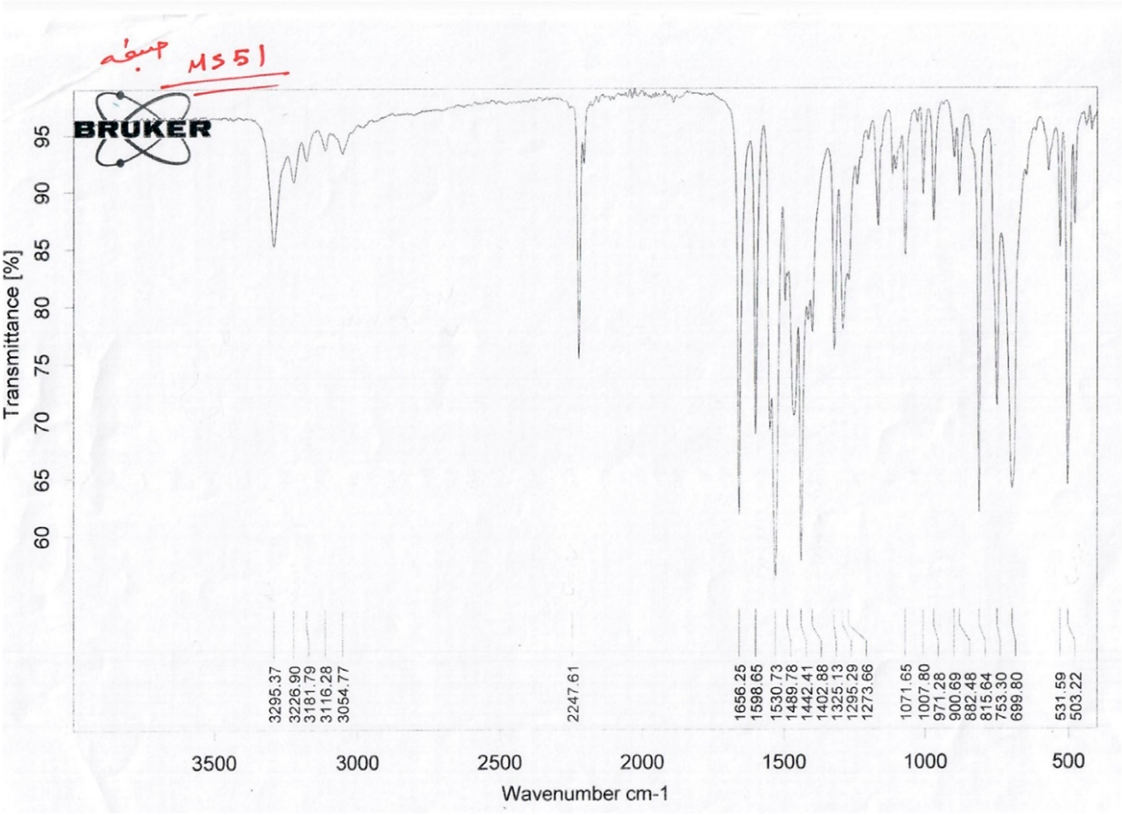

IR chart of compound 7.

03-Jan-07 19:33:04

Cairo University
Micro Analytical Center

DI Analysis
Shimadzu Qp-2010 Plus

Sample Information

Analyzed by	: Dr. Mai Younis
Analyzed	: 03/01/2007 07:14:31 ,
Sample Name	: M11
Sample ID	:
Customer Name	: Dr.Mohamed Soliman - Science - Cairo
Data File	: C:\GCMSsolution\Data\Project1\M11.QGD
Org Data File	: C:\GCMSsolution\Data\Project1\M11.QGD
Method File	: C:\GCMSsolution\Data\Project1\High Temperature Op
Org Method File	: C:\GCMSsolution\Data\Project1\High Temperature Op
Report File	:
Tuning File	: C:\GCMSsolution\System\Tune1_default.qgt
$EndIf$Modified by	: Dr. Mai Younis
Modified	: 03/01/2007 07:22:20 ,

Method

——— Analytical Line 1 ———

IonSourceTemp	:250.00 °C
[MS Table]	
--Group 1 - Event 1--	
Start Time	:0.00min
End Time	:10.00min
ACQ Mode	:Scan
Event Time	:0.50sec
Scan Speed	:1000
Start m/z	:50.00
End m/z	:510.00
Electron Voltage	: 70 eV
Ionization Mode	: EI

C:\GCMSsolution\Data\Project1\M11.QGD

Chromatogram M11 C:\GCMSsolution\Data\Project1\M11.QGD

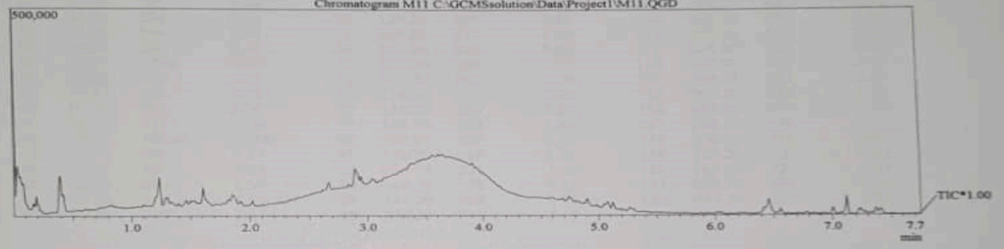

Spectrum

Line#:1 R.Time:2.7(Scan#:323)
MassPeaks:122
RawMode:Single 2.7(323) BasePeak:93(10830)
BG Mode:None Group 1 - Event 1

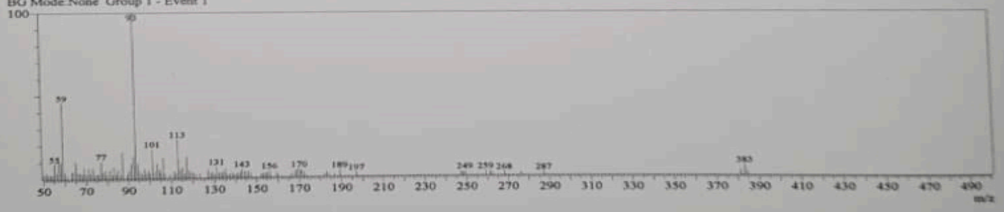

Mass Table
Line#:1 R.Time:2.7(Scan#:323)
MassPeaks:122
RawMode:Single 2.7(323) BasePeak:93(10830)
BG Mode:None Group 1 - Event 1

#	m/z	Abs. In	Rel. Int.	#	m/z	Abs. In	Rel. Int.	#	m/z	Abs. In	Rel. Int.
1	50.00	276	2.55	4	53.00	279	2.58	7	56.00	404	3.73
2	51.00	415	3.83	5	54.00	260	2.40	8	57.05	1145	10.57
3	52.00	265	2.45	6	55.05	975	9.00	9	58.05	1057	9.76

1 / 2

Mass chart of compound 7.

03-Jan-07 19:33:04

#	m/z	Abs. In	Rel. Int.
10	59.00	4956	45.76
11	59.95	445	4.11
12	61.00	268	2.47
13	63.10	476	4.40
14	64.00	457	4.22
15	65.00	1063	9.82
16	66.00	394	3.64
17	67.00	385	3.55
18	68.00	297	2.74
19	69.00	735	6.79
20	69.95	318	2.94
21	71.10	706	6.52
22	72.10	329	3.04
23	73.10	671	6.20
24	74.10	214	1.98
25	75.10	306	2.83
26	76.00	282	2.60
27	77.00	1119	10.33
28	78.00	423	3.91
29	79.00	508	4.69
30	80.00	271	2.50
31	81.15	575	5.31
32	82.10	241	2.23
33	83.10	761	7.03
34	84.10	284	2.62
35	85.05	568	5.24
36	86.20	250	2.31
37	87.15	1738	16.05
38	89.20	257	2.37
39	90.15	506	4.67
40	91.15	952	8.79
41	92.20	1417	13.08
42	93.15	10830	100.00
43	94.20	990	9.14
44	95.25	481	4.44
45	96.30	281	2.59
46	97.25	610	5.63
47	98.30	337	3.11

#	m/z	Abs. In	Rel. Int.
48	99.30	497	4.59
49	100.15	337	3.11
50	101.15	1897	17.52
51	102.25	429	3.96
52	103.25	940	8.68
53	104.25	578	5.34
54	105.25	378	3.49
55	106.15	1352	12.48
56	107.20	282	2.60
57	109.20	228	2.11
58	111.30	452	4.17
59	112.25	313	2.89
60	113.15	2515	23.22
61	114.25	529	4.88
62	115.20	692	6.39
63	116.25	328	3.03
64	117.20	1399	12.92
65	118.20	514	4.75
66	119.20	366	3.38
67	120.35	381	3.52
68	121.30	247	2.28
69	123.30	273	2.52
70	127.20	513	4.74
71	128.20	322	2.97
72	129.20	391	3.61
73	130.20	212	1.96
74	131.20	721	6.66
75	132.20	298	2.75
76	133.20	345	3.19
77	134.20	375	3.46
78	135.20	564	5.21
79	136.20	202	1.87
80	137.20	234	2.16
81	138.20	316	2.92
82	139.20	231	2.13
83	140.20	230	2.12
84	141.20	305	2.82
85	142.20	340	3.14

#	m/z	Abs. In	Rel. Int.
86	143.10	542	5.00
87	144.10	401	3.70
88	145.10	374	3.45
89	146.10	401	3.70
90	147.10	287	2.65
91	152.10	247	2.28
92	153.10	295	2.72
93	154.10	295	2.72
94	155.10	274	2.53
95	156.10	410	3.79
96	159.10	308	2.84
97	160.10	204	1.88
98	166.10	247	2.28
99	168.10	406	3.75
100	169.00	489	4.52
101	170.05	502	4.64
102	171.00	430	3.97
103	172.00	342	3.16
104	173.00	218	2.01
105	182.00	220	2.03
106	183.00	306	2.83
107	184.00	206	1.90
108	187.00	212	1.96
109	189.20	426	3.93
110	197.20	270	2.49
111	247.20	239	2.21
112	248.20	218	2.01
113	249.20	278	2.57
114	259.20	281	2.59
115	261.20	230	2.12
116	262.20	249	2.30
117	268.20	230	2.12
118	276.20	210	1.94
119	287.20	220	2.03
120	381.20	329	3.04
121	383.30	663	6.12
122	384.30	279	2.58

Mass chart of compound 7.

2. -((4-(2-(3-Methyl-5-oxo-1-phenyl-1H-pyrazol-4(5H)-ylidene)hydrazinyl)phenyl)selanyl)-N-phenylacetamide (8)

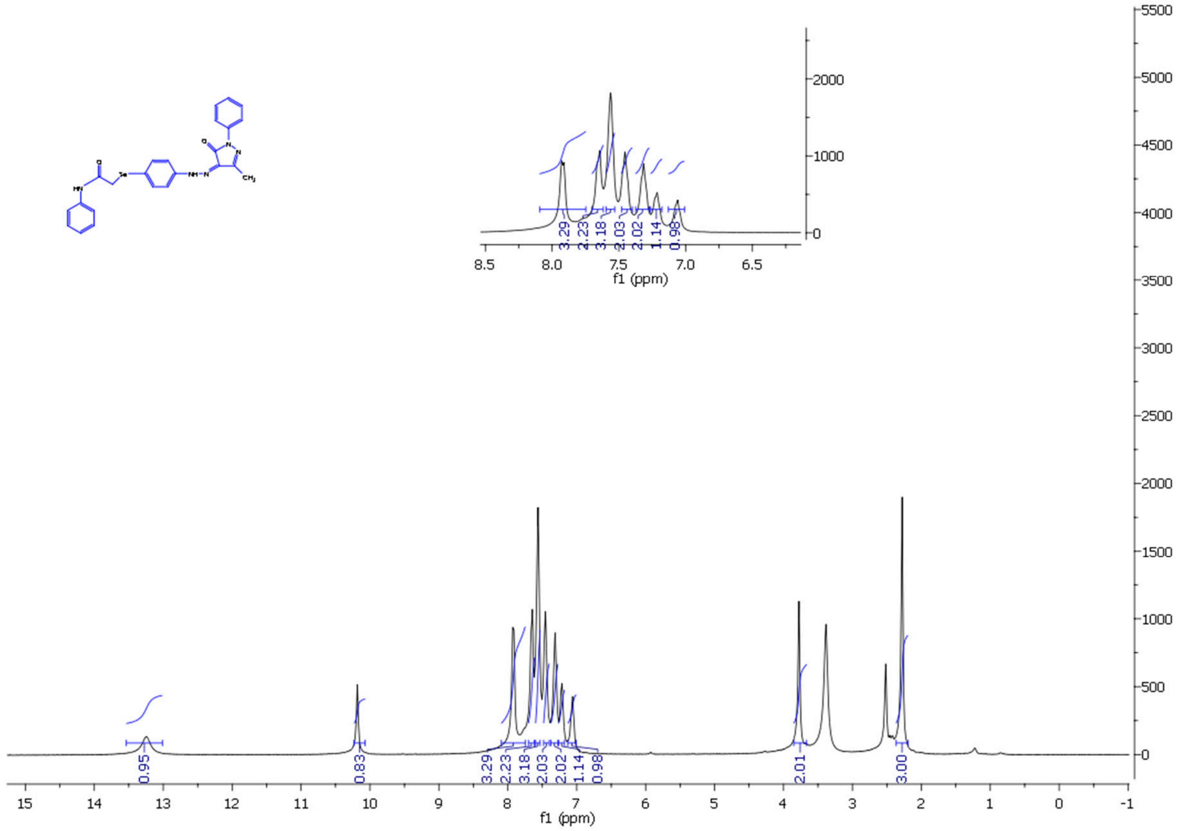

^{1}HNMR chart of compound 8.

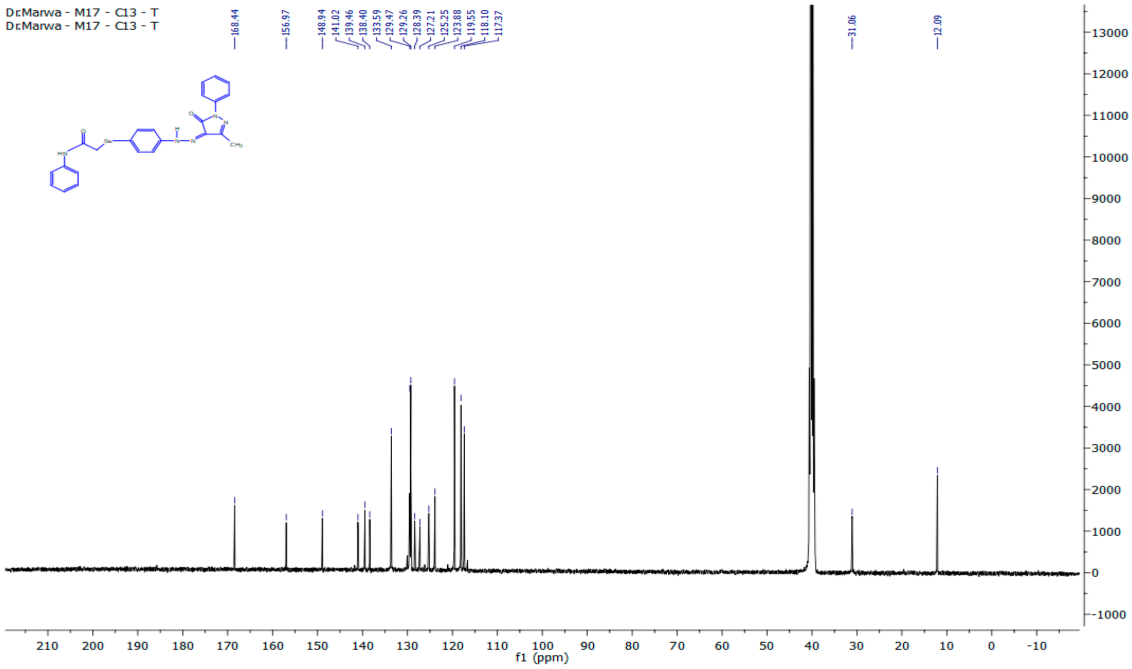

^{13}CNMR chart of compound 8.

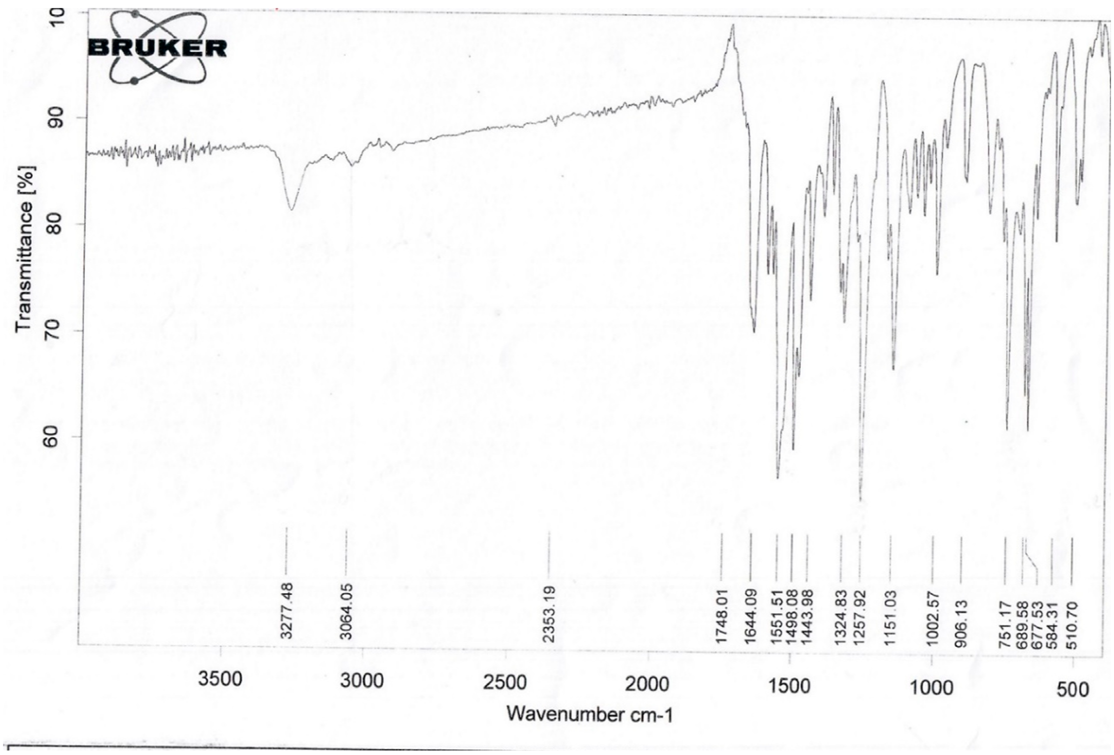

IR chart of compound 8.

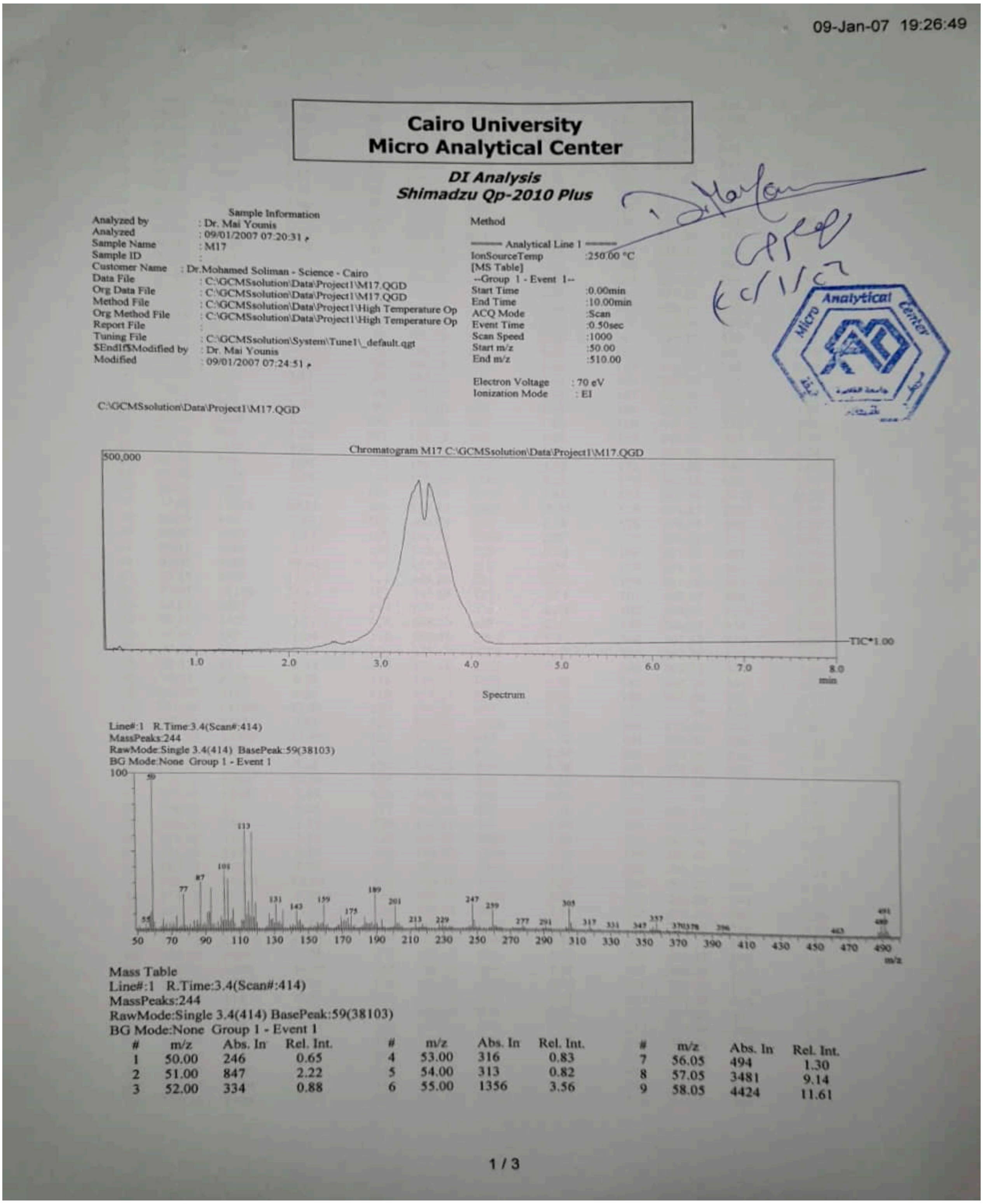

09-Jan-07 19:26:49

Cairo University
Micro Analytical Center

DI Analysis
Shimadzu Qp-2010 Plus

Sample Information

Analyzed by	: Dr. Mai Younis
Analyzed	: 09/01/2007 07:20:31
Sample Name	: M17
Sample ID	:
Customer Name	: Dr.Mohamed Soliman - Science - Cairo
Data File	: C:\GCMSsolution\Data\Project1\M17.QGD
Org Data File	: C:\GCMSsolution\Data\Project1\M17.QGD
Method File	: C:\GCMSsolution\Data\Project1\High Temperature Op
Org Method File	: C:\GCMSsolution\Data\Project1\High Temperature Op
Report File	:
Tuning File	: C:\GCMSsolution\System\Tune1_default.qgt
$EndIf$Modified by	: Dr. Mai Younis
Modified	: 09/01/2007 07:24:51

Method

——— Analytical Line 1 ———

IonSourceTemp	:250.00 °C
[MS Table]	
--Group 1 - Event 1--	
Start Time	:0.00min
End Time	:10.00min
ACQ Mode	:Scan
Event Time	:0.50sec
Scan Speed	:1000
Start m/z	:50.00
End m/z	:510.00
Electron Voltage	: 70 eV
Ionization Mode	: EI

C:\GCMSsolution\Data\Project1\M17.QGD

Chromatogram M17 C:\GCMSsolution\Data\Project1\M17.QGD

Spectrum

Line#:1 R.Time:3.4(Scan#:414)
MassPeaks:244
RawMode:Single 3.4(414) BasePeak:59(38103)
BG Mode:None Group 1 - Event 1

Mass Table
Line#:1 R.Time:3.4(Scan#:414)
MassPeaks:244
RawMode:Single 3.4(414) BasePeak:59(38103)
BG Mode:None Group 1 - Event 1

#	m/z	Abs. In	Rel. Int.	#	m/z	Abs. In	Rel. Int.	#	m/z	Abs. In	Rel. Int.
1	50.00	246	0.65	4	53.00	316	0.83	7	56.05	494	1.30
2	51.00	847	2.22	5	54.00	313	0.82	8	57.05	3481	9.14
3	52.00	334	0.88	6	55.00	1356	3.56	9	58.05	4424	11.61

1 / 3

MS chart of compound 8.

#	m/z	Abs. In	Rel. Int.	#	m/z	Abs. In	Rel. Int.	#	m/z	Abs. In	Rel. Int.
10	59.00	38103	100.00	79	131.15	6160	16.17	148	203.20	1551	4.07
11	59.95	1820	4.78	80	132.15	1653	4.34	149	204.20	647	1.70
12	60.95	422	1.11	81	133.15	1963	5.15	150	205.20	465	1.22
13	62.95	680	1.78	82	134.20	1257	3.30	151	211.20	254	0.67
14	64.05	865	2.27	83	135.15	4635	12.16	152	213.20	1214	3.19
15	65.00	2724	7.15	84	136.20	401	1.05	153	214.20	294	0.77
16	66.00	710	1.86	85	137.20	295	0.77	154	215.30	438	1.15
17	67.00	1353	3.55	86	139.20	812	2.13	155	216.35	353	0.93
18	68.00	279	0.73	87	140.15	521	1.37	156	217.30	1161	3.05
19	69.00	1503	3.94	88	141.10	1206	3.17	157	218.20	366	0.96
20	70.05	667	1.75	89	142.15	853	2.24	158	219.20	582	1.53
21	71.00	1618	4.25	90	143.15	4414	11.58	159	220.20	206	0.54
22	72.05	776	2.04	91	144.15	1768	4.64	160	227.25	1026	2.69
23	73.00	3345	8.78	92	145.15	2326	6.10	161	228.35	302	0.79
24	74.15	338	0.89	93	146.15	1084	2.84	162	229.30	1054	2.77
25	75.05	948	2.49	94	147.15	932	2.45	163	230.35	438	1.15
26	76.05	766	2.01	95	148.10	238	0.62	164	231.30	663	1.74
27	77.00	8690	22.81	96	149.10	233	0.61	165	232.40	260	0.68
28	78.00	1186	3.11	97	150.10	257	0.67	166	233.40	567	1.49
29	79.05	880	2.31	98	151.10	716	1.88	167	234.40	522	1.37
30	80.05	391	1.03	99	152.10	337	0.88	168	235.35	454	1.19
31	81.00	1228	3.22	100	153.20	695	1.82	169	241.40	238	0.62
32	82.05	488	1.28	101	154.15	601	1.58	170	243.40	284	0.75
33	83.05	2157	5.66	102	155.20	1526	4.00	171	244.30	282	0.74
34	84.05	724	1.90	103	156.10	783	2.05	172	245.30	780	2.05
35	85.05	2125	5.58	104	157.10	1242	3.26	173	246.35	672	1.76
36	86.05	1231	3.23	105	158.15	1393	3.66	174	247.30	6174	16.20
37	87.05	11510	30.21	106	159.15	6065	15.92	175	248.25	2275	5.97
38	88.05	944	2.48	107	160.05	791	2.08	176	249.35	645	1.69
39	89.05	1659	4.35	108	161.20	1094	2.87	177	250.30	215	0.56
40	90.15	1261	3.31	109	162.20	228	0.60	178	251.35	681	1.79
41	91.05	4162	10.92	110	163.20	212	0.56	179	254.30	201	0.53
42	92.15	4300	11.29	111	165.20	255	0.67	180	257.30	343	0.90
43	93.05	10139	26.61	112	166.15	438	1.15	181	258.35	408	1.07
44	94.05	1281	3.36	113	167.05	854	2.24	182	259.30	4655	12.22
45	95.10	1415	3.71	114	168.10	897	2.35	183	260.25	954	2.50
46	96.05	395	1.04	115	169.15	2995	7.86	184	261.30	778	2.04
47	97.05	1812	4.76	116	170.10	1742	4.57	185	262.20	478	1.25
48	98.15	821	2.15	117	171.10	2263	5.94	186	263.35	497	1.30
49	99.10	3154	8.28	118	172.10	1850	4.86	187	264.30	343	0.90
50	100.15	2187	5.74	119	173.15	2658	6.98	188	265.30	273	0.72
51	101.10	14188	37.24	120	174.25	932	2.45	189	271.30	561	1.47
52	102.15	2211	5.80	121	175.20	3206	8.41	190	272.30	254	0.67
53	103.10	12370	32.46	122	176.25	655	1.72	191	273.30	438	1.15
54	104.15	2058	5.40	123	177.20	852	2.24	192	274.30	218	0.57
55	105.15	2670	7.01	124	178.20	220	0.58	193	275.30	358	0.94
56	106.10	5052	13.26	125	179.20	230	0.60	194	276.30	223	0.59
57	107.10	1346	3.53	126	180.10	260	0.68	195	277.30	847	2.22
58	108.10	327	0.86	127	181.05	557	1.46	196	278.25	579	1.52
59	109.10	546	1.43	128	182.15	761	2.00	197	279.20	210	0.55
60	110.15	428	1.12	129	183.15	3002	7.88	198	285.40	340	0.89
61	111.10	2439	6.40	130	184.05	1349	3.54	199	289.40	382	1.00
62	112.15	2037	5.35	131	185.15	1189	3.12	200	290.20	281	0.74
63	113.10	24138	63.35	132	186.10	1230	3.23	201	291.20	769	2.02
64	114.15	3561	9.35	133	187.10	1543	4.05	202	292.20	244	0.64
65	115.15	6835	17.94	134	188.25	982	2.58	203	293.20	337	0.88
66	116.15	3243	8.51	135	189.20	8473	22.24	204	294.20	218	0.57
67	117.15	23756	62.35	136	190.15	1348	3.54	205	303.30	650	1.71
68	118.15	3004	7.88	137	191.15	567	1.49	206	304.35	520	1.36
69	119.10	6458	16.95	138	192.20	260	0.68	207	305.35	5423	14.23
70	120.10	1714	4.50	139	193.25	2305	6.05	208	306.20	1751	4.60
71	121.10	318	0.83	140	194.15	453	1.19	209	307.20	438	1.15
72	123.10	271	0.71	141	195.20	313	0.82	210	317.40	847	2.22
73	125.15	588	1.54	142	197.20	252	0.66	211	318.40	356	0.93
74	126.25	971	2.55	143	198.20	348	0.91	212	323.40	214	0.56
75	127.15	3951	10.37	144	199.15	372	0.98	213	331.40	270	0.71
76	128.20	2330	6.12	145	200.25	626	1.64	214	335.40	228	0.60
77	129.20	2634	6.91	146	201.20	5601	14.70	215	347.40	231	0.61
78	130.25	1484	3.89	147	202.15	1253	3.29	216	348.40	201	0.53

2 / 3

MS chart of compound 8.

09-Jan-07 19:26:49

#	m/z	Abs. In	Rel. Int.	#	m/z	Abs. In	Rel. Int.	#	m/z	Abs. In	Rel. Int.
217	353.30	439	1.15	227	368.40	254	0.67	237	487.35	1100	2.89
218	354.25	560	1.47	228	369.40	225	0.59	238	488.30	1206	3.17
219	355.20	1089	2.86	229	370.40	375	0.98	239	489.30	2599	6.82
220	356.35	512	1.34	230	375.40	233	0.61	240	490.45	1121	2.94
221	357.25	2074	5.44	231	378.40	207	0.54	241	491.35	5274	13.84
222	358.25	415	1.09	232	396.40	244	0.64	242	492.35	1586	4.16
223	359.30	380	1.00	233	397.40	214	0.56	243	493.30	1258	3.30
224	363.40	497	1.30	234	463.40	214	0.56	244	494.30	412	1.08
225	364.40	246	0.65	235	483.40	207	0.54				
226	367.40	214	0.56	236	485.40	210	0.55				

MS chart of compound 8.

2. -((4-(2-(3-Hydroxynaphthalen-1(4H)-ylidene)hydrazinyl)phenyl)selanyl)-N-phenylacetamide (9)

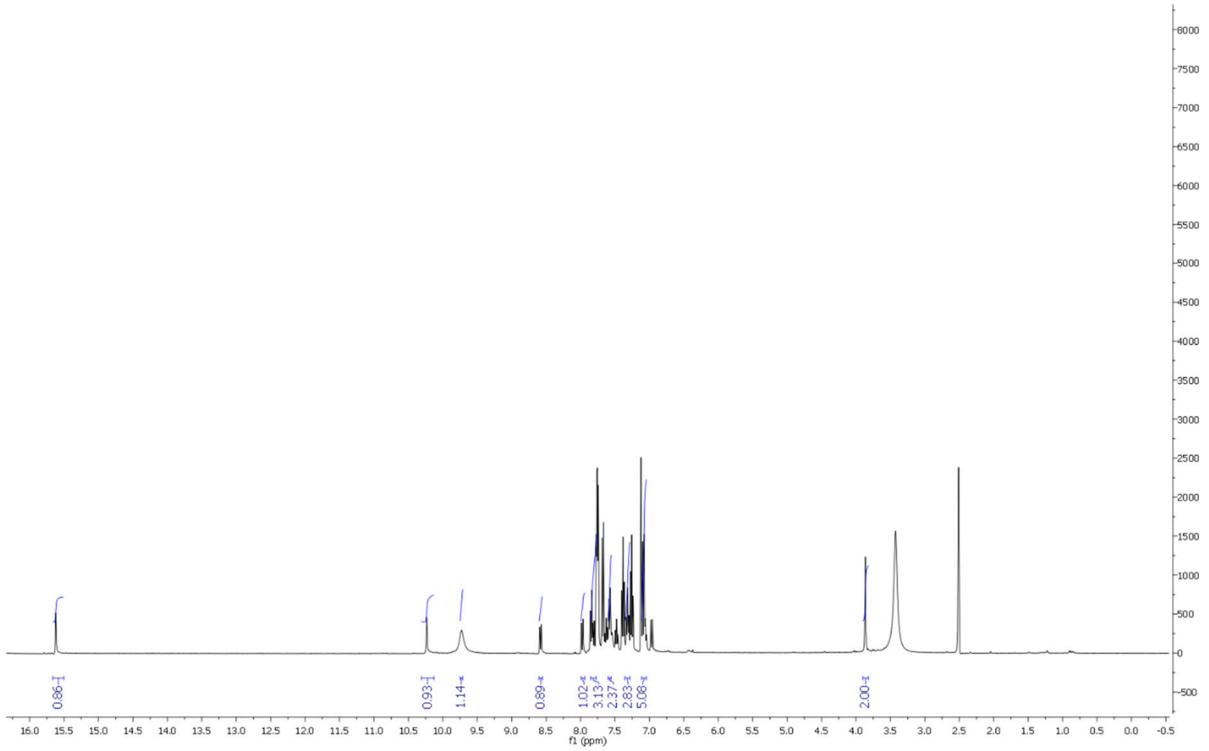

^{1}HNMR chart of compound 9.

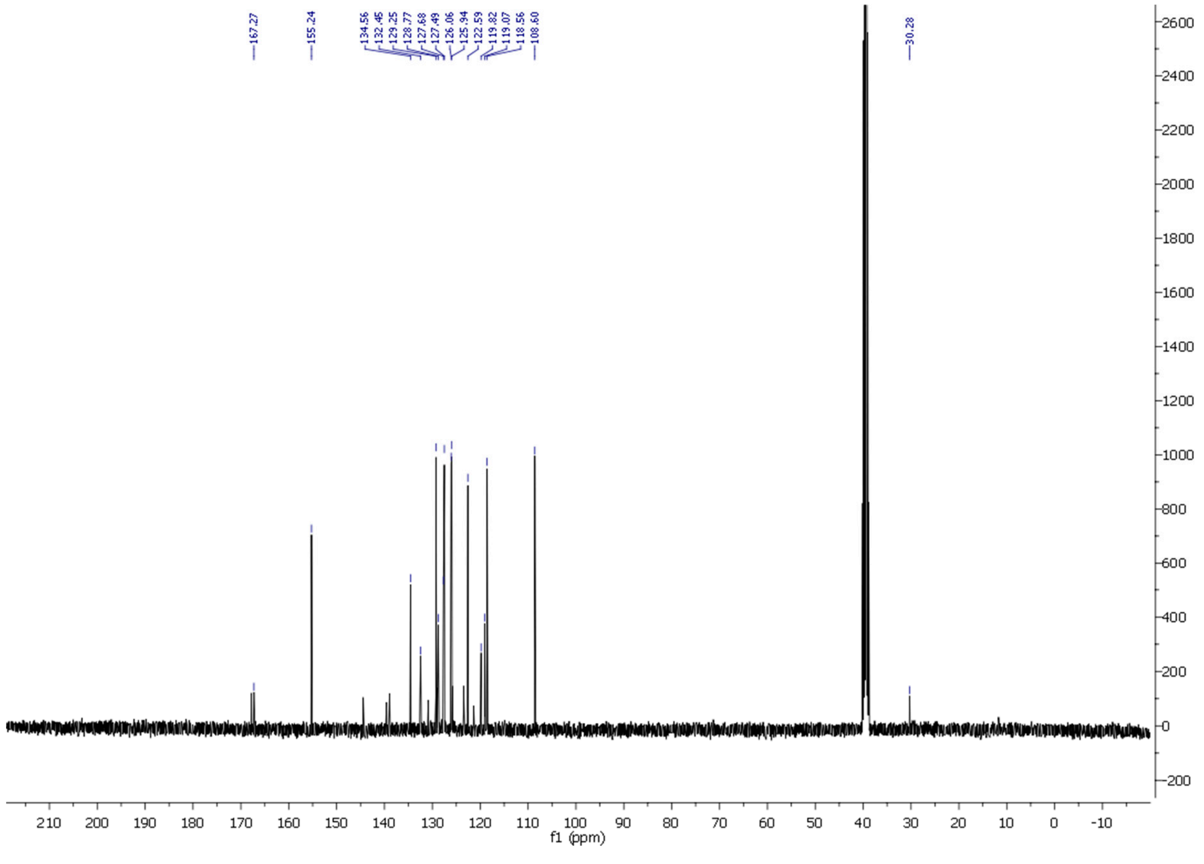

^{13}CNMR chart of compound 9.

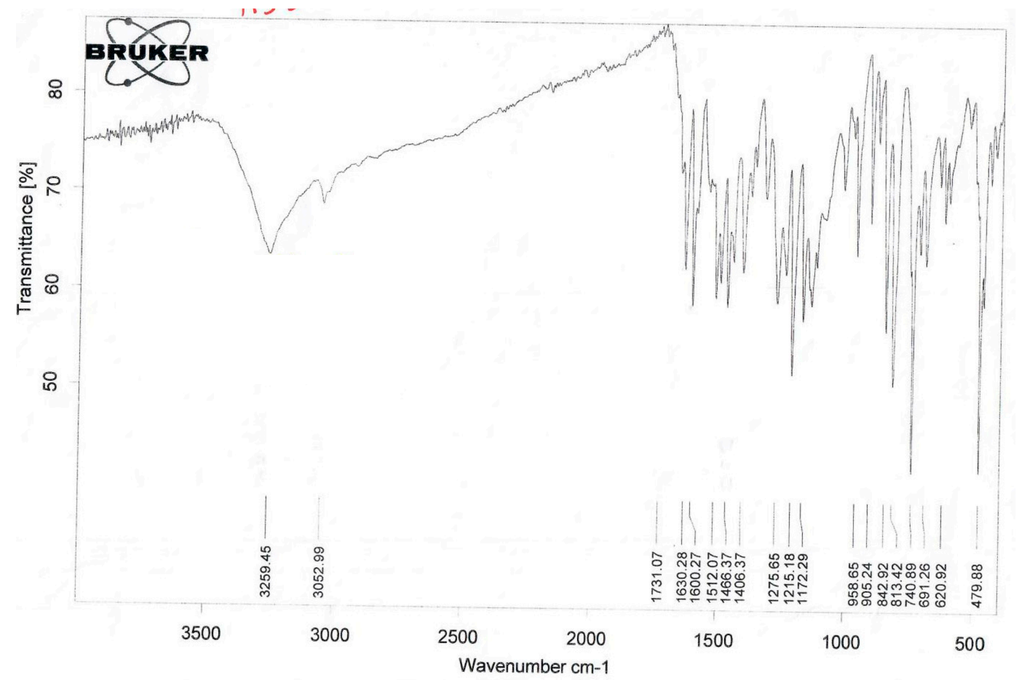

IR chart of compound 9.

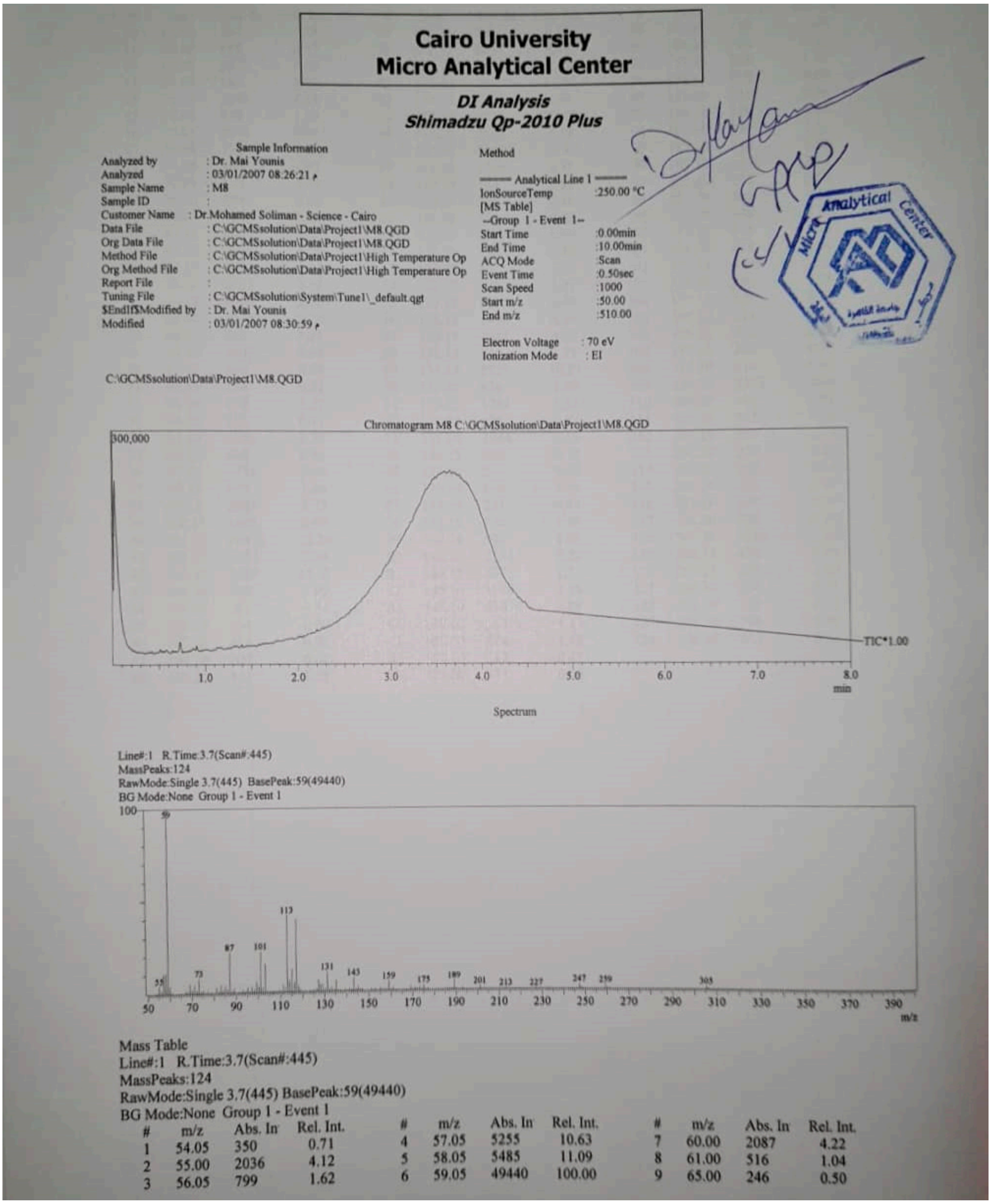

Cairo University
Micro Analytical Center

DI Analysis
Shimadzu Qp-2010 Plus

Sample Information

Analyzed by	: Dr. Mai Younis
Analyzed	: 03/01/2007 08:26:21
Sample Name	: M8
Sample ID	:
Customer Name	: Dr.Mohamed Soliman - Science - Cairo
Data File	: C:\GCMSsolution\Data\Project1\M8.QGD
Org Data File	: C:\GCMSsolution\Data\Project1\M8.QGD
Method File	: C:\GCMSsolution\Data\Project1\High Temperature Op
Org Method File	: C:\GCMSsolution\Data\Project1\High Temperature Op
Report File	:
Tuning File	: C:\GCMSsolution\System\Tune1_default.qgt
$EndIf$Modified by	: Dr. Mai Younis
Modified	: 03/01/2007 08:30:59

Method

—— Analytical Line 1 ——

IonSourceTemp	:250.00 °C
[MS Table]	
--Group 1 - Event 1--	
Start Time	:0.00min
End Time	:10.00min
ACQ Mode	:Scan
Event Time	:0.50sec
Scan Speed	:1000
Start m/z	:50.00
End m/z	:510.00
Electron Voltage	: 70 eV
Ionization Mode	: EI

C:\GCMSsolution\Data\Project1\M8.QGD

Mass Table
Line#:1 R.Time:3.7(Scan#:445)
MassPeaks:124
RawMode:Single 3.7(445) BasePeak:59(49440)
BG Mode:None Group 1 - Event 1

#	m/z	Abs. In	Rel. Int.	#	m/z	Abs. In	Rel. Int.	#	m/z	Abs. In	Rel. Int.
1	54.05	350	0.71	4	57.05	5255	10.63	7	60.00	2087	4.22
2	55.00	2036	4.12	5	58.05	5485	11.09	8	61.00	516	1.04
3	56.05	799	1.62	6	59.05	49440	100.00	9	65.00	246	0.50

Mass chart of compound 9.

03-Jan-07 20:34:23

#	m/z	Abs. In	Rel. Int.
10	67.05	754	1.53
11	68.05	481	0.97
12	69.05	2572	5.20
13	70.05	884	1.79
14	71.05	2249	4.55
15	72.05	818	1.65
16	73.05	3970	8.03
17	74.05	522	1.06
18	75.05	949	1.92
19	77.00	702	1.42
20	79.00	289	0.58
21	81.05	1491	3.02
22	82.15	495	1.00
23	83.10	2346	4.75
24	84.15	856	1.73
25	85.10	2065	4.18
26	86.15	1019	2.06
27	87.10	10767	21.78
28	88.15	597	1.21
29	89.10	1402	2.84
30	91.10	316	0.64
31	92.10	254	0.51
32	93.10	778	1.57
33	94.15	353	0.71
34	95.10	1380	2.79
35	96.15	400	0.81
36	97.10	1774	3.59
37	98.15	819	1.66
38	99.15	2842	5.75
39	100.15	1695	3.43
40	101.10	10977	22.20
41	102.15	1453	2.94
42	103.15	7600	15.37
43	104.10	492	1.00
44	105.10	268	0.54
45	106.10	294	0.59
46	107.20	406	0.82
47	108.20	207	0.42
48	109.20	551	1.11

#	m/z	Abs. In	Rel. Int.
49	110.15	358	0.72
50	111.15	2057	4.16
51	112.15	1294	2.62
52	113.10	20577	41.62
53	114.15	3228	6.53
54	115.15	6130	12.40
55	116.25	2562	5.18
56	117.15	19493	39.43
57	118.20	1634	3.31
58	119.15	784	1.59
59	120.20	238	0.48
60	121.20	335	0.68
61	123.20	377	0.76
62	124.20	238	0.48
63	125.15	504	1.02
64	126.25	697	1.41
65	127.15	3205	6.48
66	128.15	1996	4.04
67	129.15	2014	4.07
68	130.15	845	1.71
69	131.15	5225	10.57
70	132.20	636	1.29
71	133.20	1244	2.52
72	134.25	478	0.97
73	135.15	3044	6.16
74	136.15	370	0.75
75	137.20	273	0.55
76	139.10	670	1.36
77	140.10	222	0.45
78	141.15	730	1.48
79	142.15	506	1.02
80	143.10	3564	7.21
81	144.15	598	1.21
82	145.10	1274	2.58
83	146.10	436	0.88
84	147.10	559	1.13
85	151.10	574	1.16
86	152.10	212	0.43
87	153.00	473	0.96

#	m/z	Abs. In	Rel. Int.
88	155.10	738	1.49
89	156.10	302	0.61
90	157.15	568	1.15
91	158.15	315	0.64
92	159.10	2518	5.09
93	160.10	329	0.67
94	161.10	649	1.31
95	167.10	230	0.47
96	169.10	1058	2.14
97	170.10	295	0.60
98	171.15	598	1.21
99	172.10	378	0.76
100	173.15	878	1.78
101	174.20	218	0.44
102	175.15	1257	2.54
103	176.20	250	0.51
104	177.15	355	0.72
105	185.20	214	0.43
106	186.20	332	0.67
107	187.20	388	0.78
108	188.20	214	0.43
109	189.20	2372	4.80
110	190.15	403	0.82
111	193.10	505	1.02
112	201.15	857	1.73
113	203.20	327	0.66
114	213.20	268	0.54
115	219.20	220	0.44
116	227.20	247	0.50
117	233.20	239	0.48
118	247.20	1263	2.55
119	248.25	470	0.95
120	259.25	1032	2.09
121	260.30	254	0.51
122	261.30	215	0.43
123	305.30	788	1.59
124	306.30	313	0.63

Mass chart of compound 9.

4. -Oxo-4-((4-((2-oxo-2-(phenylamino)ethyl)selanyl)phenyl)amino)but-2-enoic acid (10)

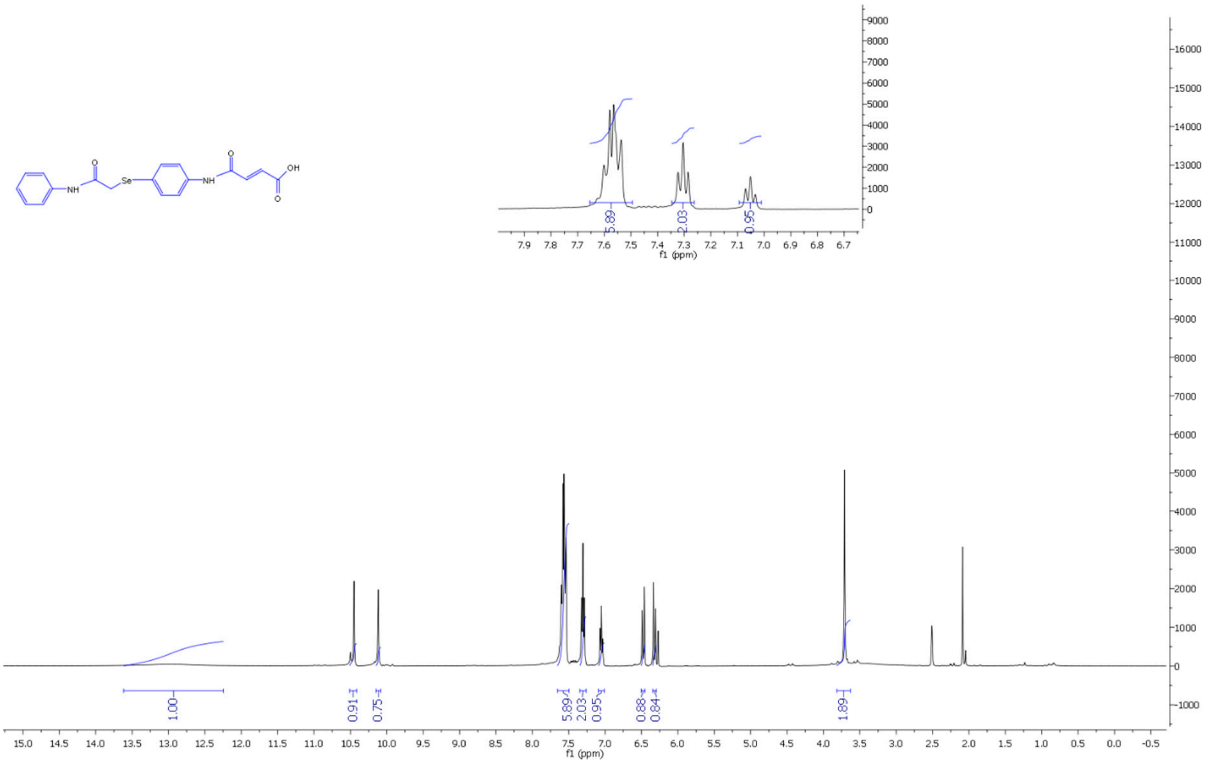

^{1}HNMR chart of compound 10.

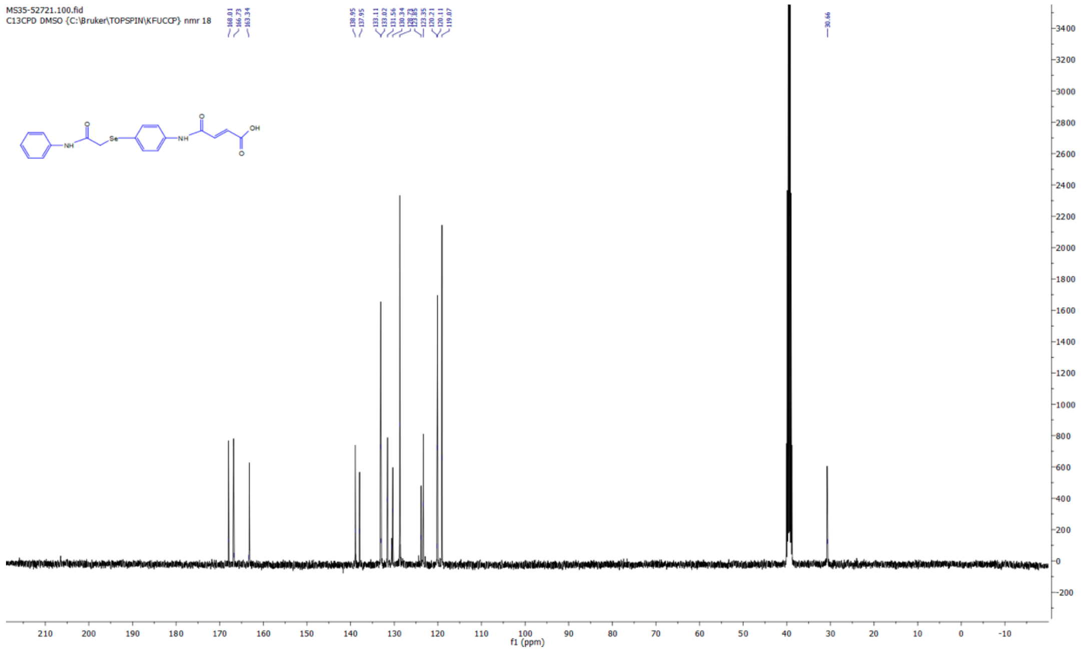

^{13}CNMR chart of compound 10.

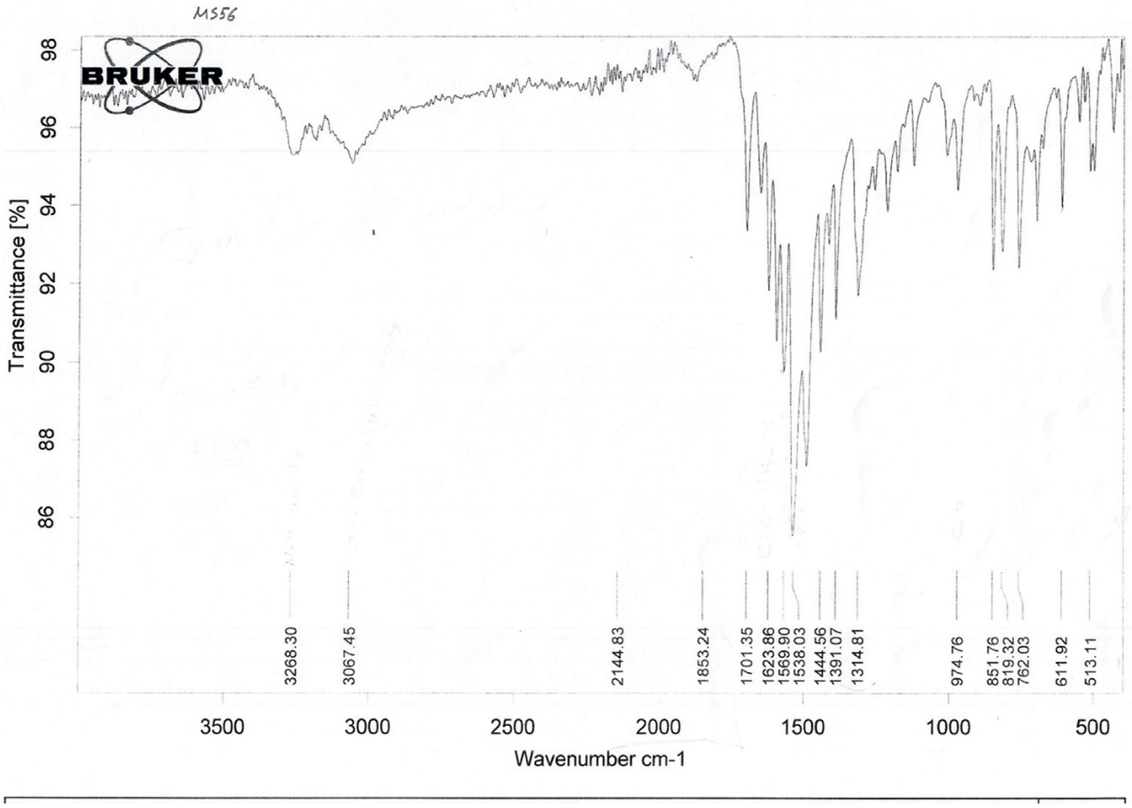

IR chart of compound 10.

03-Jan-07 19:39:43

Cairo University
Micro Analytical Center

DI Analysis
Shimadzu Qp-2010 Plus

Sample Information

Analyzed by	: Dr. Mai Younis
Analyzed	: 03/01/2007 07:26:42 ‎م
Sample Name	: M6
Sample ID	:
Customer Name	: Dr.Mohamed Soliman - Science - Cairo
Data File	: C:\GCMSsolution\Data\Project1\M6.QGD
Org Data File	: C:\GCMSsolution\Data\Project1\M6.QGD
Method File	: C:\GCMSsolution\Data\Project1\High Temperature Op
Org Method File	: C:\GCMSsolution\Data\Project1\High Temperature Op
Report File	:
Tuning File	: C:\GCMSsolution\System\Tune1_default.qgt
$EndIf$Modified by	: Dr. Mai Younis
Modified	: 03/01/2007 07:33:22 ‎م

Method

——— Analytical Line 1 ———

IonSourceTemp	:250.00 °C
[MS Table]	
--Group 1 - Event 1--	
Start Time	:0.00min
End Time	:10.00min
ACQ Mode	:Scan
Event Time	:0.50sec
Scan Speed	:1000
Start m/z	:50.00
End m/z	:510.00
Electron Voltage	: 70 eV
Ionization Mode	: EI

C:\GCMSsolution\Data\Project1\M6.QGD

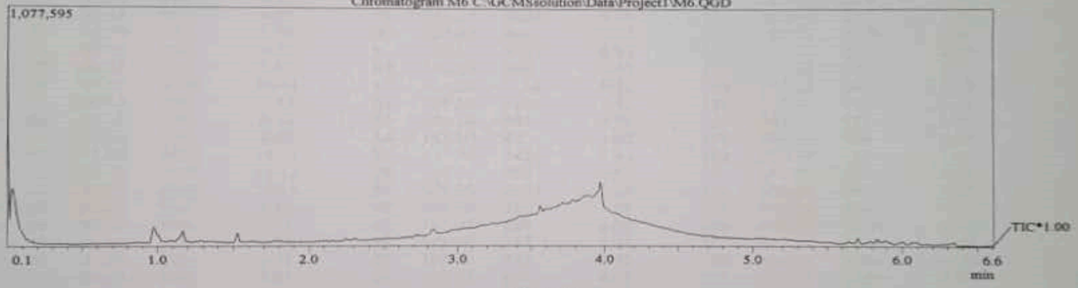

Spectrum

Line#:1 R.Time:3.7(Scan#:448)
MassPeaks:142(Peak Elimination m/z: 386.20)
RawMode:Single 3.7(448) BasePeak:59(19272)
BG Mode:None Group 1 - Event 1

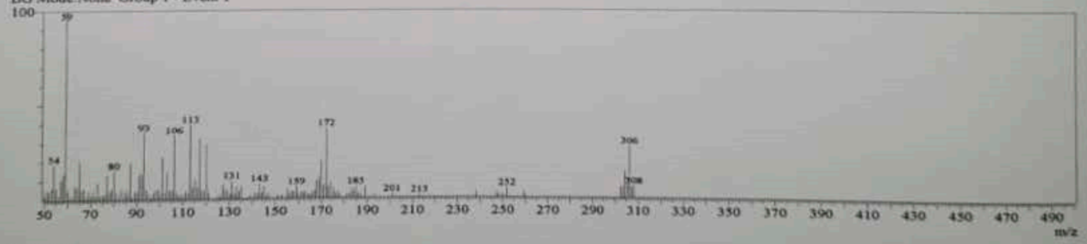

Mass Table
Line#:1 R.Time:3.7(Scan#:448)
MassPeaks:142(Peak Elimination m/z: 386.20)
RawMode:Single 3.7(448) BasePeak:59(19272)
BG Mode:None Group 1 - Event 1

#	m/z	Abs. In	Rel. Int.	#	m/z	Abs. In	Rel. Int.	#	m/z	Abs. In	Rel. Int.
1	49.95	449	2.33	4	53.05	1240	6.43	7	56.05	450	2.33
2	51.00	1002	5.20	5	54.00	3606	18.71	8	57.05	2062	10.70
3	51.95	825	4.28	6	55.00	1185	6.15	9	58.05	2621	13.60

1 / 2

Mass chart of compound 10.

03-Jan-07 19:39:43

#	m/z	Abs. In	Rel. Int.
10	59.05	19272	100.00
11	59.95	927	4.81
12	61.00	361	1.87
13	62.05	357	1.85
14	63.00	1372	7.12
15	64.05	1203	6.24
16	65.00	3887	20.17
17	66.05	991	5.14
18	67.10	1082	5.61
19	68.00	294	1.53
20	69.05	838	4.35
21	70.15	463	2.40
22	71.10	858	4.45
23	72.15	370	1.92
24	73.05	1708	8.86
25	74.10	401	2.08
26	75.15	540	2.80
27	76.15	466	2.42
28	77.05	2465	12.79
29	78.10	793	4.11
30	79.10	1054	5.47
31	80.10	2911	15.10
32	81.10	726	3.77
33	82.20	236	1.22
34	83.15	957	4.97
35	84.25	332	1.72
36	85.15	848	4.40
37	86.15	571	2.96
38	87.15	3741	19.41
39	88.15	296	1.54
40	89.15	779	4.04
41	90.15	774	4.02
42	91.10	2535	13.15
43	92.10	2567	13.32
44	93.10	6780	35.18
45	94.15	928	4.82
46	95.10	599	3.11
47	96.20	252	1.31
48	97.25	677	3.51
49	98.15	888	4.61
50	99.20	966	5.01
51	100.15	581	3.01
52	101.10	4239	22.00
53	102.15	628	3.26
54	103.15	2688	13.95

#	m/z	Abs. In	Rel. Int.
55	104.20	884	4.59
56	105.15	829	4.30
57	106.15	6490	33.68
58	107.20	695	3.61
59	108.20	346	1.80
60	109.15	460	2.39
61	110.20	234	1.21
62	111.15	761	3.95
63	112.15	555	2.88
64	113.15	7562	39.24
65	114.15	1097	5.69
66	115.20	2058	10.68
67	116.25	1016	5.27
68	117.20	6113	31.72
69	118.20	831	4.31
70	119.15	785	4.07
71	120.15	5573	28.92
72	121.10	623	3.23
73	125.10	372	1.93
74	126.25	350	1.82
75	127.15	1402	7.27
76	128.25	858	4.45
77	129.15	825	4.28
78	130.25	420	2.18
79	131.15	1803	9.36
80	132.15	464	2.41
81	133.15	1144	5.94
82	134.15	681	3.53
83	135.20	1252	6.50
84	139.20	310	1.61
85	141.10	521	2.70
86	142.10	473	2.45
87	143.10	1473	7.64
88	144.10	508	2.64
89	145.05	1184	6.14
90	146.10	351	1.82
91	147.05	388	2.01
92	151.00	247	1.28
93	153.00	273	1.42
94	155.05	847	4.39
95	156.10	367	1.90
96	157.05	674	3.50
97	157.95	563	2.92
98	159.10	1130	5.86
99	159.90	484	2.51

#	m/z	Abs. In	Rel. Int.
100	161.15	640	3.32
101	162.05	497	2.58
102	162.90	596	3.09
103	164.20	385	2.00
104	164.90	257	1.33
105	165.90	545	2.83
106	167.05	734	3.81
107	168.00	1727	8.96
108	169.00	2081	10.80
109	170.00	3783	19.63
110	171.05	1261	6.54
111	172.05	7125	36.97
112	172.95	1082	5.61
113	173.95	1640	8.51
114	175.15	974	5.05
115	176.00	362	1.88
116	177.00	548	2.84
117	178.00	442	2.29
118	181.00	273	1.42
119	182.00	369	1.91
120	183.05	910	4.72
121	184.05	665	3.45
122	185.00	1114	5.78
123	185.95	367	1.90
124	186.90	343	1.78
125	189.20	1110	5.76
126	193.20	252	1.31
127	201.10	327	1.70
128	213.10	247	1.28
129	238.10	490	2.54
130	247.15	571	2.96
131	248.10	306	1.59
132	250.10	282	1.46
133	251.80	929	4.82
134	259.20	596	3.09
135	260.20	206	1.07
136	302.20	1070	5.55
137	303.25	1140	5.92
138	304.20	2822	14.64
139	305.25	1229	6.38
140	306.20	5262	27.30
141	307.15	1037	5.38
142	308.20	1170	6.07

Mass chart of compound 10.

4. -Oxo-4-((4-((2-oxo-2-(phenylamino)ethyl)selanyl)phenyl)amino)butanoic acid (11)

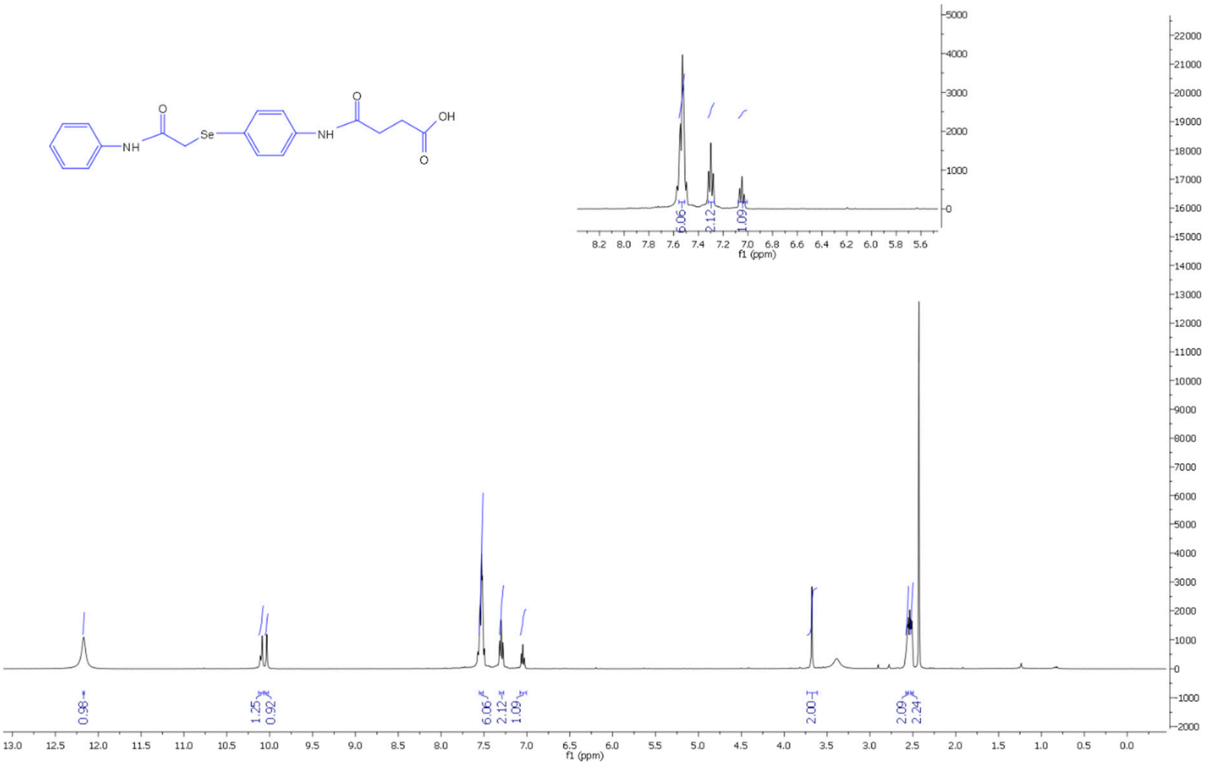

^{1}HNMR chart of compound 11.

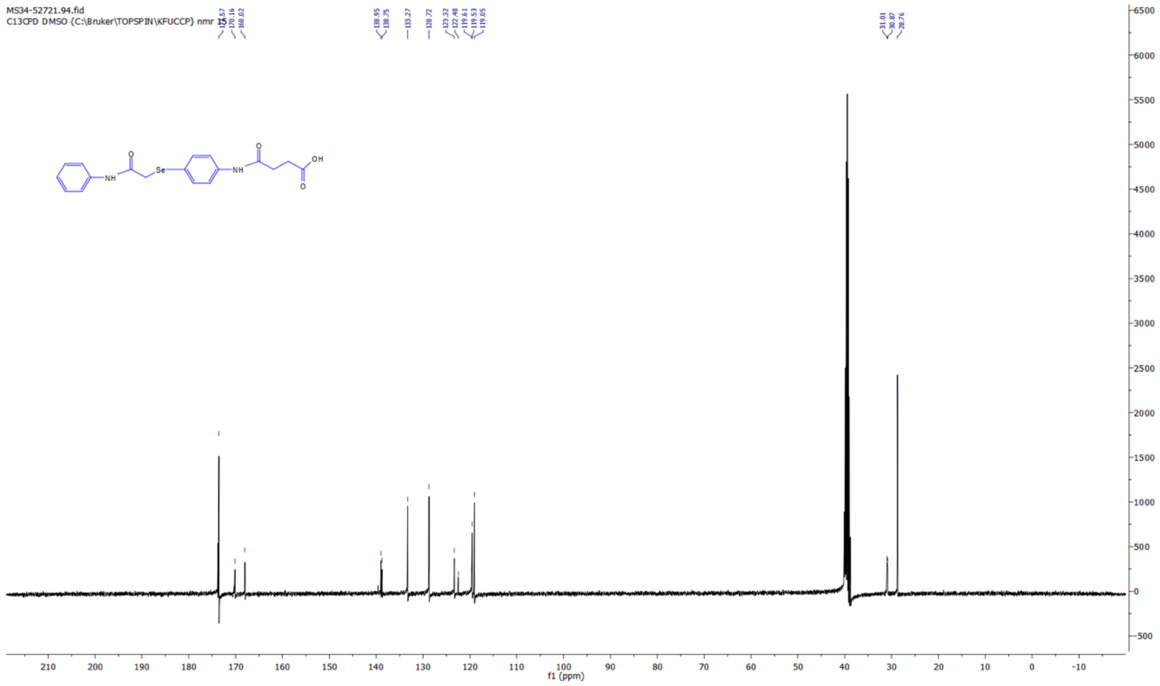

^{13}CNMR chart of compound 11.

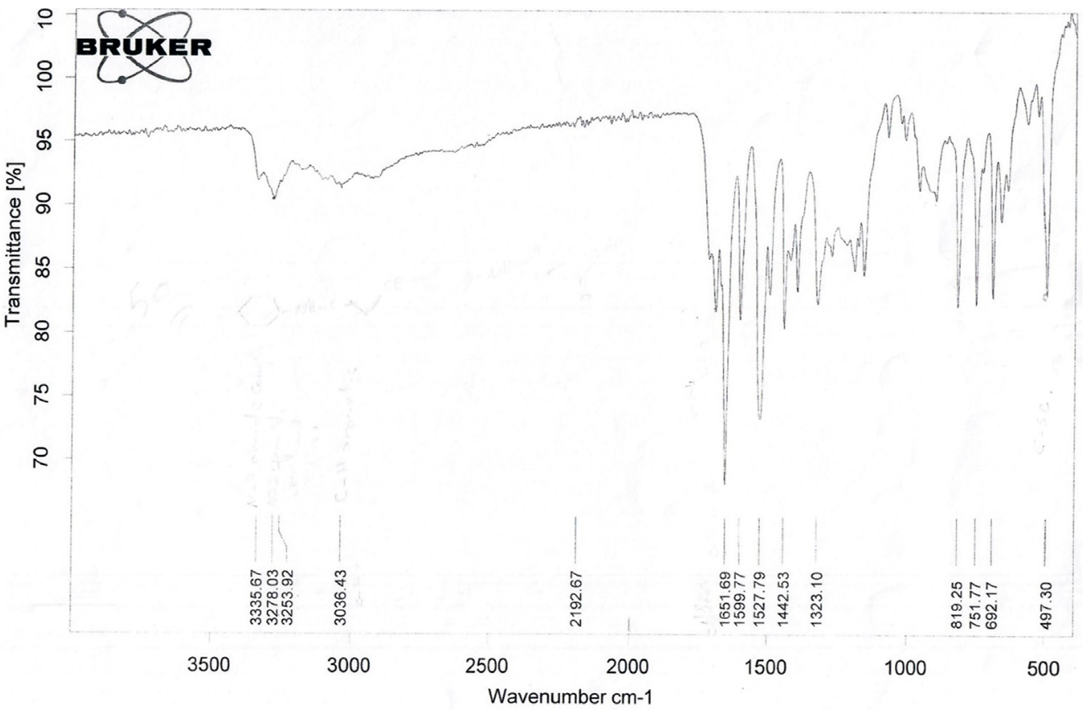

IR chart of compound 11.

Cairo University
Micro Analytical Center

DI Analysis
Shimadzu Qp-2010 Plus

Sample Information		Method	
Analyzed by	: Dr. Mai Younis	—— Analytical Line 1 ——	
Analyzed	: 09/01/2007 06:23:32 م	IonSourceTemp	:250.00 °C
Sample Name	: M5	[MS Table]	
Sample ID	:	--Group 1 - Event 1--	
Customer Name	: Dr.Mohamed Soliman - Science - Cairo	Start Time	:0.00min
Data File	: C:\GCMSsolution\Data\Project1\M5.QGD	End Time	:10.00min
Org Data File	: C:\GCMSsolution\Data\Project1\M5.QGD	ACQ Mode	:Scan
Method File	: C:\GCMSsolution\Data\Project1\High Temperature Op	Event Time	:0.50sec
Org Method File	: C:\GCMSsolution\Data\Project1\High Temperature Op	Scan Speed	:1000
Report File	:	Start m/z	:50.00
Tuning File	: C:\GCMSsolution\System\Tune1_default.qgt	End m/z	:510.00
$EndIf$Modified by	: Dr. Mai Younis		
Modified	: 09/01/2007 06:27:51 م		
		Electron Voltage	: 70 eV
		Ionization Mode	: EI

C:\GCMSsolution\Data\Project1\M5.QGD

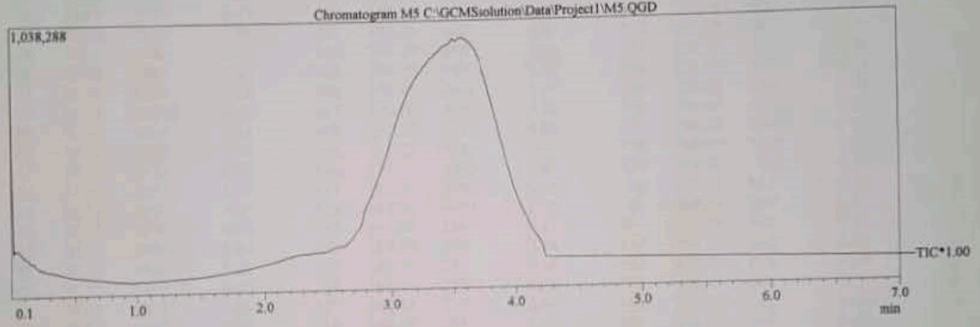

Spectrum

Line#:1 R.Time:3.5(Scan#:420)
MassPeaks:233
RawMode:Single 3.5(420) BasePeak:59(200624)
BG Mode:None Group 1 - Event 1

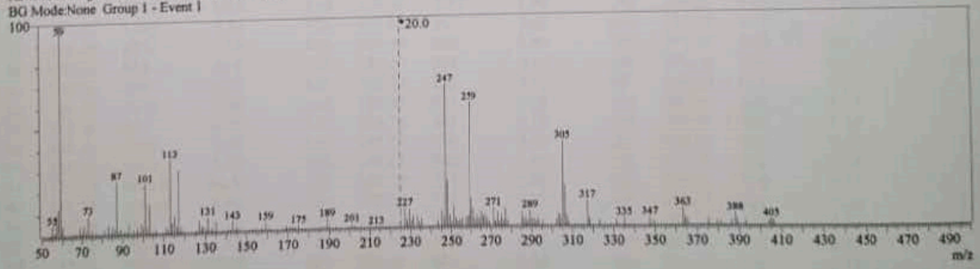

Mass Table
Line#:1 R.Time:3.5(Scan#:420)
MassPeaks:233
RawMode:Single 3.5(420) BasePeak:59(200624)
BG Mode:None Group 1 - Event 1

#	m/z	Abs. In	Rel. Int.	#	m/z	Abs. In	Rel. Int.	#	m/z	Abs. In	Rel. Int.
1	50.05	469	0.23	4	53.05	812	0.40	7	56.15	3322	1.66
2	51.05	749	0.37	5	54.05	1018	0.51	8	57.05	19341	9.64
[illegible]	52.00	494	0.25	6	55.05	9864	4.92	9	58.15	25060	12.49

Mass chart of compound 11.

#	m/z	Abs. In	Rel. Int.
10	59.05	200624	100.00
11	60.05	8758	4.37
12	61.05	1986	0.99
13	62.10	359	0.18
14	63.10	1100	0.55
15	64.05	921	0.46
16	65.05	2511	1.25
17	66.10	910	0.45
18	67.10	2422	1.21
19	68.15	1311	0.65
20	69.10	8111	4.04
21	70.10	3582	1.79
22	71.10	9319	4.65
23	72.15	3512	1.75
24	73.10	18257	9.10
25	74.10	2212	1.10
26	75.05	4454	2.22
27	76.15	725	0.36
28	77.10	3671	1.83
29	78.05	848	0.42
30	79.10	1372	0.68
31	80.15	1881	0.94
32	81.05	5370	2.68
33	82.15	1541	0.77
34	83.10	9586	4.78
35	84.15	2440	1.22
36	85.10	8862	4.42
37	86.15	4769	2.38
38	87.10	50266	25.05
39	88.05	3026	1.51
40	89.10	5838	2.91
41	90.10	1134	0.57
42	91.10	3532	1.76
43	92.15	2339	1.17
44	93.10	9934	4.95
45	94.15	1686	0.84
46	95.10	4430	2.21
47	96.15	1374	0.68
48	97.10	6033	3.01
49	98.15	1987	0.99
50	99.15	10203	5.09
51	100.15	6077	3.03
52	101.10	46953	23.40
53	102.15	5364	2.67
54	103.10	27556	13.74
55	104.15	2268	1.13
56	105.15	1405	0.70
57	106.10	4273	2.13
58	107.10	914	0.46
59	108.10	439	0.22
60	109.15	1330	0.66
61	110.15	851	0.42
62	111.15	6392	3.19
63	112.15	3503	1.75
64	113.15	69018	34.40
65	114.15	10012	4.99
66	115.15	16417	8.18
67	116.25	6882	3.43
68	117.15	59348	29.58
69	118.15	5363	2.67
70	119.15	2485	1.24
71	120.15	1464	0.73
72	121.25	506	0.25
73	122.30	268	0.13
74	123.30	639	0.32
75	124.25	357	0.18
76	125.20	1369	0.68
77	126.25	1935	0.96
78	127.20	10630	5.30
79	128.20	5354	2.67
80	129.20	5953	2.97
81	130.25	2673	1.33
82	131.20	14449	7.20
83	132.20	2164	1.08
84	133.20	4103	2.05
85	134.25	1003	0.50
86	135.20	9860	4.91
87	136.20	762	0.38
88	137.20	679	0.34
89	138.25	330	0.16
90	139.15	1550	0.77
91	140.25	682	0.34
92	141.15	1951	0.97
93	142.25	1233	0.61
94	143.15	9602	4.79
95	144.20	1748	0.87
96	145.15	3927	1.96
97	146.20	1193	0.59
98	147.20	1556	0.78
99	148.20	206	0.10
100	149.20	239	0.12
101	150.20	209	0.10
102	151.15	1348	0.67
103	152.25	453	0.23
104	153.20	919	0.46
105	154.15	589	0.29
106	155.15	2339	1.17
107	156.20	554	0.28
108	157.15	1711	0.85
109	158.25	773	0.39
110	159.20	8001	3.99
111	160.20	1095	0.55
112	161.20	1553	0.77
113	162.20	258	0.13
114	163.20	282	0.14
115	165.20	242	0.12
116	167.15	437	0.22
117	168.15	795	0.40
118	169.15	3351	1.67
119	170.10	1852	0.92
120	171.15	2069	1.03
121	172.10	2833	1.41
122	173.20	2655	1.32
123	174.15	860	0.43
124	175.20	4017	2.00
125	176.15	947	0.47
126	177.20	988	0.49
127	181.20	258	0.13
128	182.20	225	0.11
129	183.10	631	0.31
130	184.20	361	0.18
131	185.25	904	0.45
132	186.15	940	0.47
133	187.20	1377	0.69
134	188.25	731	0.36
135	189.20	9526	4.75
136	190.15	1552	0.77
137	191.15	685	0.34
138	193.20	1868	0.93
139	194.20	311	0.16
140	195.20	230	0.11
141	196.20	220	0.11
142	197.20	228	0.11
143	199.20	354	0.18
144	200.25	421	0.21
145	201.20	3977	1.98
146	202.15	917	0.46
147	203.15	1285	0.64
148	204.10	359	0.18
149	205.10	295	0.15
150	211.10	212	0.11
151	213.20	986	0.49
152	214.20	214	0.11
153	215.20	482	0.24
154	217.15	702	0.35
155	219.15	498	0.25
156	227.25	878	0.44
157	228.35	342	0.17
158	229.30	834	0.42
159	230.35	316	0.16
160	231.30	540	0.27
161	233.35	570	0.28
162	234.40	230	0.11
163	235.40	386	0.19
164	243.40	274	0.14
165	245.25	755	0.38
166	246.35	427	0.21
167	247.30	6767	3.37
168	248.25	2136	1.06
169	249.30	537	0.27
170	250.30	214	0.11
171	251.35	921	0.46
172	252.25	387	0.19
173	253.30	233	0.12
174	254.30	297	0.15
175	255.30	370	0.18
176	257.40	434	0.22
177	258.35	484	0.24
178	259.30	5902	2.94
179	260.20	1292	0.64
180	261.25	811	0.40
181	262.30	329	0.16
182	263.30	503	0.25
183	264.35	322	0.16
184	265.30	676	0.34
185	266.25	505	0.25
186	267.20	532	0.27
187	268.20	335	0.17
188	269.20	215	0.11
189	271.30	846	0.42
190	272.30	241	0.12
191	273.30	663	0.33
192	274.30	228	0.11
193	275.25	519	0.26
194	276.30	257	0.13
195	277.30	820	0.41
196	278.30	250	0.12
197	285.30	633	0.32
198	286.30	306	0.15
199	287.30	382	0.19
200	288.30	220	0.11
201	289.30	726	0.36
202	290.30	305	0.15
203	291.30	311	0.16
204	292.30	226	0.11
205	293.30	302	0.15
206	295.30	241	0.12
207	302.30	284	0.14
208	303.30	574	0.29
209	304.25	442	0.22
210	305.35	3968	1.98
211	306.20	1903	0.95
212	307.15	500	0.25
213	308.20	220	0.11
214	317.30	1186	0.59
215	318.25	352	0.18
216	323.30	273	0.14

Mass chart of compound 11.

09-Jan-07 18:29:40

#	m/z	Abs. In	Rel. Int.	#	m/z	Abs. In	Rel. Int.	#	m/z	Abs. In	Rel. Int.
217	331.30	249	0.12	223	365.50	273	0.14	229	389.20	255	0.13
218	335.15	363	0.18	224	375.50	303	0.15	230	393.20	202	0.10
219	347.45	383	0.19	225	379.50	230	0.11	231	404.20	215	0.11
220	349.50	206	0.10	226	381.50	212	0.11	232	405.20	297	0.15
221	363.40	804	0.40	227	386.50	266	0.13	233	406.20	274	0.14
222	364.45	380	0.19	228	388.20	564	0.28				

Mass chart of compound 11.

2. -((4-(1,3-Dioxoisoindolin-2-yl)phenyl)selanyl)-N-phenylacetamide (12)

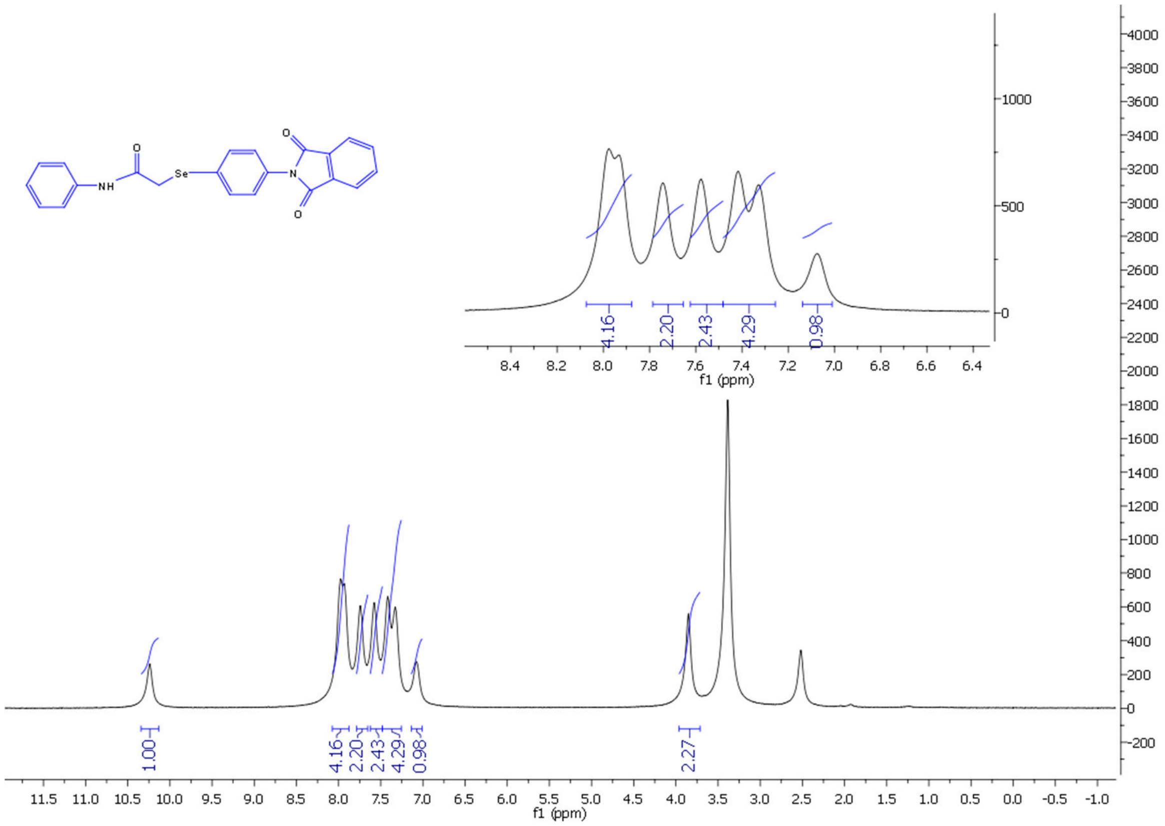

^{1}HNMR chart of compound 12.

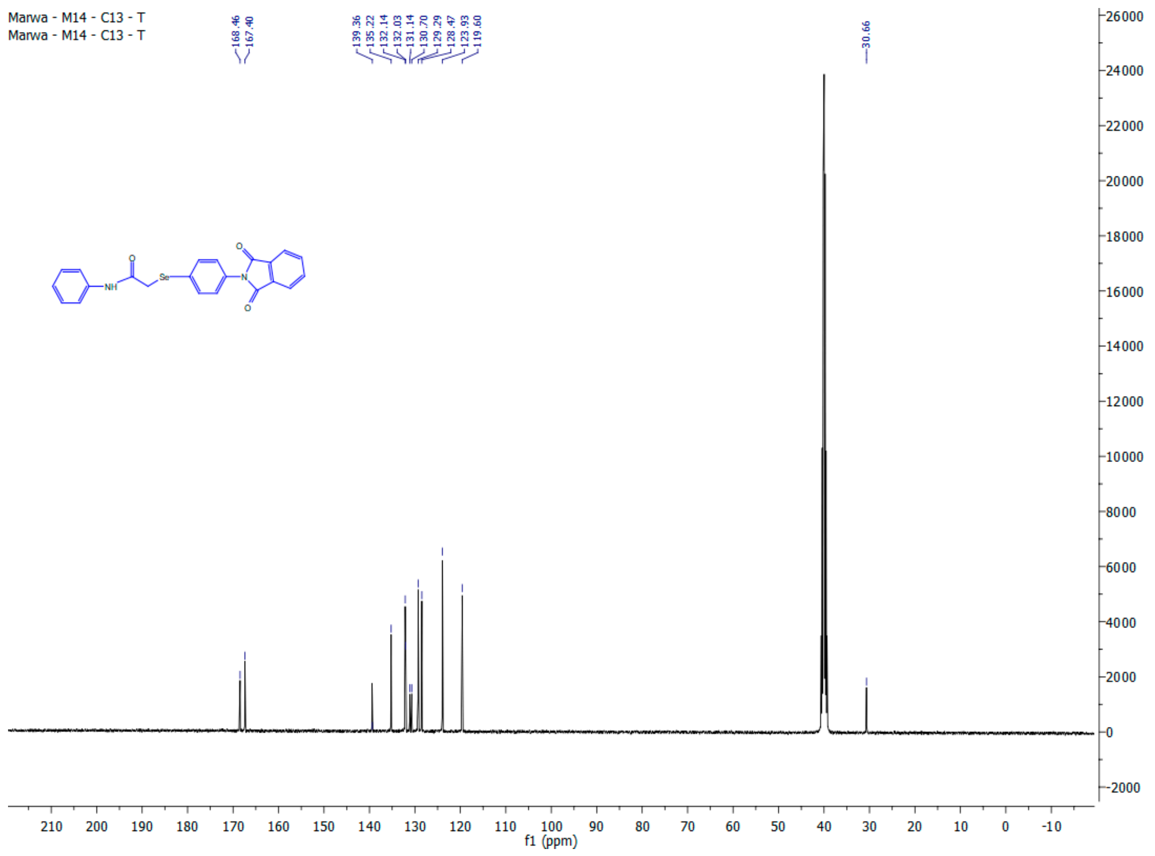

^{13}CNMR chart of compound 12.

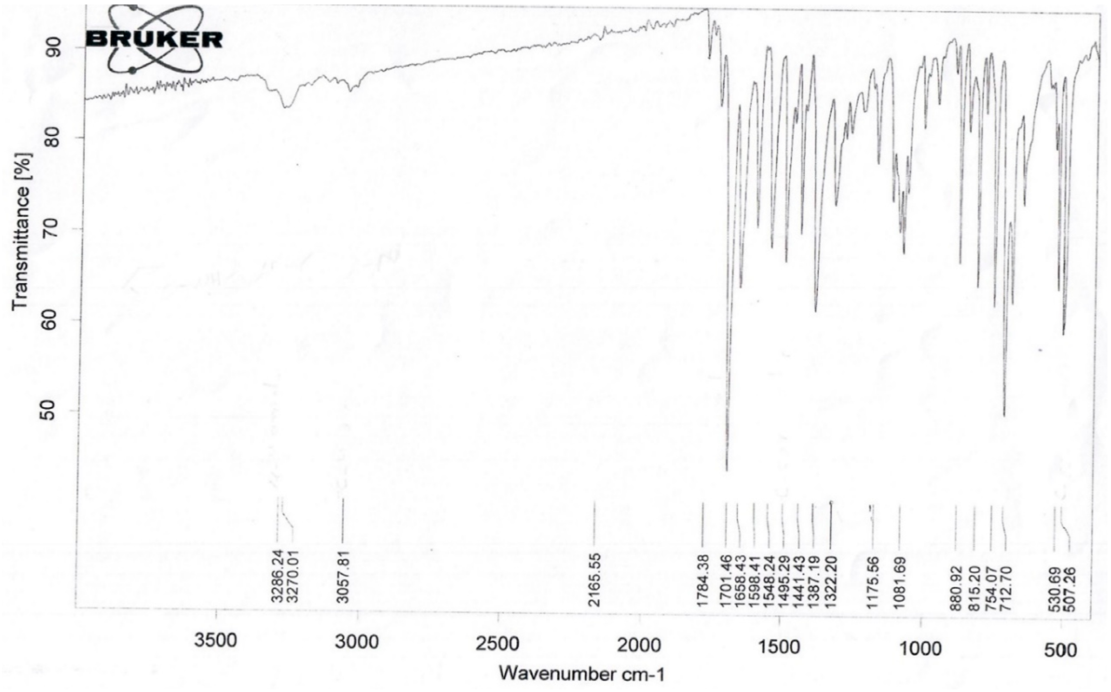

IR chart of compound 12.

09-Jan-07 19:12:07

Cairo University
Micro Analytical Center

DI Analysis
Shimadzu Qp-2010 Plus

Sample Information

Analyzed by	: Dr. Mai Younis
Analyzed	: 09/01/2007 07:02:42
Sample Name	: M14
Sample ID	:
Customer Name	: Dr.Mohamed Soliman - Science - Cairo
Data File	: C:\GCMSsolution\Data\Project1\M14.QGD
Org Data File	: C:\GCMSsolution\Data\Project1\M14.QGD
Method File	: C:\GCMSsolution\Data\Project1\High Temperature Op
Org Method File	: C:\GCMSsolution\Data\Project1\High Temperature Op
Report File	:
Tuning File	: C:\GCMSsolution\System\Tune1_default.qgt
$EndIf$Modified by	: Dr. Mai Younis
Modified	: 09/01/2007 07:10:38

Method

——— Analytical Line 1 ———

IonSourceTemp	:250.00 °C
[MS Table]	
--Group 1 - Event 1--	
Start Time	:0.00min
End Time	:10.00min
ACQ Mode	:Scan
Event Time	:0.50sec
Scan Speed	:1000
Start m/z	:50.00
End m/z	:510.00
Electron Voltage	: 70 eV
Ionization Mode	: EI

C:\GCMSsolution\Data\Project1\M14.QGD

Chromatogram M14 C:\GCMSsolution\Data\Project1\M14.QGD

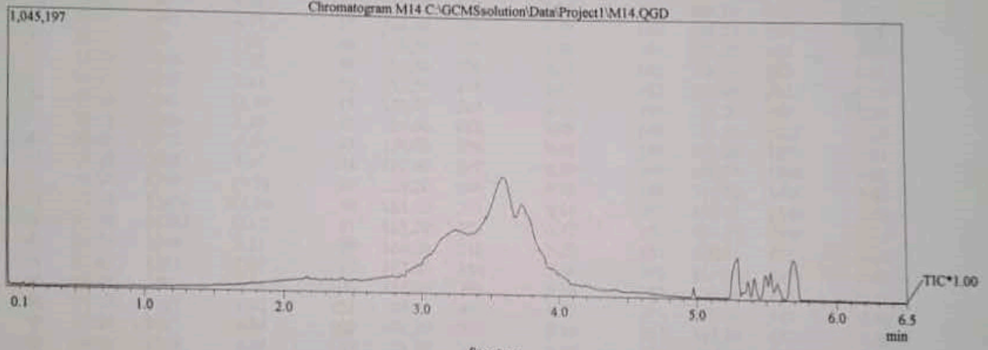

Spectrum

Line#:1 R.Time:3.6(Scan#:433)
MassPeaks:167
RawMode:Single 3.6(433) BasePeak:59(62218)
BG Mode:None Group 1 - Event 1

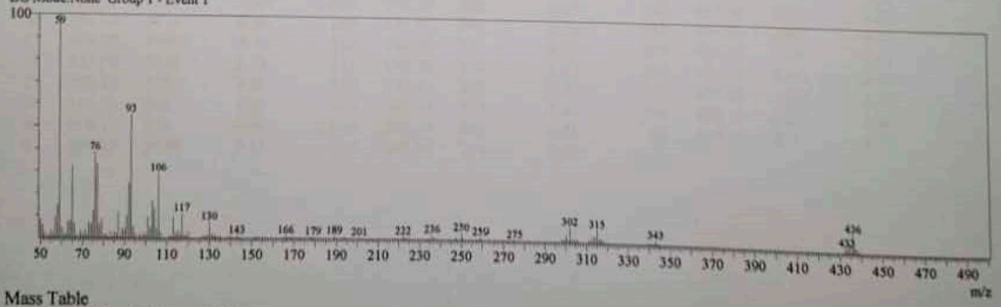

Mass Table
Line#:1 R.Time:3.6(Scan#:433)
MassPeaks:167
RawMode:Single 3.6(433) BasePeak:59(62218)
BG Mode:None Group 1 - Event 1

#	m/z	Abs. In	Rel. Int.	#	m/z	Abs. In	Rel. Int.	#	m/z	Abs. In	Rel. Int.
1	50.00	5129	8.24	4	53.00	546	0.88	7	56.05	992	1.59
2	51.00	3476	5.59	5	54.05	544	0.87	8	57.00	5849	9.40
3	52.00	1367	2.20	6	55.00	2030	3.26	9	58.05	9138	14.69

1 / 2

Mass chart of compound 12.

09-Jan-07 19:12:07

#	m/z	Abs. In	Rel. Int.	#	m/z	Abs. In	Rel. Int.	#	m/z	Abs. In	Rel. Int.
10	59.00	62218	100.00	63	114.15	1184	1.90	116	201.20	362	0.58
11	60.00	2788	4.48	64	115.15	2168	3.48	117	217.20	238	0.38
12	61.05	743	1.19	65	116.25	1183	1.90	118	218.20	300	0.48
13	62.05	1175	1.89	66	117.15	6904	11.10	119	222.15	787	1.26
14	63.00	4690	7.54	67	118.15	965	1.55	120	223.10	292	0.47
15	64.05	4854	7.80	68	119.10	1142	1.84	121	224.10	516	0.83
16	65.00	20110	32.32	69	120.15	2119	3.41	122	235.20	241	0.39
17	66.00	3959	6.36	70	121.10	201	0.32	123	236.15	1313	2.11
18	67.00	1012	1.63	71	126.20	282	0.45	124	237.15	323	0.52
19	68.15	451	0.72	72	127.15	795	1.28	125	238.20	228	0.37
20	69.05	1924	3.09	73	128.15	637	1.02	126	247.30	631	1.01
21	70.05	933	1.50	74	129.15	764	1.23	127	248.30	225	0.36
22	71.05	2197	3.53	75	130.15	4535	7.29	128	250.20	1961	3.15
23	72.05	1086	1.75	76	131.20	1324	2.13	129	251.15	448	0.72
24	73.05	4423	7.11	77	132.15	1056	1.70	130	257.20	231	0.37
25	74.05	3849	6.19	78	133.15	697	1.12	131	258.25	327	0.53
26	75.05	7578	12.18	79	134.10	497	0.80	132	259.35	823	1.32
27	76.00	23719	38.12	80	135.10	599	0.96	133	275.30	262	0.42
28	77.00	20535	33.00	81	138.30	212	0.34	134	298.20	689	1.11
29	78.05	3876	6.23	82	139.30	537	0.86	135	299.25	966	1.55
30	79.05	5345	8.59	83	140.30	438	0.70	136	300.20	2108	3.39
31	80.00	1150	1.85	84	141.25	302	0.49	137	301.15	619	0.99
32	81.05	1262	2.03	85	142.20	204	0.33	138	302.15	4196	6.74
33	82.05	443	0.71	86	143.15	707	1.14	139	303.10	842	1.35
34	83.05	1947	3.13	87	144.20	233	0.37	140	304.25	859	1.38
35	84.15	635	1.02	88	145.20	289	0.46	141	305.30	908	1.46
36	85.10	1838	2.95	89	151.20	426	0.68	142	306.30	382	0.61
37	86.15	1332	2.14	90	152.30	418	0.67	143	310.20	212	0.34
38	87.05	7343	11.80	91	153.25	369	0.59	144	311.20	782	1.26
39	88.15	1086	1.75	92	155.00	622	1.00	145	312.20	1023	1.64
40	89.05	2727	4.38	93	156.00	250	0.40	146	313.20	1870	3.01
41	90.15	2468	3.97	94	157.00	242	0.39	147	314.25	1906	3.06
42	91.10	6399	10.28	95	159.20	546	0.88	148	315.20	3626	5.83
43	92.15	15430	24.80	96	164.20	273	0.44	149	316.20	1262	2.03
44	93.10	34252	55.05	97	165.20	233	0.37	150	317.20	1198	1.93
45	94.05	3244	5.21	98	166.20	748	1.20	151	318.10	423	0.68
46	95.05	1165	1.87	99	167.20	350	0.56	152	319.10	287	0.46
47	96.15	349	0.56	100	168.20	359	0.58	153	339.10	215	0.35
48	97.10	1185	1.90	101	169.20	457	0.73	154	340.10	303	0.49
49	98.15	408	0.66	102	170.20	310	0.50	155	341.20	657	1.06
50	99.10	1518	2.44	103	171.20	254	0.41	156	342.20	226	0.36
51	100.15	1049	1.69	104	172.20	263	0.42	157	343.20	1252	2.01
52	101.10	6006	9.65	105	173.20	222	0.36	158	344.20	298	0.48
53	102.20	2993	4.81	106	175.20	359	0.58	159	345.20	345	0.55
54	103.15	10461	16.81	107	177.25	411	0.66	160	432.35	1134	1.82
55	104.15	8355	13.43	108	178.30	394	0.63	161	433.30	1231	1.98
56	105.15	2712	4.36	109	179.15	492	0.79	162	434.30	2681	4.31
57	106.10	17996	28.92	110	180.20	231	0.37	163	435.35	1002	1.61
58	107.10	1737	2.79	111	189.20	753	1.21	164	436.30	5252	8.44
59	108.10	239	0.38	112	190.20	220	0.35	165	437.25	1483	2.38
60	111.10	863	1.39	113	193.20	377	0.61	166	438.25	1121	1.80
61	112.15	574	0.92	114	194.20	361	0.58	167	439.30	254	0.41
62	113.15	5878	9.45	115	195.20	214	0.34				

Mass chart of compound 12.

2. -((4-(2,5-Dioxo-2,5-dihydro-1H-pyrrol-1-yl)phenyl)selanyl)-N-phenylacetamide (13)

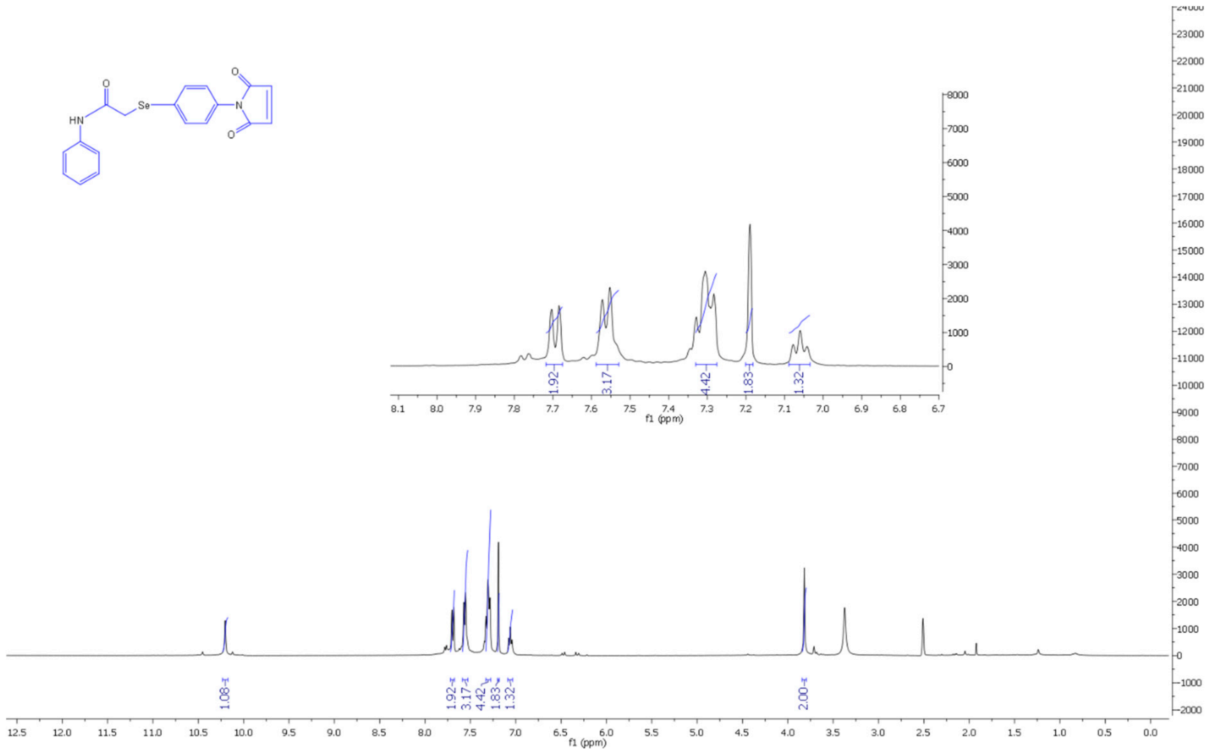

^{1}HNMR chart of compound 13.

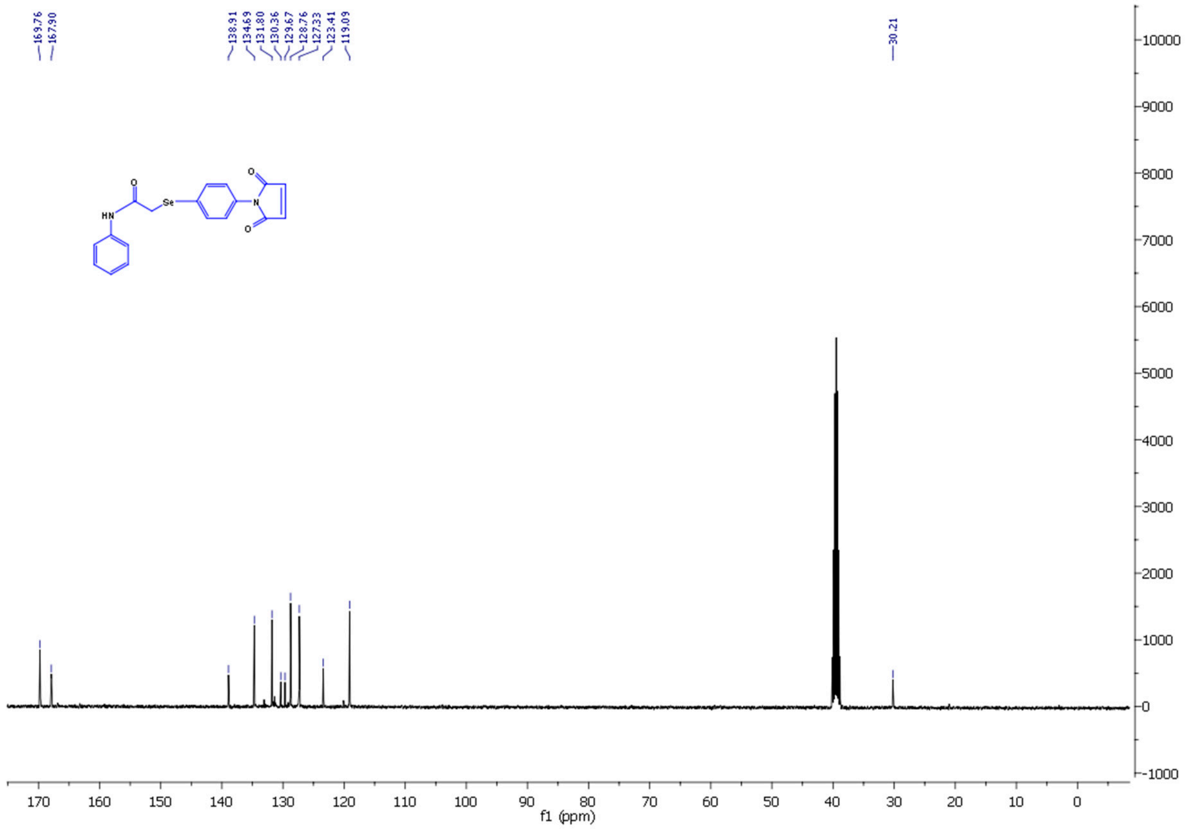

^{13}CNMR chart of compound 13.

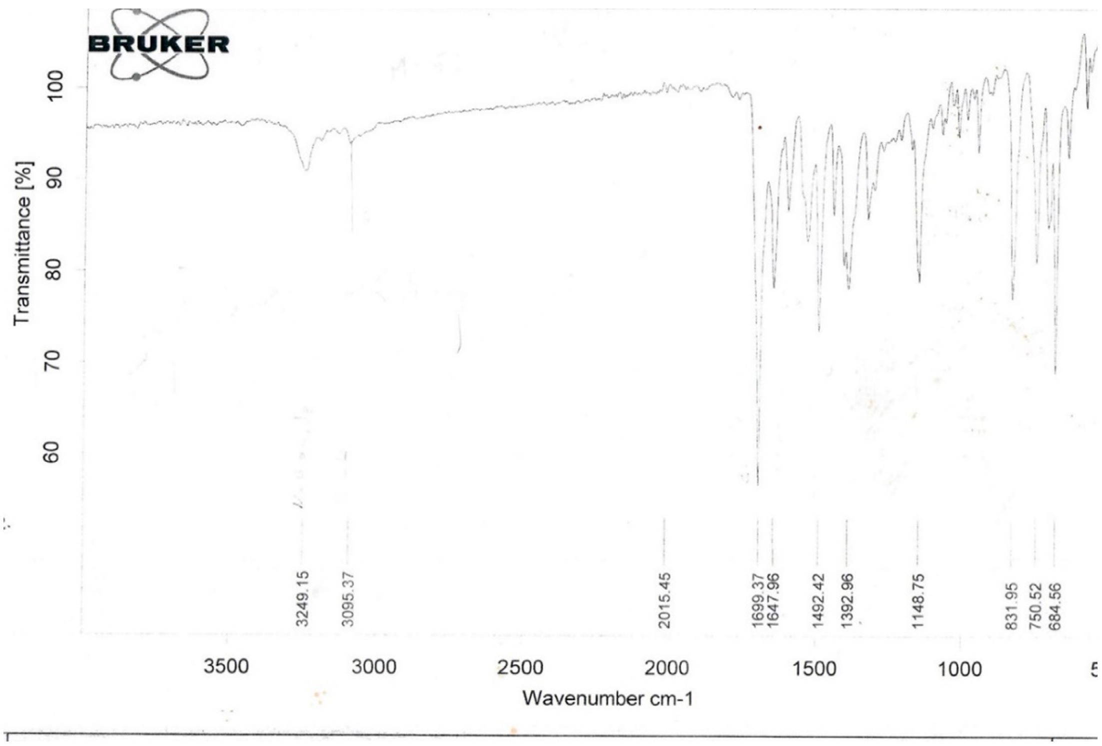

IR chart of compound 13.

03-Jan-07 19:44:03

Cairo University
Micro Analytical Center

DI Analysis
Shimadzu Qp-2010 Plus

Sample Information

Analyzed by	: Dr. Mai Younis
Analyzed	: 03/01/2007 07:37:16 م
Sample Name	: M7
Sample ID	:
Customer Name	: Dr.Mohamed Soliman - Science - Cairo
Data File	: C:\GCMSsolution\Data\Project1\M7.QGD
Org Data File	: C:\GCMSsolution\Data\Project1\M7.QGD
Method File	: C:\GCMSsolution\Data\Project1\High Temperature Op
Org Method File	: C:\GCMSsolution\Data\Project1\High Temperature Op
Report File	:
Tuning File	: C:\GCMSsolution\System\Tune1_default.qgt
$EndIf$Modified by	: Dr. Mai Younis
Modified	: 03/01/2007 07:42:22 م

Method

===== Analytical Line 1 =====

IonSourceTemp	:250.00 °C
[MS Table]	
--Group 1 - Event 1--	
Start Time	:0.00min
End Time	:10.00min
ACQ Mode	:Scan
Event Time	:0.50sec
Scan Speed	:1000
Start m/z	:50.00
End m/z	:510.00
Electron Voltage	: 70 eV
Ionization Mode	: EI

C:\GCMSsolution\Data\Project1\M7.QGD

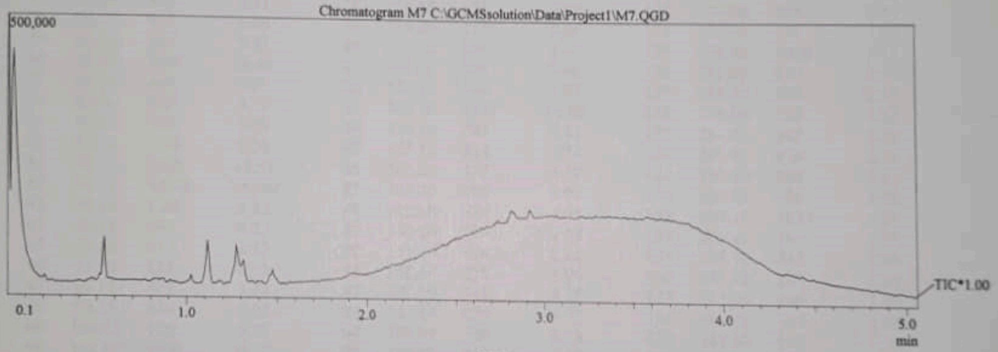

Spectrum

Line#:1 R.Time:2.8(Scan#:334)
MassPeaks:144
RawMode:Single 2.8(334) BasePeak:93(14348)
BG Mode:None Group 1 - Event 1

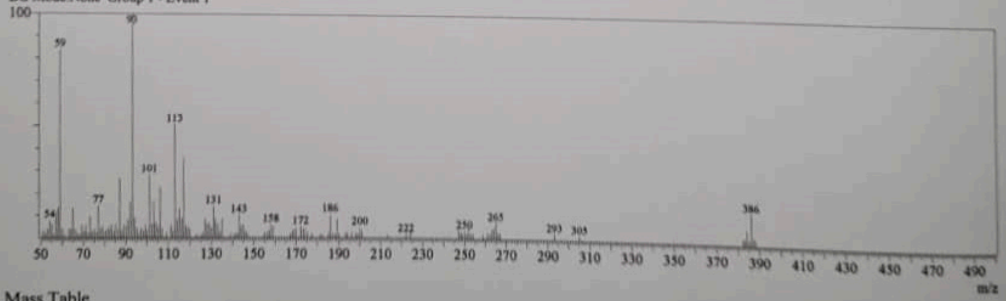

Mass Table
Line#:1 R.Time:2.8(Scan#:334)
MassPeaks:144
RawMode:Single 2.8(334) BasePeak:93(14348)
BG Mode:None Group 1 - Event 1

#	m/z	Abs. In	Rel. Int.	#	m/z	Abs. In	Rel. Int.	#	m/z	Abs. In	Rel. Int.
1	51.00	484	3.37	4	54.05	1131	7.88	7	57.05	1687	11.76
2	52.00	281	1.96	5	55.05	909	6.34	8	58.05	1929	13.44
3	53.05	559	3.90	6	56.00	223	1.55	9	59.00	12022	83.79

1 / 2

Mass chart of compound 13.

03-Jan-07 19:44:03

#	m/z	Abs. In	Rel. Int.	#	m/z	Abs. In	Rel. Int.	#	m/z	Abs. In	Rel. Int.
10	60.05	598	4.17	55	106.15	3243	22.60	100	175.30	382	2.66
11	61.00	215	1.50	56	107.10	558	3.89	101	177.30	233	1.62
12	63.10	586	4.08	57	109.10	322	2.24	102	181.30	214	1.49
13	64.15	584	4.07	58	111.10	782	5.45	103	182.30	220	1.53
14	65.05	1930	13.45	59	112.15	427	2.98	104	185.15	316	2.20
15	66.10	542	3.78	60	113.15	7270	50.67	105	186.10	1516	10.57
16	67.10	308	2.15	61	114.10	1106	7.71	106	187.10	308	2.15
17	68.10	210	1.46	62	115.15	1873	13.05	107	188.20	241	1.68
18	69.15	805	5.61	63	116.20	1218	8.49	108	189.20	1180	8.22
19	70.15	396	2.76	64	117.20	5150	35.89	109	190.20	255	1.78
20	71.10	798	5.56	65	118.15	793	5.53	110	193.20	324	2.26
21	72.15	317	2.21	66	119.10	682	4.75	111	194.20	282	1.97
22	73.10	1369	9.54	67	120.10	545	3.80	112	196.20	370	2.58
23	74.10	372	2.59	68	123.10	210	1.46	113	198.20	308	2.15
24	75.15	587	4.09	69	125.10	202	1.41	114	199.10	220	1.53
25	76.15	338	2.36	70	126.15	294	2.05	115	200.15	687	4.79
26	77.10	2065	14.39	71	127.15	1192	8.31	116	201.10	462	3.22
27	78.15	596	4.15	72	128.10	889	6.20	117	213.10	215	1.50
28	79.10	742	5.17	73	129.10	946	6.59	118	222.10	254	1.77
29	80.20	410	2.86	74	130.15	623	4.34	119	224.10	428	2.98
30	81.15	616	4.29	75	131.20	2028	14.13	120	247.20	494	3.44
31	82.15	556	3.88	76	132.10	1172	8.17	121	248.15	374	2.61
32	83.10	807	5.62	77	133.10	882	6.15	122	249.00	302	2.10
33	84.20	418	2.91	78	134.15	378	2.63	123	250.00	498	3.47
34	85.15	927	6.46	79	135.20	1154	8.04	124	251.00	271	1.89
35	86.15	461	3.21	80	139.20	266	1.85	125	252.00	1020	7.11
36	87.10	3794	26.44	81	141.20	295	2.06	126	253.00	250	1.74
37	88.15	440	3.07	82	142.10	268	1.87	127	254.00	308	2.15
38	89.10	828	5.77	83	143.10	1447	10.09	128	259.00	218	1.52
39	90.15	730	5.09	84	144.10	785	5.47	129	261.00	342	2.38
40	91.10	1148	8.00	85	145.15	824	5.74	130	262.00	310	2.16
41	92.15	2228	15.53	86	146.20	457	3.19	131	263.10	564	3.93
42	93.15	14348	100.00	87	147.20	289	2.01	132	264.15	686	4.78
43	94.10	1265	8.82	88	155.00	362	2.52	133	265.15	1033	7.20
44	95.15	648	4.52	89	156.20	359	2.50	134	266.10	391	2.73
45	96.10	311	2.17	90	157.10	404	2.82	135	267.10	342	2.38
46	97.15	625	4.36	91	158.10	855	5.96	136	293.15	386	2.69
47	98.15	471	3.28	92	159.10	742	5.17	137	305.20	286	1.99
48	99.15	864	6.02	93	167.10	225	1.57	138	382.25	380	2.65
49	100.15	506	3.53	94	167.95	399	2.78	139	383.20	482	3.36
50	101.15	4021	28.02	95	168.95	555	3.87	140	384.20	977	6.81
51	102.20	858	5.98	96	169.90	612	4.27	141	385.30	334	2.33
52	103.15	2384	16.62	97	172.05	723	5.04	142	386.25	2044	14.25
53	104.10	961	6.70	98	173.15	596	4.15	143	387.25	513	3.58
54	105.20	710	4.95	99	174.15	597	4.16	144	388.20	410	2.86

Mass chart of compound 13.

2. -((4-(2,5-Dioxopyrrolidin-1-yl)phenyl)selanyl)-N-phenylacetamide (14)

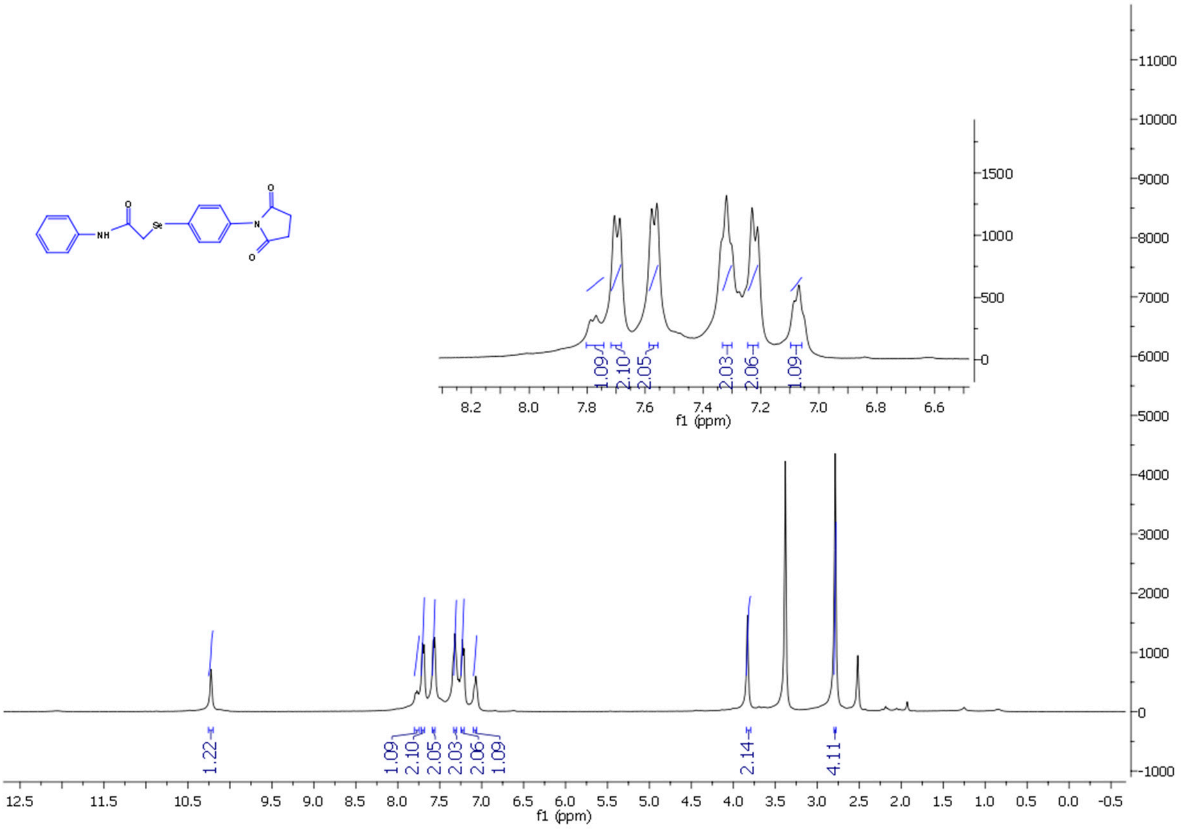

^{1}HNMR chart of compound 14.

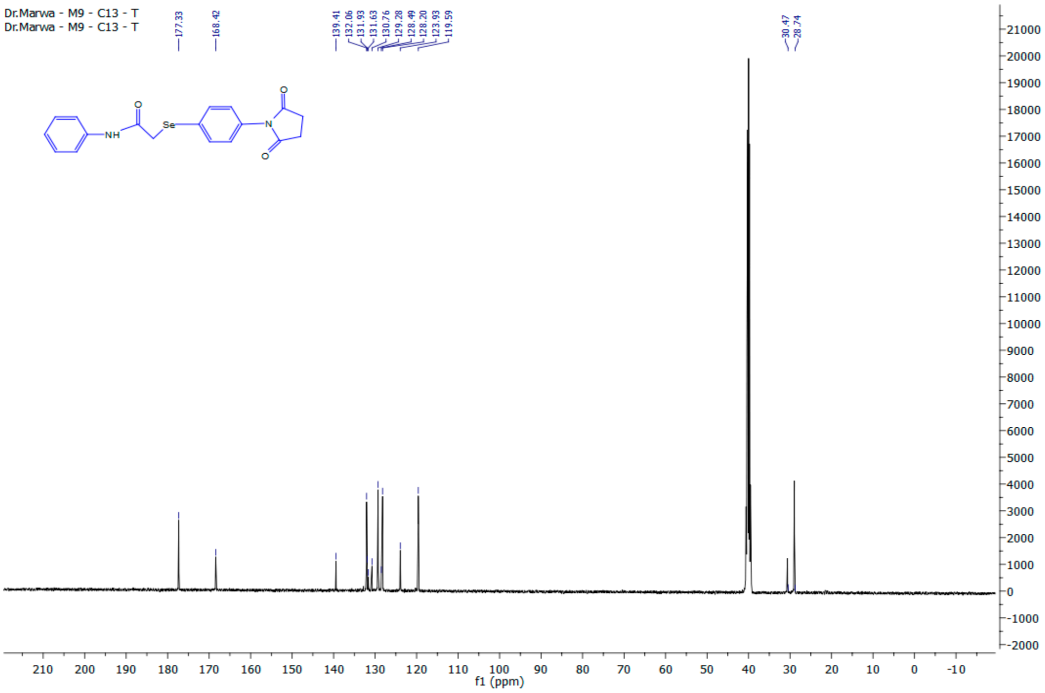

^{13}CNMR chart of compound 14.

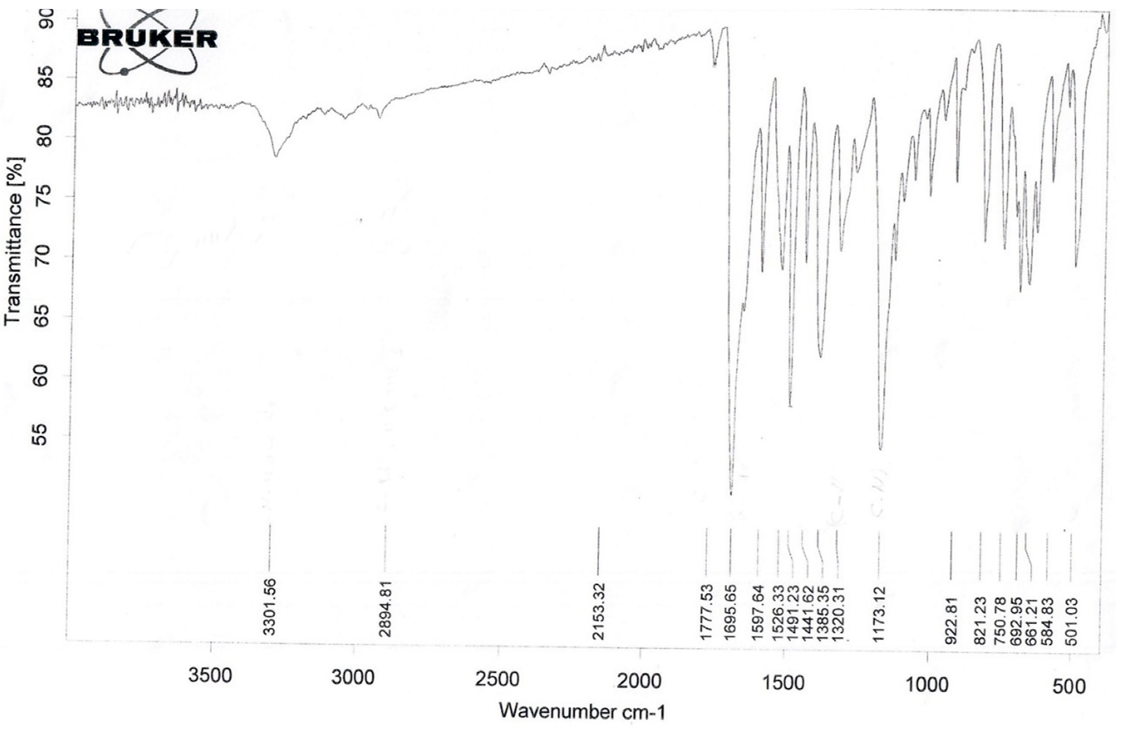

IR chart of compound 14.

09-Jan-07 18:40:45

Cairo University
Micro Analytical Center

DI Analysis
Shimadzu Qp-2010 Plus

Sample Information

Analyzed by	: Dr. Mai Younis
Analyzed	: 09/01/2007 06:30:54 ,
Sample Name	: M9
Sample ID	:
Customer Name	: Dr.Mohamed Soliman - Science - Cairo
Data File	: C:\GCMSsolution\Data\Project1\M9.QGD
Org Data File	: C:\GCMSsolution\Data\Project1\M9.QGD
Method File	: C:\GCMSsolution\Data\Project1\High Temperature Op
Org Method File	: C:\GCMSsolution\Data\Project1\High Temperature Op
Report File	:
Tuning File	: C:\GCMSsolution\System\Tune1_default.qgt
$EndIf$Modified by	: Dr. Mai Younis
Modified	: 09/01/2007 06:35:37 ,

Method

——— Analytical Line 1 ———

IonSourceTemp	:250.00 °C
[MS Table]	
--Group 1 - Event 1--	
Start Time	:0.00min
End Time	:10.00min
ACQ Mode	:Scan
Event Time	:0.50sec
Scan Speed	:1000
Start m/z	:50.00
End m/z	:510.00
Electron Voltage	: 70 eV
Ionization Mode	: EI

C:\GCMSsolution\Data\Project1\M9.QGD

Chromatogram M9 C:\GCMSsolution\Data\Project1\M9.QGD

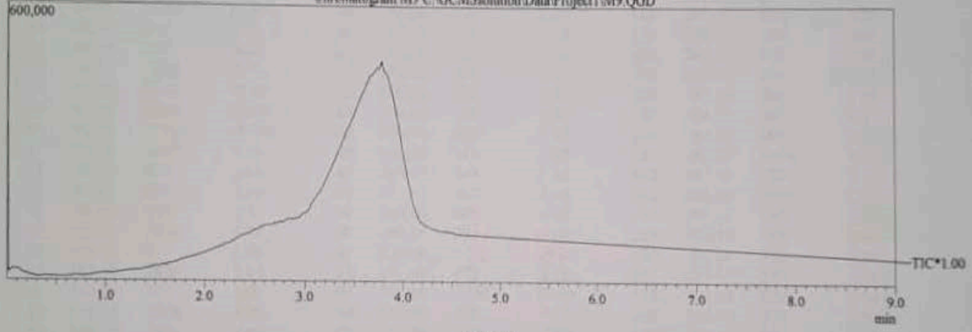

Spectrum

Line#:1 R.Time:4.2(Scan#:500)
MassPeaks:144
RawMode:Single 4.2(500) BasePeak:93(17151)
BG Mode:None Group 1 - Event 1

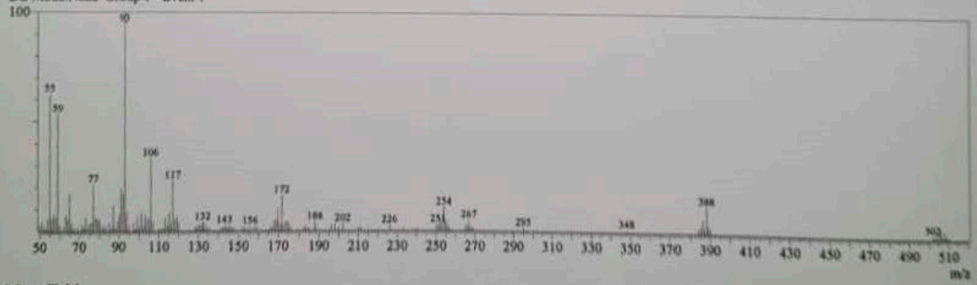

Mass Table
Line#:1 R.Time:4.2(Scan#:500)
MassPeaks:144
RawMode:Single 4.2(500) BasePeak:93(17151)
BG Mode:None Group 1 - Event 1

#	m/z	Abs. In	Rel. Int.	#	m/z	Abs. In	Rel. Int.	#	m/z	Abs. In	Rel. Int.
1	50.00	423	2.47	4	53.00	268	1.56	7	56.00	961	5.60
2	50.95	816	4.76	5	54.05	964	5.62	8	57.00	1338	7.80
3	51.95	417	2.43	6	55.05	10736	62.60	9	58.05	1097	6.40

1 / 2

Mass chart of compound 14.

09-Jan-07 18:40:45

#	m/z	Abs. In	Rel. Int.
10	59.05	9140	53.29
11	59.95	478	2.79
12	62.05	359	2.09
13	63.00	1220	7.11
14	64.05	964	5.62
15	65.05	2926	17.06
16	65.95	609	3.55
17	66.90	338	1.97
18	68.90	332	1.94
19	71.15	544	3.17
20	72.15	307	1.79
21	73.05	1031	6.01
22	74.10	270	1.57
23	75.10	590	3.44
24	76.15	640	3.73
25	77.05	3662	21.35
26	78.15	1126	6.57
27	79.10	945	5.51
28	80.05	842	4.91
29	81.20	455	2.65
30	82.20	279	1.63
31	83.20	399	2.33
32	85.10	644	3.75
33	86.10	228	1.33
34	87.10	1996	11.64
35	88.15	349	2.03
36	89.15	759	4.43
37	90.15	1322	7.71
38	91.10	3364	19.61
39	92.15	2880	16.79
40	93.10	17151	100.00
41	94.10	1497	8.73
42	95.00	446	2.60
43	97.10	626	3.65
44	98.10	210	1.22
45	99.10	1079	6.29
46	100.15	324	1.89
47	101.15	1489	8.68
48	102.25	357	2.08
49	103.15	1338	7.80
50	104.15	1011	5.89
51	105.15	965	5.63
52	106.10	5740	33.47
53	107.15	672	3.92
54	110.10	201	1.17

#	m/z	Abs. In	Rel. Int.
55	111.10	209	1.22
56	112.20	231	1.35
57	113.15	1032	6.02
58	114.15	437	2.55
59	115.15	1402	8.17
60	116.15	577	3.36
61	117.15	3945	23.00
62	118.15	835	4.87
63	119.10	1098	6.40
64	120.15	651	3.80
65	128.20	281	1.64
66	129.20	303	1.77
67	130.20	486	2.83
68	131.10	382	2.23
69	132.15	668	3.89
70	133.15	563	3.28
71	134.20	321	1.87
72	135.20	204	1.19
73	141.20	236	1.38
74	142.20	210	1.22
75	143.00	404	2.36
76	144.00	326	1.90
77	145.00	284	1.66
78	146.00	383	2.23
79	147.00	257	1.50
80	148.00	220	1.28
81	152.00	231	1.35
82	153.00	207	1.21
83	154.00	239	1.39
84	156.00	329	1.92
85	158.00	233	1.36
86	159.00	318	1.85
87	166.00	244	1.42
88	167.00	247	1.44
89	168.00	802	4.68
90	169.05	796	4.64
91	170.00	1732	10.10
92	171.05	472	2.75
93	172.00	2748	16.02
94	172.95	468	2.73
95	174.05	734	4.28
96	175.10	671	3.91
97	176.10	380	2.22
98	182.10	228	1.33
99	183.10	350	2.04

#	m/z	Abs. In	Rel. Int.
100	184.10	212	1.24
101	185.10	218	1.27
102	188.15	656	3.82
103	189.10	252	1.47
104	196.00	399	2.33
105	198.00	521	3.04
106	200.00	292	1.70
107	202.20	522	3.04
108	210.20	286	1.67
109	211.20	252	1.47
110	226.20	401	2.34
111	233.20	217	1.27
112	238.20	231	1.35
113	240.20	209	1.22
114	250.10	428	2.50
115	251.15	468	2.73
116	252.10	986	5.75
117	253.10	473	2.76
118	254.10	1831	10.68
119	255.05	819	4.78
120	256.00	417	2.43
121	257.00	201	1.17
122	265.00	402	2.34
123	266.10	430	2.51
124	267.05	832	4.85
125	268.10	330	1.92
126	269.10	273	1.59
127	295.10	233	1.36
128	348.10	202	1.18
129	384.15	390	2.27
130	385.20	606	3.53
131	386.20	1106	6.45
132	387.15	386	2.25
133	388.20	2092	12.20
134	389.10	545	3.18
135	390.10	449	2.62
136	502.10	310	1.81
137	503.10	212	1.24
138	504.10	538	3.14
139	505.10	332	1.94
140	506.15	732	4.27
141	507.10	254	1.48
142	508.10	614	3.58
143	509.10	247	1.44
144	510.10	236	1.38

Mass chart of compound 14.

4. -((4-(Methylselanyl)phenyl)amino)-4-oxobut-2-enoic acid (15)

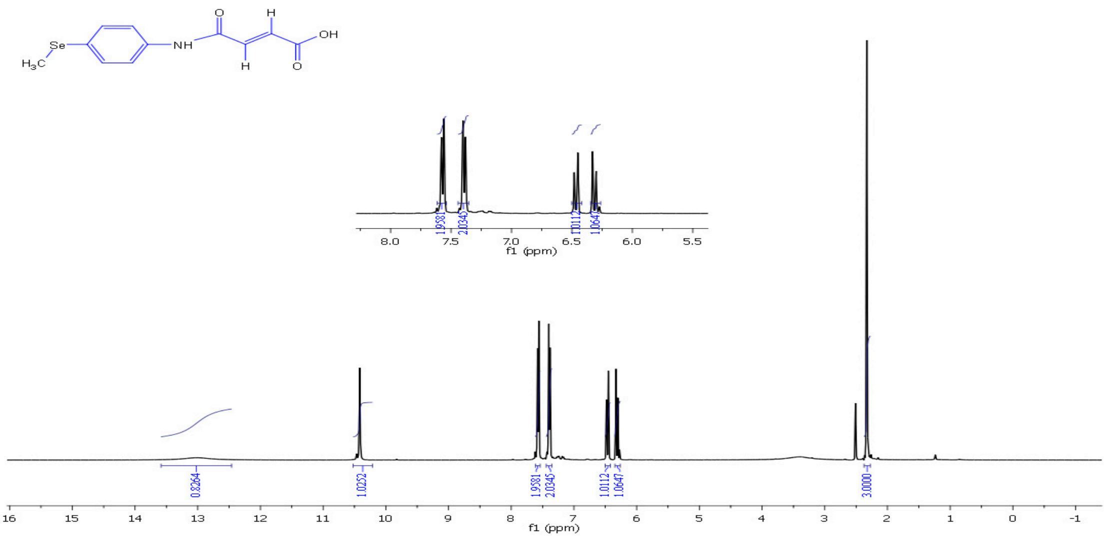

^{1}HNMR chart of compound 15.

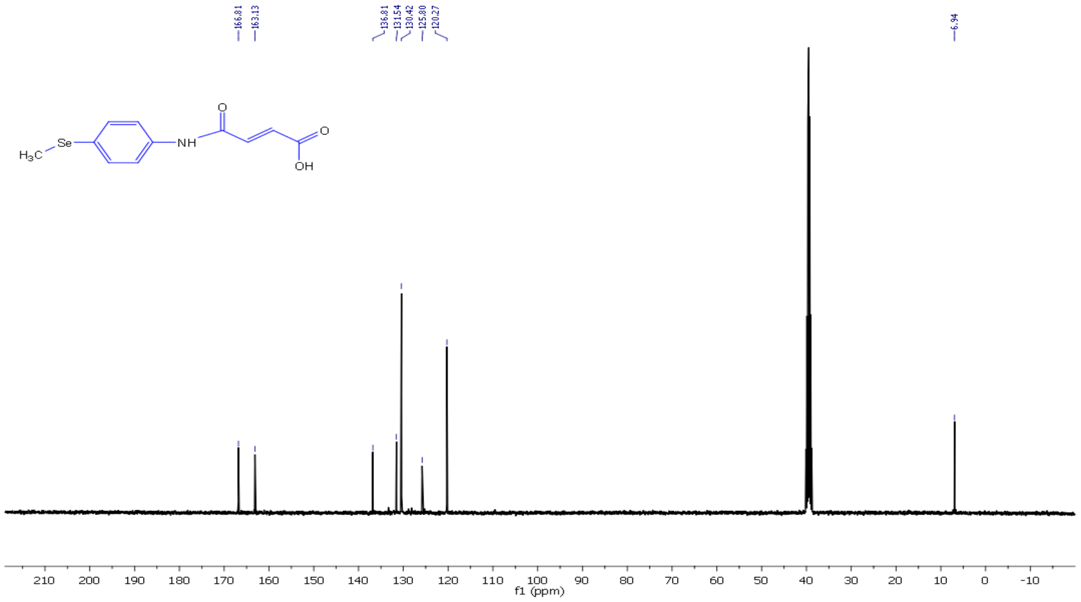

^{13}CNMR chart of compound 15.

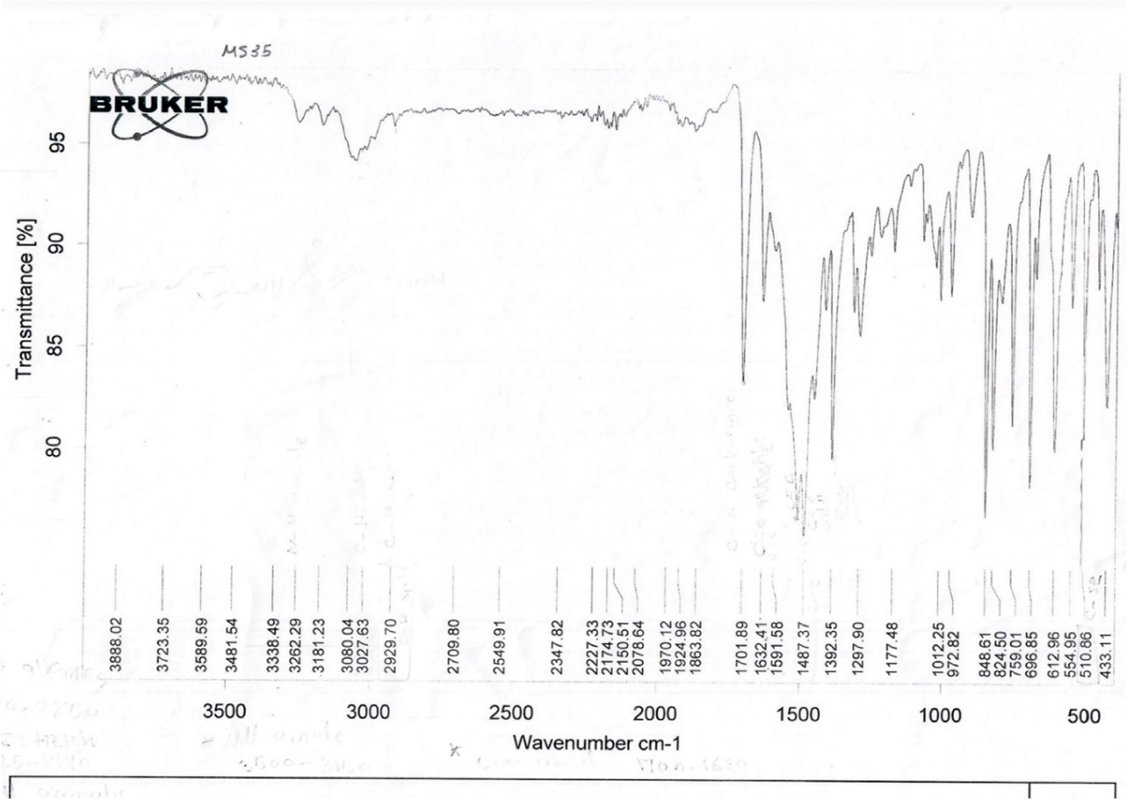

IR chart of compound 15.

4. -((4-(Methylselanyl)phenyl)amino)-4-oxobutanoic acid (16)

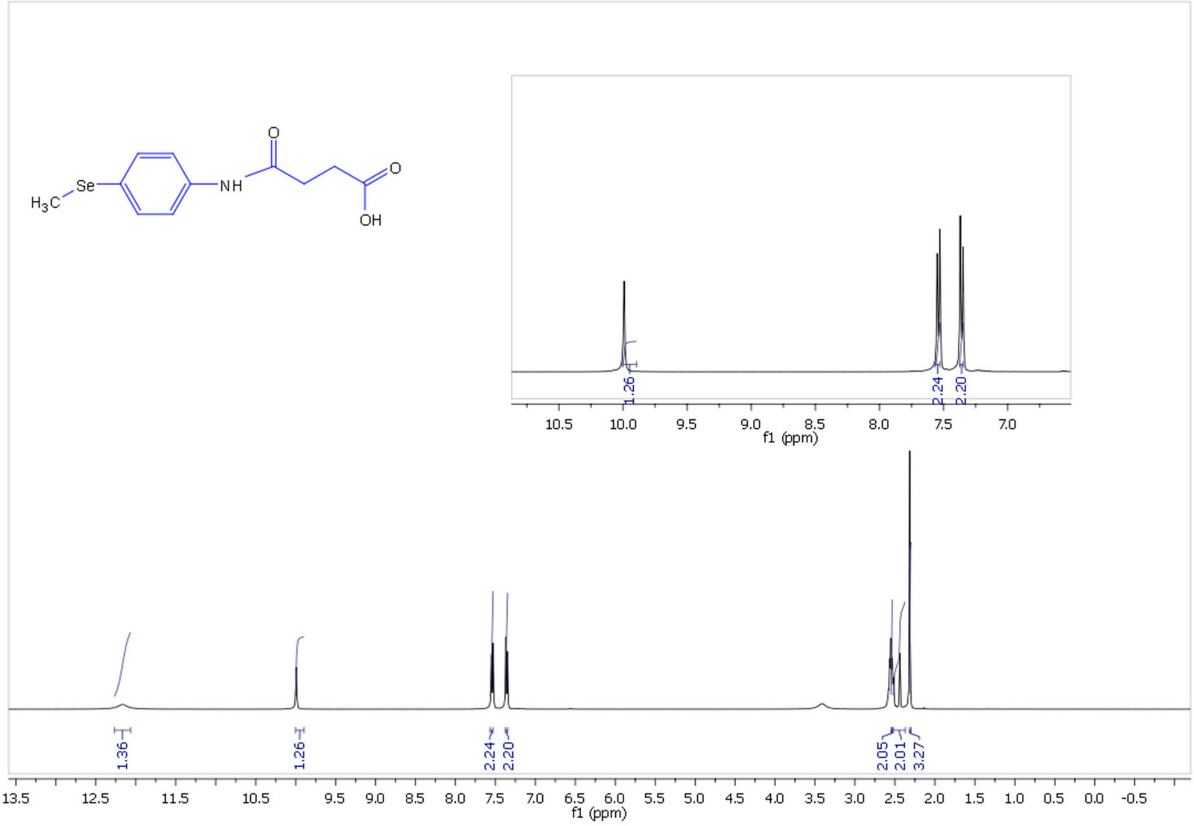

^{1}HNMR chart of compound 16.

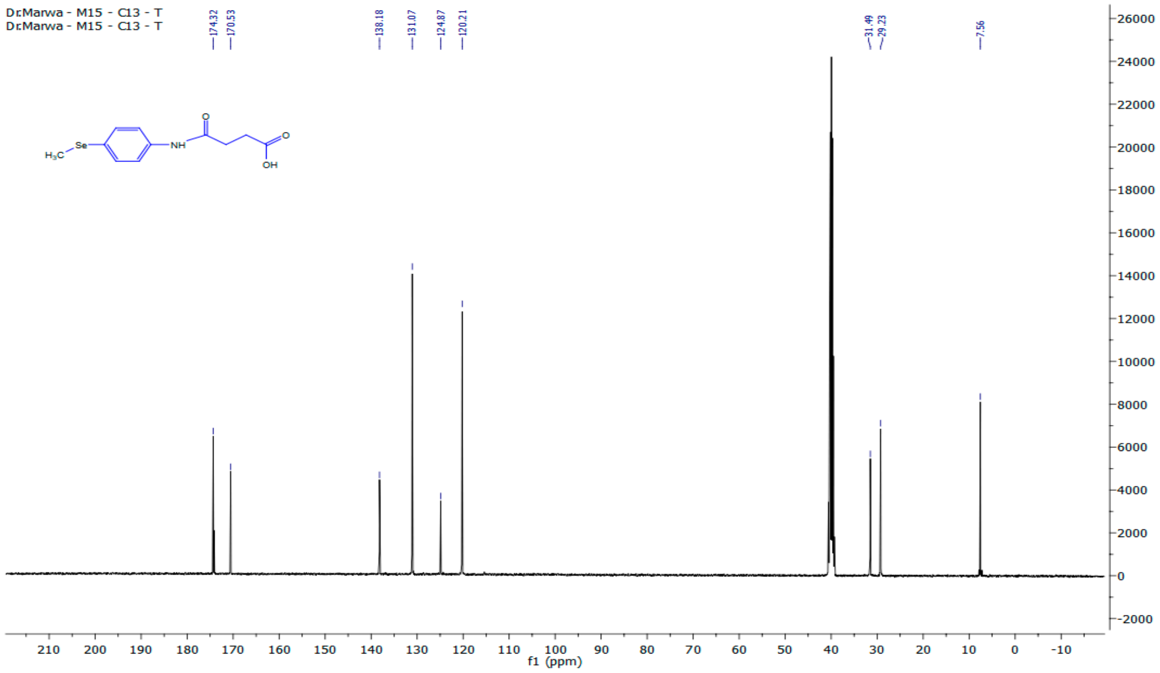

^{13}CNMR chart of compound 16.

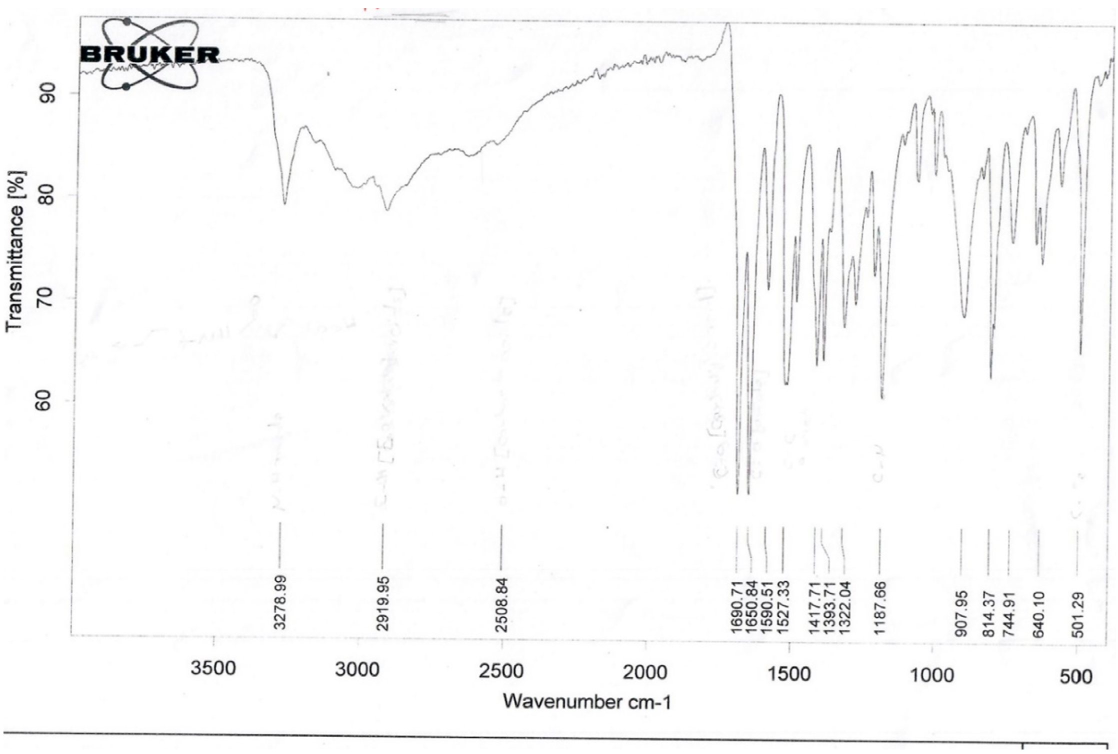

IR chart of compound 16.

03-Jan-07 19:55:25

Cairo University
Micro Analytical Center

DI Analysis
Shimadzu Qp-2010 Plus

Sample Information

Analyzed by	: Dr. Mai Younis
Analyzed	: 03/01/2007 07:48:46 ↗
Sample Name	: M15
Sample ID	:
Customer Name	: Dr.Mohamed Soliman - Science - Cairo
Data File	: C:\GCMSsolution\Data\Project1\M15.QGD
Org Data File	: C:\GCMSsolution\Data\Project1\M15.QGD
Method File	: C:\GCMSsolution\Data\Project1\High Temperature Op
Org Method File	: C:\GCMSsolution\Data\Project1\High Temperature Op
Report File	:
Tuning File	: C:\GCMSsolution\System\Tune1_default.qgt
$EndIf$Modified by	: Dr. Mai Younis
Modified	: 03/01/2007 07:53:06 ↗

Method

—— Analytical Line 1 ——

IonSourceTemp	:250.00 °C
[MS Table]	
--Group 1 - Event 1--	
Start Time	:0.00min
End Time	:10.00min
ACQ Mode	:Scan
Event Time	:0.50sec
Scan Speed	:1000
Start m/z	:50.00
End m/z	:510.00
Electron Voltage	: 70 eV
Ionization Mode	: EI

C:\GCMSsolution\Data\Project1\M15.QGD

Chromatogram M15 C:\GCMSsolution\Data\Project1\M15.QGD

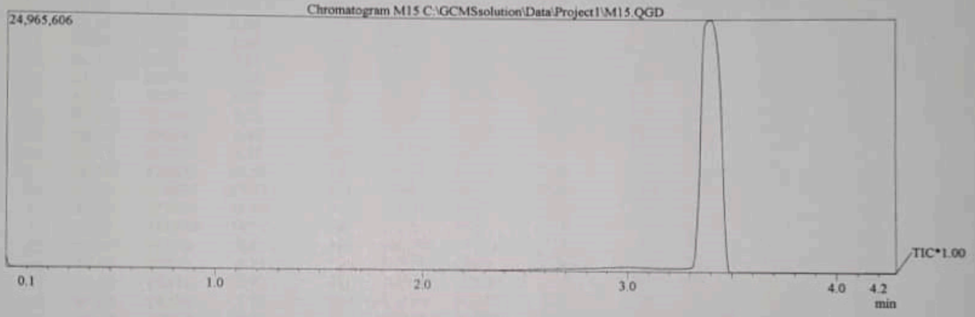

Spectrum

Line#:1 R.Time:3.4(Scan#:407)
MassPeaks:394
RawMode:Single 3.4(407) BasePeak:287(2089556)
BG Mode:None Group 1 - Event 1

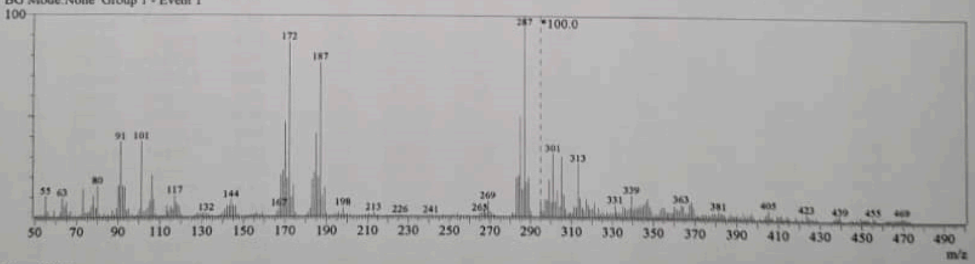

Mass Table
Line#:1 R.Time:3.4(Scan#:407)
MassPeaks:394
RawMode:Single 3.4(407) BasePeak:287(2089556)
BG Mode:None Group 1 - Event 1

#	m/z	Abs. In	Rel. Int.	#	m/z	Abs. In	Rel. Int.	#	m/z	Abs. In	Rel. Int.
1	50.05	17581	0.84	4	53.05	17686	0.85	7	56.05	62802	3.01
2	51.05	22534	1.08	5	54.15	30586	1.46	8	57.10	16265	0.78
3	52.05	28157	1.35	6	55.05	214920	10.29	9	58.15	12379	0.59

1 / 3

Mass chart of compound 16.

#	m/z	Abs. In	Rel. Int.	#	m/z	Abs. In	Rel. Int.	#	m/z	Abs. In	Rel. Int.
10	59.10	72260	3.46	79	128.15	38165	1.83	148	197.05	17331	0.83
11	60.10	6482	0.31	80	129.10	30689	1.47	149	198.00	88777	4.25
12	61.10	14902	0.71	81	130.10	36759	1.76	150	199.00	16978	0.81
13	62.15	48916	2.34	82	131.15	39269	1.88	151	200.00	24246	1.16
14	63.05	196136	9.39	83	132.15	41664	1.99	152	201.10	15726	0.75
15	64.05	115658	5.54	84	133.15	30199	1.45	153	202.05	3955	0.19
16	65.05	159959	7.66	85	134.15	7024	0.34	154	203.10	3054	0.15
17	66.10	35310	1.69	86	135.15	14680	0.70	155	204.15	2804	0.13
18	67.05	62264	2.98	87	136.05	4147	0.20	156	205.15	1512	0.07
19	68.10	6596	0.32	88	137.05	5741	0.27	157	206.10	4425	0.21
20	69.05	15934	0.76	89	138.05	9649	0.46	158	207.05	3297	0.16
21	70.05	6240	0.30	90	139.05	21669	1.04	159	208.05	3025	0.14
22	71.05	15009	0.72	91	140.05	50307	2.41	160	209.00	9794	0.47
23	72.05	23517	1.13	92	141.05	80621	3.86	161	210.05	10722	0.51
24	73.05	287776	13.77	93	142.05	114802	5.49	162	211.00	22684	1.09
25	74.05	49683	2.38	94	143.05	109432	5.24	163	212.05	8847	0.42
26	75.05	44227	2.12	95	144.05	175341	8.39	164	213.00	37367	1.79
27	76.05	57749	2.76	96	145.05	120423	5.76	165	214.00	9646	0.46
28	77.10	109404	5.24	97	146.10	109319	5.23	166	215.05	8231	0.39
29	78.05	221220	10.59	98	147.10	34255	1.64	167	215.95	2207	0.11
30	79.15	94231	4.51	99	148.10	4594	0.22	168	217.15	1503	0.07
31	80.05	321571	15.39	100	149.05	2331	0.11	169	218.05	1098	0.05
32	81.05	35840	1.72	101	150.05	2360	0.11	170	219.05	4946	0.24
33	82.10	11682	0.56	102	151.05	5296	0.25	171	220.00	1407	0.07
34	83.05	34698	1.66	103	152.05	10064	0.48	172	221.05	2167	0.10
35	84.15	17021	0.81	104	153.05	14012	0.67	173	222.05	4659	0.22
36	85.15	26825	1.28	105	154.05	21357	1.02	174	223.05	5285	0.25
37	86.05	18257	0.87	106	155.05	22471	1.08	175	224.00	9612	0.46
38	87.10	66999	3.21	107	156.05	41473	1.98	176	225.05	6570	0.31
39	88.15	29614	1.42	108	157.05	28160	1.35	177	226.00	14482	0.69
40	89.15	86792	4.15	109	158.10	16183	0.77	178	227.05	5162	0.25
41	90.10	325633	15.58	110	159.05	42041	2.01	179	228.00	3465	0.17
42	91.10	775436	37.11	111	160.05	6895	0.33	180	229.10	1710	0.08
43	92.10	327406	15.67	112	161.10	8466	0.41	181	230.20	839	0.04
44	93.05	311848	14.92	113	162.05	1687	0.08	182	231.20	1178	0.06
45	94.00	71172	3.41	114	163.05	1841	0.09	183	232.15	622	0.03
46	95.00	90073	4.31	115	164.05	4164	0.20	184	233.15	1195	0.06
47	96.05	19271	0.92	116	165.05	10239	0.49	185	234.05	620	0.03
48	97.05	24737	1.18	117	166.05	54316	2.60	186	235.05	1171	0.06
49	98.15	14064	0.67	118	167.05	84681	4.05	187	236.05	1699	0.08
50	99.15	28019	1.34	119	168.05	436951	20.91	188	237.05	2994	0.14
51	100.15	77714	3.72	120	169.05	477440	22.85	189	238.05	4599	0.22
52	101.05	779259	37.29	121	170.05	985109	47.14	190	239.05	8560	0.41
53	102.10	64188	3.07	122	171.05	391690	18.75	191	240.05	7329	0.35
54	103.10	65001	3.11	123	172.05	181185	86.71	192	241.05	11100	0.53
55	104.15	94868	4.54	124	172.95	199194	9.53	193	242.05	7457	0.36
56	105.15	170858	8.18	125	174.05	327860	15.69	194	243.05	5167	0.25
57	106.15	436976	20.91	126	175.00	39367	1.88	195	244.05	2548	0.12
58	107.10	187714	8.98	127	176.15	8428	0.40	196	245.05	2392	0.11
59	108.10	27407	1.31	128	177.15	3196	0.15	197	246.25	1578	0.08
60	109.10	13706	0.66	129	178.05	1827	0.09	198	247.25	11075	0.53
61	110.15	13926	0.67	130	179.05	3206	0.15	199	248.20	5223	0.25
62	111.15	16858	0.81	131	180.05	11700	0.56	200	249.05	1914	0.09
63	112.15	16257	0.78	132	181.05	43872	2.10	201	250.05	5865	0.28
64	113.15	115233	5.51	133	182.05	81717	3.91	202	251.05	6677	0.32
65	114.05	59668	2.86	134	183.05	387229	18.53	203	252.05	13833	0.66
66	115.05	105126	5.03	135	184.05	464882	22.25	204	253.05	4888	0.23
67	116.15	78157	3.74	136	185.05	857299	41.03	205	254.05	26554	1.27
68	117.10	223964	10.72	137	186.05	405310	19.40	206	254.95	5686	0.27
69	118.05	146104	6.99	138	187.05	160064	76.60	207	256.05	5929	0.28
70	119.10	114753	5.49	139	187.95	225414	10.79	208	257.15	1994	0.10
71	120.05	38768	1.86	140	189.05	304356	14.57	209	258.25	1582	0.08
72	121.10	10726	0.51	141	189.95	33951	1.62	210	259.25	9327	0.45
73	122.15	4847	0.23	142	191.05	3668	0.18	211	260.15	3348	0.16
74	123.20	4134	0.20	143	192.05	6654	0.32	212	261.15	2919	0.14
75	124.15	2961	0.14	144	193.05	6043	0.29	213	262.15	4234	0.20
76	125.15	6932	0.33	145	194.00	18380	0.88	214	263.10	6830	0.33
77	126.15	11885	0.57	146	195.05	19569	0.94	215	264.15	10188	0.49
78	127.15	27977	1.34	147	196.00	45022	2.15	216	265.15	34523	1.65

Mass chart of compound 16.

03-Jan-07 19:55:25

#	m/z	Abs. In	Rel. Int.
217	266.15	45458	2.18
218	267.10	84745	4.06
219	268.15	43059	2.06
220	269.10	161260	7.72
221	270.00	63358	3.03
222	271.05	37486	1.79
223	272.05	20513	0.98
224	273.05	5892	0.28
225	274.05	3429	0.16
226	275.10	2324	0.11
227	276.15	2080	0.10
228	277.15	2707	0.13
229	278.15	2967	0.14
230	279.15	1987	0.10
231	280.15	3850	0.18
232	281.15	39626	1.90
233	282.15	30595	1.46
234	283.10	408831	19.57
235	284.15	431177	20.63
236	285.10	105568	50.52
237	286.15	284952	13.64
238	287.10	208955	100.00
239	288.05	363754	17.41
240	289.10	409465	19.60
241	290.05	64052	3.07
242	291.05	7848	0.38
243	292.05	1304	0.06
244	293.25	1184	0.06
245	294.20	1049	0.05
246	295.15	1199	0.06
247	296.25	582	0.03
248	297.15	1839	0.09
249	298.20	1780	0.09
250	299.15	3948	0.19
251	300.15	1514	0.07
252	301.10	6570	0.31
253	302.15	1648	0.08
254	303.20	2801	0.13
255	304.35	1115	0.05
256	305.30	6258	0.30
257	306.25	2332	0.11
258	307.20	841	0.04
259	308.45	421	0.02
260	309.35	505	0.02
261	310.35	495	0.02
262	311.40	1135	0.05
263	312.45	1039	0.05
264	313.35	5571	0.27
265	314.30	1985	0.09
266	315.25	968	0.05
267	316.25	739	0.04
268	317.30	1958	0.09
269	318.25	1306	0.06
270	319.25	825	0.04
271	320.35	897	0.04
272	321.25	1581	0.08
273	322.25	464	0.02
274	323.35	974	0.05
275	324.25	395	0.02
276	325.25	639	0.03

#	m/z	Abs. In	Rel. Int.
277	326.35	349	0.02
278	327.35	589	0.03
279	328.30	366	0.02
280	329.40	516	0.02
281	330.45	409	0.02
282	331.40	1290	0.06
283	332.25	578	0.03
284	333.35	535	0.03
285	334.35	541	0.03
286	335.35	1220	0.06
287	336.30	985	0.05
288	337.35	816	0.04
289	338.40	1033	0.05
290	339.40	2332	0.11
291	340.35	846	0.04
292	341.35	1193	0.06
293	342.25	938	0.04
294	343.25	1274	0.06
295	344.25	1103	0.05
296	345.25	1356	0.06
297	346.25	1648	0.08
298	347.30	1969	0.09
299	348.25	1158	0.06
300	349.30	722	0.03
301	350.30	255	0.01
302	351.35	427	0.02
303	352.35	339	0.02
304	353.35	839	0.04
305	354.35	1071	0.05
306	355.30	1121	0.05
307	356.35	630	0.03
308	357.25	641	0.03
309	358.25	649	0.03
310	359.35	601	0.03
311	360.25	1294	0.06
312	361.30	945	0.05
313	362.35	885	0.04
314	363.40	1425	0.07
315	364.35	1304	0.06
316	365.45	631	0.03
317	366.45	570	0.03
318	367.45	1430	0.07
319	368.50	1743	0.08
320	369.35	1085	0.05
321	370.40	388	0.02
322	371.35	355	0.02
323	372.30	249	0.01
324	373.35	447	0.02
325	374.30	468	0.02
326	375.45	468	0.02
327	376.45	393	0.02
328	377.45	345	0.02
329	378.45	565	0.03
330	379.45	606	0.03
331	380.50	442	0.02
332	381.45	765	0.04
333	382.45	549	0.03
334	383.40	609	0.03
335	384.40	375	0.02
336	386.20	233	0.01

#	m/z	Abs. In	Rel. Int.
337	387.20	538	0.03
338	388.20	382	0.02
339	389.15	331	0.02
340	390.10	430	0.02
341	391.10	270	0.01
342	392.10	270	0.01
343	393.40	713	0.03
344	394.35	329	0.02
345	395.35	456	0.02
346	396.40	398	0.02
347	397.40	665	0.03
348	398.40	239	0.01
349	401.40	209	0.01
350	403.30	266	0.01
351	404.30	530	0.03
352	405.30	961	0.05
353	406.30	274	0.01
354	407.30	297	0.01
355	409.55	495	0.02
356	410.45	410	0.02
357	411.45	526	0.03
358	413.10	206	0.01
359	414.10	473	0.02
360	415.10	292	0.01
361	418.40	364	0.02
362	421.40	250	0.01
363	423.50	614	0.03
364	424.45	397	0.02
365	425.50	236	0.01
366	426.50	234	0.01
367	428.00	404	0.02
368	436.60	217	0.01
369	437.55	422	0.02
370	438.60	438	0.02
371	439.55	456	0.02
372	444.60	201	0.01
373	445.60	222	0.01
374	447.60	284	0.01
375	449.60	415	0.02
376	450.60	225	0.01
377	451.40	385	0.02
378	452.40	252	0.01
379	453.40	257	0.01
380	455.55	452	0.02
381	456.50	318	0.02
382	461.50	226	0.01
383	462.50	228	0.01
384	463.50	326	0.02
385	464.50	206	0.01
386	465.50	298	0.01
387	466.50	260	0.01
388	467.50	335	0.02
389	468.45	333	0.02
390	469.45	502	0.02
391	470.25	303	0.01
392	471.20	420	0.02
393	472.20	222	0.01
394	473.20	279	0.01

3 / 3

Mass chart of compound 16.

2. -((4-Formamidophenyl)selanyl)-N-phenylacetamide (17)

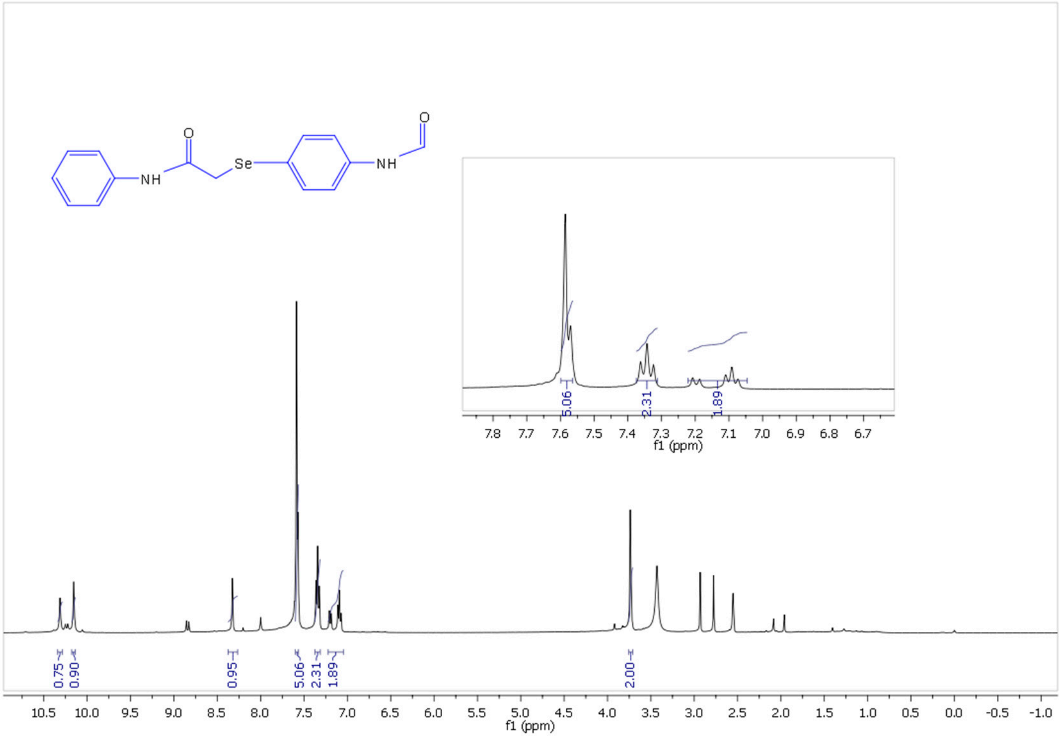

^{1}HNMR chart of compound 17.

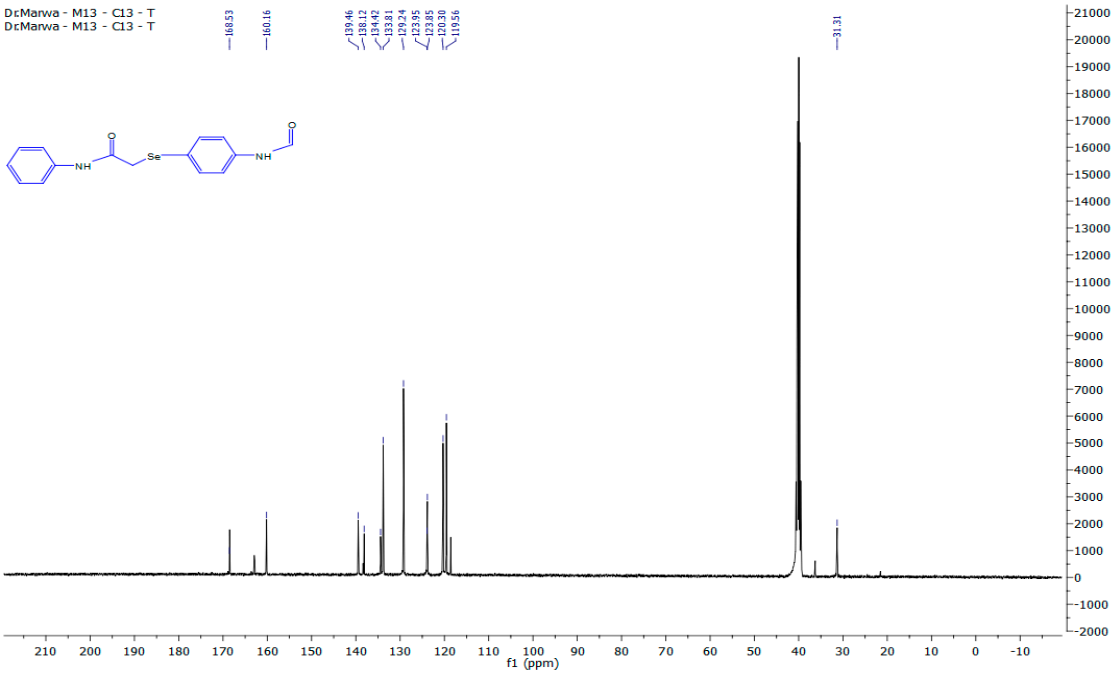

^{13}CNMR chart of compound 17.

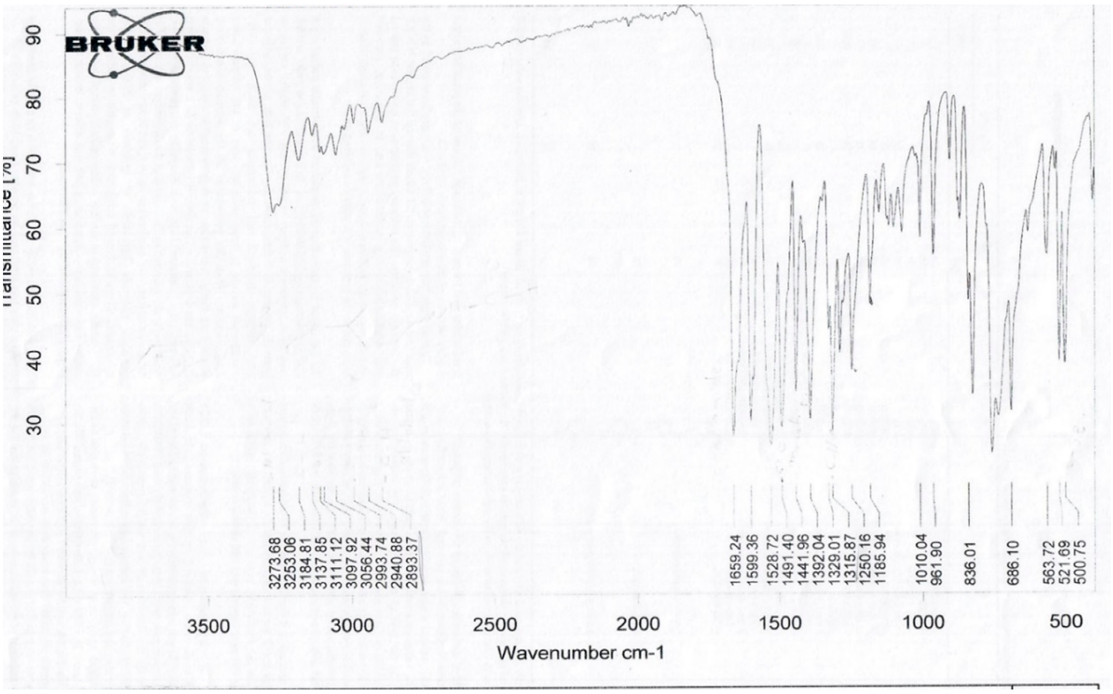

IR chart of compound 17.

09-Jan-07 19:01:40

Cairo University
Micro Analytical Center

DI Analysis
Shimadzu Qp-2010 Plus

Sample Information	
Analyzed by	: Dr. Mai Younis
Analyzed	: 09/01/2007 06:55:02
Sample Name	: M13
Sample ID	:
Customer Name	: Dr.Mohamed Soliman - Science - Cairo
Data File	: C:\GCMSsolution\Data\Project1\M13.QGD
Org Data File	: C:\GCMSsolution\Data\Project1\M13.QGD
Method File	: C:\GCMSsolution\Data\Project1\High Temperature Op
Org Method File	: C:\GCMSsolution\Data\Project1\High Temperature Op
Report File	:
Tuning File	: C:\GCMSsolution\System\Tune1_default.qgt
$EndIf$Modified by	: Dr. Mai Younis
Modified	: 09/01/2007 06:59:34

Method

—— Analytical Line 1 ——

IonSourceTemp	:250.00 °C
[MS Table]	
--Group 1 - Event 1--	
Start Time	:0.00min
End Time	:10.00min
ACQ Mode	:Scan
Event Time	:0.50sec
Scan Speed	:1000
Start m/z	:50.00
End m/z	:510.00
Electron Voltage	: 70 eV
Ionization Mode	: EI

C:\GCMSsolution\Data\Project1\M13.QGD

Chromatogram M13 C:\GCMSsolution\Data\Project1\M13.QGD

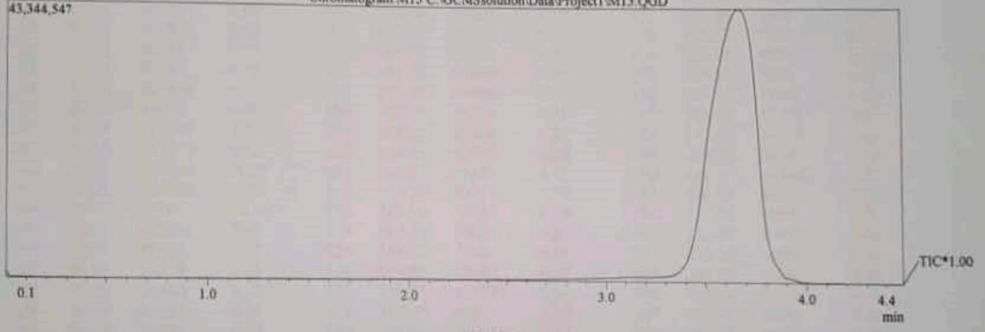

Spectrum

Line#:1 R.Time:3.7(Scan#:443)
MassPeaks:451
RawMode:Single 3.7(443) BasePeak:93(6620138)
BG Mode:None Group 1 - Event 1

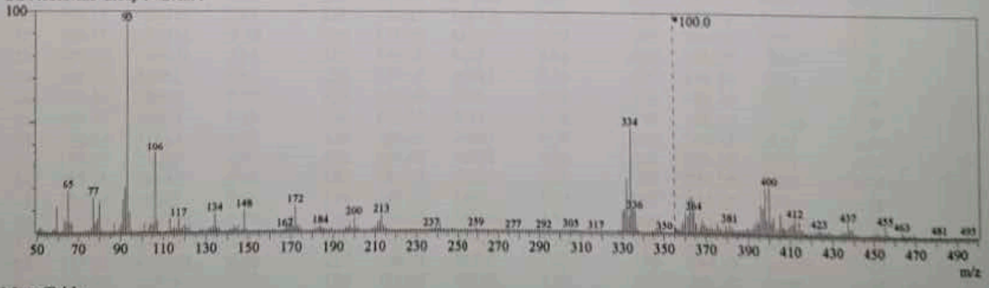

Mass Table
Line#:1 R.Time:3.7(Scan#:443)
MassPeaks:451
RawMode:Single 3.7(443) BasePeak:93(6620138)
BG Mode:None Group 1 - Event 1

#	m/z	Abs. In	Rel. Int.	#	m/z	Abs. In	Rel. Int.	#	m/z	Abs. In	Rel. Int.
1	50.05	64718	0.98	4	53.00	76921	1.16	7	56.05	17383	0.26
2	51.00	179194	2.71	5	54.05	52872	0.80	8	57.05	83642	1.26
3	52.00	121746	1.84	6	55.00	38990	0.59	9	58.05	103957	1.57

1 / 4

Mass chart of compound 17.

09-Jan-07 19:01:40

#	m/z	Abs. In	Rel. Int.	#	m/z	Abs. In	Rel. Int.	#	m/z	Abs. In	Rel. Int.
10	59.05	762738	11.52	79	128.20	48687	0.74	148	197.05	94900	1.43
11	60.00	39180	0.59	80	129.15	52354	0.79	149	198.05	228567	3.45
12	61.05	28877	0.44	81	130.15	39904	0.60	150	199.05	60789	0.92
13	62.05	86176	1.30	82	131.20	102884	1.55	151	200.05	402020	6.07
14	63.05	376489	5.69	83	132.15	127840	1.93	152	201.10	94058	1.42
15	64.05	343556	5.19	84	133.25	159204	2.40	153	202.05	81960	1.24
16	65.00	125441	18.95	85	134.15	558347	8.43	154	203.10	19982	0.30
17	66.00	316444	4.78	86	135.20	143865	2.17	155	204.20	5932	0.09
18	67.00	252023	3.81	87	136.15	120390	1.82	156	205.15	3390	0.05
19	68.05	20615	0.31	88	137.15	23229	0.35	157	206.15	2398	0.04
20	69.05	40637	0.61	89	138.15	8635	0.13	158	207.10	10630	0.16
21	70.05	20860	0.32	90	139.15	25633	0.39	159	208.15	14035	0.21
22	71.05	47083	0.71	91	140.15	32277	0.49	160	209.10	88855	1.34
23	72.05	18062	0.27	92	141.05	77301	1.17	161	210.10	113991	1.72
24	73.05	90935	1.37	93	142.10	88839	1.34	162	211.10	246505	3.72
25	74.05	51753	0.78	94	143.10	171345	2.59	163	212.15	284352	4.30
26	75.05	71606	1.08	95	144.05	106308	1.61	164	213.10	466052	7.04
27	76.05	123775	1.87	96	145.05	189105	2.86	165	214.10	152212	2.30
28	77.05	104915	15.85	97	146.10	40647	0.61	166	215.10	113542	1.72
29	78.05	291372	4.40	98	147.15	77680	1.17	167	216.05	30071	0.45
30	79.10	402618	6.08	99	148.10	652399	9.85	168	217.15	15291	0.23
31	80.05	904607	13.66	100	149.10	66252	1.00	169	218.25	3201	0.05
32	81.05	89362	1.35	101	150.15	14815	0.22	170	219.20	6522	0.10
33	82.15	13817	0.21	102	151.15	14249	0.22	171	220.25	1742	0.03
34	83.05	53150	0.80	103	152.10	10823	0.16	172	221.20	2590	0.04
35	84.15	16798	0.25	104	153.10	17164	0.26	173	222.25	1863	0.03
36	85.05	52281	0.79	105	154.10	20170	0.30	174	223.20	5394	0.08
37	86.15	36731	0.55	106	155.10	30508	0.46	175	224.15	5885	0.09
38	87.05	299345	4.52	107	156.05	26940	0.41	176	225.15	14245	0.22
39	88.15	39071	0.59	108	157.10	25250	0.38	177	226.25	17778	0.27
40	89.10	120474	1.82	109	158.15	15909	0.24	178	227.15	29058	0.44
41	90.15	277571	4.19	110	159.15	45018	0.68	179	228.15	9324	0.14
42	91.10	969734	14.65	111	160.15	11299	0.17	180	229.20	11346	0.17
43	92.15	134088	20.25	112	161.15	47777	0.72	181	230.25	4431	0.07
44	93.10	662013	100.00	113	162.10	48383	0.73	182	231.25	6060	0.09
45	94.10	627986	9.49	114	163.10	7700	0.12	183	232.30	2444	0.04
46	95.05	86841	1.31	115	164.05	3906	0.06	184	233.30	6090	0.09
47	96.10	15538	0.23	116	165.05	12040	0.18	185	234.25	3294	0.05
48	97.10	36660	0.55	117	166.05	42537	0.64	186	235.20	8393	0.13
49	98.15	16162	0.24	118	167.05	65312	0.99	187	236.15	6219	0.09
50	99.10	54922	0.83	119	168.05	191919	2.90	188	237.10	48682	0.74
51	100.15	40893	0.62	120	169.05	228777	3.46	189	238.15	51670	0.78
52	101.10	271913	4.11	121	170.05	429354	6.49	190	239.10	124788	1.88
53	102.15	51501	0.78	122	171.05	190978	2.88	191	240.15	41957	0.63
54	103.15	175218	2.65	123	172.05	778535	11.76	192	241.10	255247	3.86
55	104.10	272790	4.12	124	173.00	172532	2.61	193	242.05	40317	0.61
56	105.15	256316	3.87	125	174.05	145476	2.20	194	243.10	51441	0.78
57	106.15	237542	35.88	126	175.10	46359	0.70	195	244.05	9209	0.14
58	107.10	340534	5.14	127	176.15	9157	0.14	196	245.25	10412	0.16
59	108.10	33452	0.51	128	177.15	10381	0.16	197	246.35	7814	0.12
60	109.10	18940	0.29	129	178.10	3983	0.06	198	247.25	66234	1.00
61	110.15	10624	0.16	130	179.15	5016	0.08	199	248.20	25500	0.39
62	111.15	41814	0.63	131	180.10	23463	0.35	200	249.15	6025	0.09
63	112.15	33229	0.50	132	181.10	36838	0.56	201	250.20	2871	0.04
64	113.15	399427	6.03	133	182.10	72735	1.10	202	251.25	9309	0.14
65	114.15	88341	1.33	134	183.15	119878	1.81	203	252.15	5526	0.08
66	115.10	148778	2.25	135	184.05	145090	2.19	204	253.20	13094	0.20
67	116.15	94289	1.42	136	185.05	84927	1.28	205	254.15	5364	0.08
68	117.15	407008	6.15	137	186.05	81705	1.23	206	255.10	14478	0.22
69	118.10	127194	1.92	138	187.05	40030	0.60	207	256.25	4049	0.06
70	119.10	141374	2.14	139	188.15	24113	0.36	208	257.25	5968	0.09
71	120.15	214859	3.25	140	189.20	91054	1.38	209	258.35	6217	0.09
72	121.15	136567	2.06	141	190.15	16238	0.25	210	259.30	56857	0.86
73	122.15	144379	2.18	142	191.20	5303	0.08	211	260.25	12338	0.19
74	123.15	18376	0.28	143	192.05	2421	0.04	212	261.25	8431	0.13
75	124.20	4745	0.07	144	193.05	14591	0.22	213	262.25	4942	0.07
76	125.20	12316	0.19	145	194.05	17289	0.26	214	263.20	5977	0.09
77	126.25	18435	0.28	146	195.05	17423	0.26	215	264.25	3659	0.06
78	127.15	71096	1.07	147	196.05	95795	1.45	216	265.30	6020	0.09

Mass chart of compound 17.

09-Jan-07 19:01:40

#	m/z	Abs. In	Rel. Int.
217	266.25	2392	0.04
218	267.25	2519	0.04
219	268.25	1364	0.02
220	269.25	2243	0.03
221	270.35	1851	0.03
222	271.30	8262	0.12
223	272.25	3052	0.05
224	273.25	7648	0.12
225	274.20	3490	0.05
226	275.25	6435	0.10
227	276.25	3578	0.05
228	277.25	8899	0.13
229	278.25	2805	0.04
230	279.15	2261	0.03
231	280.15	1513	0.02
232	281.20	2562	0.04
233	282.15	1701	0.03
234	283.20	5198	0.08
235	284.25	2591	0.04
236	285.30	6313	0.10
237	286.25	3988	0.06
238	287.25	4385	0.07
239	288.15	5229	0.08
240	289.25	12326	0.19
241	290.15	10069	0.15
242	291.15	6834	0.10
243	292.15	15721	0.24
244	293.15	5461	0.08
245	294.10	4239	0.06
246	295.20	2617	0.04
247	296.15	1179	0.02
248	297.25	1758	0.03
249	298.30	810	0.01
250	299.30	1918	0.03
251	300.30	1439	0.02
252	301.30	3206	0.05
253	302.25	5124	0.08
254	303.25	12315	0.19
255	304.25	10805	0.16
256	305.30	53647	0.81
257	306.25	29714	0.45
258	307.25	8410	0.13
259	308.10	3871	0.06
260	309.15	2095	0.03
261	310.15	1089	0.02
262	311.35	1708	0.03
263	312.45	1195	0.02
264	313.40	5183	0.08
265	314.35	2253	0.03
266	315.35	2831	0.04
267	316.35	3310	0.05
268	317.35	16464	0.25
269	318.25	7596	0.11
270	319.25	4366	0.07
271	320.15	7480	0.11
272	321.30	5231	0.08
273	322.25	2753	0.04
274	323.40	5988	0.09
275	324.35	1733	0.03
276	325.25	1269	0.02
277	326.25	1134	0.02
278	327.25	4863	0.07
279	328.20	60062	0.91
280	329.25	51474	0.78
281	330.20	615934	9.30
282	331.25	674160	10.18
283	332.20	161486	24.39
284	333.25	489479	7.39
285	334.20	315106	47.60

#	m/z	Abs. In	Rel. Int.
286	335.15	715759	10.81
287	336.15	634481	9.58
288	337.15	126918	1.92
289	338.15	15393	0.23
290	339.35	3999	0.06
291	340.25	1389	0.02
292	341.25	1752	0.03
293	342.25	4295	0.06
294	343.25	5184	0.08
295	344.25	33478	0.51
296	345.25	36712	0.55
297	346.20	85266	1.29
298	347.25	35101	0.53
299	348.20	164510	2.48
300	349.15	40497	0.61
301	350.20	33236	0.50
302	351.15	8819	0.13
303	352.25	1703	0.03
304	353.45	2482	0.04
305	354.40	1545	0.02
306	355.40	1586	0.02
307	356.40	1156	0.02
308	357.25	1278	0.02
309	358.25	2576	0.04
310	359.25	3362	0.05
311	360.25	6111	0.09
312	361.45	4948	0.07
313	362.35	10306	0.16
314	363.40	10681	0.16
315	364.35	6214	0.09
316	365.35	3109	0.05
317	366.45	1259	0.02
318	367.45	2443	0.04
319	368.45	3725	0.06
320	369.45	2541	0.04
321	370.45	1816	0.03
322	371.35	1408	0.02
323	372.35	1876	0.03
324	373.35	1535	0.02
325	374.35	1404	0.02
326	375.35	3285	0.05
327	376.30	1646	0.02
328	377.35	1362	0.02
329	378.35	960	0.01
330	379.40	2518	0.04
331	380.35	1186	0.02
332	381.35	2813	0.04
333	382.40	1489	0.02
334	383.35	1063	0.02
335	384.35	1036	0.02
336	385.35	1020	0.02
337	386.35	1152	0.02
338	387.40	1196	0.02
339	388.35	1138	0.02
340	389.40	1822	0.03
341	390.35	1487	0.02
342	391.15	1121	0.02
343	392.15	1817	0.03
344	393.15	2981	0.05
345	394.15	4088	0.06
346	395.15	3615	0.05
347	396.10	8650	0.13
348	397.15	7903	0.12
349	398.10	13686	0.21
350	399.10	4279	0.06
351	400.15	14071	0.21
352	401.10	3751	0.06
353	402.10	5124	0.08
354	403.35	1483	0.02

#	m/z	Abs. In	Rel. Int.
355	404.35	2159	0.03
356	405.40	6396	0.10
357	406.35	2397	0.04
358	407.15	1804	0.03
359	408.10	1804	0.03
360	409.15	2233	0.03
361	410.15	3190	0.05
362	411.15	3417	0.05
363	412.15	4648	0.07
364	413.10	1796	0.03
365	414.15	4418	0.07
366	415.15	1428	0.02
367	416.10	1807	0.03
368	417.10	642	0.01
369	417.55	650	0.01
370	418.45	644	0.01
371	419.45	1030	0.02
372	420.55	636	0.01
373	421.55	1322	0.02
374	422.55	1071	0.02
375	423.50	1802	0.03
376	424.45	1519	0.02
377	425.55	1144	0.02
378	426.45	1143	0.02
379	427.25	636	0.01
380	428.20	1647	0.02
381	429.30	887	0.01
382	430.25	843	0.01
383	431.35	684	0.01
384	432.35	680	0.01
385	433.35	1037	0.02
386	434.30	1010	0.02
387	435.35	1719	0.03
388	436.35	1160	0.02
389	437.30	3846	0.06
390	438.35	1849	0.03
391	439.35	2395	0.04
392	440.30	951	0.01
393	441.45	843	0.01
394	442.45	652	0.01
395	443.65	419	0.01
396	444.55	470	0.01
397	445.45	451	0.01
398	446.50	497	0.01
399	447.50	598	0.01
400	448.50	566	0.01
401	449.55	908	0.01
402	450.55	751	0.01
403	451.50	1526	0.02
404	452.50	913	0.01
405	453.45	825	0.01
406	454.55	760	0.01
407	455.50	3054	0.05
408	456.50	1143	0.02
409	457.45	728	0.01
410	458.45	509	0.01
411	459.50	607	0.01
412	460.55	458	0.01
413	461.55	463	0.01
414	462.45	615	0.01
415	463.50	1478	0.02
416	464.45	769	0.01
417	465.50	703	0.01
418	466.45	587	0.01
419	467.40	855	0.01
420	468.45	568	0.01
421	469.50	1156	0.02
422	470.45	420	0.01
423	471.50	342	0.01

3 / 4

Mass chart of compound 17.

2.-((4-. Acetamidophenyl)selanyl)-N-phenylacetamide (18)

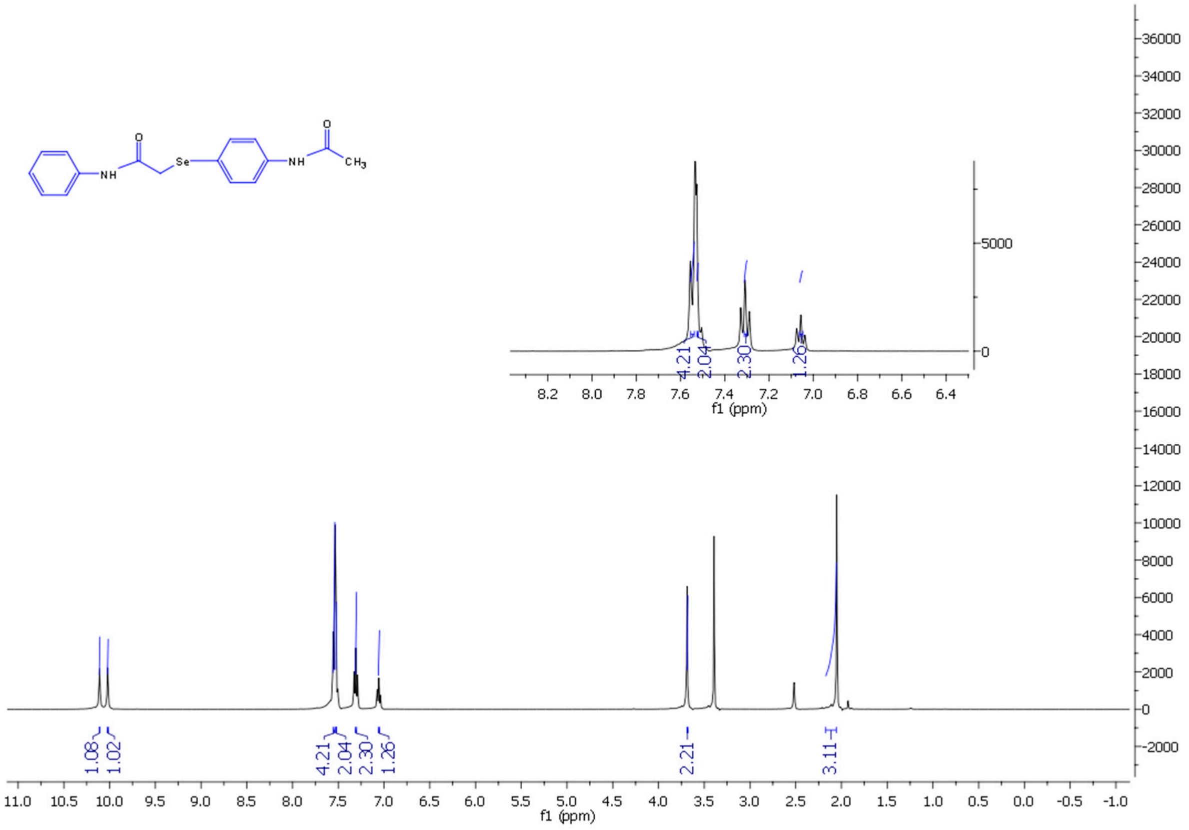

^{1}HNMR chart of compound 18.

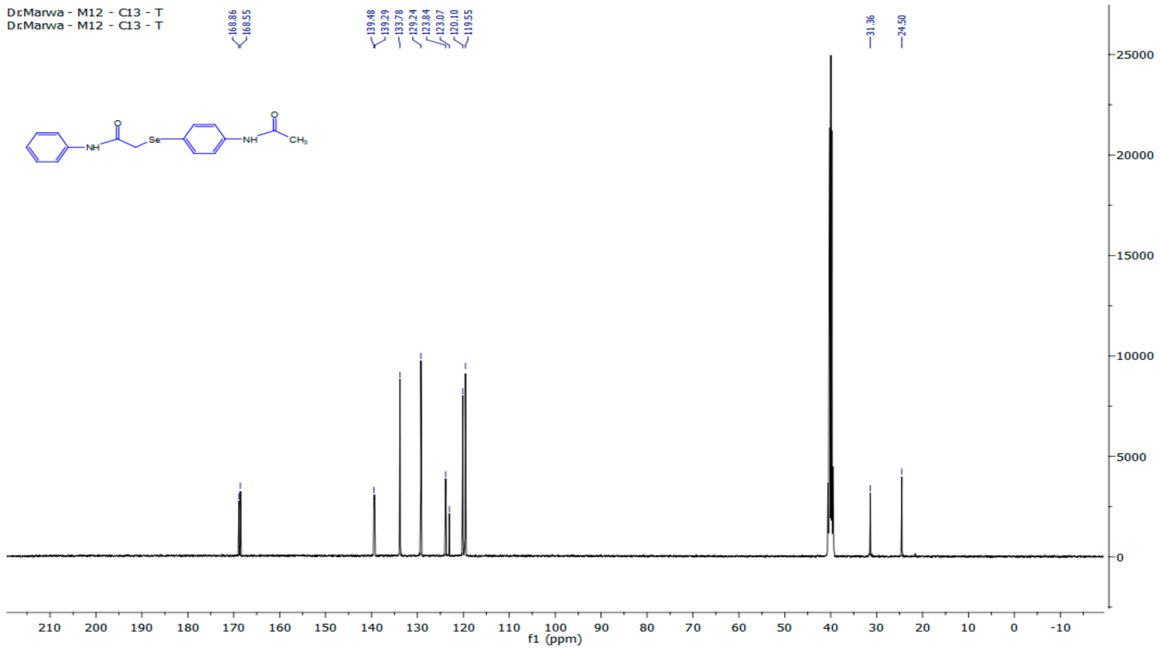

^{13}CNMR chart of compound 18.

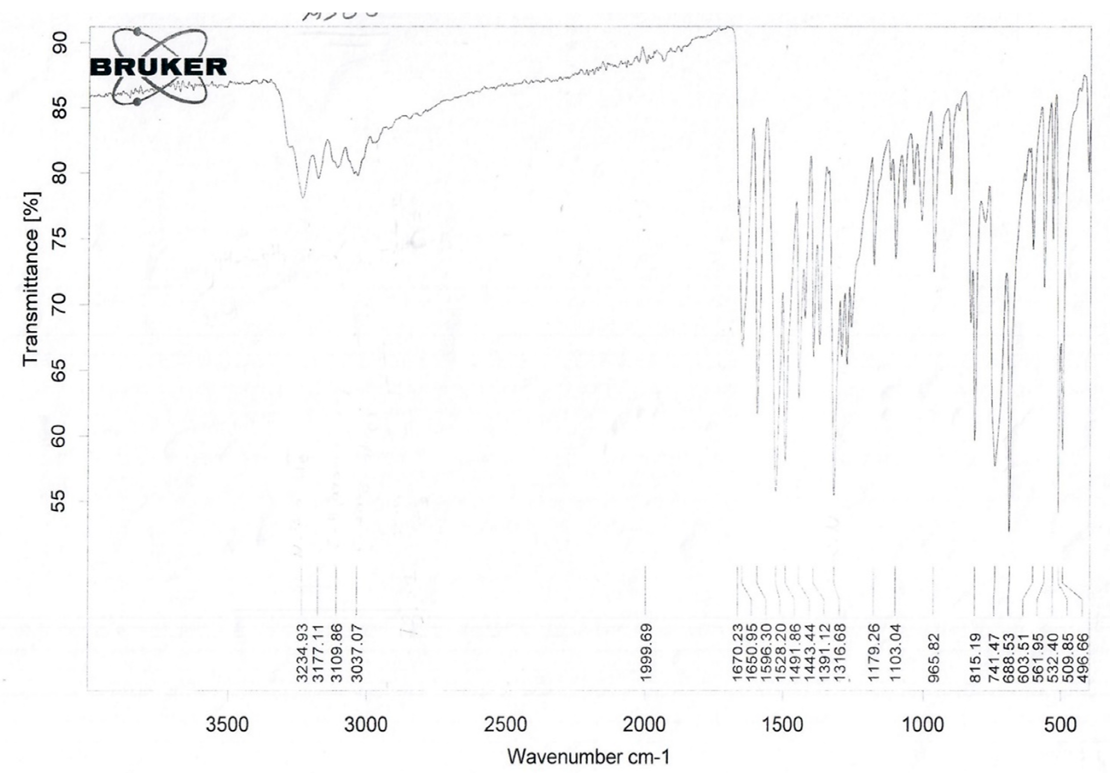

IR chart of compound 18.

09-Jan-07 18:53:57

Cairo University
Micro Analytical Center

DI Analysis
Shimadzu Qp-2010 Plus

Sample Information

Analyzed by	: Dr. Mai Younis
Analyzed	: 09/01/2007 06:47:37
Sample Name	: M12
Sample ID	:
Customer Name	: Dr.Mohamed Soliman - Science - Cairo
Data File	: C:\GCMSsolution\Data\Project1\M12.QGD
Org Data File	: C:\GCMSsolution\Data\Project1\M12.QGD
Method File	: C:\GCMSsolution\Data\Project1\High Temperature Op
Org Method File	: C:\GCMSsolution\Data\Project1\High Temperature Op
Report File	:
Tuning File	: C:\GCMSsolution\System\Tune1_default.qgt
$EndIf$Modified by	: Dr. Mai Younis
Modified	: 09/01/2007 06:52:10

Method

——— Analytical Line 1 ———

IonSourceTemp	:250.00 °C
[MS Table]	
--Group 1 - Event 1--	
Start Time	:0.00min
End Time	:10.00min
ACQ Mode	:Scan
Event Time	:0.50sec
Scan Speed	:1000
Start m/z	:50.00
End m/z	:510.00
Electron Voltage	: 70 eV
Ionization Mode	: EI

C:\GCMSsolution\Data\Project1\M12.QGD

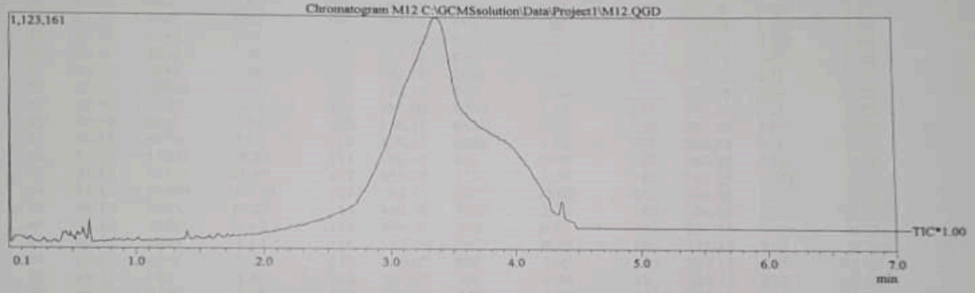

Spectrum

Line#:1 R.Time:3.3(Scan#:401)
MassPeaks:219(Peak Elimination m/z: 363.20)
RawMode:Single 3.3(401) BasePeak:93(95694)
BG Mode:None Group 1 - Event 1

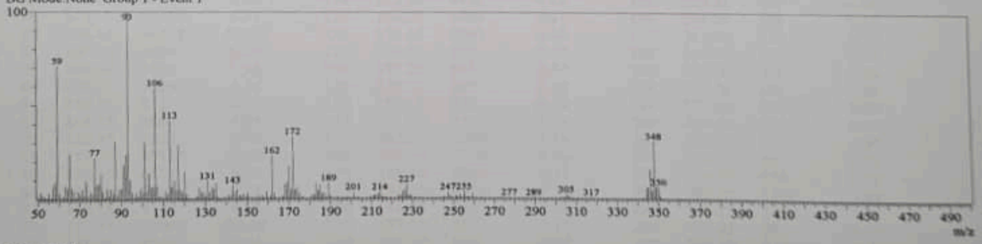

Mass Table
Line#:1 R.Time:3.3(Scan#:401)
MassPeaks:219(Peak Elimination m/z: 363.20)
RawMode:Single 3.3(401) BasePeak:93(95694)
BG Mode:None Group 1 - Event 1

#	m/z	Abs. In	Rel. Int.	#	m/z	Abs. In	Rel. Int.	#	m/z	Abs. In	Rel. Int.
1	50.05	1143	1.19	4	53.05	1208	1.26	7	56.05	917	0.96
2	51.05	3364	3.52	5	54.05	874	0.91	8	57.05	7005	7.32
3	52.05	1968	2.06	6	55.05	3126	3.27	9	58.15	8830	9.23

1 / 3

Mass chart of compound 18.

#	m/z	Abs. In	Rel. Int.	#	m/z	Abs. In	Rel. Int.	#	m/z	Abs. In	Rel. Int.
10	59.05	68029	71.09	79	128.20	3466	3.62	148	199.20	522	0.55
11	60.05	3247	3.39	80	129.15	3340	3.49	149	200.25	380	0.40
12	61.10	1105	1.15	81	130.15	1477	1.54	150	201.20	3238	3.38
13	62.15	1373	1.43	82	131.15	9085	9.49	151	202.15	650	0.68
14	63.05	6992	7.31	83	132.15	3434	3.59	152	203.20	785	0.82
15	64.05	5673	5.93	84	133.20	5804	6.07	153	204.20	358	0.37
16	65.05	22893	23.92	85	134.15	4956	5.18	154	205.20	250	0.26
17	66.05	5814	6.08	86	135.20	7857	8.21	155	208.20	212	0.22
18	67.05	4120	4.31	87	136.15	980	1.02	156	209.10	492	0.51
19	68.05	861	0.90	88	137.20	415	0.43	157	210.10	1074	1.12
20	69.05	3898	4.07	89	138.10	206	0.22	158	211.15	1503	1.57
21	70.10	2206	2.31	90	139.10	994	1.04	159	212.05	1801	1.88
22	71.05	4722	4.93	91	140.15	907	0.95	160	213.15	2193	2.29
23	72.15	1688	1.76	92	141.10	1871	1.96	161	214.10	3126	3.27
24	73.05	9428	9.85	93	142.10	1818	1.90	162	215.10	1017	1.06
25	74.05	1825	1.91	94	143.15	6823	7.13	163	216.20	702	0.73
26	75.10	3526	3.68	95	144.10	2967	3.10	164	217.20	468	0.49
27	76.15	2623	2.74	96	145.10	4441	4.64	165	219.20	463	0.48
28	77.05	21107	22.06	97	146.15	1868	1.95	166	221.20	206	0.22
29	78.10	6929	7.24	98	147.15	1447	1.51	167	223.10	1100	1.15
30	79.10	8017	8.38	99	148.15	2268	2.37	168	224.10	1362	1.42
31	80.10	12634	13.20	100	149.25	1065	1.11	169	225.10	3089	3.23
32	81.10	3770	3.94	101	150.15	3099	3.24	170	226.15	4439	4.64
33	82.15	954	1.00	102	151.10	1185	1.24	171	227.10	6956	7.27
34	83.10	5201	5.44	103	152.15	579	0.61	172	228.05	1562	1.63
35	84.10	1455	1.52	104	153.10	825	0.86	173	229.15	1592	1.66
36	85.05	4810	5.03	105	154.10	510	0.53	174	230.15	367	0.38
37	86.15	2684	2.80	106	155.10	1601	1.67	175	231.10	332	0.35
38	87.05	29573	30.90	107	156.15	806	0.84	176	233.10	236	0.25
39	88.10	2010	2.10	108	157.20	1270	1.33	177	234.10	223	0.23
40	89.10	4921	5.14	109	158.15	648	0.68	178	245.25	546	0.57
41	90.15	5474	5.72	110	159.15	3681	3.85	179	246.35	348	0.36
42	91.10	17527	18.32	111	160.15	617	0.64	180	247.30	3137	3.28
43	92.15	22821	23.85	112	161.15	1505	1.57	181	248.25	1288	1.35
44	93.10	95694	100.00	113	162.15	22071	23.06	182	249.25	360	0.38
45	94.10	9804	10.25	114	163.10	2625	2.74	183	250.10	225	0.24
46	95.10	3286	3.43	115	164.10	302	0.32	184	251.15	900	0.94
47	96.15	1094	1.14	116	165.00	207	0.22	185	252.10	753	0.79
48	97.10	3546	3.71	117	166.05	986	1.03	186	253.15	1652	1.73
49	98.15	1401	1.46	118	167.05	997	1.04	187	254.15	557	0.58
50	99.10	5223	5.46	119	168.05	6969	7.28	188	255.10	3217	3.36
51	100.15	3404	3.56	120	169.05	8420	8.80	189	256.10	644	0.67
52	101.10	28862	30.16	121	170.05	16846	17.60	190	257.15	801	0.84
53	102.15	3490	3.65	122	171.15	4839	5.06	191	258.30	242	0.25
54	103.15	13157	13.75	123	172.05	31912	33.35	192	259.30	2337	2.44
55	104.10	6031	6.30	124	173.10	4436	4.64	193	260.25	457	0.48
56	105.15	6037	6.31	125	174.05	5654	5.91	194	261.20	358	0.37
57	106.15	56728	59.28	126	175.15	2965	3.10	195	263.20	215	0.22
58	107.10	6409	6.70	127	176.15	735	0.77	196	265.20	231	0.24
59	108.10	1154	1.21	128	177.20	831	0.87	197	271.20	231	0.24
60	109.15	1394	1.46	129	179.20	207	0.22	198	273.20	271	0.28
61	110.15	531	0.55	130	180.15	372	0.39	199	277.30	366	0.38
62	111.15	3867	4.04	131	181.10	1566	1.64	200	289.30	334	0.35
63	112.15	2258	2.36	132	182.10	2070	2.16	201	302.35	330	0.34
64	113.15	40125	41.93	133	183.15	7753	8.10	202	303.35	465	0.49
65	114.15	6111	6.39	134	184.10	3969	4.15	203	304.35	456	0.48
66	115.15	9322	9.74	135	185.05	6974	7.29	204	305.35	1808	1.89
67	116.25	4143	4.33	136	186.05	2683	2.80	205	306.35	1250	1.31
68	117.15	27692	28.94	137	187.05	3021	3.16	206	307.35	381	0.40
69	118.15	4540	4.74	138	188.20	873	0.91	207	308.40	214	0.22
70	119.15	3169	3.31	139	189.20	7823	8.18	208	317.40	388	0.41
71	120.15	14054	14.69	140	190.10	1295	1.35	209	342.30	578	0.60
72	121.10	2130	2.23	141	191.10	566	0.59	210	343.25	435	0.45
73	122.10	297	0.31	142	193.25	929	0.97	211	344.25	5545	5.79
74	123.20	450	0.47	143	194.15	297	0.31	212	345.25	6019	6.29
75	124.20	260	0.27	144	195.20	310	0.32	213	346.25	14908	15.58
76	125.25	783	0.82	145	196.00	377	0.39	214	347.25	3995	4.17
77	126.25	1150	1.20	146	197.05	413	0.43	215	348.20	29553	30.88
78	127.20	5855	6.12	147	198.10	591	0.62	216	349.15	6151	6.43

Mass chart of compound 18.

09-Jan-07 18:53:57

#	m/z	Abs. In	Rel. Int.	#	m/z	Abs. In	Rel. Int.	#	m/z	Abs. In	Rel. Int.
217	350.20	5881	6.15	218	351.15	1253	1.31	219	352.20	215	0.22

Mass chart of compound 18.

2. -Chloro-N-(4-((phenylamino)ethyl)selanyl)phenyl)acetamide (19)

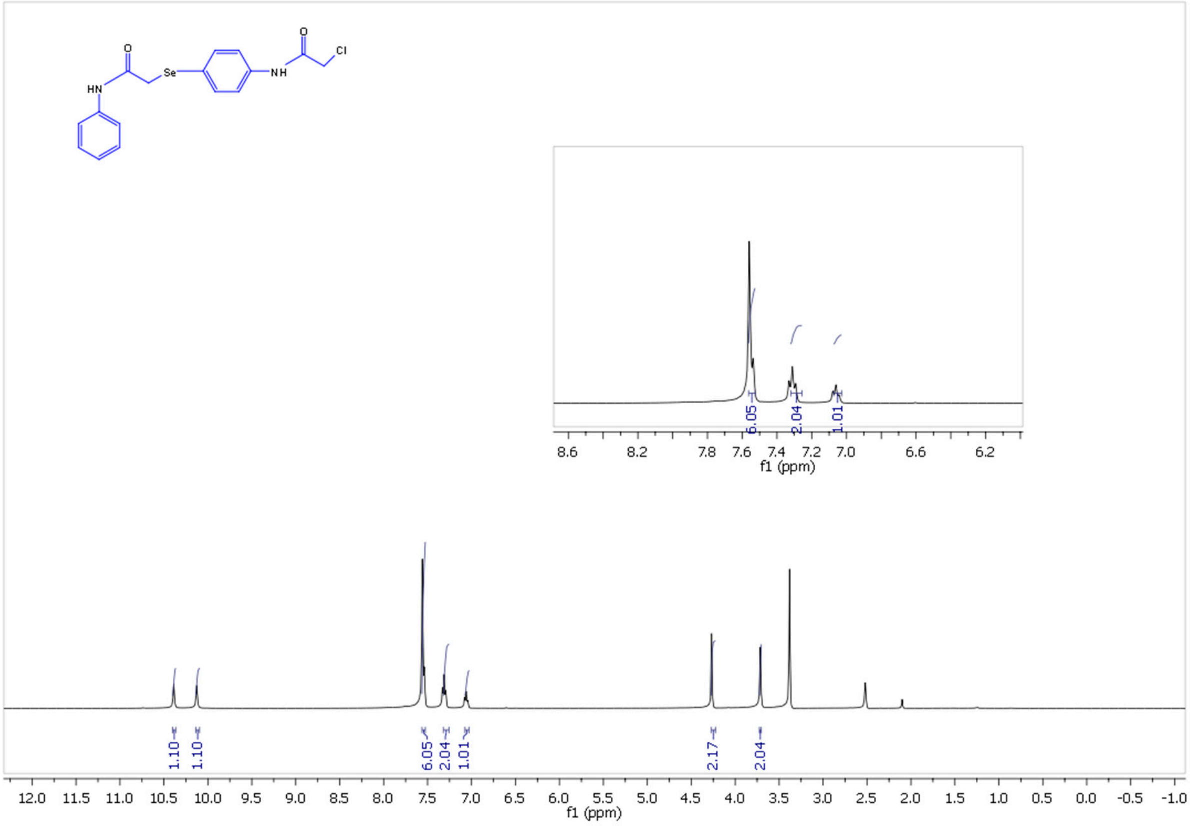

^{1}HNMR chart of compound 19.

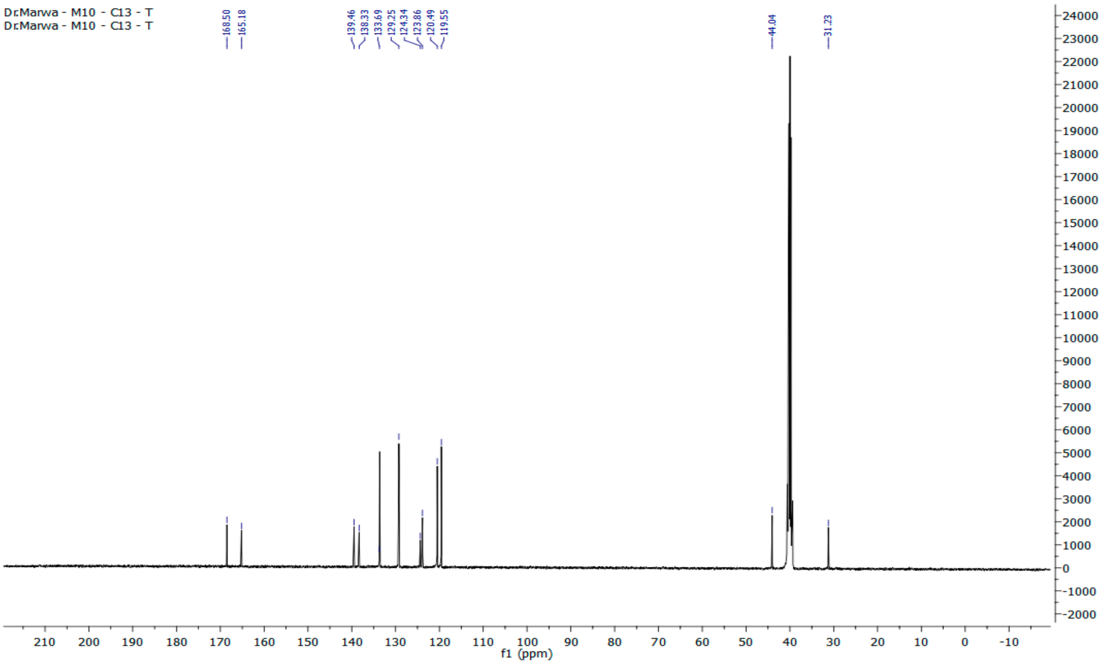

^{13}CNMR chart of compound 19.

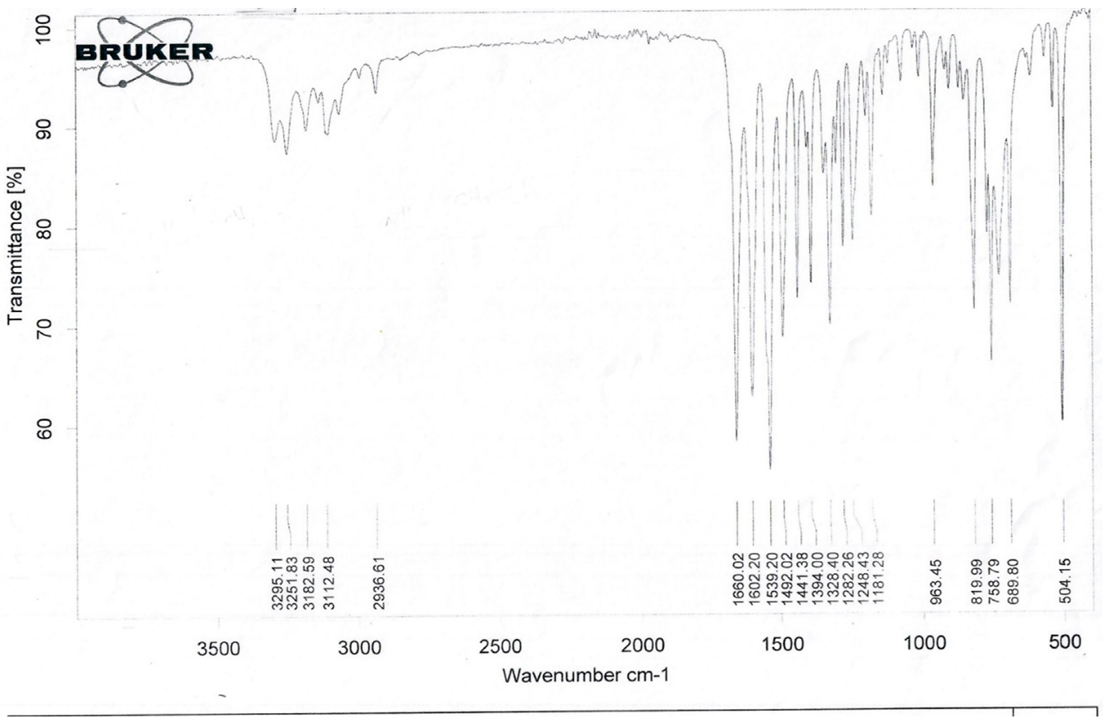

IR chart of compound 19.

09-Jan-07 18:45:55

Cairo University
Micro Analytical Center

DI Analysis
Shimadzu Qp-2010 Plus

Sample Information

Analyzed by	: Dr. Mai Younis
Analyzed	: 09/01/2007 06:39:36
Sample Name	: M10
Sample ID	:
Customer Name	: Dr.Mohamed Soliman - Science - Cairo
Data File	: C:\GCMSsolution\Data\Project1\M10.QGD
Org Data File	: C:\GCMSsolution\Data\Project1\M10.QGD
Method File	: C:\GCMSsolution\Data\Project1\High Temperature Op
Org Method File	: C:\GCMSsolution\Data\Project1\High Temperature Op
Report File	:
Tuning File	: C:\GCMSsolution\System\Tune1_default.qgt
$EndIf$Modified by	: Dr. Mai Younis
Modified	: 09/01/2007 06:44:41

Method

—— Analytical Line 1 ——

IonSourceTemp	:250.00 °C
[MS Table]	
--Group 1 - Event 1--	
Start Time	:0.00min
End Time	:10.00min
ACQ Mode	:Scan
Event Time	:0.50sec
Scan Speed	:1000
Start m/z	:50.00
End m/z	:510.00
Electron Voltage	: 70 eV
Ionization Mode	: EI

C:\GCMSsolution\Data\Project1\M10.QGD

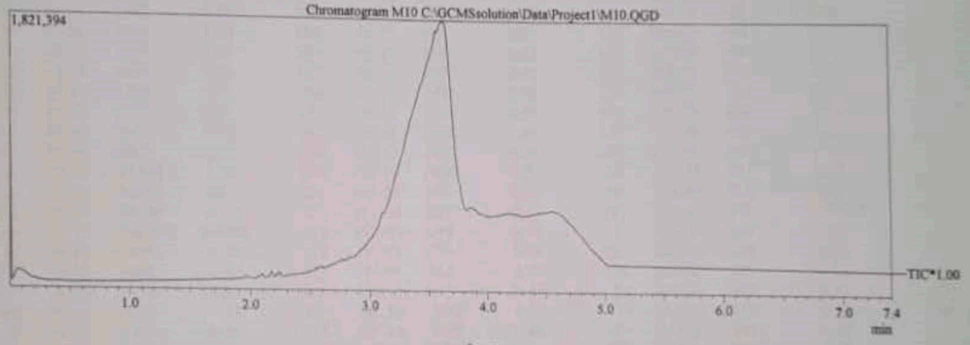

Spectrum

Line#:1 R.Time:3.6(Scan#:436)
MassPeaks:259
RawMode:Single 3.6(436) BasePeak:93(309785)
BG Mode:None Group 1 - Event 1

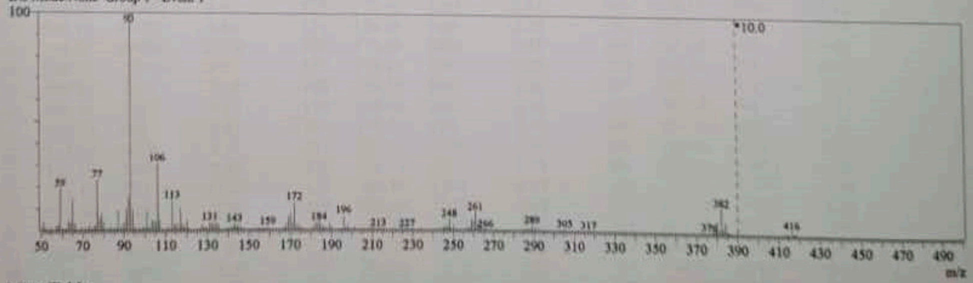

Mass Table
Line#:1 R.Time:3.6(Scan#:436)
MassPeaks:259
RawMode:Single 3.6(436) BasePeak:93(309785)
BG Mode:None Group 1 - Event 1

#	m/z	Abs. In	Rel. Int.	#	m/z	Abs. In	Rel. Int.	#	m/z	Abs. In	Rel. Int.
1	50.05	3334	1.08	4	53.05	1609	0.52	7	56.10	1194	0.39
2	51.00	12022	3.88	5	54.10	1364	0.44	8	57.05	6653	2.15
3	52.00	4254	1.37	6	55.05	3126	1.01	9	58.15	8302	2.68

1 / 3

Mass chart of compound 19.

#	m/z	Abs. In	Rel. Int.
10	59.05	59114	19.08
11	60.05	2990	0.97
12	61.05	1608	0.52
13	62.10	3034	0.98
14	63.05	14297	4.62
15	64.10	11959	3.86
16	65.05	43949	14.19
17	66.10	9985	3.22
18	67.05	4185	1.35
19	68.10	849	0.27
20	69.10	3761	1.21
21	70.10	2010	0.65
22	71.10	4170	1.35
23	72.15	1650	0.53
24	73.10	8271	2.67
25	74.05	2387	0.77
26	75.05	4567	1.47
27	76.05	8200	2.65
28	77.05	71682	23.14
29	78.10	15521	5.01
30	79.05	23897	7.71
31	80.05	12598	4.07
32	81.05	3828	1.24
33	82.10	1092	0.35
34	83.10	5438	1.76
35	84.10	1391	0.45
36	85.10	4644	1.50
37	86.15	2388	0.77
38	87.10	27711	8.95
39	88.10	2431	0.78
40	89.10	6863	2.22
41	90.10	11140	3.60
42	91.10	30806	9.94
43	92.15	48689	15.72
44	93.10	309785	100.00
45	94.10	28588	9.23
46	95.05	4608	1.49
47	96.05	1124	0.36
48	97.10	3633	1.17
49	98.15	1531	0.49
50	99.10	5111	1.65
51	100.15	3424	1.11
52	101.10	27753	8.96
53	102.15	4204	1.36
54	103.15	13393	4.32
55	104.10	14455	4.67
56	105.15	11438	3.69
57	106.15	95284	30.76
58	107.10	15391	4.97
59	108.10	1505	0.49
60	109.15	1215	0.39
61	110.15	662	0.21
62	111.10	4062	1.31
63	112.15	2482	0.80
64	113.15	42218	13.63
65	114.15	7235	2.34
66	115.10	10521	3.40
67	116.25	5856	1.89
68	117.15	31984	10.32
69	118.10	7996	2.58
70	119.10	5562	1.80
71	120.15	13420	4.33
72	121.10	3252	1.05
73	122.30	511	0.16
74	123.30	601	0.19
75	124.15	358	0.12
76	125.15	912	0.29
77	126.15	1363	0.44
78	127.15	7214	2.33
79	128.20	4470	1.44
80	129.15	4012	1.30
81	130.25	2421	0.78
82	131.20	10694	3.45
83	132.15	8834	2.85
84	133.15	8970	2.90
85	134.20	8575	2.77
86	135.20	7785	2.51
87	136.25	768	0.25
88	137.20	540	0.17
89	138.25	468	0.15
90	139.15	1592	0.51
91	140.15	2120	0.68
92	141.15	3421	1.10
93	142.15	3953	1.28
94	143.15	8407	2.71
95	144.15	5971	1.93
96	145.10	5670	1.83
97	146.15	4119	1.33
98	147.15	1789	0.58
99	148.10	652	0.21
100	149.10	342	0.11
101	150.25	304	0.10
102	151.20	940	0.30
103	152.15	984	0.32
104	153.15	1495	0.48
105	154.15	1397	0.45
106	155.15	2431	0.78
107	156.15	1992	0.64
108	157.10	2060	0.66
109	158.15	1192	0.38
110	159.20	3892	1.26
111	160.15	785	0.25
112	161.20	1092	0.35
113	162.20	295	0.10
114	163.20	276	0.09
115	164.20	215	0.07
116	165.15	477	0.15
117	166.10	1633	0.53
118	167.15	2138	0.69
119	168.10	9178	2.96
120	169.10	17590	5.68
121	170.05	23569	7.61
122	171.15	10585	3.42
123	172.05	39077	12.61
124	173.10	7209	2.33
125	174.10	7082	2.29
126	175.20	2806	0.91
127	176.15	789	0.25
128	177.20	894	0.29
129	178.20	354	0.11
130	179.15	370	0.12
131	180.10	1098	0.35
132	181.15	2416	0.78
133	182.10	9703	3.13
134	183.15	10484	3.38
135	184.10	10593	3.42
136	185.05	5904	1.91
137	186.10	5242	1.69
138	187.10	2793	0.90
139	188.15	1465	0.47
140	189.20	7946	2.57
141	190.20	1689	0.55
142	191.20	428	0.14
143	192.20	255	0.08
144	193.20	1017	0.33
145	194.15	702	0.23
146	195.15	990	0.32
147	196.10	20135	6.50
148	197.10	2802	0.90
149	198.10	7782	2.51
150	199.10	1244	0.40
151	200.15	710	0.23
152	201.20	3329	1.07
153	202.15	750	0.24
154	203.15	817	0.26
155	204.15	672	0.22
156	205.10	388	0.13
157	206.10	265	0.09
158	207.10	306	0.10
159	208.10	388	0.13
160	209.10	1094	0.35
161	210.05	812	0.26
162	211.05	1053	0.34
163	212.05	1204	0.39
164	213.10	1490	0.48
165	214.00	745	0.24
166	215.15	520	0.17
167	216.10	266	0.09
168	217.10	361	0.12
169	219.10	396	0.13
170	224.10	202	0.07
171	225.10	263	0.08
172	226.05	345	0.11
173	227.20	678	0.22
174	228.15	363	0.12
175	228.95	314	0.10
176	229.95	405	0.13
177	231.40	468	0.15
178	232.35	300	0.10
179	233.35	531	0.17
180	234.40	255	0.08
181	235.40	345	0.11
182	236.40	247	0.08
183	237.40	222	0.07
184	239.40	297	0.10
185	242.20	345	0.11
186	243.15	319	0.10
187	244.10	2414	0.78
188	245.15	3007	0.97
189	246.10	6802	2.20
190	247.15	5722	1.85
191	248.10	15470	4.99
192	249.05	2213	0.71
193	250.10	6556	2.12
194	251.05	1064	0.34
195	252.10	937	0.30
196	254.10	273	0.09
197	255.30	478	0.15
198	256.15	381	0.12
199	257.10	3887	1.25
200	258.10	4492	1.45
201	259.10	14574	4.70
202	260.15	13616	4.40
203	261.10	25454	8.22
204	262.15	8992	2.90
205	263.10	11991	3.87
206	264.05	2914	0.94
207	265.10	2270	0.73
208	266.20	649	0.21
209	267.20	326	0.11
210	271.30	567	0.18
211	273.30	462	0.15
212	274.30	222	0.07
213	275.30	250	0.08
214	276.30	260	0.08
215	277.35	521	0.17
216	282.30	316	0.10

2 / 3

Mass chart of compound 19.

09-Jan-07 18:45:55

#	m/z	Abs. In	Rel. Int.	#	m/z	Abs. In	Rel. Int.	#	m/z	Abs. In	Rel. Int.
217	283.30	210	0.07	232	298.10	201	0.06	247	381.25	5978	1.93
218	284.10	233	0.08	233	301.25	419	0.14	248	382.20	35959	11.61
219	285.10	1406	0.45	234	302.10	418	0.13	249	383.15	7607	2.46
220	286.15	1499	0.48	235	303.15	878	0.28	250	384.20	16228	5.24
221	287.15	3635	1.17	236	304.25	291	0.09	251	385.10	3350	1.08
222	288.15	1400	0.45	237	305.30	2286	0.74	252	386.20	2276	0.73
223	289.10	7086	2.29	238	306.25	705	0.23	253	387.30	481	0.16
224	290.10	1409	0.45	239	307.20	452	0.15	254	388.30	223	0.07
225	291.10	3271	1.06	240	317.20	268	0.09	255	413.30	215	0.07
226	292.10	657	0.21	241	323.20	252	0.08	256	414.30	282	0.09
227	293.20	716	0.23	242	376.30	522	0.17	257	416.20	735	0.24
228	294.20	228	0.07	243	377.20	271	0.09	258	417.20	204	0.07
229	295.15	477	0.15	244	378.20	5782	1.87	259	418.15	432	0.14
230	296.10	263	0.08	245	379.25	6464	2.09				
231	297.10	268	0.09	246	380.20	17572	5.67				

Mass chart of compound 19.

2. -((4-(((Dimethylamino)methylene)amino)phenyl)selanyl)-N-phenylacetamide (20)

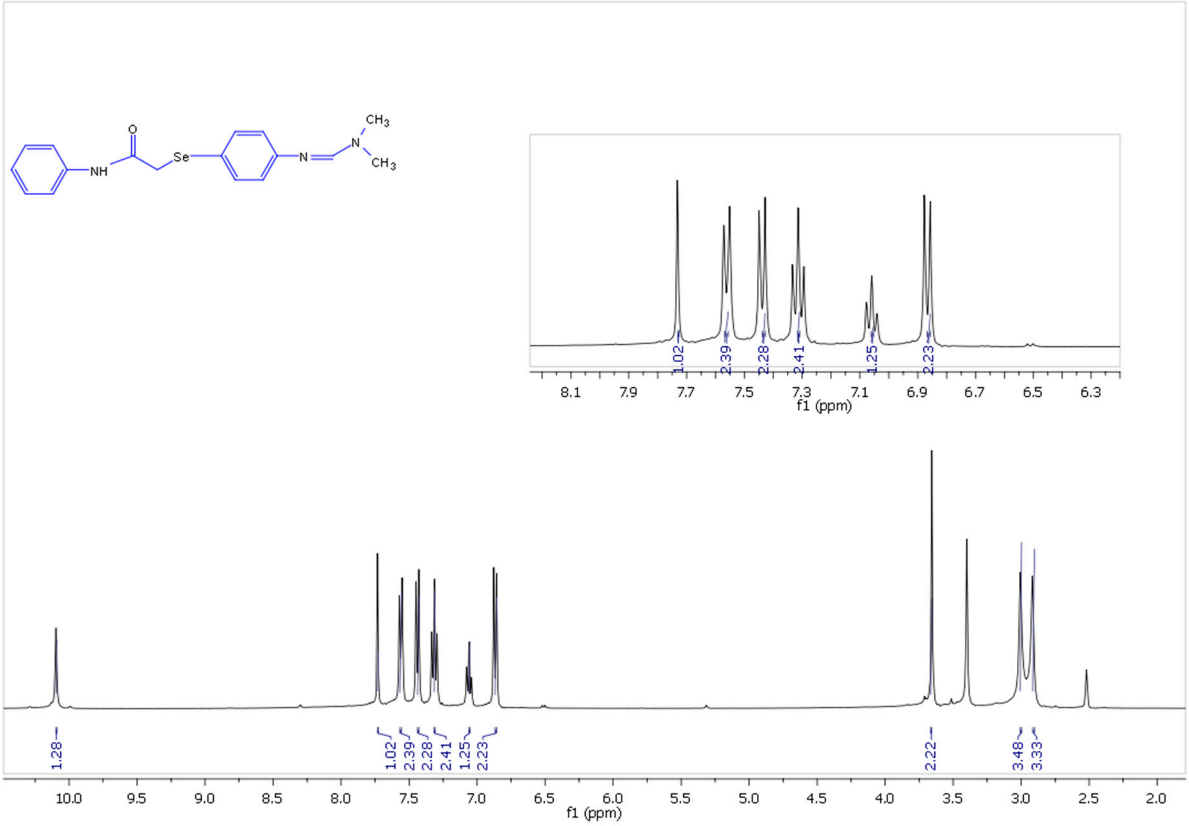

^{1}HNMR chart of compound 20.

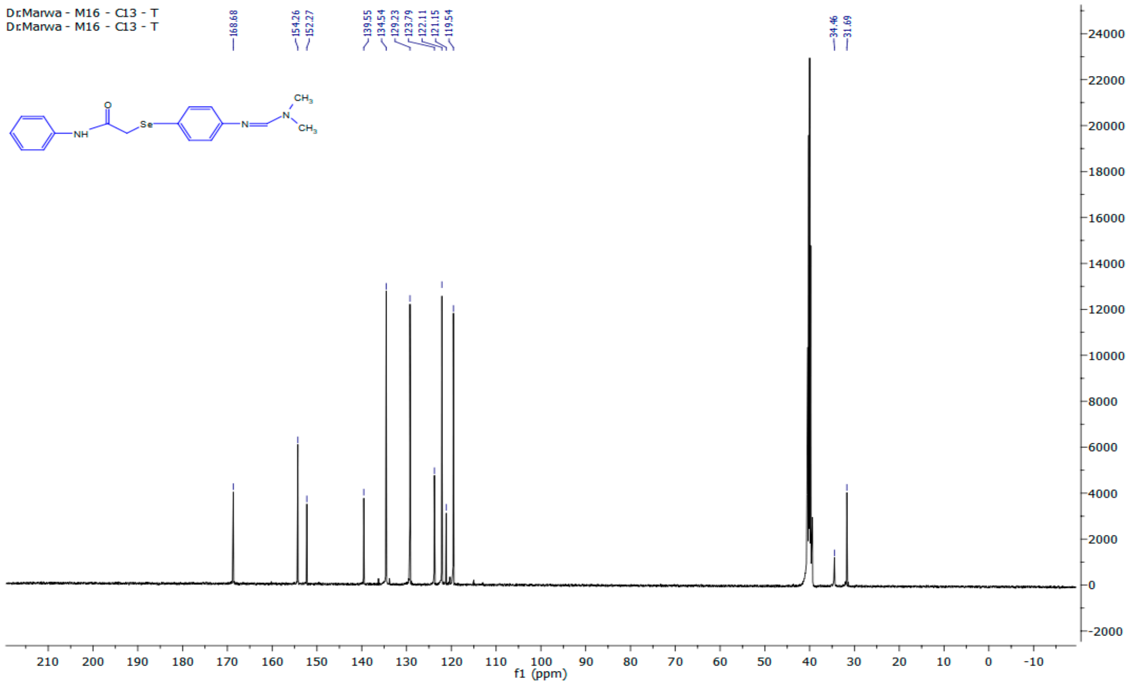

^{13}CNMR chart of compound 20.

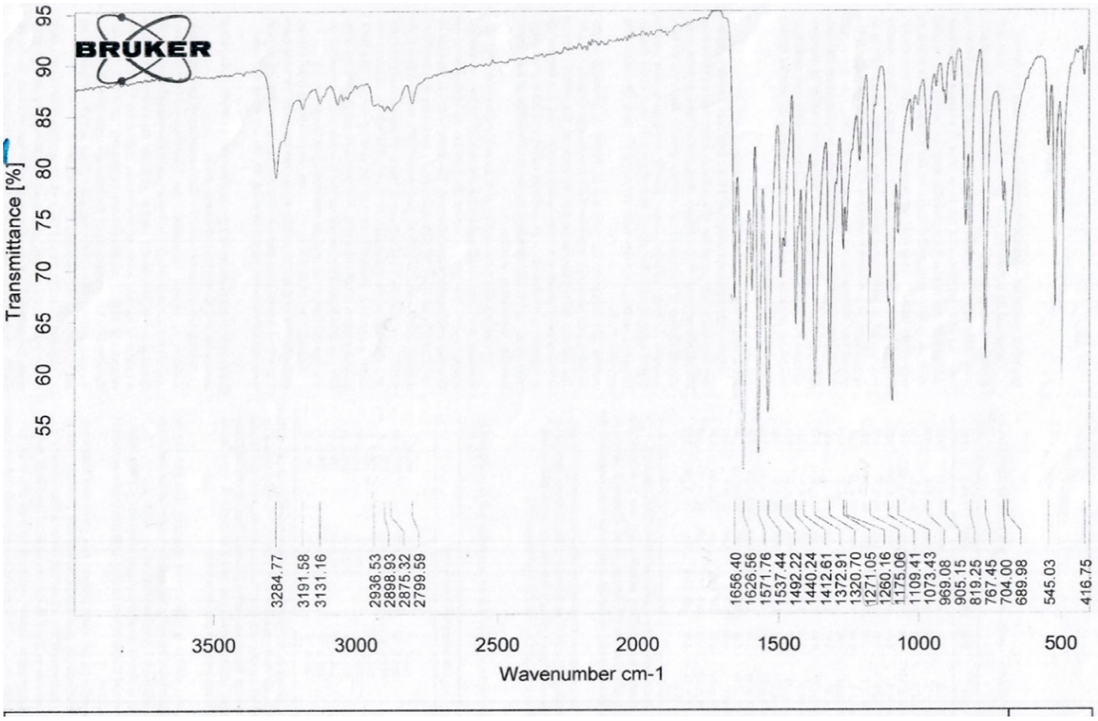

IR chart of compound 20.

09-Jan-07 19:21:17

Cairo University
Micro Analytical Center

DI Analysis
Shimadzu Qp-2010 Plus

Sample Information

Analyzed by	: Dr. Mai Younis
Analyzed	: 09/01/2007 07:13:44 م
Sample Name	: M16
Sample ID	:
Customer Name	: Dr.Mohamed Soliman - Science - Cairo
Data File	: C:\GCMSsolution\Data\Project1\M16.QGD
Org Data File	: C:\GCMSsolution\Data\Project1\M16.QGD
Method File	: C:\GCMSsolution\Data\Project1\High Temperature Op
Org Method File	: C:\GCMSsolution\Data\Project1\High Temperature Op
Report File	:
Tuning File	: C:\GCMSsolution\System\Tune1_default.qgt
$EndIf$Modified by	: Dr. Mai Younis
Modified	: 09/01/2007 07:17:56 م

Method

——— Analytical Line 1 ———

IonSourceTemp	:250.00 °C
[MS Table]	
--Group 1 - Event 1--	
Start Time	:0.00min
End Time	:10.00min
ACQ Mode	:Scan
Event Time	:0.50sec
Scan Speed	:1000
Start m/z	:50.00
End m/z	:510.00
Electron Voltage	: 70 eV
Ionization Mode	: EI

C:\GCMSsolution\Data\Project1\M16.QGD

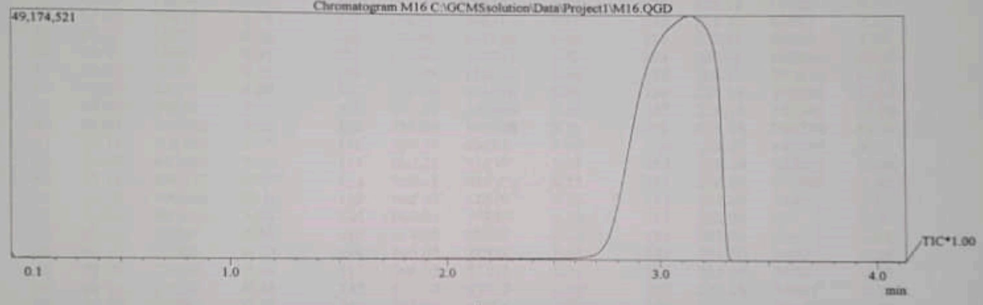

Spectrum

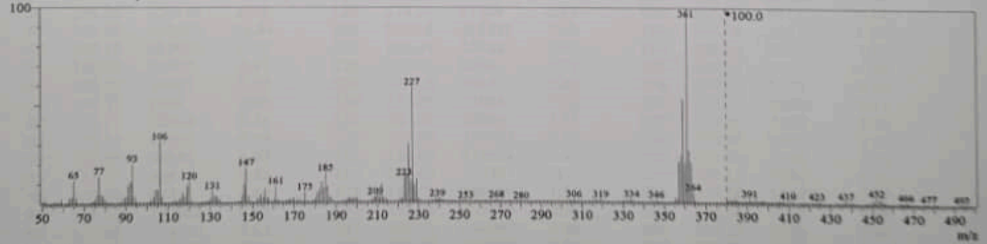

Mass Table
Line#:1 R.Time:3.0(Scan#:362)
MassPeaks:428
RawMode:Single 3.0(362) BasePeak:361(5015363)
BG Mode:None Group 1 - Event 1

#	m/z	Abs. In	Rel. Int.	#	m/z	Abs. In	Rel. Int.	#	m/z	Abs. In	Rel. Int.
1	50.00	45807	0.91	4	53.00	13918	0.28	7	56.05	44911	0.90
2	51.00	89281	1.78	5	54.05	16543	0.33	8	57.05	55536	1.11
3	52.00	38135	0.76	6	55.05	22229	0.44	9	58.05	20929	0.42

1 / 4

Mass chart of compound 20.

#	m/z	Abs. In	Rel. Int.
10	59.05	99497	1.98
11	60.00	8874	0.18
12	61.05	9033	0.18
13	62.05	33465	0.67
14	63.05	148541	2.96
15	64.05	154491	3.08
16	65.00	594753	11.86
17	66.00	136188	2.72
18	67.00	34662	0.69
19	68.05	12871	0.26
20	69.05	32376	0.65
21	70.10	13036	0.26
22	71.05	24826	0.49
23	72.05	9302	0.19
24	73.05	30849	0.62
25	74.05	49838	0.99
26	75.05	107700	2.15
27	76.05	223920	4.46
28	77.05	693813	13.83
29	78.05	211887	4.22
30	79.05	190570	3.80
31	80.05	93455	1.86
32	81.05	46849	0.93
33	82.10	17646	0.35
34	83.05	35012	0.70
35	84.05	13273	0.26
36	85.10	15681	0.31
37	86.05	7679	0.15
38	87.05	42253	0.84
39	88.15	28830	0.57
40	89.05	146432	2.92
41	90.15	178182	3.55
42	91.10	455826	9.09
43	92.15	550275	10.97
44	93.05	100866	20.11
45	94.05	182219	3.63
46	95.00	44287	0.88
47	96.10	17561	0.35
48	97.10	24356	0.49
49	98.15	17037	0.34
50	99.05	14864	0.30
51	100.15	11717	0.23
52	101.15	47235	0.94
53	102.15	65109	1.30
54	103.15	167673	3.34
55	104.10	356937	7.12
56	105.15	330155	6.58
57	106.10	157689	31.44
58	107.10	162070	3.23
59	108.05	20707	0.41
60	109.10	18487	0.37
61	110.15	8948	0.18
62	111.15	18063	0.36
63	112.15	19385	0.39
64	113.10	80316	1.60
65	114.15	42878	0.85
66	115.05	84145	1.68
67	116.15	147865	2.95
68	117.05	251282	5.01
69	118.15	185624	3.70
70	119.15	446781	8.91
71	120.05	559245	11.15
72	121.10	63710	1.27
73	122.10	23422	0.47
74	123.10	10271	0.20
75	124.15	7157	0.14
76	125.15	11111	0.22
77	126.10	20262	0.40
78	127.10	31345	0.62
79	128.05	43505	0.87
80	129.15	43785	0.87
81	130.15	119980	2.39
82	131.15	304557	6.07
83	132.15	190052	3.79
84	133.15	169311	3.38
85	134.15	104114	2.08
86	135.15	37430	0.75
87	136.10	15561	0.31
88	137.20	7714	0.15
89	138.15	9184	0.18
90	139.15	13239	0.26
91	140.15	13883	0.28
92	141.15	22865	0.46
93	142.15	20511	0.41
94	143.15	34130	0.68
95	144.25	52499	1.05
96	145.25	201359	4.01
97	146.20	490233	9.77
98	147.15	894287	17.83
99	148.15	169110	3.37
100	149.15	50215	1.00
101	150.05	13265	0.26
102	151.05	18634	0.37
103	152.05	76831	1.53
104	153.05	101714	2.03
105	154.00	191201	3.81
106	155.05	118534	2.36
107	156.00	341970	6.82
108	157.00	123164	2.46
109	158.00	109708	2.19
110	159.15	49173	0.98
111	160.25	71679	1.43
112	161.15	417302	8.32
113	162.15	61338	1.22
114	163.05	17909	0.36
115	164.05	42353	0.84
116	165.05	27526	0.55
117	166.05	37181	0.74
118	167.05	65012	1.30
119	168.05	60608	1.21
120	169.05	107748	2.15
121	170.00	93930	1.87
122	171.05	44795	0.89
123	172.05	90302	1.80
124	173.15	35783	0.71
125	174.25	41789	0.83
126	175.15	255495	5.09
127	176.15	33728	0.67
128	177.05	8646	0.17
129	178.05	14057	0.28
130	179.05	51084	1.02
131	180.05	92640	1.85
132	181.05	238763	4.76
133	182.05	314424	6.27
134	183.05	524391	10.46
135	184.05	366690	7.31
136	185.05	735891	14.67
137	186.05	401093	8.00
138	187.00	160268	3.20
139	188.05	101262	2.02
140	189.10	22097	0.44
141	190.05	6502	0.13
142	191.15	5444	0.11
143	192.05	14833	0.30
144	193.15	21397	0.43
145	194.05	42113	0.84
146	195.15	45530	0.91
147	196.05	94181	1.88
148	197.10	82913	1.65
149	198.05	89474	1.78
150	199.05	99997	1.99
151	200.05	86027	1.72
152	201.05	28580	0.57
153	202.05	15556	0.31
154	203.15	3393	0.07
155	204.15	6257	0.12
156	205.15	4571	0.09
157	206.10	14186	0.28
158	207.15	24866	0.50
159	208.10	108055	2.15
160	209.10	125135	2.50
161	210.05	240845	4.80
162	211.05	118740	2.37
163	212.05	441850	8.81
164	212.95	82885	1.65
165	214.05	85602	1.71
166	215.05	14478	0.29
167	216.05	2333	0.05
168	217.15	1977	0.04
169	218.15	1652	0.03
170	219.15	3746	0.07
171	220.15	11985	0.24
172	221.10	64873	1.29
173	222.15	95023	1.89
174	223.10	608946	12.14
175	224.15	702419	14.01
176	225.10	148188	29.55
177	226.15	551369	10.99
178	227.10	294335	58.69
179	228.05	447109	8.91
180	229.10	554807	11.06
181	230.05	70100	1.40
182	231.05	5640	0.11
183	232.05	967	0.02
184	233.25	1941	0.04
185	234.20	2450	0.05
186	235.25	5458	0.11
187	236.20	14065	0.28
188	237.20	22108	0.44
189	238.20	56311	1.12
190	239.20	61671	1.23
191	240.15	59264	1.18
192	241.10	30422	0.61
193	242.10	21420	0.43
194	243.10	8774	0.17
195	244.15	4217	0.08
196	245.15	3337	0.07
197	246.15	2891	0.06
198	247.25	9773	0.19
199	248.15	5289	0.11
200	249.10	4170	0.08
201	250.15	4587	0.09
202	251.10	7658	0.15
203	252.15	5358	0.11
204	253.10	13489	0.27
205	254.05	2797	0.06
206	255.15	4486	0.09
207	256.20	2332	0.05
208	257.25	2717	0.05
209	258.25	2055	0.04
210	259.25	7397	0.15
211	260.20	2879	0.06
212	261.15	3156	0.06
213	262.15	4750	0.09
214	263.15	4143	0.08
215	264.15	9049	0.18
216	265.15	10956	0.22

2 / 4

Mass chart of compound 20.

#	m/z	Abs. In	Rel. Int.
10	59.05	99497	1.98
11	60.00	8874	0.18
12	61.05	9033	0.18
13	62.05	33465	0.67
14	63.05	148541	2.96
15	64.05	154491	3.08
16	65.00	594753	11.86
17	66.00	136188	2.72
18	67.00	34662	0.69
19	68.05	12871	0.26
20	69.05	32376	0.65
21	70.10	13036	0.26
22	71.05	24826	0.49
23	72.05	9302	0.19
24	73.05	30849	0.62
25	74.05	49838	0.99
26	75.05	107700	2.15
27	76.05	223920	4.46
28	77.05	693813	13.83
29	78.05	211887	4.22
30	79.05	190570	3.80
31	80.05	93455	1.86
32	81.05	46849	0.93
33	82.10	17646	0.35
34	83.05	35012	0.70
35	84.05	13273	0.26
36	85.10	15681	0.31
37	86.05	7679	0.15
38	87.05	42253	0.84
39	88.15	28830	0.57
40	89.05	146432	2.92
41	90.15	178182	3.55
42	91.10	455826	9.09
43	92.15	550275	10.97
44	93.05	100866	20.11
45	94.05	182219	3.63
46	95.00	44287	0.88
47	96.10	17561	0.35
48	97.10	24356	0.49
49	98.15	17037	0.34
50	99.05	14864	0.30
51	100.15	11717	0.23
52	101.15	47235	0.94
53	102.15	65109	1.30
54	103.15	167673	3.34
55	104.10	356937	7.12
56	105.15	330155	6.58
57	106.10	157689	31.44
58	107.10	162070	3.23
59	108.05	20707	0.41
60	109.10	18487	0.37
61	110.15	8948	0.18
62	111.15	18063	0.36
63	112.15	19385	0.39
64	113.10	80316	1.60
65	114.15	42878	0.85
66	115.05	84145	1.68
67	116.15	147865	2.95
68	117.05	251282	5.01
69	118.15	185624	3.70
70	119.15	446781	8.91
71	120.05	559245	11.15
72	121.10	63710	1.27
73	122.10	23422	0.47
74	123.10	10271	0.20
75	124.15	7157	0.14
76	125.15	11111	0.22
77	126.10	20262	0.40
78	127.10	31345	0.62

#	m/z	Abs. In	Rel. Int.
79	128.05	43505	0.87
80	129.15	43785	0.87
81	130.15	119980	2.39
82	131.15	304557	6.07
83	132.15	190052	3.79
84	133.15	169311	3.38
85	134.15	104114	2.08
86	135.15	37430	0.75
87	136.10	15561	0.31
88	137.20	7714	0.15
89	138.15	9184	0.18
90	139.15	13239	0.26
91	140.15	13883	0.28
92	141.15	22865	0.46
93	142.15	20511	0.41
94	143.15	34130	0.68
95	144.25	52499	1.05
96	145.25	201359	4.01
97	146.20	490233	9.77
98	147.15	894287	17.83
99	148.15	169110	3.37
100	149.15	50215	1.00
101	150.05	13265	0.26
102	151.05	18634	0.37
103	152.05	76831	1.53
104	153.05	101714	2.03
105	154.00	191201	3.81
106	155.05	118534	2.36
107	156.00	341970	6.82
108	157.00	123164	2.46
109	158.00	109708	2.19
110	159.15	49173	0.98
111	160.25	71679	1.43
112	161.15	417302	8.32
113	162.15	61338	1.22
114	163.05	17909	0.36
115	164.05	42353	0.84
116	165.05	27526	0.55
117	166.05	37181	0.74
118	167.05	65012	1.30
119	168.05	60608	1.21
120	169.05	107748	2.15
121	170.00	93930	1.87
122	171.05	44795	0.89
123	172.05	90302	1.80
124	173.15	35783	0.71
125	174.25	41789	0.83
126	175.15	255495	5.09
127	176.15	33728	0.67
128	177.05	8646	0.17
129	178.05	14057	0.28
130	179.05	51084	1.02
131	180.05	92640	1.85
132	181.05	238763	4.76
133	182.05	314424	6.27
134	183.05	524391	10.46
135	184.05	366690	7.31
136	185.05	735891	14.67
137	186.05	401093	8.00
138	187.00	160268	3.20
139	188.05	101262	2.02
140	189.10	22097	0.44
141	190.05	6502	0.13
142	191.15	5444	0.11
143	192.05	14833	0.30
144	193.15	21397	0.43
145	194.05	42113	0.84
146	195.15	45530	0.91
147	196.05	94181	1.88

#	m/z	Abs. In	Rel. Int.
148	197.10	82913	1.65
149	198.05	89474	1.78
150	199.05	99997	1.99
151	200.05	86027	1.72
152	201.05	28580	0.57
153	202.05	15556	0.31
154	203.15	3393	0.07
155	204.15	6257	0.12
156	205.15	4571	0.09
157	206.10	14186	0.28
158	207.15	24866	0.50
159	208.10	108055	2.15
160	209.10	125135	2.50
161	210.05	240845	4.80
162	211.05	118740	2.37
163	212.05	441850	8.81
164	212.95	82885	1.65
165	214.05	85602	1.71
166	215.05	14478	0.29
167	216.05	2333	0.05
168	217.15	1977	0.04
169	218.15	1652	0.03
170	219.15	3746	0.07
171	220.15	11985	0.24
172	221.10	64873	1.29
173	222.15	95023	1.89
174	223.10	608946	12.14
175	224.15	702419	14.01
176	225.10	148188	29.55
177	226.15	551369	10.99
178	227.10	294335	58.69
179	228.05	447109	8.91
180	229.10	554807	11.06
181	230.05	70100	1.40
182	231.05	5640	0.11
183	232.05	967	0.02
184	233.25	1941	0.04
185	234.20	2450	0.05
186	235.25	5458	0.11
187	236.20	14065	0.28
188	237.20	22108	0.44
189	238.20	56311	1.12
190	239.20	61671	1.23
191	240.15	59264	1.18
192	241.10	30422	0.61
193	242.10	21420	0.43
194	243.10	8774	0.17
195	244.15	4217	0.08
196	245.15	3337	0.07
197	246.15	2891	0.06
198	247.25	9773	0.19
199	248.15	5289	0.11
200	249.10	4170	0.08
201	250.15	4587	0.09
202	251.10	7658	0.15
203	252.15	5358	0.11
204	253.10	13489	0.27
205	254.05	2797	0.06
206	255.15	4486	0.09
207	256.20	2332	0.05
208	257.25	2717	0.05
209	258.25	2055	0.04
210	259.25	7397	0.15
211	260.20	2879	0.06
212	261.15	3156	0.06
213	262.15	4750	0.09
214	263.15	4143	0.08
215	264.15	9049	0.18
216	265.15	10956	0.22

2 / 4

Mass chart of compound 20.

09-Jan-07 19:21:17

#	m/z	Abs. In	Rel. Int.	#	m/z	Abs. In	Rel. Int.	#	m/z	Abs. In	Rel. Int.
424	480.40	270	0.01	426	493.40	244	0.00	428	495.40	234	0.00
425	481.40	276	0.01	427	494.40	233	0.00				

Mass chart of compound 20.

2. -((4-((2-Hydroxybenzylidene)amino)phenyl)selanyl)-N-phenylacetamide (21)

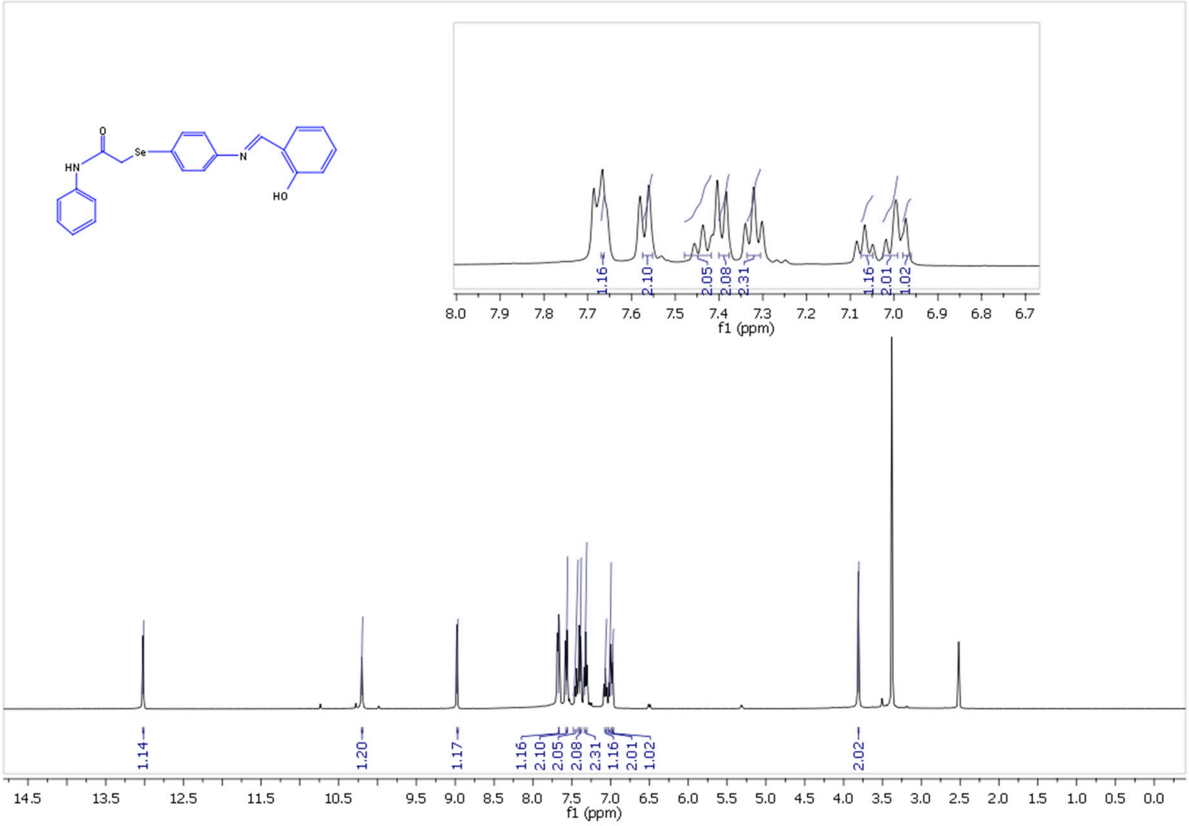

^{1}HNMR chart of compound 21.

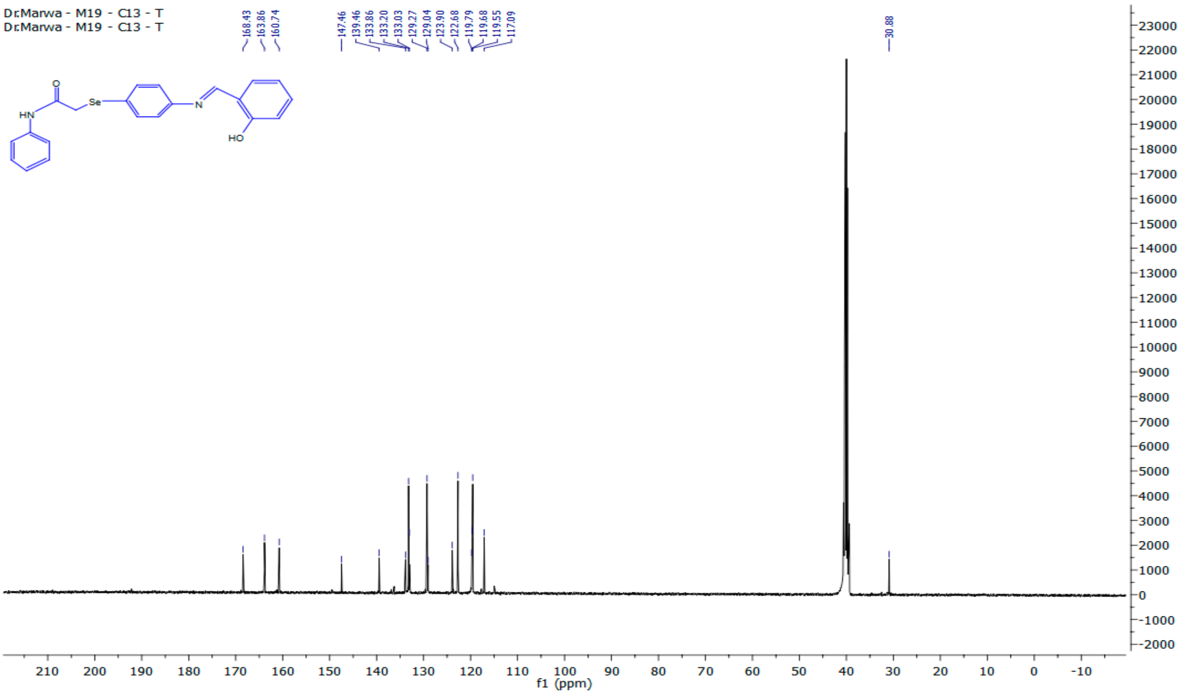

^{13}CNMR chart of compound 21.

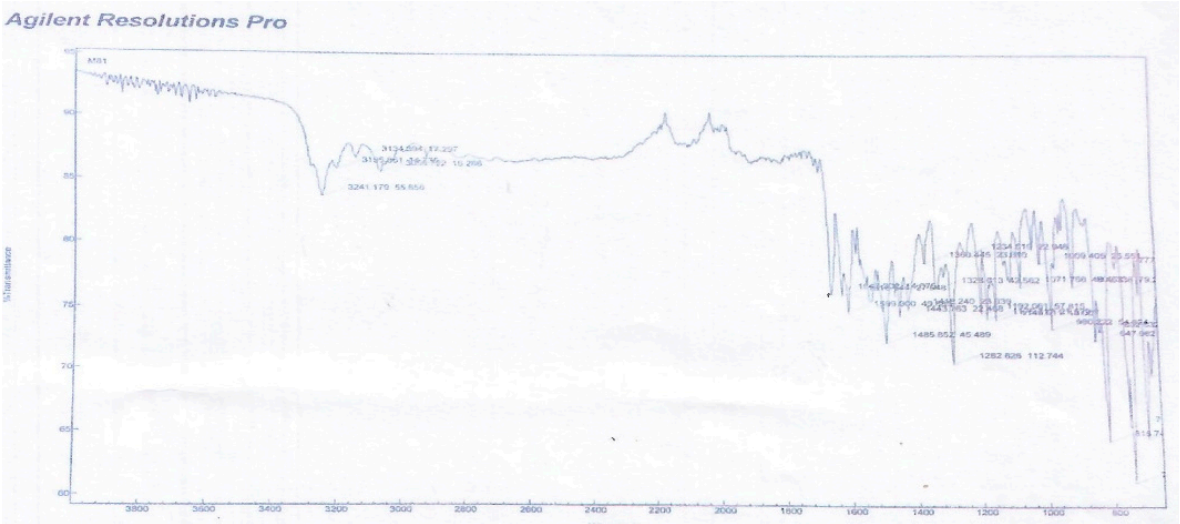

IR chart of compound 21.

Cairo University
Micro Analytical Center

DI Analysis
Shimadzu Qp-2010 Plus

Sample Information		Method	
Analyzed by	: Dr. Mai Younis		
Analyzed	: 03/01/2007 08:19:07 م	—— Analytical Line 1 ——	
Sample Name	: M19	IonSourceTemp	:250.00 °C
Sample ID	:	[MS Table]	
Customer Name	: Dr.Mohamed Soliman - Science - Cairo	--Group 1 - Event 1--	
Data File	: C:\GCMSsolution\Data\Project1\M19.QGD	Start Time	:0.00min
Org Data File	: C:\GCMSsolution\Data\Project1\M19.QGD	End Time	:10.00min
Method File	: C:\GCMSsolution\Data\Project1\High Temperature Op	ACQ Mode	:Scan
Org Method File	: C:\GCMSsolution\Data\Project1\High Temperature Op	Event Time	:0.50sec
Report File	:	Scan Speed	:1000
Tuning File	: C:\GCMSsolution\System\Tune1_default.qgt	Start m/z	:50.00
$EndIf$Modified by	: Dr. Mai Younis	End m/z	:510.00
Modified	: 03/01/2007 08:24:23 م		
		Electron Voltage	: 70 eV
		Ionization Mode	: EI

C:\GCMSsolution\Data\Project1\M19.QGD

Chromatogram M19 C:\GCMSsolution\Data\Project1\M19.QGD

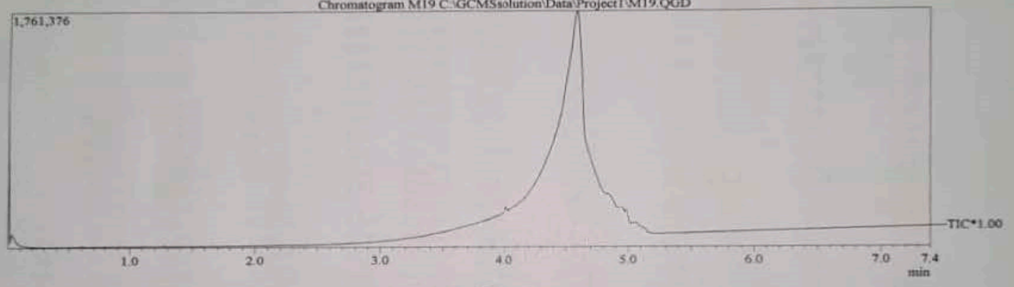

Spectrum

Line#:1 R.Time:4.6(Scan#:550)
MassPeaks:249
RawMode:Single 4.6(550) BasePeak:93(154162)
BG Mode:None Group 1 - Event 1

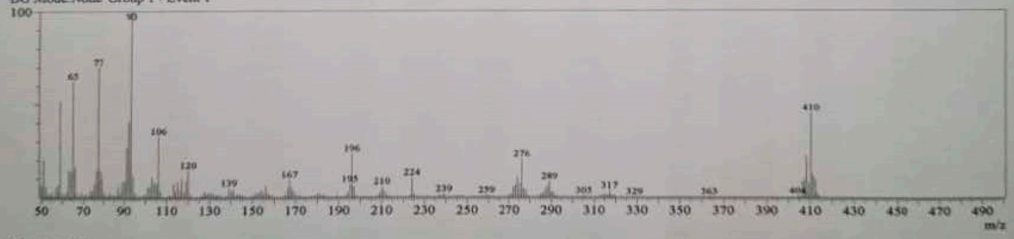

Mass Table
Line#:1 R.Time:4.6(Scan#:550)
MassPeaks:249
RawMode:Single 4.6(550) BasePeak:93(154162)
BG Mode:None Group 1 - Event 1

#	m/z	Abs. In	Rel. Int.	#	m/z	Abs. In	Rel. Int.	#	m/z	Abs. In	Rel. Int.
1	50.05	9941	6.45	4	53.00	3146	2.04	7	56.05	1300	0.84
2	51.00	31618	20.51	5	54.05	2070	1.34	8	57.05	8007	5.19
3	52.05	9015	5.85	6	55.00	4350	2.82	9	58.05	10029	6.51

1 / 3

Mass chart of compound 21.

#	m/z	Abs. In	Rel. Int.
10	59.00	80090	51.95
11	60.00	3423	2.22
12	61.05	1523	0.99
13	62.05	4833	3.14
14	63.00	22641	14.69
15	64.05	21756	14.11
16	65.00	96671	62.71
17	66.05	25346	16.44
18	67.00	3822	2.48
19	68.05	1019	0.66
20	69.00	4719	3.06
21	70.05	1412	0.92
22	71.00	2980	1.93
23	72.05	1155	0.75
24	73.05	5382	3.49
25	74.05	5353	3.47
26	75.05	11419	7.41
27	76.05	22124	14.35
28	77.05	107779	69.91
29	78.05	21921	14.22
30	79.05	16489	10.70
31	80.00	3681	2.39
32	81.05	1864	1.21
33	82.25	924	0.60
34	83.15	3723	2.41
35	84.15	1443	0.94
36	85.05	2051	1.33
37	86.15	1681	1.09
38	87.05	8608	5.58
39	88.15	2907	1.89
40	89.10	12663	8.21
41	90.15	13998	9.08
42	91.10	41100	26.66
43	92.15	62683	40.66
44	93.10	154162	100.00
45	94.05	17651	11.45
46	95.05	3385	2.20
47	96.05	989	0.64
48	97.10	1580	1.02
49	98.15	1053	0.68
50	99.15	2366	1.53
51	100.15	2234	1.45
52	101.10	8564	5.56
53	102.10	8698	5.64
54	103.10	16996	11.02
55	104.10	12300	7.98
56	105.15	11228	7.28
57	106.10	50300	32.63
58	107.10	5321	3.45
59	108.05	612	0.40
60	109.20	420	0.27
61	110.15	351	0.23
62	111.05	1400	0.91
63	112.15	1404	0.91
64	113.15	11033	7.16
65	114.15	5041	3.27
66	115.10	12935	8.39
67	116.15	5462	3.54
68	117.10	16015	10.39
69	118.15	3720	2.41
70	119.15	13481	8.74
71	120.10	22258	14.44
72	121.10	2612	1.69
73	122.00	402	0.26
74	123.00	465	0.30
75	124.00	318	0.21
76	125.05	682	0.44
77	126.10	1628	1.06
78	127.15	3814	2.47
79	128.10	4290	2.78
80	129.05	2828	1.83
81	130.05	3826	2.48
82	131.05	3618	2.35
83	132.10	2506	1.63
84	133.15	2019	1.31
85	134.10	1935	1.26
86	135.15	1998	1.30
87	136.15	460	0.30
88	137.15	549	0.36
89	138.15	1141	0.74
90	139.10	7617	4.94
91	140.15	5418	3.51
92	141.10	6183	4.01
93	142.10	1927	1.25
94	143.10	2729	1.77
95	144.10	2178	1.41
96	145.10	1897	1.23
97	146.05	454	0.29
98	146.95	500	0.32
99	150.05	786	0.51
100	151.05	2431	1.58
101	152.05	3644	2.36
102	153.05	3889	2.52
103	154.00	6706	4.35
104	155.05	3892	2.52
105	156.00	8926	5.79
106	157.00	3092	2.01
107	157.95	2315	1.50
108	159.10	1986	1.29
109	160.05	340	0.22
110	161.20	418	0.27
111	164.15	677	0.44
112	165.15	2069	1.34
113	166.15	6853	4.45
114	167.10	14562	9.45
115	168.10	8484	5.50
116	169.00	4481	2.91
117	170.00	1841	1.19
118	171.00	1369	0.89
119	172.10	1559	1.01
120	173.00	729	0.47
121	174.20	388	0.25
122	175.15	841	0.55
123	176.15	370	0.24
124	177.15	970	0.63
125	178.15	1262	0.82
126	179.15	1633	1.06
127	180.10	3102	2.01
128	181.05	2696	1.75
129	182.05	1426	0.93
130	183.05	2231	1.45
131	184.00	980	0.64
132	184.95	694	0.45
133	186.10	410	0.27
134	187.10	532	0.35
135	188.10	255	0.17
136	189.15	1753	1.14
137	190.15	529	0.34
138	191.10	444	0.29
139	192.10	470	0.30
140	193.15	1360	0.88
141	194.10	4385	2.84
142	195.15	10674	6.92
143	196.10	35916	23.30
144	197.05	8941	5.80
145	198.00	1698	1.10
146	199.05	310	0.20
147	200.00	326	0.21
148	201.15	862	0.56
149	202.10	274	0.18
150	203.00	390	0.25
151	204.05	310	0.20
152	205.15	550	0.36
153	206.20	212	0.14
154	207.10	385	0.25
155	208.15	1177	0.76
156	209.15	3487	2.26
157	210.10	8828	5.73
158	211.10	5748	3.73
159	212.10	2607	1.69
160	213.10	1186	0.77
161	214.05	577	0.37
162	214.90	375	0.24
163	215.90	209	0.14
164	217.00	394	0.26
165	218.00	215	0.14
166	219.00	703	0.46
167	220.00	255	0.17
168	221.00	273	0.18
169	222.00	238	0.15
170	223.15	1315	0.85
171	224.10	16098	10.44
172	225.05	2490	1.62
173	226.05	319	0.21
174	227.10	310	0.20
175	228.10	206	0.13
176	229.10	348	0.23
177	230.10	226	0.15
178	231.10	209	0.14
179	235.10	322	0.21
180	236.15	477	0.31
181	237.10	1633	1.06
182	238.15	638	0.41
183	239.15	2349	1.52
184	240.15	311	0.20
185	243.20	202	0.13
186	244.00	242	0.16
187	245.00	503	0.33
188	246.05	378	0.25
189	247.15	1312	0.85
190	248.20	530	0.34
191	249.20	220	0.14
192	253.10	489	0.32
193	255.20	214	0.14
194	256.15	308	0.20
195	257.15	492	0.32
196	258.15	536	0.35
197	259.15	1450	0.94
198	260.15	618	0.40
199	261.25	298	0.19
200	269.15	355	0.23
201	270.15	1225	0.79
202	271.15	2364	1.53
203	272.10	7778	5.05
204	273.10	10260	6.66
205	274.10	16420	10.65
206	275.10	10938	7.10
207	276.10	31231	20.26
208	277.05	7306	4.74
209	278.10	5727	3.71
210	279.05	1100	0.71
211	283.10	228	0.15
212	284.15	644	0.42
213	285.15	2446	1.59
214	286.10	3465	2.25
215	287.15	6826	4.43
216	288.15	9503	6.16

2 / 3

Mass chart of compound 21..

03-Jan-07 20:25

#	m/z	Abs. In	Rel. Int.	#	m/z	Abs. In	Rel. Int.	#	m/z	Abs. In	Rel. Int.
217	289.10	12778	8.29	228	315.15	2664	1.73	239	404.25	1190	0.77
218	290.10	4220	2.74	229	316.15	1527	0.99	240	405.25	972	0.63
219	291.10	3932	2.55	230	317.15	5175	3.36	241	406.25	12764	8.28
220	292.05	1076	0.70	231	318.05	1735	1.13	242	407.25	14784	9.59
221	293.10	482	0.31	232	319.15	1207	0.78	243	408.25	34663	22.48
222	302.10	201	0.13	233	320.00	447	0.29	244	409.25	11515	7.47
223	305.25	1045	0.68	234	329.00	218	0.14	245	410.25	69323	44.97
224	306.30	410	0.27	235	363.50	415	0.27	246	411.20	19482	12.64
225	312.15	298	0.19	236	364.50	249	0.16	247	412.25	14909	9.67
226	313.15	1136	0.74	237	375.50	222	0.14	248	413.20	3940	2.56
227	314.15	1388	0.90	238	393.50	265	0.17	249	414.20	746	0.48

Mass chart of compound 21.

10. Shaaban, S.; Zarrouk, A.; Vervandier-Fasseur, D.; Al-Faiyz, Y.S.; El-Sawy, H.; Althagafi, I.; Andreoletti, P.; Cherkaoui-Malki, M. Cytoprotective organoselenium compounds for oligodendrocytes. *Arabian Journal of Chemistry* **2021**, *14*, 103051.
23. El-Senduny, F.F.; Shabana, S.M.; Rösel, D.; Brabek, J.; Althagafi, I.; Angeloni, G.; Manolikakes, G.; Shaaban, S. Urea-functionalized organoselenium compounds as promising anti-HepG2 and apoptosis-inducing agents. *Future Medicinal Chemistry* **2021**, *13*, 1655-1677.
24. Shaaban, S.; Shabana, S.M.; Al-Faiyz, Y.S.; Manolikakes, G.; El-Senduny, F.F. Enhancing the chemosensitivity of HepG2 cells towards cisplatin by organoselenium pseudopeptides. *Bioorg Chem* **2021**, *109*, 104713, doi:10.1016/j.bioorg.2021.104713.
28. Shaaban, S.; Ashmawy, A.M.; Negm, A.; Wessjohann, L.A. Synthesis and biochemical studies of novel organic selenides with increased selectivity for hepatocellular carcinoma and breast adenocarcinoma. *Eur J Med Chem* **2019**, *179*, 515-526, doi:10.1016/j.ejmech.2019.06.075.
29. Shaaban, S.; Negm, A.; Ashmawy, A.M.; Ahmed, D.M.; Wessjohann, L.A. Combinatorial synthesis, in silico, molecular and biochemical studies of tetrazole-derived organic selenides with increased selectivity against hepatocellular carcinoma. **2016**, *122*, 55-71,doi:https://doi.org/10.1016/j.ejmech.2016.06.005.
48. Shaaban, S.; Negm, A.; Sobh, M.A.; Wessjohann, L.A. Expeditious Entry to Functionalized Pseudo-peptidic Organoselenide Redox Modulators via Sequential Ugi/SN Methodology. *Anticancer Agents Med Chem* **2016**, *16*, 621-632, doi:10.2174/1871520615666150916092035.
